# Solvent-dependent chemoselective synthesis of different isoquinolinones mediated by the hypervalent iodine(III) reagent PISA

**DOI:** 10.3762/bjoc.20.167

**Published:** 2024-08-07

**Authors:** Ze-Nan Hu, Yan-Hui Wang, Jia-Bing Wu, Ze Chen, Dou Hong, Chi Zhang

**Affiliations:** 1 State Key Laboratory of Elemento-Organic Chemistry, The Research Institute of Elemento-Organic Chemistry, College of Chemistry, Nankai University, 94 Weijin Road, Tianjin 300071, P. R. China

**Keywords:** annulation, C–H amination, hypervalent iodine reagent, iodine(III), isoquinolinone, solvent-dependence

## Abstract

Isoquinolinone is an important heterocyclic framework in natural products and biologically active molecules, and the efficient synthesis of this structural motif has received much attention in recent years. Herein, we report a (phenyliodonio)sulfamate (PISA)-mediated, solvent-dependent synthesis of different isoquinolinone derivatives. The method provides highly chemoselective access to 3- or 4-substituted isoquinolinone derivatives by reacting *o*-alkenylbenzamide derivatives with PISA in either acetonitrile or wet hexafluoro-2-isopropanol.

## Introduction

**Figure 1:**
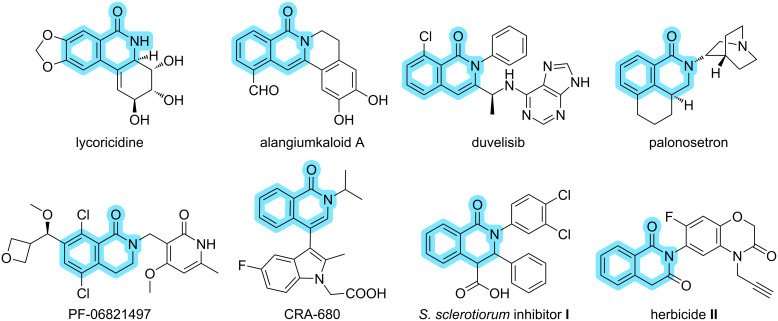
Selected natural products, pharmaceuticals, and biologically active compounds having an isoquinolinone scaffold.

Isoquinolinone is an important heterocyclic structure found in many natural products and biologically active compounds, including pharmaceuticals [1]. For instance, lycoricidine, found in the medicinal plant *Lycoris radiata*, may inhibit the MCPyV LT protein activity and thus block cancer formation [2]. Alangiumkaloids A, an isoquinolinone alkaloid isolated from *Alangium salviiforlium*, was reported to exhibit cytotoxic activity against cancer cells [3]. In 2018, duvelisib, a dual inhibitor of phosphoinositide-3 kinases, was firstly approved by the FDA for the treatment of adult patients with relapsed or refractory chronic lymphocytic leukaemia or small lymphocytic lymphoma [4]. Palonosetron is a key component of Akynzeo^®^, used for the prevention of acute and delayed nausea and vomiting of cancer patients who are receiving chemotherapy [5]. As an active compound, PF-06821497 showed potent tumor growth inhibition in mouse xenograft models [6]. CRA-680 was efficacious in both a house dust mouse model of allergic lung inflammation and a guinea pig allergen challenge model of lung inflammation [7]. In addition, isoquinolinone compounds not only prevent and control plant diseases but also have some herbicidal activity. Compound **I** showed good inhibitory activity against *Sclerotinia sclerotiorum* on detached oilseed rape leaves [8], and compound **II** has excellent herbicidal activity against dicot weeds, such as *Zinnia elegans* Jacq. and *Abutilon theophrasti* Medicus (Figure 1) [9]. Therefore, in recent years, isoquinolinone derivatives have attracted considerable attention, and successful synthetic methods involving the isoquinolinone framework have been reported.

A number of appealing methods for the synthesis of isoquinolinone scaffolds using transition metal reagents, including cobalt [10], copper [11], rhodium [12–14], palladium [15–17], silver [18], and gold [19] catalysts, have been reported. However, compared to the widespread use of metal catalysts, the synthesis of isoquinolinone scaffolds mediated by environmentally friendly nonmetallic reagents as an attractive alternative is less developed. In 2014, Antonchick and Manna firstly reported the synthesis of a series of 3,4-diaryl-substituted isoquinolinone derivatives through oxidative annulation between alkynes and benzamide derivatives using iodobenzene as a catalyst and peracetic acid as a terminal oxidant [20]. Recently, Kočovský et al. disclosed a method employing 2-methylbenzamide and benzonitrile to yield 3-aryl-substituted isoquinolinone derivatives in the presence of *n*-butyllithium [21]. On the other hand, the intramolecular oxidative cyclization is also a viable option for the preparation of isoquinolinone derivatives. In 2020, two reports have been published on the conversion of alkyne-tethered *N*-alkoxybenzamides to isoquinolinones by intramolecular oxidative annulation, either electrochemically or using the hypervalent iodine reagent phenyliodine(III) diacetate (PIDA) [22–23]. And more recently, Du and our group have developed a method for the chemoselective cycloisomerization of *o*-alkenylbenzamides to 3-arylisoquinolinones, using PhIO as oxidant in combination with a catalytic amount of trimethylsilyl trifluoromethanesulfonate [24]. Although considerable progress has been made in the synthesis of isoquinolinone derivatives, there is still the need to develop chemoselective strategies based on easily adjustable factors, such as solvent selection to obtain 3- or 4-substituted isoquinolinone derivatives.

**Scheme 1:**
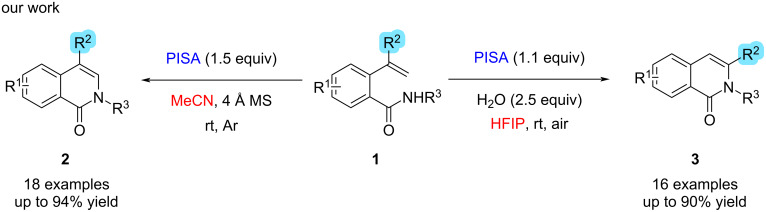
Chemoselective and PISA-mediated, solvent-controlled synthesis of different isoquinolinone derivatives **2** and **3**.

In 2018, our group has reported the zwitterionic water-soluble hypervalent iodine reagent (phenyliodonio)sulfamate (PISA). In water, PISA is strongly acidic, and the pH value can reach 2.05 in a saturated aqueous solution. With PISA, various indoles have been synthesized via C–H amination of 2-alkenylanilines involving an aryl migration–intramolecular cyclization cascade with excellent chemoselectivity in aqueous CH_3_CN [25]. Herein, as part of our continuing studies of heterocyclic scaffold synthesis mediated by hypervalent iodine reagents, we present the solvent-dependent chemoselective synthesis of a series of isoquinolinones mediated by PISA using 2-alkenylbenzamide derivatives as substrates (Scheme 1).

## Results and Discussion

We began by exploring the reaction of *N*-methoxy-2-(prop-1-en-2-yl)benzamide (**1a**) with PISA (1.5 equiv) in anhydrous acetonitrile at room temperature under argon atmosphere. 4-Methylisoquinolinone **2a** was the sole product in the reaction, with a yield of 86% in 20 minutes (Table 1, entry 1). Encouraged by this result, we added additives to the reaction with the aim of further increasing the chemical yield of **2a**. When 1.5 equivalents of water were added to the reaction, the yield of **2a** dropped to 79% (Table 1, entry 2). The reduced yield of **2a** indicated that this reaction could benefit from a dry solvent. Therefore, 4 Å molecular sieves or anhydrous sodium sulfate were added to the reaction mixture. When 4 Å molecular sieves were added, the yield of **2a** slightly increased to 88%, which was superior to using Na_2_SO_4_ (Table 1, entries 3 and 4). Next, different commercially available iodanes were employed as oxidants, such as PIDA, phenyliodine(III) bis(trifluoroacetate) (PIFA), *N*-tosyliminobenzyliodinane (PhINTs), iodosylbenzene (PhIO), and Koser’s reagent (HTIB) (Table 1, entries 5–9). Of the reagents tested, PISA gave the best result. Furthermore, screening of different solvents showed that acetonitrile was superior for this reaction (Table S1, Supporting Information File 1). Based on the screening results, the optimized reaction conditions for the conversion of **1a** to the 4-subsitituted isoquinolinone **2a** were as follows: 1.5 equivalents of PISA and 4 Å MS in anhydrous CH_3_CN (0.1 M of **2a**) under argon atmosphere at room temperature for 20 min.

**Table 1:** Optimization of the reaction conditions for the synthesis of 4-substituted isoquinolinone **2a**^a^.

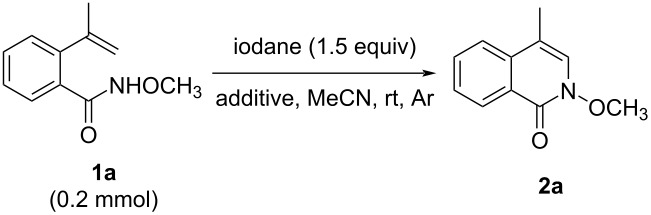			

entry	iodane	additive	yield of **2a**, %^b^

1	PISA	—	86
2	PISA	H_2_O (1.5 equiv)	79
**3**	**PISA**	**4 Å MS**	**88**
4	PISA	Na_2_SO_4_	81
5	PIDA	4 Å MS	52
6	PIFA	4 Å MS	77
7	PhINTs	4 Å MS	61
8	PhIO	4 Å MS	0
9	HTIB	4 Å MS	52

^a^Reactions were carried out using **1a** (0.2 mmol), hypervalent iodine reagent (1.5 equiv), and 4 Å MS (7.6 mg) in MeCN (2.0 mL) at room temperature under argon atmosphere. ^b^Isolated yield.

**Scheme 2:**
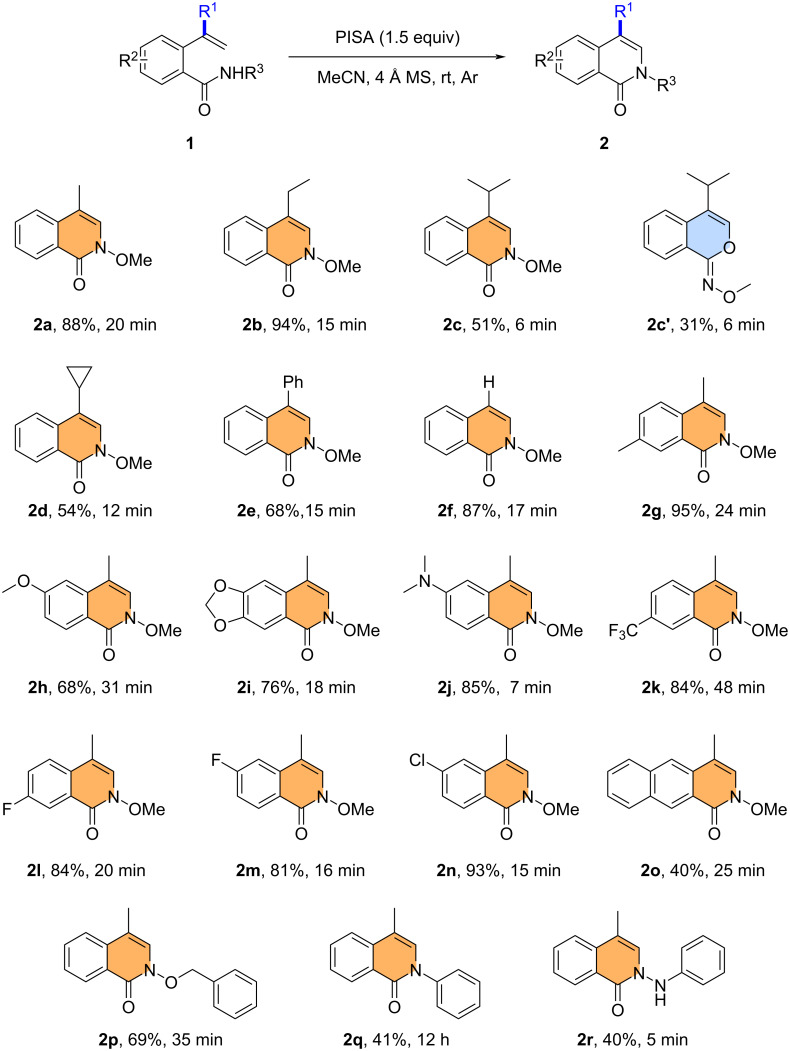
Substrate scope for the synthesis of 4-substituted isoquinolinones **2**. Reaction conditions: **1** (0.3 mmol), PISA (1.5 equiv), and 4 Å MS (10 mg) in MeCN (3.0 mL).

With the optimal reaction conditions in hand, we explored the scope of the method by testing various 2-alkenylbenzamide derivatives **1** (Scheme 2). When R^1^ was ethyl, isopropyl, cyclopropyl, phenyl, or hydrogen, respectively, the intramolecular amination smoothly gave the corresponding 4-substituted isoquinolinone products **2b**,**c**,**d**–**f** in 51–94% yield. Notably, when **1c** was used as the substrate, the cycloisomerization product **2c'** was observed in 31% yield besides **2c** in 51% yield. Additional experiments were then carried out using *N*-methoxy-2-(prop-1-en-2-yl)benzamide with different substituents R^2^. Both electron-donating (methyl, alkoxy, dimethylamino) and electron-withdrawing substituents (fluoro, chloro, trifluoromethyl) were well tolerated on the phenyl ring and gave the desired products **2g**–**n** in 68–95% yield. Furthermore, a substrate containing a naphthalene moiety was also compatible with the reaction conditions, giving the corresponding ring-fused product **2o** in 40% yield. It is worth noting that when the *N*-substituent was phenyl, benzyloxy, or phenylamino, the reaction still proceeded well, and the corresponding products **2p**,**q**,**r** were obtained in 69%, 41%, and 40% yield, respectively.

**Scheme 3:**
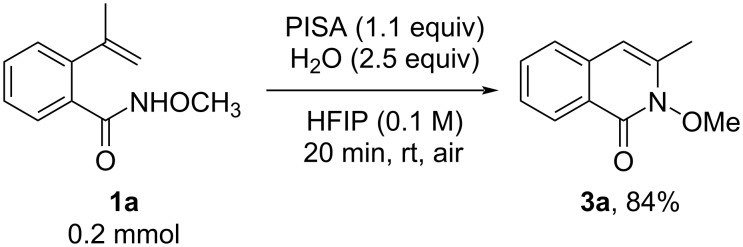
Optimal reaction conditions for the synthesis of 3-substituted isoquinolinone **3a**.

Interestingly, when screening solvents for the synthesis of 4-methylisoquinolinones, we were surprised to discover that when hexafluoro-2-propanol (HFIP) was used as the solvent, 3-methylisoquinolinone **3a**, an isomer of **2a**, was formed in 51% yield. Apparently, the change of solvent resulted in a different chemoselectivity of the reaction. With this in mind, we investigated the reaction conditions (see Supporting Information File 1 for details) and obtained the optimal conditions for the synthesis of 3-methylisoquinolinone as follows: reacting 1.1 equivalents of PISA in HFIP (0.1 M of **1a**) containing 2.5 equivalents of H_2_O at room temperature for 20 minutes (Scheme 3).

**Scheme 4:**
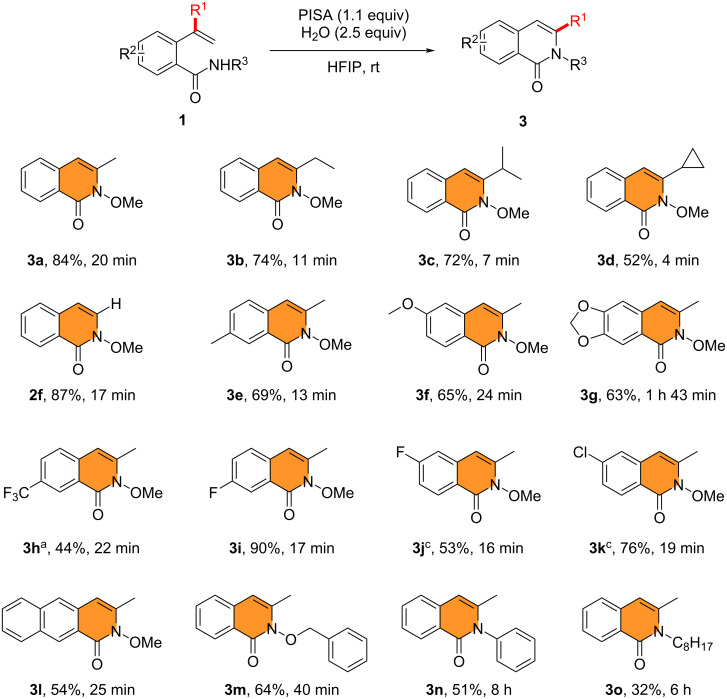
Substrate scope for the synthesis of 3-substituted isoquinolinones **3**. Reaction conditions: **1** (0.3 mmol), PISA (1.1 equiv), and H_2_O (2.5 equiv) in HFIP (3.0 mL) at room temperature. Isolated yield is stated. ^a^The yield of **2k** was 56%. ^b^The yield of **2m** was 24%. ^c^The yield of **2n** was 11%.

The general applicability of PISA in wet HFIP solvent was studied. When R^1^ was ethyl, isopropyl, cyclopropyl, or hydrogen, respectively, the substrates could be successfully converted to the products **3b**–**d** and **2f** in 52–87% yield with this method. In addition, a good or high yield of 3-methylisoquinolinones **3e**–**k**, with different substituents on the phenyl ring, was also obtained. It is worth noting that when an electron-withdrawing group (trifluoromethyl, fluoro, chloro) was located on the phenyl ring, various amounts of 4-substituted isoquinolinone derivatives **2k**,**m**,**n** were observed along with the formation of **3h**,**j**,**k**, respectively. Furthermore, a substrate containing a naphthalene unit was also compatible with the reaction conditions, leading to **3l**. In particular, the presence of diverse nitrogen protecting groups, such as benzyloxy, phenyl, and alkyl, did not affect the smooth reaction, affording **3m**–**o** in a moderate yield of 32–64% (Scheme 4). Just by changing the solvent of the reaction, we were able to obtain the isomeric 3- or 4-substituted isoquinolinone derivatives with excellent chemoselectivity. These interesting findings led us to investigate the reaction mechanism.

**Scheme 5:**
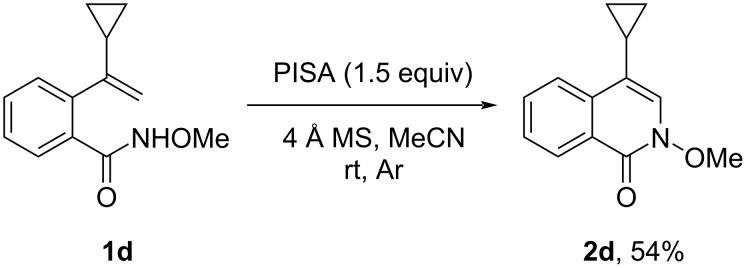
Control experiment to test for radical intermediates.

To gain insight into the mechanism and chemoselectivity of the reactions above, we performed a control experiment. With acetonitrile as the solvent, a radical clock experiment was carried out with **1d** under the optimal reaction conditions, resulting in the formation of **2d** in 54% yield, and no cyclopropyl ring opening products were observed. This result suggested that no radical intermediates were generated during the reaction (Scheme 5).

**Scheme 6:**
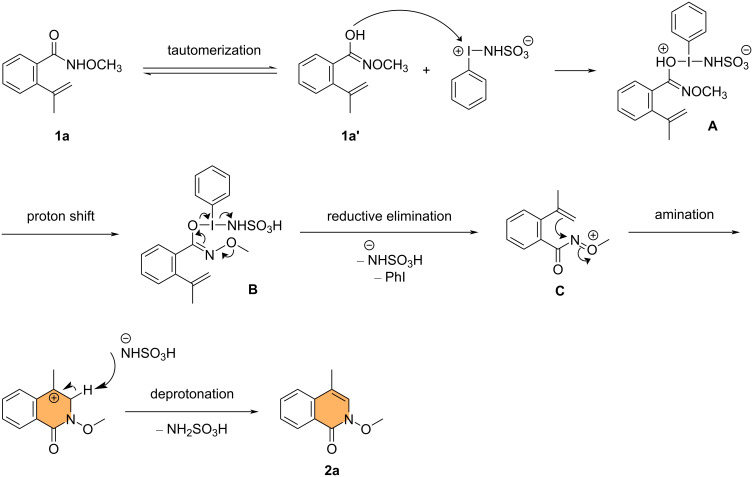
Proposed mechanism for the reaction between **1a** and PISA in anhydrous acetonitrile.

According to the aforementioned control experiment and literature precedents, we proposed a mechanism for the formation of 4-substituted isoquinolinone derivatives, including **2a**. The reaction begins by tautomerization of **1a**, and PISA undergoes an electrophilic reaction with **1a'** to form the iodane intermediate **A**. The iodane **A** then undergoes a proton shift to provide intermediate **B**. Intermediate **B** collapses via reductive elimination to give nitrenium ion **C**, along with the release of iodobenzene and sulfamate. Finally, nucleophilic attack of the olefin moiety of **C** on the electrophilic nitrogen atom, followed by the deprotonation with sulfamate, gives the 4-substituted isoquinolinone derivative **2a** (Scheme 6).

**Scheme 7:**
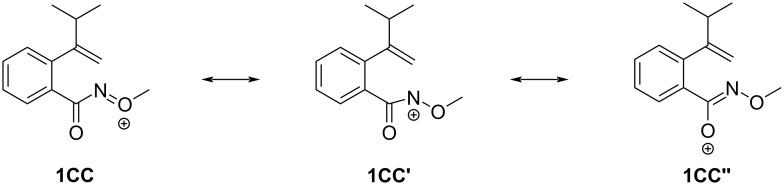
Two other resonance structures of the intermediate **1CC**.

Looking into the formation of **2c'** from **1c** (Scheme 2), two other resonance structures for the initially formed intermediate **1CC**, namely **1CC'** and **1CC''**, are shown in Scheme 7. The oxygen atom in the amide motif of the substrate **1c** may act as an electrophilic center, forming a C–O bond with the alkenyl group to give the isochromen-1-one oxime product **2c'**.

**Scheme 8:**
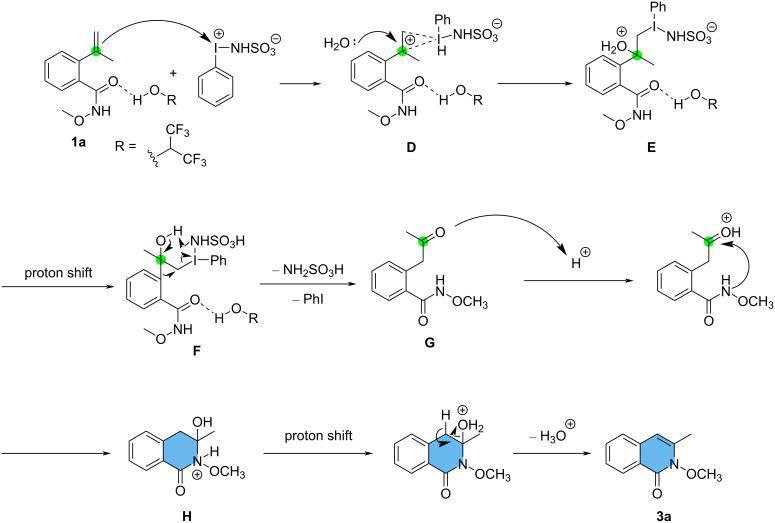
Proposed mechanism for the reaction between **1a** and PISA in wet HFIP.

When wet HFIP was used as the solvent, the reaction followed a different pathway. HFIP, a strong hydrogen bonding donor [26–28], interacts with the amide moiety of the substrate, and thus preventing the possible interaction between the amide moiety and PISA, as opposed to CH_3_CN. The olefin moiety of the complex then interacts with the exposed central iodine(III) atom in PISA [25], forming the intermediate **D**. Similar cyclic iodonium intermediates were also postulated for the synthesis of benzofuran derivatives from styrene derivatives by iodane reagents [29–30]. Subsequently, intermediate **D** is attacked by H_2_O at the benzylic carbon atom to afford intermediate **E**. Intramolecular proton shift occurs, generating the intermediate **F**, which undergoes phenyl migration and reductive elimination, along with the release of iodobenzene and sulfamic acid. Cyclization of protonated **G** takes place to afford the intermediate **H**. Finally, release of water and β-proton elimination produces the rearranged product **3a** (Scheme 8).

## Conclusion

In summary, we reported the efficient synthesis of isoquinolinone derivatives using a PISA-mediated methodology that chemoselectively yielded 3- or 4-substituted isoquinolinone derivatives by simply adjusting the solvent. When acetonitrile was used, the 4-substituted isoquinolinone derivatives were the reaction products, whereas hexafluoro-2-propanol led to 3-substituted isoquinolinones. The solvent-dependent chemoselective synthesis of isoquinolinone derivatives is interesting and unprecedented. Further research on synthetic utility of PISA, a unique zwitterionic hypervalent iodine(III) reagent, is underway in our laboratory.

## Supporting Information

File 1 Experimental details, optimization studies, compound characterization data, and spectra.

## Data Availability

All data that supports the findings of this study is available in the published article and/or the supporting information to this article.
