# Supplementary material for: Solvent-dependent chemoselective synthesis of different isoquinolinones mediated by the hypervalent iodine(III) reagent PISA
Source: Beilstein J Org Chem. 2024 Aug 7;20:1914–21. doi: 10.3762/bjoc.20.167 (PMC11318619; doi:10.3762/bjoc.20.167)
Supplement: File 1 — Experimental details, optimization studies, compound characterization data, and spectra. [file Beilstein_J_Org_Chem-20-1914-s001.pdf]

BEILSTEIN JOURNAL OF ORGANIC CHEMISTRY

Supporting Information

for

Solvent-dependent chemoselective synthesis of different isoquinolinones mediated by the hypervalent iodine(III) reagent PISA

Ze-Nan Hu, Yan-Hui Wang, Jia-Bing Wu, Ze Chen, Dou Hong and Chi Zhang

Beilstein J. Org. Chem. **2024,** *20,* 1914–1921. doi:10.3762/bjoc.20.167

Experimental details, optimization studies, compound characterization data, and spectra

License and Terms: This is a supporting information file under the terms of the Creative Commons Attribution License (https://creativecommons.org/licenses/by/4.0). Please note that the reuse, redistribution and reproduction in particular requires that the author(s) and source are credited and that individual graphics may be subject to special legal provisions.
The license is subject to the *Beilstein Journal of Organic Chemistry* terms and conditions: (https://www.beilstein-journals.org/bjoc/terms)

I. General experimental

Some chemicals were used as received from commercial suppliers without further purification. Other chemicals were prepared by the reported procedures. All solvents before use were dried and purified according to the standard procedure. NMR spectra were recorded for ^{1}H NMR (400 MHz) and ^{13}C NMR (100 MHz) using TMS as an internal standard and Bruker AV 400 as an instrument. The following abbreviations were used to describe peak patterns where appropriate: singlet (s), doublet (d), triplet (t), multiplet (m). High-resolution mass spectroscopy (HRMS) was recorded on a high-resolution ESI–FTICR mass spectrometer (Varian 7.0 T). IR spectra were recorded with a FT-IR Bruker EQUINOX55 spectrometer in KBr pellets. Melting points were determined on a RY-1 electrothermal micromelting point apparatus.

II. Experimental procedures

General procedure (I) for the synthesis of benzamide 1a and 1f–o

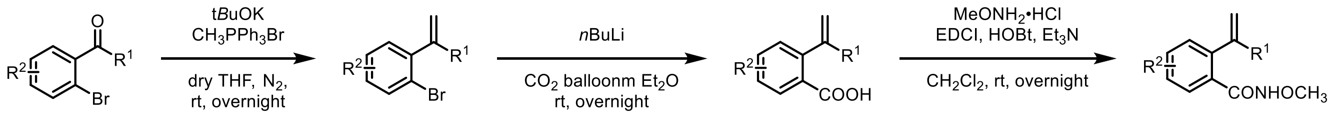

Benzamide **1a** and **1f**–**o** were synthesized according to literature procedure.[1] For example, to the mixture of methyl triphenylphosphonium bromide (3 equiv, 15 mmol) and *t*-BuOK (3 equiv, 15 mmol) was added anhydrous THF (20.0 mL) and stirred at rt for 0.5 hour under argon, then a solution of **A** (1 equiv, 5 mmol) in THF (5.0 mL) was added dropwise. The resulting reaction mixture was stirred overnight at room temperature and quenched with saturated NH_4Cl solution. The organic layer was separated and the aqueous layer was extracted with EtOAc (2 × 50 mL). The combined organic layers were dried over Na_2SO_4, filtered, and concentrated in vacuo. The residue was purified by flash chromatography on silica gel using petroleum/EtOAc as eluent to yield **B** as a colorless oil.

To the solution of **B** (1.0 equiv, 5 mmol) in Et_2O was added dropwise *n*-BuLi (1.2 equiv, 6 mmol) at 0 °C, after 30 min, to the lithiated mixture was insert a CO_2 balloon. The mixture was allowed to warm to room temperature and stirred for overnight. The reaction was quenched with

saturated $NaHCO_3$ (15 mL) solution and washed with Et_2O (2 × 10 mL). The aqueous layer was then acidified with 2 N HCl to pH 1 and extracted with Et_2O (3 × 20 mL). The combined organic layers were dried over Na_2SO_4, filtered, and concentrated under reduced pressure to give **C** as a colorless solid.

To a solution of **C** (1 equiv, 2 mmol) in DCM (5 mL), were added NH_2OMe•HCl (1.5 equiv, 3 mmol), triethylamine (5 equiv, 10 mmol). After stirring for 5 min, 1-(3-dimethylaminopropyl)-3-ethylcarbodiimide hydrochloride (EDCI, 1.5 equiv, 3 mmol), 1-hydroxybenzotriazole (HOBt, 1.5 equiv, 3 mmol) were added. The reaction mixture was stirred overnight at room temperature. After completion of the reaction (monitored by TLC), water and DCM were added to the reaction mixture. The organic phase was washed with aqueous HCl (1.0 M), saturated $NaHCO_3$ solution, brine, dried over Na_2SO_4, filtered and concentrated in vacuo. The residue was purified by silica gel column chromatography to afford substrate **1** as a white solid.

General procedure (II) for the synthesis of benzamide 1b–e

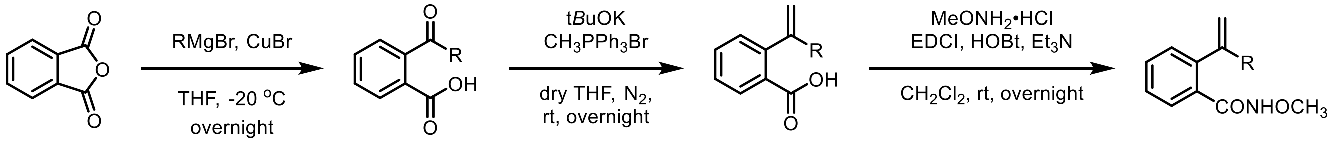

Phthalic anhydride (1.0 equiv, 10 mmol), copper bromide (0.07 equiv, 0.7 mmol) and anhydrous THF (15 mL) were added to a flame-dried flask and cooled to −20 °C under N_2 atmosphere. RMgBr (1.1 equiv, 11 mmol) was added dropwise to the mixture over 1 h. The reaction mixture was stirred overnight at −20 °C, then allowed to warm to room temperature, quenched with water, basified with aqueous NaOH (3.0 M) until pH 13–14 and washed with diethyl ether. The resulting aqueous phase was acidified with aqueous HCl (2.0 M) until pH 1–2 and extracted twice with ethyl acetate. The combined organic layers were washed with water, brine, dried over Na_2SO_4, filtered and concentrated in vacuo. The crude product was dissolved in DCM and the solid was filtered off. The crude benzoic acid derivative was used for the next step without further purification. From benzoic acid derivative, the corresponding benzamide derivate was synthesized by the same method **1** (**method A**).

General procedure (III) for the synthesis of benzamide 1p–r

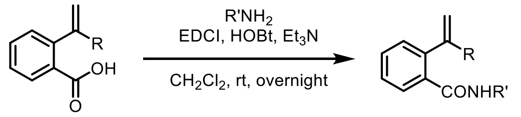

To a solution of **C** (1 equiv, 2 mmol) in DCM (5 mL), were added R'NH_2 (1.5 equiv, 3 mmol), triethylamine (5 equiv, 10 mmol). After stirring for 5 min, 1-(3-dimethylaminopropyl)-3-ethylcarbodiimide hydrochloride (EDCI, 1.5 equiv, 3 mmol), 1-hydroxybenzotriazole (HOBt, 1.5 equiv, 3 mmol) were added. The reaction mixture was stirred overnight at room temperature. After completion of the reaction (monitored by TLC), water and DCM were added to the reaction mixture. The organic phase was washed with aqueous HCl (1.0 M), saturated $NaHCO_3$ solution, brine, dried over Na_2SO_4, filtered and concentrated in vacuo. The residue was purified by silica gel column chromatography to afford substrate **1p**–**r** as a white solid.

General procedure (IV) for the synthesis of 4-substituted isoquinolin-1(2*H*)-one

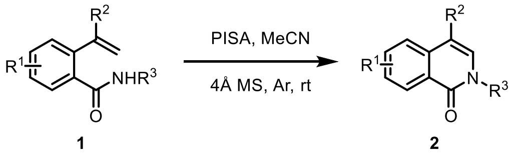

To a solution of PISA (0.45 mmol, 1.5 equiv), 4 Å MS (7.6 mg) in dry MeCN (3 mL, 0.1 M), were added **1** (1 equiv, 0.3 mmol) in dry MeCN under N_2 atmosphere. The reaction mixture was stirred at room temperature. After completion of the reaction (monitored by TLC), $NaHCO_3$ solution, saturated $Na_2S_2O_3$ solution and EtOAc were added to the reaction mixture. The organic phase was washed with brine, dried over Na_2SO_4, filtered and concentrated in vacuo. The residue was purified by silica gel column chromatography to afford product **2**.

General procedure (V) for the synthesis of 3-substituted isoquinolin-1(2*H*)-one

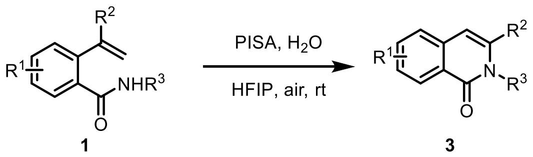

To a solution of **1** (0.3 mmol), H_2O (0.75 mmol, 2.5 equiv) in HFIP (3 mL, 0.1 M), were added PISA (0.33 mmol, 1.1 equiv). The reaction mixture was stirred at room temperature. After

completion of the reaction (monitored by TLC), $NaHCO_3$ solution, saturated $Na_2S_2O_3$ solution and EtOAc were added to the reaction mixture. The organic phase was washed with brine, dried over Na_2SO_4, filtered and concentrated in vacuo. The residue was purified by silica gel column chromatography to afford product **3**.

III. Optimization studies

Table S1: Optimization of the reaction conditions for the synthesis of 4-methylisoquinolinone.[a]

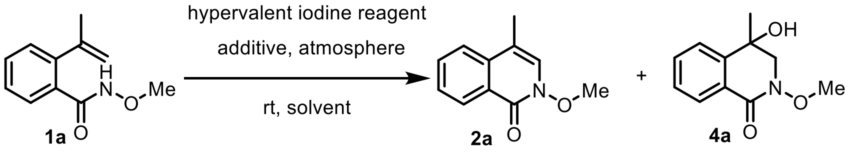

entry	hypervalent iodine reagent	additive	atmosphere	solvent	time	yield (%)[b] 2a	4a
1	PISA	H_2O (1.5 equiv)	air	MeCN	50 min	79	13
2	PISA	H_2O (1.5 equiv)	Ar	MeCN	5 min	80	6
3	PISA	-	Ar	MeCN	20 min	86	8
4	uncrystalized PISA	4Å MS	Ar	MeCN	1 h	88	11
5	uncrystalized PISA	Na_2SO_4 (7.6 mg)	Ar	MeCN	1 h	81	14
6	PhIO	4Å MS	Ar	MeCN	1.5 h	-	-
7	PIFA	4Å MS	Ar	MeCN	20 min	77	-
8	PIDA	4Å MS	Ar	MeCN	20 min	52	28
9	HTIB	4Å MS	Ar	MeCN	20 min	52	-
10	PhINTs	4Å MS	Ar	MeCN	20 min	61	-
11	*p*-TolICl$_2$	4Å MS	Ar	MeCN	20 min	-	-
12	*p*-TolFI$_2$	4Å MS	Ar	MeCN	30 min	79	-
13[c]	IBX	4Å MS	Ar	MeCN	overnight	-	-
14[d]	IBX-KSO$_3$	4Å MS	Ar	MeCN	overnight	-	-
15[e]	uncrystalized PISA	4Å MS	Ar	$CHCl_3$	1 h	67	5
16	uncrystalized PISA	4Å MS	Ar	DCM	1 h	80	-
17	uncrystalized PISA	4Å MS	Ar	DCE	3 h	78	-
18	uncrystalized PISA	4Å MS	Ar	toluene	1.5 h	74	12
19	uncrystalized PISA	4Å MS	Ar	nitrobenzene	50 min	74	-
20	uncrystalized PISA	4Å MS	Ar	Et_2O	1.67 h	76	12
21	uncrystalized PISA	4Å MS	Ar	THF	1.67 h	76	-
22	uncrystalized PISA	4Å MS	Ar	EA	1.67 h	84	-
23[f]	uncrystalized PISA	4Å MS	Ar	DMF	25 min	59	-
24[g]	uncrystalized PISA	4Å MS	Ar	HFIP	1.67 h	0	-
25	uncrystalized PISA	4Å MS	Ar	H_2O	35 min	51	41

[a]Reaction conditions: **1a** (0.2 mmol), hypervalent iodine reagent (1.5 equiv), 4 Å MS (7.6 mg) in solvent (2.0 mL) at rt. [b]Yield of isolated product after column chromatography. [c]The recovery of 1a was 77%. [d]The recovery of 1a was 96%. [e]The recovery of 1a was 21%. [f]The recovery of 1a was 30%. [g]The yield of 3a was 51%.

Table S2: Optimization of the reaction conditions for the synthesis 3-methylisoquinolinone.[a]

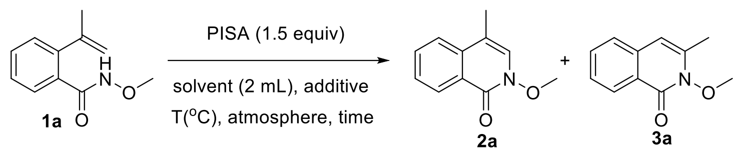

entry	solvent	additive	T(°C)	atmosphere	time	yield (%)[b] 2a	3a
1	HFIP	4Å MS	rt	Ar	1 h	0	51
2	HFIP	-	rt	Ar	20 min	0	55
3	HFIP	-	rt	air	20 min	0	57
4	HFIP	H_2O(1.5 equiv)	rt	air	20 min	0	64
5[c]	HFIP	H_2O(1.5 equiv)	rt	air	13 min	0	62
6[d]	HFIP	H_2O(1.5 equiv)	rt	air	12 min	0	38
7[e]	HFIP	H_2O(1.5 equiv)	rt	air	13 min	0	62
8	HFIP	H_2O(0.5 equiv)	rt	air	12 min	0	61
9	HFIP	H_2O(2.5 equiv)	rt	air	9 min	0	69
10	HFIP	H_2O(4.5 equiv)	rt	air	18 min	0	69
11	HFIP : H_2O = 3 : 1	-	rt	air	30 min	24	37
12	Trifluoroethanol	-	rt	air	20 min	11	54
13	Perfluoro-tert-butanol	-	rt	air	20 min	24	37
14	HFIP	-	0	air	21 min	0	54
15	HFIP	-	40	air	23 min	3	48
16[f]	HFIP	H_2O(2.5 equiv)	rt	air	13 min	0	84

[a]Reaction conditions: 1a (0.2 mmol), PISA (1.5 equiv) in solvent (2.0 mL) at rt. [b]Yield of isolated product after column chromatography. [c]Using PISA recrystallized in water. [d]HFIP was 1 mL, concentration of 1 in the solvent was 0.2 mol/L. [e]HFIP was 4 mL, concentration of 1 in the solvent was 0.05 mol/L. [f]Using 1.1 equiv PISA.

IV. Characterization data

_N_-Methoxy-2-(prop-1-en-2-yl)benzamide (1a) [1]

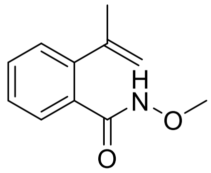

Colorless solid; m.p. 77-79 °C; yield: 68% (EA/PE = 1/4); ^{1}H NMR (400 MHz, CDCl$_3$) δ: 9.91 (s, 1H), 7.36-7.28 (m, 2H), 7.22-7.15 (m, 2H), 5.10 (s, 1H), 4.95 (s, 1H), 3.66 (s, 3H), 2.01 (s, 3H); ^{13}C NMR (100 MHz, CDCl$_3$) δ: 167.1, 144.3, 141.9, 131.0, 130.0, 128.1, 127.9, 126.7, 115.6, 63.2, 23.5.

2-(But-1-en-2-yl)-_N_-methoxybenzamide (1b) [1]

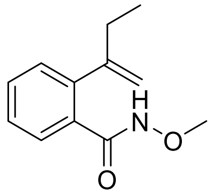

Yellowish oil; yield: 73% (EA/PE = 1/4); ^{1}H NMR (400 MHz, CDCl$_3$) δ: 8.77 (s, 1H), 7.62 (d, J = 6.9 Hz, 1H), 7.46-7.28 (m, 2H), 7.18 (d, J = 7.3 Hz, 1H), 5.23 (s, 1H), 5.09 (s, 1H), 3.81 (s, 3H), 2.38 (d, J = 7.1 Hz, 2H), 1.03 (t, J = 7.1 Hz, 3H); ^{13}C NMR (100 MHz, CDCl$_3$) δ: 167.3, 151.9, 141.5, 130.9, 130.7, 129.2, 128.9, 127.5, 114.3, 64.2, 30.6, 12.4.

_N_-Methoxy-2-(3-methylbut-1-en-2-yl)benzamide (1c) [2]

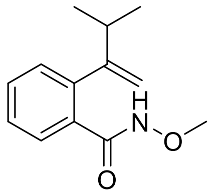

Colorless oil; yield: 56% (EA/PE = 1/3); ^{1}H NMR (400 MHz, CDCl$_3$) δ: 8.74 (s, 1H), 7.69 (d, J = 7.5 Hz, 1H), 7.38 (dtd, J = 24.5, 7.5, 1.4 Hz, 2H), 7.16 (dd, J = 7.5, 1.1 Hz, 1H), 5.26 (s, 1H), 5.10 (s, 1H), 3.83 (s, 3H), 2.62-2.46 (m, 1H), 1.05 (d, J = 6.8 Hz, 6H); ^{13}C NMR (100 MHz, CDCl$_3$) δ: 167.3, 157.3, 141.7, 130.9, 130.8, 129.8, 129.2, 127.6, 113.0, 64.4, 34.9, 21.5.

2-(1-Cyclopropylvinyl)-*N*-methoxybenzamide (1d) [2]

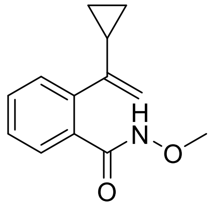

Colorless oil; yield: 70% (EA/PE = 1/3); ^{1}H NMR (400 MHz, $CDCl_3$) δ: 8.88 (s, 1H), 7.73 (d, J = 7.5 Hz, 1H), 7.39 (dtd, J = 22.0, 7.4, 1.2 Hz, 2H), 7.19 (d, J = 7.3 Hz, 1H), 5.18 (s, 1H), 5.03 (s, 1H), 3.86 (s, 3H), 1.65 (ddd, J = 13.4, 8.3, 5.2 Hz, 2H), 0.86-0.72 (m, 2H), 0.50 (d, J = 5.3 Hz, 2H); ^{13}C NMR (100 MHz, CDCl3) δ: 151.9, 139.8, 131.1, 130.7, 129.5, 129.2, 127.8, 112.5, 110.0, 64.3, 17.4, 7.4.

***N*-Methoxy-2-(1-phenylvinyl)benzamide (1e)** [2]

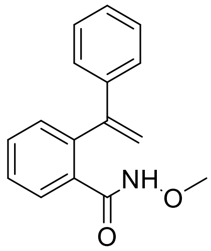

Yellow oil; yield: 20% (EA/PE = 1/1); ^{1}H NMR (400 MHz, $CDCl_3$) δ: 9.71 (s, 1H), 7.36 (d, J = 7.2 Hz, 2H), 7.31-7.09 (m, 7H), 5.62 (s, 1H), 5.26 (s, 1H), 3.20 (s, 3H); ^{13}C NMR (100 MHz, $CDCl_3$) δ: 165.9, 147.9, 140.0, 139.8, 132.4, 130.3, 130.0, 127.9, 127.7, 127.3, 127.2, 126.7, 115.5, 62.7.

***N*-Methoxy-2-vinylbenzamide (1f)** [3]

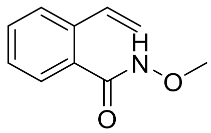

Colorless oil; yield: 83% (EA/PE = 1/8); ^{1}H NMR (400 MHz, $CDCl_3$) δ: 8.35 (s, 1H), 7.59 (d, J = 8.0 Hz, 1H), 7.44 (s, 2H), 7.10-6.97 (m, 1H), 5.75 (d, J = 17.2 Hz, 1H), 5.39 (d, J = 10.8 Hz, 1H), 3.90 (s, 3H); ^{13}C NMR (100 MHz, $CDCl_3$) δ: 167.2, 136.4, 133.8, 131.6, 130.7, 127.7, 127.6, 126.2, 117.2, 64.5.

N-Methoxy-5-methyl-2-(prop-1-en-2-yl)benzamide (1g) [2]

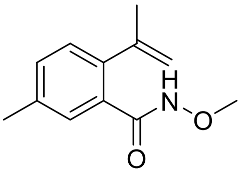

Colorless solid; m.p. 84-86 °C; yield: 80% (EA/PE = 1/4); ^{1}H NMR (400 MHz, $CDCl_3$) δ: 8.65 (s, 1H), 7.43 (s, 1H), 7.22 (d, J = 7.6 Hz, 1H), 7.12 (d, J = 8.0 Hz, 1H), 5.23 (s, 1H), 5.07 (s, 1H), 3.84 (s, 3H), 2.35 (s, 3H), 2.08 (s, 3H); ^{13}C NMR (100 MHz, $CDCl_3$) δ: 167.6, 145.4, 139.0, 137.4, 131.5, 130.6, 129.3, 128.5, 116.3, 64.1, 24.2, 20.8; IR (KBr); 3126, 2935, 1657, 1507, 1444, 1310, 1042, 968, 892, 828 cm^{-1}; HRMS (ESI): calcd for $C_{12}H_{15}NO_2 \cdot H^+$ $[M+H]^+$: 206.1176, found: 206.1175.

N,4-Dimethoxy-2-(prop-1-en-2-yl)benzamide (1h) [1]

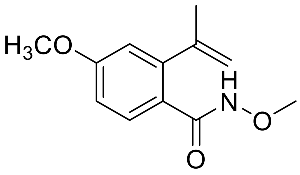

Colorless solid; m.p. 78-82 °C; yield: 80% (EA/PE = 1/3); ^{1}H NMR (400 MHz, $CDCl_3$) δ: 8.85 (s, 1H), 7.57 (d, J = 8.4 Hz, 1H), 6.81 (dd, J = 8.6, 1.8 Hz, 1H), 6.69 (d, J = 2.0 Hz, 1H), 5.23 (s, 1H), 5.08 (s, 1H), 3.81 (s, 6H), 2.06 (s, 3H); ^{13}C NMR (100 MHz, $CDCl_3$) δ: 167.1, 161.3, 146.0, 143.9, 130.9, 123.1, 116.4, 114.2, 112.6, 64.1, 55.3, 24.1.

N-Methoxy-6-(prop-1-en-2-yl)benzo[d][1,3]dioxole-5-carboxamide (1i) [1]

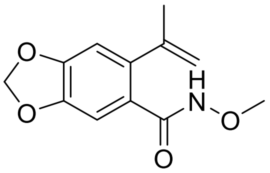

Colorless solid; m.p. 121-123 °C; yield: 79% (EA/PE = 1/2); ^{1}H NMR (400 MHz, $CDCl_3$) δ: 8.67 (s, 1H), 7.11 (s, 1H), 6.66 (s, 1H), 5.99 (s, 2H), 5.23 (s, 1H), 5.07 (s, 1H), 3.83 (s, 3H), 2.06 (s, 3H); ^{13}C NMR (100 MHz, $CDCl_3$) δ: 166.8, 149.5, 146.9, 145.6, 137.1, 124.4, 116.6, 108.9, 108.8, 101.7, 64.1, 24.4.

4-(Dimethylamino)-*N*-methoxy-2-(prop-1-en-2-yl)benzamide (1j)

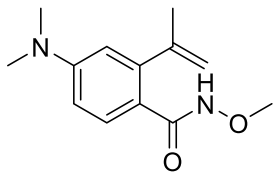

Colorless oil; yield 60% (EA/PE = 1/1); ^{1}H NMR (400 MHz, $CDCl_3$) *δ*: 8.84 (s, 1H), 7.65 (d, *J* = 8.7 Hz, 1H), 6.61 (dd, *J* = 8.8, 2.6 Hz, 1H), 6.40 (d, *J* = 2.7 Hz, 1H), 5.27-5.20 (m, 1H), 5.12 (s, 1H), 3.83 (s, 3H), 3.00 (s, 6H), 2.08 (s, 3H). ^{13}C NMR (100 MHz, $CDCl_3$) δ 167.6, 151.9, 148.0, 143.5, 130.9, 117.2, 115.8, 111.3, 110. 5, 64.2, 40.0, 24.5.

***N*-Methoxy-2-(prop-1-en-2-yl)-5-(trifluoromethyl)benzamide (1k)** [1]

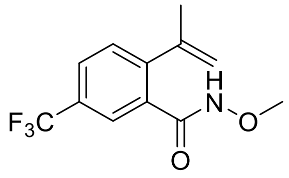

Colorless solid; m.p. 80-84 °C; yield: 59% (EA/PE = 1/7); ^{1}H NMR (400 MHz, $CDCl_3$) *δ*: 8.80 (s, 1H), 7.86 (s, 1H), 7.66 (dd, *J* = 8.0, 1.2 Hz, 1H), 7.37 (d, *J* = 8.0 Hz, 1H), 5.32 (s, 1H), 5.13 (s, 1H), 3.86 (s, 3H), 2.11 (s, 3H); ^{13}C NMR (100 MHz, $CDCl_3$) *δ*: 165.9, 145.7, 143.8, 131.8, 129.5 (q, *J* = 32.8 Hz), 129.2, 127.2, 125.5, 123.5 (q, *J* = 270.6 Hz), 117.4, 63.9, 23.6; ^{19}F NMR (376 MHz, $CDCl_3$) *δ*: -62.70; IR (KBr); 3215, 2983, 1565, 1509, 1340, 1294, 1167, 1109, 940, 910, 850, 780 cm^{-1}; HRMS (ESI): calcd for $C_{12}H_{12}F_3NO_2{\cdot}H^+$ $[M+H]^+$: 260.0893, found: 260.0892.

5-Fluoro-*N*-methoxy-2-(prop-1-en-2-yl)benzamide (1l) [1]

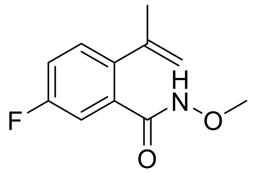

Colorless solid; m.p. 64-66 °C; yield: 13% (EA/PE = 1/2); ^{1}H NMR (400 MHz, $CDCl_3$) *δ*: 8.86 (s, 1H), 7.29 (d, *J* = 8.0 Hz, 1H), 7.22 (dd, *J* = 8.4, 5.6 Hz, 1H), 7.10 (dd, *J* = 8.4, 2.8 Hz, 1H), 5.26 (s, 1H), 5.08 (s, 1H), 3.84 (s, 3H), 2.08 (s, 3H); ^{13}C NMR (100 MHz, $CDCl_3$) *δ*: 165.9, 161.6 (d, *J* = 246.7 Hz), 144.7, 138.0 (d, *J* = 3.5 Hz), 132.7 (d, *J* = 2.4 Hz), 130.6 (d, *J* = 7.5 Hz), 117.8 (d, *J* = 19.6 Hz), 117.0, 115.8 (d, *J* = 22.4 Hz), 64.2, 24.2; ^{19}F NMR (376 MHz, $CDCl_3$) *δ*: -118.48.

4-Fluoro-*N*-methoxy-2-(prop-1-en-2-yl)benzamide (1m) [1]

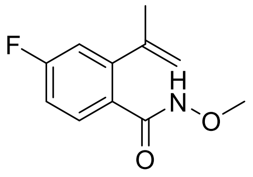

Colorless solid; m.p. 113-115 °C; yield: 29% (EA/PE = 1/3); ^{1}H NMR (400 MHz, $CDCl_3$) *δ*: 9.10 (s, 1H), 7.49 (s, 1H), 7.04-6.80 (m, 2H), 5.21 (s, 1H), 5.05 (s, 1H), 3.77 (s, 3H), 2.04 (s, 3H); ^{13}C NMR (100 MHz, $CDCl_3$) *δ*: 166.4, 163.6 (d, *J* = 250.1 Hz), 144.8 (d, *J* = 8.1 Hz), 144.2, 131.0 (d, *J* = 8.6 Hz), 127.2, 117., 115.5 (d, *J* = 22.1 Hz), 114.3 (d, *J* = 21.3 Hz), 64.0, 23.7; ^{19}F NMR (376 MHz, $CDCl_3$) *δ*: -108.89.

4-Chloro-*N*-methoxy-2-(prop-1-en-2-yl)benzamide (1n) [1]

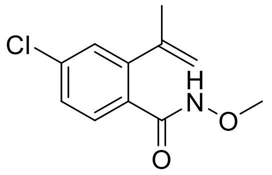

Colorless solid; m.p. 107-109 °C; yield: 54% (EA/PE = 1/4); ^{1}H NMR (400 MHz, $CDCl_3$) *δ*: 8.70 (s, 1H), 7.56 (d, *J* = 7.6 Hz, 1H), 7.32 (d, *J* = 8.0 Hz, 1H), 7.25 (d, *J* = 11.2 Hz, 1H), 5.29 (s, 1H), 5.12 (s, 1H), 3.85 (s, 3H), 2.10 (s, 3H); ^{13}C NMR (100 MHz, $CDCl_3$) *δ*: 166.3, 144.0, 143.8, 136.5, 130.0, 129.4, 128.6, 127.4, 117.1, 64.0, 23.7.

***N*-Methoxy-3-(prop-1-en-2-yl)-2-naphthamide (1o)**

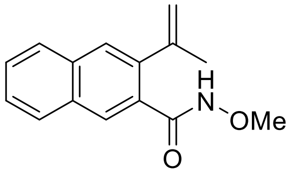

Colorless solid; m.p. 122-124 °C; yield: 46% (EA/PE = 1/4); ^{1}H NMR (400 MHz, $CDCl_3$) *δ*: 8.90 (s, 1H), 8.13 (s, 1H), 7.82 (dd, *J* = 12.8, 8.1 Hz, 2H), 7.69 (s, 1H), 7.52 (dtd, *J* = 14.7, 7.0, 1.2 Hz, 2H), 5.30 (s, 1H), 5.20 (s, 1H), 3.89 (s, 3H), 2.16 (s, 4H); ^{13}C NMR (100 MHz, $CDCl_3$) *δ*: 167.36, 145.95, 138.81, 134.15, 131.82, 129.64, 128.38, 127.92, 127.68, 126.77, 116.61, 64.39, 24.33. HRMS (ESI): calcd for $C_{15}H_{15}NO_2{\cdot}Na^+$ $[M+Na]^+$: 264.0995, found: 264.1000.

N-(Benzyloxy)-2-(prop-1-en-2-yl)benzamide (1p) [2]

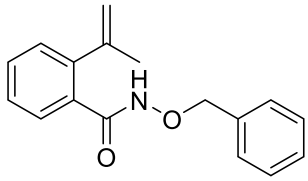

Colorless solid; m.p. 84-86 °C; yield: 65% (EA/PE = 1/4); ^{1}H NMR (400 MHz, $CDCl_3$) *δ*: 8.72 (s, 1H), 7.53 (d, *J* = 7.4 Hz, 1H), 7.46-7.24 (m, 9H), 7.19 (d, *J* = 7.5 Hz, 1H), 5.15-5.06 (m, 1H), 5.00 (d, *J* = 10.0 Hz, 3H), 2.01 (s, 3H); ^{13}C NMR (100 MHz, $CDCl_3$) δ 167.28, 145.47, 142.14, 137.34, 135.46, 130.76, 129.05, 128.73, 128.63, 128.49, 128.39, 128.02, 127.43, 116.42, 78.09, 77.95, 77.45, 77.13, 76.81, 24.25. RMS (ESI): calcd for $C_{17}H_{17}NO_2{\cdot}Na^+$ $[M+Na]^+$: 290.1151, found: 290.1155.

N-Phenyl-2-(prop-1-en-2-yl)benzamide (1q) [4]

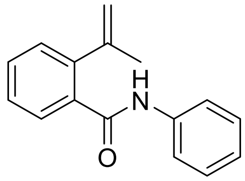

Colorless solid; m.p. 104-105 °C; yield: 74% (EA/PE = 1/4); ^{1}H NMR (400 MHz, $CDCl_3$) *δ*: 7.99 (s, 1H), 7.78 (d, *J* = 7.5 Hz, 1H), 7.58 (d, *J* = 7.8 Hz, 2H), 7.45 (td, *J* = 7.5, 1.4 Hz, 1H), 7.42 – 7.31 (m, 4H), 7.30 – 7.26 (m, 1H), 7.14 (t, *J* = 7.4 Hz, 1H), 5.33 (s, 1H), 5.21 (s, 1H), 2.10 (s, 3H); ^{13}C NMR (100 MHz, $CDCl_3$) *δ*: 167.0, 146.8, 141.8, 138.1, 133.9, 130.9, 129.1, 129.1, 129.0, 127.9, 124.5, 119.7, 116.3, 24.3. HRMS (ESI): calcd for $C_{16}H_{15}NO{\cdot}H^+$ $[M+H]^+$: 238.1226, found: 238.1229.

N-Octyl-2-(prop-1-en-2-yl)benzamide (1r)

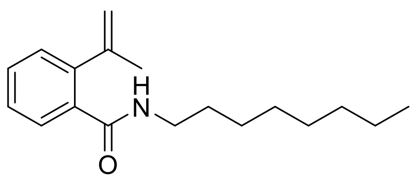

Colorless solid; m.p. 107-108 °C; yield: 69% (EA/PE = 1/2); ^{1}H NMR (400 MHz, $CDCl_3$) *δ*: 7.65 (d, J = 7.4 Hz, 1H), 7.35 (dt, J = 22.4, 7.4 Hz, 2H), 7.21 (d, J = 7.5 Hz, 1H), 6.15 (s, 1H), 5.22 (s, 1H), 5.08 (s, 1H), 3.39 (dt, J = 7.1, 3.6 Hz, 2H), 2.07 (s, 3H), 1.59 – 1.49 (m, 2H), 1.30 (d, J

= 16.4 Hz, 10H), 0.88 (dd, J = 6.7, 5.3 Hz, 3H); ^{13}C NMR (100 MHz, $CDCl_3$) *δ*: 169.0, 146.7, 141.6, 130.2, 128.8, 128.6, 127.5, 115.76, 40.1, 31.8, 29.3, 27.1, 24.3, 22.6, 14.1. HRMS (ESI): calcd for $C_{18}H_{27}NO·H^+$ $[M+H]^+$: 274.2165, found: 274.2168.

***N'*-Phenyl-2-(prop-1-en-2-yl)benzohydrazide (1s)**

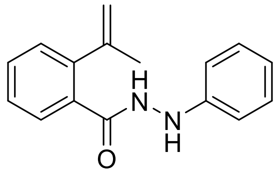

Colorless solid; m.p. 97-98 °C; yield: 27% (EA/PE = 1/2); ^{1}H NMR (400 MHz, $CDCl_3$) *δ:* 7.88 (s, 1H), 7.67 (d, J = 7.5 Hz, 1H), 7.45 (t, J = 7.3 Hz, 1H), 7.36 (t, J = 7.4 Hz, 1H), 7.31 – 7.18 (m, 1H), 6.92 (t, J = 9.4 Hz, 1H), 5.28 (s, 1H), 5.12 (s, 1H), 2.12 (s, 1H); ^{13}C NMR (100 MHz, CDCl3) *δ:* 169.2, 148.0, 145.9, 142.2, 131.7, 131.0, 129.2, 129.0, 128.8, 127.6, 121.5, 116.5, 113.9, 24.7.

2-Methoxy-4-methylisoquinolin-1(2*H*)-one (2a) [5]

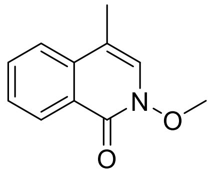

Colorless solid; m.p. 67-69 °C; yield: 88% (EA/PE = 1/3); ^{1}H NMR (400 MHz, $CDCl_3$) *δ*: 8.50 (d, *J* = 8.0 Hz, 1H), 7.70 (d, *J* = 8.0 Hz, 1H), 7.60 (d, *J* = 8.0 Hz, 1H), 7.52 (d, *J* = 8.0 Hz, 1H), 7.16 (s, 1H), 4.08 (s, 3H), 2.29 (s, 3H); ^{13}C NMR (100 MHz, $CDCl_3$) *δ*: 157.7, 136.4, 132.1, 128.0, 127.3, 126.9, 126.8, 123.3, 112.1, 64.2, 15.2.

4-Ethyl-2-methoxyisoquinolin-1(2*H*)-one (2b) [6]

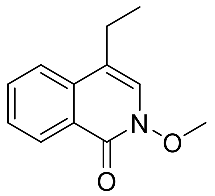

Colorless solid; m.p. 86-89 °C; yield: 94% (EA/PE = 1/3); ^{1}H NMR (400 MHz, $CDCl_3$) *δ*: 8.51 (d, *J* = 8.0 Hz, 1H), 7.74-7.62 (m, 2H), 7.51 (t, *J* = 7.6 Hz, 1H), 7.14 (s, 1H), 4.09 (s, 3H), 2.74 (q,

J = 7.2 Hz, 2H), 1.31 (t, J = 7.4 Hz, 3H); ^{13}C NMR (100 MHz, $CDCl_3$) δ: 157.6, 135.8, 132.0, 128.2, 127.5, 126.7, 126.0, 122.9, 118.0, 64.2, 22.1, 13.4.

4-Isopropyl-2-methoxyisoquinolin-1(2*H*)-one (2c)

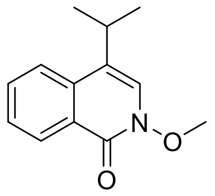

Colorless oil; yield: 51% (EA/PE = 1/3); ^{1}H NMR (400 MHz, $CDCl_3$) δ: 8.53 (ddd, J = 8.1, 1.4, 0.5 Hz, 1H), 7.85-7.61 (m, 2H), 7.52 (ddd, J = 8.1, 6.9, 1.3 Hz, 1H), 7.16 (d, J = 0.8 Hz, 1H), 4.11 (s, 3H), 3.29 (dt, J = 13.4, 6.8 Hz, 1H), 1.33 (d, J = 6.8 Hz, 6H); ^{13}C NMR (100 MHz, $CDCl_3$) δ: 157.5, 135.5, 132.1, 128.4, 127.6, 126.7, 124.9, 122.8, 122.7, 64.2, 26.6, 22.6.

(Z)-4-Isopropyl-1H-isochromen-1-one O-methyl oxime (2c')

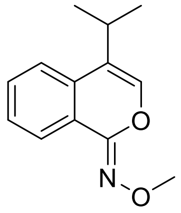

Colorless oil; yield: 31% (EA/PE = 1/4); ^{1}H NMR (400 MHz, $CDCl_3$) δ: 7.90 (d, J = 7.9 Hz, 1H), 7.40 – 7.31 (m, 1H), 7.22 (t, J = 7.6 Hz, 1H), 7.09 (d, J = 7.7 Hz, 1H), 5.82 (s, 1H), 3.96 (s, 3H), 2.69 (p, J = 6.9 Hz, 1H), 1.25 (d, J = 6.9 Hz, 6H); ^{13}C NMR (100 MHz, $CDCl_3$) δ: 153.8, 146.8, 131.6, 130.9, 127.3, 125.9, 124.8, 123.9, 98.9, 62.7, 31.8, 20.2.

4-Cyclopropyl-2-methoxyisoquinolin-1(2*H*)-one (2d)

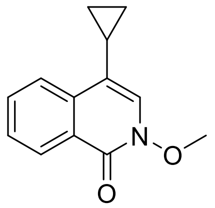

Colorless oil; yield: 54% (EA/PE = 1/3); ^{1}H NMR (400 MHz, $CDCl_3$) δ: 8.49 (d, J = 8.1 Hz, 1H), 8.05 (d, J = 8.1 Hz, 1H), 7.78-7.67 (m, 1H), 7.53 (t, J = 7.6 Hz, 1H), 7.16 (d, J = 1.0 Hz, 1H), 4.08 (s, 3H), 1.94-1.86 (m, 1H), 1.01-0.94 (m, 2H), 0.60 (q, J = 5.2 Hz, 2H); ^{13}C NMR (100 MHz, $CDCl_3$) δ: 157.8, 137.1, 132.1, 128.0, 127.3, 127.0, 126.9, 123.7, 118.0, 64.3, 10.1, 5.3.

2-Methoxy-4-phenylisoquinolin-1(2*H*)-one (2e) [7]

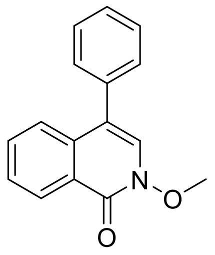

Colorless solid; m.p. 68-70 °C; yield: 68% (EA/PE = 1/2); ^{1}H NMR (400 MHz, $CDCl_3$) *δ*: 8.49 (d, *J* = 7.6 Hz, 1H), 7.62-7.30 (m, 8H), 7.25 (s, 1H), 4.07 (s, 3H).; ^{13}C NMR (100 MHz, $CDCl_3$) *δ*: 157.6, 135.5, 135.4, 132.2, 129.9, 128.7, 128.0, 128.0, 128.0, 127.2, 127.0, 125.0, 119.4, 64.4.

2-Methoxyisoquinolin-1(2*H*)-one (2f) [6]

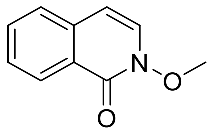

Colorless oil; yield: 87% (EA/PE = 1/3); ^{1}H NMR (400 MHz, $CDCl_3$) *δ*: 8.47 (d, *J* = 8.0 Hz, 1H), 7.66 (t, *J* = 7.6 Hz, 1H), 7.57-7.47 (m, 2H), 7.33 (d, *J* = 7.6 Hz, 1H), 6.47 (d, *J* = 7.6 Hz, 1H), 4.11 (s, 3H); ^{13}C NMR (100 MHz, $CDCl_3$) *δ*: 158.2, 136.1, 132.3, 129.4, 127.7, 127.5, 126.9, 126.2, 105.6, 64.4.

2-Methoxy-4,7-dimethylisoquinolin-1(2*H*)-one (2g)

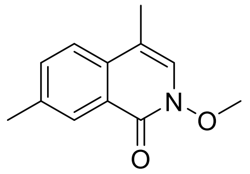

Colorless solid; m.p. 68-70 °C; yield: 95% (EA/PE = 1/4); ^{1}H NMR (400 MHz, $CDCl_3$) *δ*: 8.31 (s, 1H), 7.26 (s, 1H), 7.11 (s, 1H), 4.08 (s, 3H), 2.50 (s, 3H), 2.29 (s, 3H); ^{13}C NMR (100 MHz, $CDCl_3$) *δ*: 157.7, 137.0, 134.2, 133.6, 127.7, 127.2, 126.0, 123.3, 112.2, 64.2, 21.3, 15.2; IR (KBr); 3722, 3657, 3292, 3174, 3072, 2937, 1656, 1612, 1502, 1337, 1262, 1098, 1027, 852, 811 cm^{-1}; HRMS (ESI): calcd for $C_{12}H_{13}NO_2{\cdot}H^+$ $[M+H]^+$: 204.1019, found: 204.1019.

2,6-Dimethoxy-4-methylisoquinolin-1(2*H*)-one (2h)

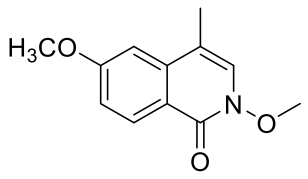

Colorless solid; m.p. 92-94 °C; yield: 68% (EA/PE = 1/2); ^{1}H NMR (400 MHz, $CDCl_3$) δ: 8.38 (d, J = 8.8 Hz, 1H), 7.15-6.99 (m, 2H), 6.89 (s, 1H), 4.05 (s, 3H), 3.91 (s, 3H), 2.23 (s, 3H); ^{13}C NMR (100 MHz, $CDCl_3$) δ: 162.6, 157.5, 138.5, 130.0, 127.5, 120.8, 115.4, 111.5, 105.2, 64.2, 55.4, 15.3; IR (KBr); 3657, 3006, 1663, 1607, 1486, 1260, 1196, 924, 854 cm^{-1}; HRMS (ESI): calcd for $C_{12}H_{13}NO_3 \cdot H^+$ $[M+H]^+$: 220.0968, found: 220.0968.

6-Methoxy-8-methyl-[1,3]dioxolo[4,5-*g*]isoquinolin-5(6*H*)-one (2i)

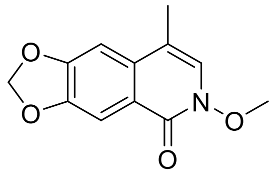

Colorless solid; m.p. 138-139 °C; yield: 76% (EA/PE = 1/2); ^{1}H NMR (400 MHz, $CDCl_3$) δ: 7.81 (s, 1H), 7.07 (s, 1H), 6.92 (s, 1H), 6.08 (s, 2H), 4.06 (s, 3H), 2.21 (s, 3H); ^{13}C NMR (100 MHz, $CDCl_3$) δ: 157.0, 151.9, 147.6, 133.8, 125.7, 122.8, 111.6, 105.9, 101.9, 101.5, 64.2, 15.6; IR (KBr); 3656, 3060, 1659, 1617, 1582, 1487, 1248, 1036, 933, 873 cm^{-1}; HRMS (ESI): calcd for $C_{12}H_{11}NO_4 \cdot H^+$ $[M+H]^+$: 234.0761, found: 234.0760.

6-(Dimethylamino)-2-methoxy-4-methylisoquinolin-1(*2H*)-one (2j)

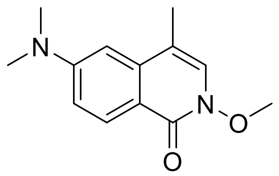

Colorless oil; yield: 85% (EA/PE = 1/1); ^{1}H NMR (400 MHz, $CDCl_3$) δ: 8.32 (d, J = 9.1 Hz, 1H), 7.08 (d, J = 1.0 Hz, 1H), 6.93 (dd, J = 9.1, 2.5 Hz, 1H), 6.54 (d, J = 2.5 Hz, 1H), 4.06 (s, 3H), 3.10 (s, 6H), 2.24 (d, J = 1.0 Hz, 3H); ^{13}C NMR (100 MHz, $CDCl_3$) δ: 158.0, 152.6, 138.1, 129.4, 127.1, 112.7, 111.5, 110.0, 102.5, 64.2, 40.2, 15.4.

2-Methoxy-4-methyl-7-(trifluoromethyl)isoquinolin-1(2*H*)-one (2k)

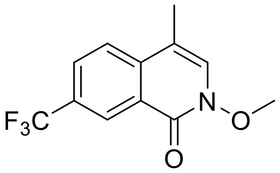

Colorless solid; m.p. 230-232 °C; yield: 84% (EA/PE = 1/3); ^{1}H NMR (400 MHz, $CDCl_3$) *δ*: 8.77 (s, 1H), 7.88 (d, *J* = 8.4 Hz, 1H), 7.72 (d, *J* = 8.8 Hz, 1H), 7.27 (s, 1H), 4.09 (s, 3H), 2.32 (s, 3H); ^{13}C NMR (100 MHz, $CDCl_3$) *δ*: 157.1, 138.8, 129.2, 128.7 (q, *J* = 33.2 Hz), 128.2 (q, *J* = 3.2 Hz), 127.2, 125.6 (q, *J* = 3.7 Hz), 124.3, 123.9 (q, *J* = 270.7 Hz), 111.5, 64.4, 15.1; ^{19}F NMR (376 MHz, $CDCl_3$) *δ*: -62.24; IR (KBr); 3655, 2991, 1666, 1516, 1330, 1260, 1093, 804, 622 cm^{-1}; HRMS (ESI): calcd for $C_{12}H_{10}F_3NO_2{\cdot}H^+$ $[M+H]^+$: 258.0736, found: 258.0738.

7-Fluoro-2-methoxy-4-methylisoquinolin-1(2*H*)-one (2l)

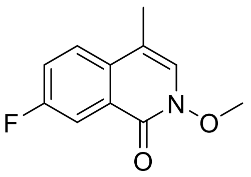

Colorless solid; m.p. 121-123 °C; yield: 84% (EA/PE = 1/3); ^{1}H NMR (400 MHz, $CDCl_3$) *δ*: 8.14 (d, *J* = 8.8 Hz, 1H), 7.67-7.54 (m, 1H), 7.42 (d, *J* = 8.0 Hz, 1H), 7.13 (s, 1H), 4.08 (s, 3H), 2.29 (s, 3H); ^{13}C NMR (100 MHz, $CDCl_3$) *δ*: 161.4 (d, *J* = 246.9 Hz), 156.9 (d, *J* = 3.2 Hz), 133.1 (d, *J* = 1.7 Hz), 129.0 (d, *J* = 8.1 Hz), 126.3 (d, *J* = 1.7 Hz), 125.8 (d, *J* = 7.9 Hz), 120.8 (d, *J* = 23.4 Hz), 113.3 (d, *J* = 22.9 Hz), 111.7, 64.2, 15.3; ^{19}F NMR (376 MHz, $CDCl_3$) *δ*: -108.61; IR (KBr); 3070, 2936, 1656, 1259, 1182, 1020, 952, 871, 816, 778, 705 cm^{-1}; HRMS (ESI): calcd for $C_{11}H_{10}FNO_2{\cdot}H^+$ $[M+H]^+$: 208.0768, found: 208.0771.

6-Fluoro-2-methoxy-4-methylisoquinolin-1(2*H*)-one (2m)

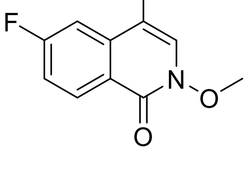

Colorless solid; m.p. 99-101 °C; yield: 81% (EA/PE = 1/2); ^{1}H NMR (400 MHz, $CDCl_3$) *δ*: 8.55-8.42 (m, 1H), 7.30-7.14 (m, 3H), 4.08 (s, 3H), 2.25 (s, 3H); ^{13}C NMR (100 MHz, $CDCl_3$) *δ*: 165.2

(d, J = 252.6 Hz), 157.1, 139.0 (d, J = 10.0 Hz), 131.2 (d, J = 9.9 Hz), 128.3, 123.8, 115.3 (d, J = 23.5 Hz), 111.4 (d, J = 3.3 Hz), 108.8 (d, J = 22.6 Hz), 64.3, 15.2; ^{19}F NMR (376 MHz, $CDCl_3$) δ: -106.58; IR (KBr); 2922, 1662, 1621, 1476, 1452, 1326, 1017, 988, 948, 857, 821, 770, 693 cm^{-1}; HRMS (ESI): calcd for $C_{11}H_{10}FNO_2{\cdot}H^+$ [M+H]+: 208.0768, found: 208.0772.

6-Chloro-2-methoxy-4-methylisoquinolin-1(2*H*)-one (2n)

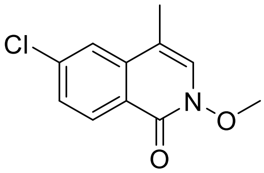

Colorless solid; m.p. 107-109 °C; yield: 93% (EA/PE = 1/2); ^{1}H NMR (400 MHz, $CDCl_3$) δ: 8.40 (d, J = 8.4 Hz, 1H), 7.55 (s, 1H), 7.44 (d, J = 8.4 Hz, 1H), 7.18 (s, 1H), 4.07 (s, 3H), 2.25 (s, 3H); ^{13}C NMR (100 MHz, $CDCl_3$) δ: 157.2, 138.8, 137.8, 129.8, 128.3, 127.2, 125.5, 122.9, 111.1, 64.3, 15.1; IR (KBr); 3066, 2939, 1662, 1614, 1470, 1429, 1323, 1094, 1017, 920, 822, 775, 687, 616 cm^{-1}; HRMS (ESI): calcd for $C_{11}H_{10}ClNO_2{\cdot}H^+$ [M+H]+: 224.0473, found: 224.0469.

2-Methoxy-4-methylbenzo[*g*]isoquinolin-1(*2H*)-one (2o)

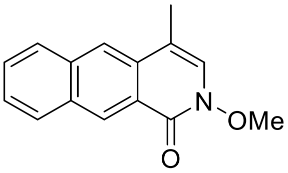

Colorless oil; yield: 40% (EA/PE = 1/3); ^{1}H NMR (400 MHz, $CDCl_3$) δ: 9.10 (s, 1H), 8.06 (d, J = 8.2 Hz, 1H), 8.04 (s, 1H), 7.98 (d, J = 8.3 Hz, 1H), 7.61 (dd, J = 11.1, 4.0 Hz, 1H), 7.55 (dd, J = 11.0, 3.9 Hz, 1H), 7.11 (s, 1H), 4.11 (s, 3H), 2.40 (s, 3H); ^{13}C NMR (100 MHz, $CDCl_3$) δ: 158.4, 135.0, 132.7, 131.5, 129.4, 129.3, 128.3, 127.9, 126.4, 126.1, 125.4, 122.2, 112.3, 64.1, 15.5. HRMS (ESI): calcd for $C_{15}H_{13}NO_2{\cdot}H^+$ [M+H]+: 240.1019, found: 240.1025.

2-(Benzyloxy)-4-methylisoquinolin-1(*2H*)-one (2p)

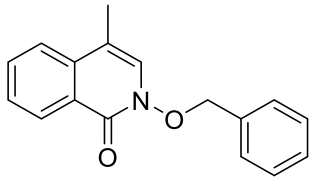

Colorless oil; yield: 69% (EA/PE = 1/10); ^{1}H NMR (400 MHz, $CDCl_3$) *δ:* 8.54 (dd, *J* = 8.1, 0.9 Hz, 1H), 7.71 (ddd, *J* = 8.3, 7.1, 1.4 Hz, 1H), 7.59 (d, *J* = 8.0 Hz, 1H), 7.56 – 7.51 (m, 1H), 7.49 (dt, *J* = 4.8, 3.0 Hz, 2H), 7.41 – 7.35 (m, 3H), 6.94 (d, *J* = 1.1 Hz, 1H), 5.29 (s, 2H), 2.20 (d, *J* = 1.1 Hz, 3H); ^{13}C NMR (100 MHz, $CDCl_3$) *δ:* 158.2, 136.5, 134.2, 132.2, 129.9, 129.2, 128.7, 128.3, 128.1, 127.3, 126.7, 123.3, 111.4, 78.4, 15.1; HRMS (ESI): calcd for $C_{17}H_{15}NO_2{\cdot}H^+$ [M+H]+: 288.0995, found: 288.1001.

4-Methyl-2-phenylisoquinolin-1(*2H*)-one (2q) [8]

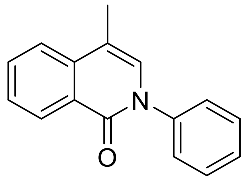

Colorless solid; m.p. 107-108 °C; yield: 41% (EA/PE = 1/2); ^{1}H NMR (400 MHz, $CDCl_3$) *δ:* 8.37 (d, *J* = 8.0 Hz, 1H), 7.64 (t, *J* = 7.5 Hz, 1H), 7.53 (t, *J* = 7.4 Hz, 2H), 7.49 – 7.40 (m, 3H), 7.35 (d, *J* = 6.4 Hz, 1H), 7.24 (s, 1H), 6.44 (s, 1H), 2.01 (s, 3H); ^{13}C NMR (101 MHz, $CDCl_3$) *δ:* 163.5, 139.5, 139.2, 137.2, 132.6, 129.6, 128.6, 128.5, 128.2, 126.1, 125.2, 122.8, 105.5, 21.7.

4-Methyl-2-(phenylamino)isoquinolin-1(*2H*)-one (2r)

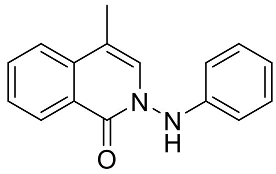

Colorless oil; yield: 40% (EA/PE = 1/5); ^{1}H NMR (400 MHz, $CDCl_3$) *δ:* 8.46 (dd, *J* = 8.0, 0.8 Hz, 1H), 7.75 (ddd, *J* = 8.3, 7.1, 1.4 Hz, 1H), 7.68 (d, *J* = 7.7 Hz, 1H), 7.58 – 7.49 (m, 1H), 7.45 (s, 1H), 7.25 – 7.19 (m, 3H), 6.96 (t, *J* = 7.4 Hz, 1H), 6.78 (dd, *J* = 8.6, 0.9 Hz, 2H), 2.35 (d, *J* = 1.1 Hz, 3H); ^{13}C NMR (101 MHz, $CDCl_3$) *δ:* 161.2, 147.3, 137.3, 132.5, 131.2, 129.4, 128.4, 126.9,

126.2, 123.4, 122.5, 114.6, 112.1, 15.4; HRMS (ESI): calcd for $C_{16}H_{14}N_2O{\cdot}Na^+$ $[M+Na]^+$: 273.0998, found: 273.1002.

2-Methoxy-3-methylisoquinolin-1(2*H*)-one (3a) [9]

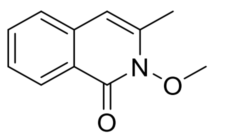

Colorless solid; m.p. 81-87 °C; yield: 84% (EA/PE = 1/4); ^{1}H NMR (400 MHz, $CDCl_3$) δ: 8.36 (d, J = 8.0 Hz, 1H), 7.58 (t, J = 7.6 Hz, 1H), 7.48-7.33 (m, 2H), 6.25 (s, 1H), 4.06 (s, 3H), 2.42 (s, 3H); ^{13}C NMR (100 MHz, $CDCl_3$) δ: 158.8, 139.1, 136.0, 132.2, 127.4, 125.7, 125.2, 104.2, 63.6, 17.1.

3-Ethyl-2-methoxyisoquinolin-1(2*H*)-one (3b) [9]

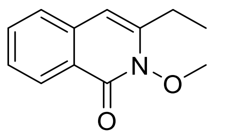

Colorless solid; m.p. 98-100 °C; yield: 74% (EA/PE = 1/5); ^{1}H NMR (400 MHz, $CDCl_3$) δ: 8.39 (d, J = 8.0 Hz, 1H), 7.60 (t, J = 7.4 Hz, 1H), 7.50-7.36 (m, 2H), 6.26 (s, 1H), 4.09 (s, 3H), 2.78 (q, J = 7.3 Hz, 2H), 1.34 (t, J = 7.4 Hz, 3H); ^{13}C NMR (100 MHz, $CDCl_3$) δ: 159.0, 144.4, 136.0, 132.2, 127.5, 125.8, 125.8, 125.5, 102.5, 63.8, 23.6, 12.4.

3-Isopropyl-2-methoxyisoquinolin-1(2*H*)-one (3c) [9]

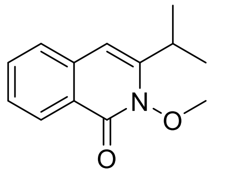

Colorless oil; yield: 72% (EA/PE = 1/3); ^{1}H NMR (400 MHz, $CDCl_3$) δ: 8.43-8.36 (m, 1H), 7.66-7.57 (m, 1H), 7.44 (ddd, J = 12.1, 8.1, 6.9 Hz, 2H), 6.30 (s, 1H), 4.09 (s, 3H), 3.20 (dt, J = 13.6, 6.8 Hz, 1H), 1.34 (d, J = 6.8 Hz, 6H); ^{13}C NMR (100 MHz, $CDCl_3$) δ: 159.0, 148.9, 136.0, 132.2, 127.5, 125.9, 125.7, 125.6, 100.8, 64.0, 28.3, 22.1.

3-Cyclopropyl-2-methoxyisoquinolin-1(*2H*)-one (3d) [10]

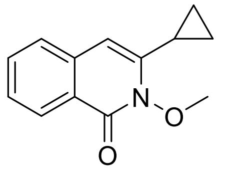

Colorless oil; yield: 52% (EA/PE = 1/3); ^{1}H NMR (400 MHz, $CDCl_3$) δ 8.39 (d, *J* = 8.1 Hz, 1H), 7.59 (t, *J* = 7.0 Hz, 1H), 7.46-7.37 (m, 2H), 6.08 (s, 1H), 4.15 (s, 3H), 2.28-2.16 (m, 1H), 1.11-1.01 (m, 2H), 0.83 (q, *J* = 5.9 Hz, 2H); ^{13}C NMR (100 MHz, $CDCl_3$) δ 159.0, 144.6, 136.0, 132.2, 127.6, 125.9, 125.7, 125.6, 100.6, 63.8, 10.6, 7.0.

2-Methoxy-3,7-dimethylisoquinolin-1(2*H*)-one (3e)

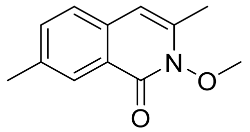

Colorless solid; m.p. 109-111 °C; yield: 69% (EA/PE = 1/5); ^{1}H NMR (400 MHz, $CDCl_3$) *δ*: 8.18 (s, 1H), 7.41 (d, *J* = 8.0 Hz, 1H), 7.31 (d, *J* = 8.1 Hz, 1H), 6.23 (s, 1H), 4.06 (s, 3H), 2.45 (s, 1H), 2.41 (s, 1H); ^{13}C NMR (100 MHz, $CDCl_3$) *δ*: 158.8, 138.0, 135.8, 133.8, 133.7, 127.0, 125.8, 125.2, 104.1, 63.5, 21.3, 17.1; IR (KBr); 2923, 1664, 1612, 1496, 1449, 1380, 1335, 1256, 977, 825, 784, 690, 592, 544 cm^{-1}; HRMS (ESI): calcd for $C_{12}H_{13}NO_2{\cdot}H^+$ $[M+H]^+$: 204.1019, found: 204.1020.

2,6-Dimethoxy-3-methylisoquinolin-1(2*H*)-one (3f)

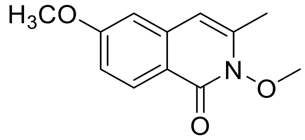

Colorless solid; m.p. 126-128 °C; yield: 65% (EA/PE = 1/4); ^{1}H NMR (400 MHz, $CDCl_3$) *δ*: 8.27 (d, *J* = 8.8 Hz, 1H), 6.98 (d, *J* = 7.6 Hz, 1H), 6.76 (s, 1H), 6.17 (s, 1H), 4.06 (s, 3H), 3.87 (s, 3H), 2.41 (s, 3H); ^{13}C NMR (100 MHz, $CDCl_3$) *δ*: 162.7, 158.6, 139.7, 138.0, 129.6, 119.6, 115.5, 106.0, 103.8, 63.6, 55.34, 17.2; IR (KBr); 3658, 2994, 1665, 1604, 1487, 1257, 1225, 1170, 978, 855, 829, 686 cm^{-1}; HRMS (ESI): calcd for $C_{12}H_{13}NO_3{\cdot}H^+$ $[M+H]^+$: 220.0968, found: 220.0965.

6-Methoxy-7-methyl-[1,3]dioxolo[4,5-*g*]isoquinolin-5(6*H*)-one (3g)

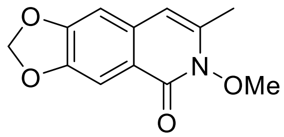

Colorless solid; m.p. 124-126 °C; yield: 63% (EA/PE = 1/2); ^{1}H NMR (400 MHz, $CDCl_3$) *δ*: 7.70 (s, 1H), 6.74 (s, 1H), 6.13 (s, 1H), 6.04 (s, 2H), 4.06 (s, 3H), 2.39 (s, 3H); ^{13}C NMR (100 MHz, $CDCl_3$) *δ*: 158.2, 151.9, 147.1, 137.6, 133.3, 121.2, 105.2, 104.0, 103.0, 101.6, 63.6, 17.0; IR (KBr); 3660, 2995, 1662, 1621, 1412, 1240, 1037, 931, 868 cm^{-1}; HRMS (ESI): calcd for $C_{12}H_{11}NO_4{\cdot}H^+$ $[M+H]^+$: 234.0761, found: 234.0762.

2-Methoxy-3-methyl-7-(trifluoromethyl)isoquinolin-1(2*H*)-one (3h)

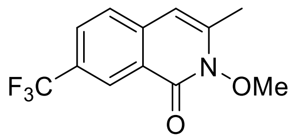

Colorless solid; m.p. 158-160 °C; yield: 44% (EA/PE = 1/3); ^{1}H NMR (400 MHz, $CDCl_3$) *δ*: 8.67 (s, 1H), 7.77 (d, *J* = 8.0 Hz, 1H), 7.52 (d, *J* = 8.4 Hz, 1H), 6.32 (s, 1H), 4.10 (s, 3H), 2.48 (s, 3H); ^{13}C NMR (100 MHz, $CDCl_3$) *δ*: 158.3, 141.9, 138.4, 128.3 (q, *J* = 3.2 Hz), 127.8 (q, *J* = 33.4 Hz), 126.2, 125.5, 125.4 (d, *J* = 4.1 Hz), 123.9 (d, *J* = 285.6 Hz), 103.6, 63.8, 17.4; ^{19}F NMR (376 MHz, $CDCl_3$) *δ*: -62.15.

7-Fluoro-2-methoxy-3-methylisoquinolin-1(2*H*)-one (3i)

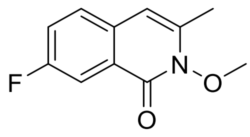

Colorless solid; m.p. 116-118 °C; yield: 90% (EA/PE = 1/3); ^{1}H NMR (400 MHz, $CDCl_3$) *δ*: 8.01 (dd, *J* = 9.2, 2.4 Hz, 1H), 7.41 (dd, *J* = 8.8, 5.2 Hz, 1H), 7.32 (td, *J* = 8.4, 2.7 Hz, 1H), 6.25 (s, 1H), 4.07 (s, 3H), 2.43 (s, 3H); ^{13}C NMR (100 MHz, $CDCl_3$) *δ*: 160.7 (d, *J* = 245.5 Hz), 158.1 (d, *J* = 3.6 Hz), 138.4 (d, *J* = 2.9 Hz), 132.67, 127.67 (d, *J* = 7.8 Hz), 127.3 (d, *J* = 7.7 Hz), 121.2 (d, *J* = 23.7 Hz), 112.5 (d, *J* = 22.7 Hz), 103.6, 63.62 (d, *J* = 3.3 Hz), 17.10 (d, *J* = 1.9 Hz); ^{19}F NMR (376 MHz, $CDCl_3$) *δ*: -113.99; IR (KBr); 2930, 1660, 1620, 1497, 1346, 1256, 1090, 954, 843, 798 cm^{-1}; HRMS (ESI): calcd for $C_{11}H_{10}FNO_2{\cdot}H^+$ $[M+H]^+$: 208.0768, found: 208.0772.

6-Fluoro-2-methoxy-3-methylisoquinolin-1(2*H*)-one (3j)

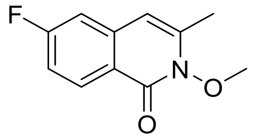

Colorless solid; m.p. 120-122 °C; yield: 53% (EA/PE = 1/5); ^{1}H NMR (400 MHz, $CDCl_3$) *δ*: 8.39 (dd, *J* = 8.8, 6.0 Hz, 1H), 7.11 (td, *J* = 8.8, 2.3 Hz, 1H), 7.04 (dd, *J* = 9.4, 2.2 Hz, 1H), 6.21 (s, 1H), 4.08 (s, 3H), 2.44 (s, 3H); ^{13}C NMR (100 MHz, $CDCl_3$) *δ*: 165.2 (d, *J* = 250.8 Hz), 158.3, 140.7, 138.2 (d, *J* = 10.5 Hz), 130.8 (d, *J* = 10.1 Hz), 122.5, 114.6 (d, *J* = 23.5 Hz), 110.0 (d, *J* = 21.8 Hz), 103.5 (d, *J* = 3.2 Hz), 63.7, 17.3; ^{19}F NMR (376 MHz, $CDCl_3$) *δ*: -109.15.

6-Chloro-2-methoxy-3-methylisoquinolin-1(2*H*)-one (3k)

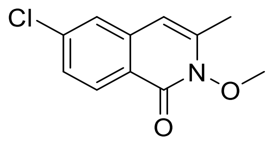

Colorless solid; m.p. 105-107 °C; yield: 76% (EA/PE = 1/8); ^{1}H NMR (400 MHz, $CDCl_3$) *δ*: 8.31 (d, *J* = 8.8 Hz, 1H), 7.41 (d, *J* = 1.6 Hz, 1H), 7.35 (dd, *J* = 8.6, 1.8 Hz, 1H), 6.19 (s, 1H), 4.08 (s, 3H), 2.45 (s, 3H); ^{13}C NMR (100 MHz, $CDCl_3$) *δ*: 158.4, 140.8, 138.7, 137.2, 129.4, 126.4, 124.5, 124.1, 103.2, 63.7, 17.3; IR (KBr); 1659, 1380, 1257, 1078, 977, 908, 825, 680 cm^{-1}; HRMS (ESI): calcd for $C_{11}H_{10}ClNO_2{\cdot}H^+$ $[M+H]^+$: 224.0473, found: 224.0469.

2-Methoxy-3-methylbenzo[*g*]isoquinolin-1(*2H*)-one (3l)

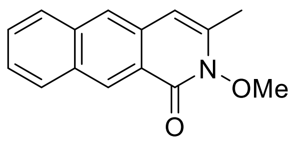

Colorless solid; m.p. 123-125 °C; yield: 54% (EA/PE = 1/5); ^{1}H NMR (400 MHz, $CDCl_3$) *δ:* 9.00 (s, 1H), 8.03 (d, *J* = 8.3 Hz, 1H), 7.89 (d, *J* = 8.0 Hz, 2H), 7.53 (dt, *J* = 14.9, 7.2 Hz, 2H), 6.38 (s, 1H), 4.11 (s, 3H), 2.47 (s, 3H); ^{13}C NMR (100 MHz, $CDCl_3$) *δ:* 159.4, 138.1, 135.4, 132.0, 131.1, 129.4, 128.9, 128.1, 127.5, 125.8, 124.5, 123.3, 104. 63.8, 17.4; HRMS (ESI): calcd for $C_{15}H_{13}NO_2{\cdot}H^+$ $[M+H]^+$: 240.1019, found: 240.1021.

2-(Benzyloxy)-3-methylisoquinolin-1(*2H*)-one (3m)

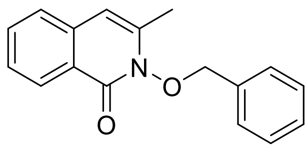

Colorless oil; yield: 64% (EA/PE = 1/10); ^{1}H NMR (400 MHz, $CDCl_3$) *δ:* 8.43 (d, *J* = 7.4 Hz, 1H), 7.66 – 7.58 (m, 1H), 7.54 (dd, *J* = 6.6, 3.0 Hz, 2H), 7.42 (ddd, *J* = 9.0, 8.5, 5.0 Hz, 5H), 6.25 (s, 1H), 5.29 (s, 2H), 2.33 (d, *J* = 0.8 Hz, 3H); ^{13}C NMR (100 MHz, $CDCl_3$) *δ:* 159.2, 139.7, 136.2, 134.2, 132.3, 129.9, 129.2, 128.7, 127.6, 125.9, 125.9, 125.3, 104.1, 77.7, 17.7; HRMS (ESI): calcd for $C_{17}H_{15}NO_2{\cdot}H^+$ $[M+H]^+$: 288.0995, found: 288.0998.

3-Methyl-2-phenylisoquinolin-1(*2H*)-one (3n) [11]

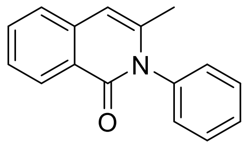

Colorless solid; m.p. 113-114 °C; yield: 51% (EA/PE = 1/5); ^{1}H NMR (400 MHz, $CDCl_3$) *δ:* 8.38 (d, *J* = 8.0 Hz, 1H), 7.68 – 7.59 (m, 1H), 7.53 (dd, *J* = 10.1, 4.7 Hz, 2H), 7.49 – 7.38 (m, 3H), 7.25 (d, *J* = 7.3 Hz, 2H), 6.44 (s, 1H), 2.01 (s, 3H); ^{13}C NMR (100 MHz, $CDCl_3$) *δ:* 163.5, 139.5, 139.2, 137.2, 132.6, 129.6, 128.6, 128.5, 128.2, 126.1, 125.2, 124.9, 105.5, 21.6; HRMS (ESI): calcd for $C_{16}H_{13}NO{\cdot}H^+$ $[M+H]^+$: 236.1070, found: 236.1071.

3-Methyl-2-octylisoquinolin-1(*2H*)-one (3o)

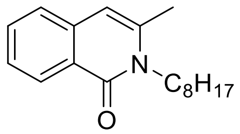

Colorless oil; yield: 32% (EA/PE = 1/5); ^{1}H NMR (400 MHz, $CDCl_3$) *δ:* 8.36 (d, *J* = 8.1 Hz, 1H), 7.57 (t, *J* = 7.5 Hz, 1H), 7.39 (d, *J* = 6.9 Hz, 2H), 6.34 (s, 1H), 4.07 (t, 2H), 1.70 (dd, *J* = 16.7, 9.2 Hz, 2H), 1.35 (dd, *J* = 47.8, 9.4 Hz, 10H), 0.87 (d, *J* = 6.9 Hz, 3H); ^{13}C NMR (100 MHz, $CDCl_3$) *δ:* 163.0, 139.0, 136.7, 132.0, 127.9, 125.8, 124.9, 124.6, 105.9, 44.5, 31.8, 29.3, 29.3, 28.9, 27.1, 22.6, 20.6, 14.1.

V. References

[1] Kou, X.; Li, Y.; Wu, L.; Zhang, X.; Yang, G.; Zhang, W. *Org. Lett.* **2015**, *17*, 5566–5569.

[2] Lei, Z.-Y.; Hu, K.; He, Y.-X.; Geng, S.; Chen, L.-N.; Zou, S.; Pan, L.; Ding, Z.-J.; Huang, F. *Org. Biomol. Chem.* **2022**, *20*, 2397 – 2401

[3] Zhang, Z.-Q.; Liu, F. *Org. Biomol. Chem.*, **2015**, *13*, 6690–6693.

[4] Zhang, W.; Chen, P.; Liu, G. *Angew. Chem. Int. Ed*. **2017**, *56*, 5336–5340.

[5] Li, Y.; Kou, X.; Ye, C.; Zhang, X.; Yang, G.; Zhang, W. *Tetrahedron Lett.* **2017**, *58*, 285–288.

[6] Huang. J.-R.; Bolm, C. *Angew. Chem. Int. Ed.* **2017**, *56*, 15921–15925.

[7] Kumar, V.; Gandeepan, P. *Eur. J. Org. Chem.* **2023**, *26*, e20230091.

[8] Stanoeva, E.; Haimova, M.; Ognyanov, V. *Liebigs Annalen der Chemie*, **1984,** *2*, 389–394.

[9] Xu, Y.; Zheng, G.; Yang, X.; Li, X. *Chem. Commun.*, **2018**, *54*, 670–673.

[10] Liu, R.; Li, M.; Xie, W.; Zhou, H.; Zhang, Y.; Qiu, G. *J. Org. Chem.* **2019**, *84*, 11763–11773.

[11] Wang, L.; Sun, M.; Ding, M.-W. *Eur. J. Org. Chem.* **2017**, *18*, 2568-2578.

VI. Spectra

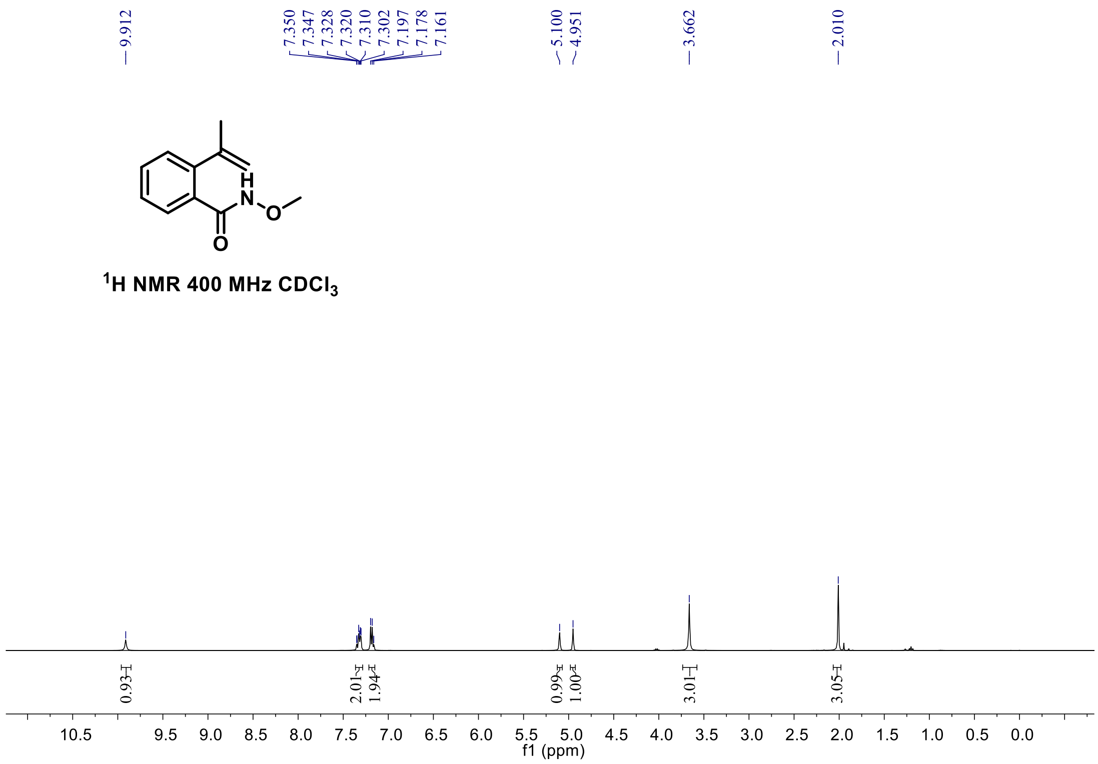

9.912
7.350
7.347
7.328
7.320
7.310
7.302
7.197
7.178
7.161
5.100
4.951
3.662
2.010
H
N
O
O
¹H NMR 400 MHz CDCl₃
0.93
2.01
1.94
0.99
1.00
3.01
3.05
10.5 9.5 9.0 8.5 8.0 7.5 7.0 6.5 6.0 5.5 5.0 4.5 4.0 3.5 3.0 2.5 2.0 1.5 1.0 0.5 0.0
f1 (ppm)

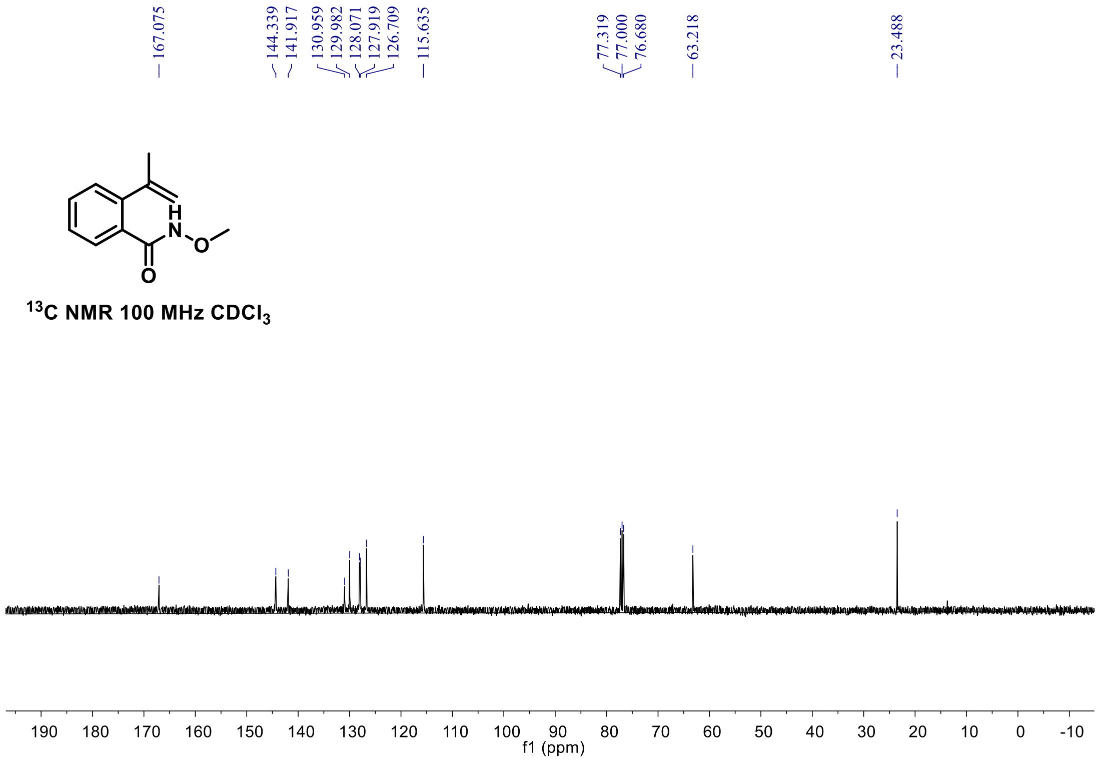

167.075
144.339
141.917
130.959
129.982
128.071
127.919
126.709
115.635
77.319
77.000
76.680
63.218
23.488
H
N
O
O
¹³C NMR 100 MHz CDCl₃
190 180 170 160 150 140 130 120 110 100 90 80 70 60 50 40 30 20 10 0 -10
f1 (ppm)

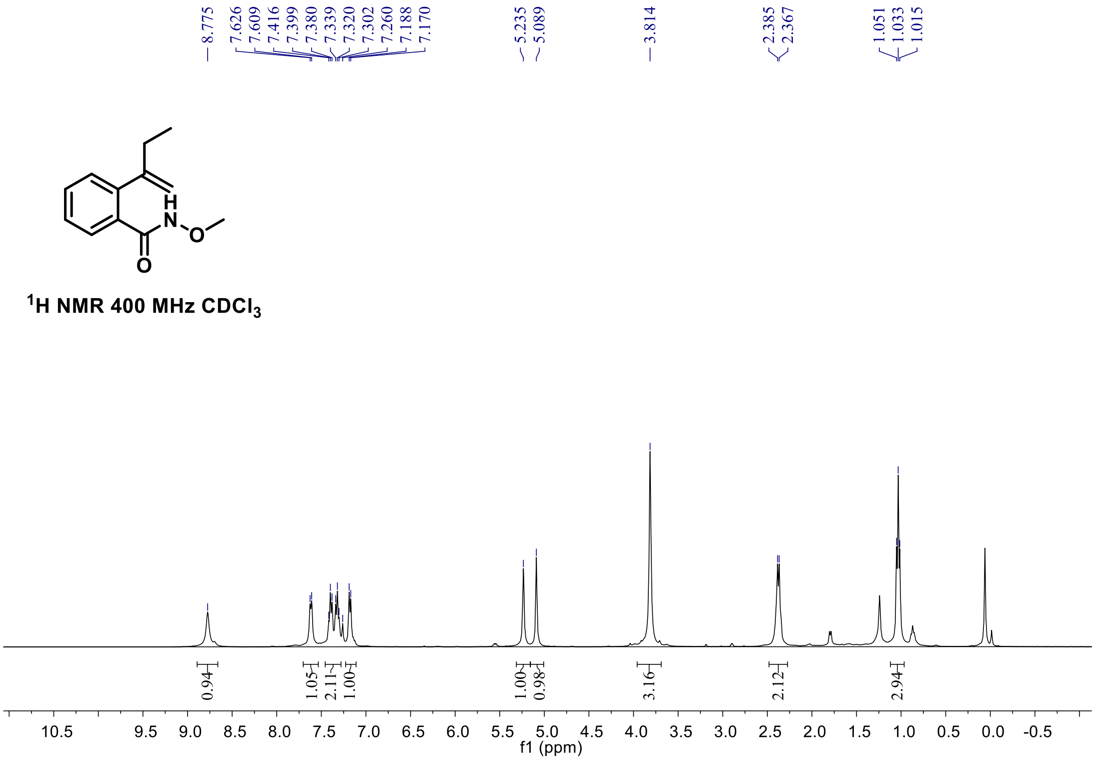
8.775
7.626
7.609
7.416
7.399
7.380
7.339
7.320
7.302
7.260
7.188
7.170
5.235
5.089
3.814
2.385
2.367
1.051
1.033
1.015
H
N
O
O
1H NMR 400 MHz CDCl3
0.94
1.05
2.11
1.00
1.00
0.98
3.16
2.12
2.94
f1 (ppm)

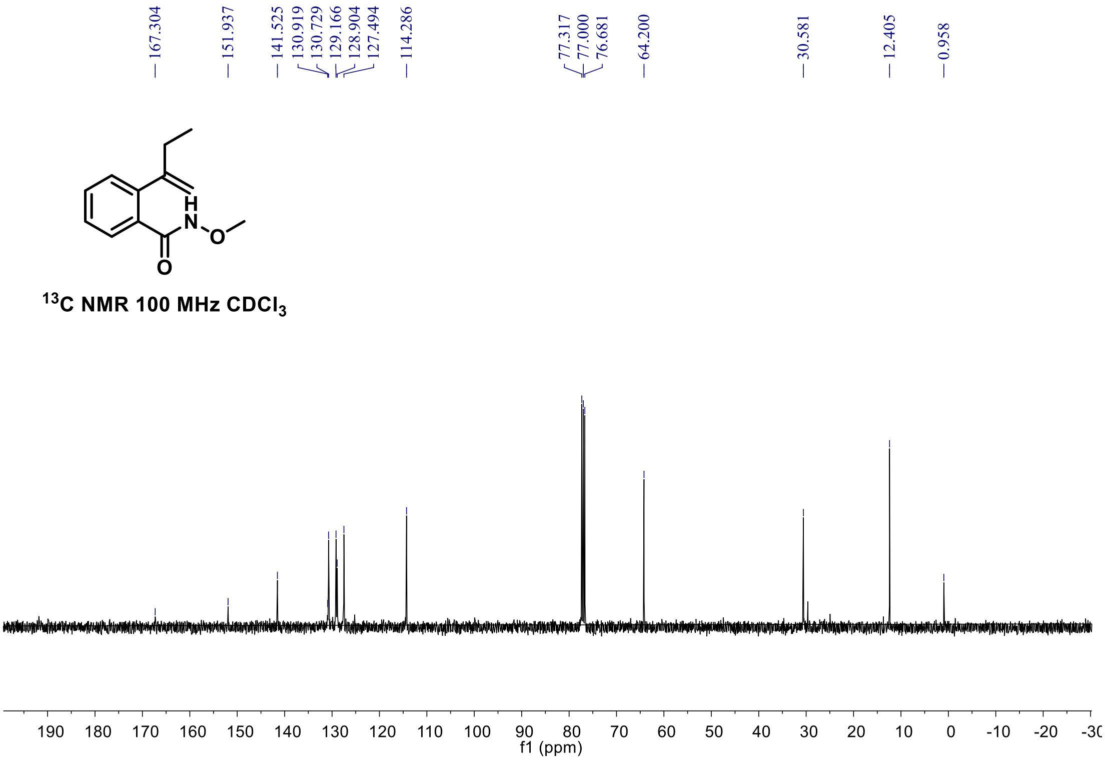
167.304
151.937
141.525
130.919
130.729
129.166
128.904
127.494
114.286
77.317
77.000
76.681
64.200
30.581
12.405
0.958
H
N
O
O
13C NMR 100 MHz CDCl3
f1 (ppm)

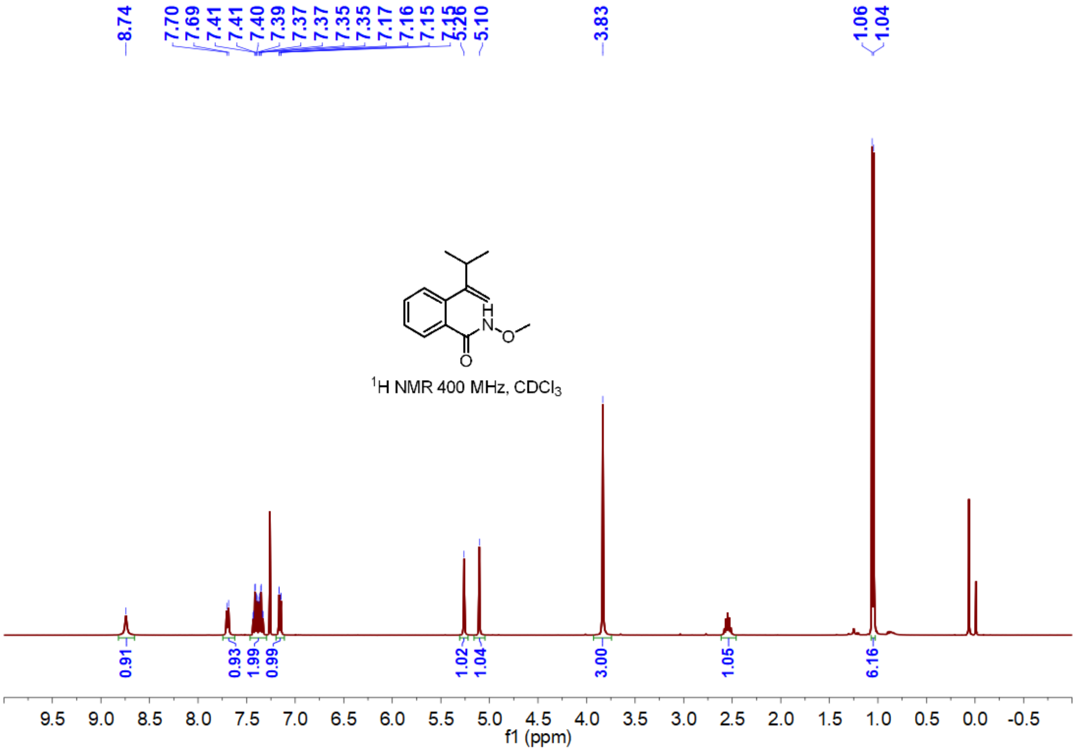

8.74
7.70
7.69
7.41
7.41
7.40
7.39
7.37
7.37
7.35
7.35
7.17
7.16
7.15
7.15
5.26
5.10
3.83
1.06
1.04
H
N
O
O
^{1}H NMR 400 MHz, CDCl$_3$
0.91
0.93
1.99
0.99
1.02
1.04
3.00
1.05
6.16
9.5 9.0 8.5 8.0 7.5 7.0 6.5 6.0 5.5 5.0 4.5 4.0 3.5 3.0 2.5 2.0 1.5 1.0 0.5 0.0 -0.5
f1 (ppm)

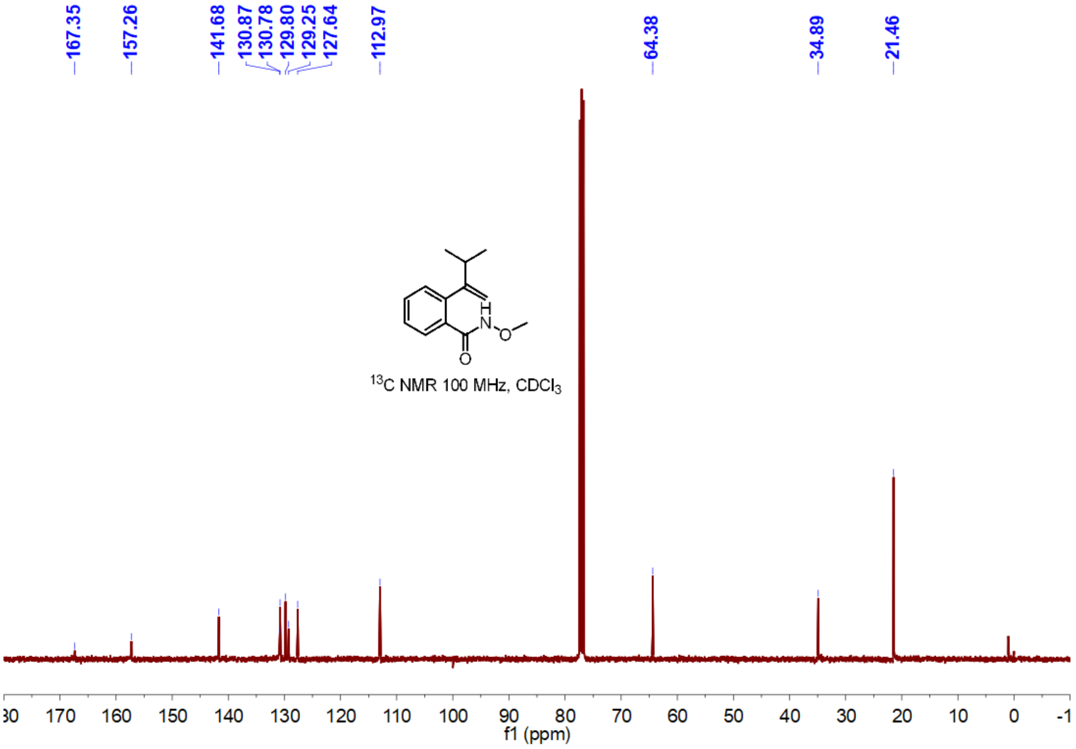

167.35
157.26
141.68
130.87
130.78
129.80
129.25
127.64
112.97
64.38
34.89
21.46
H
N
O
O
^{13}C NMR 100 MHz, CDCl$_3$
170 160 150 140 130 120 110 100 90 80 70 60 50 40 30 20 10 0
f1 (ppm)

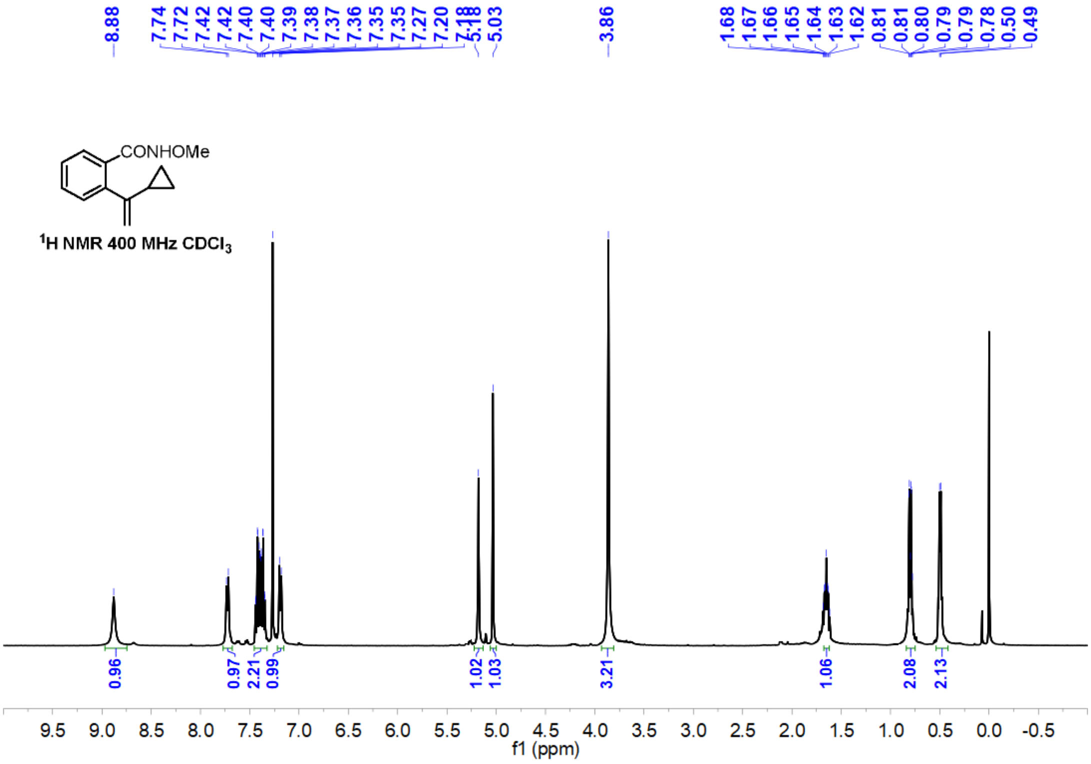
CONHOMe
1H NMR 400 MHz CDCl3
8.88
7.74
7.72
7.42
7.42
7.40
7.40
7.39
7.38
7.37
7.36
7.35
7.35
7.27
7.20
7.18
5.18
5.03
3.86
1.68
1.67
1.66
1.65
1.64
1.63
1.62
0.81
0.81
0.80
0.79
0.79
0.78
0.50
0.49
0.96
0.97
2.21
0.99
1.02
1.03
3.21
1.06
2.08
2.13
f1 (ppm)

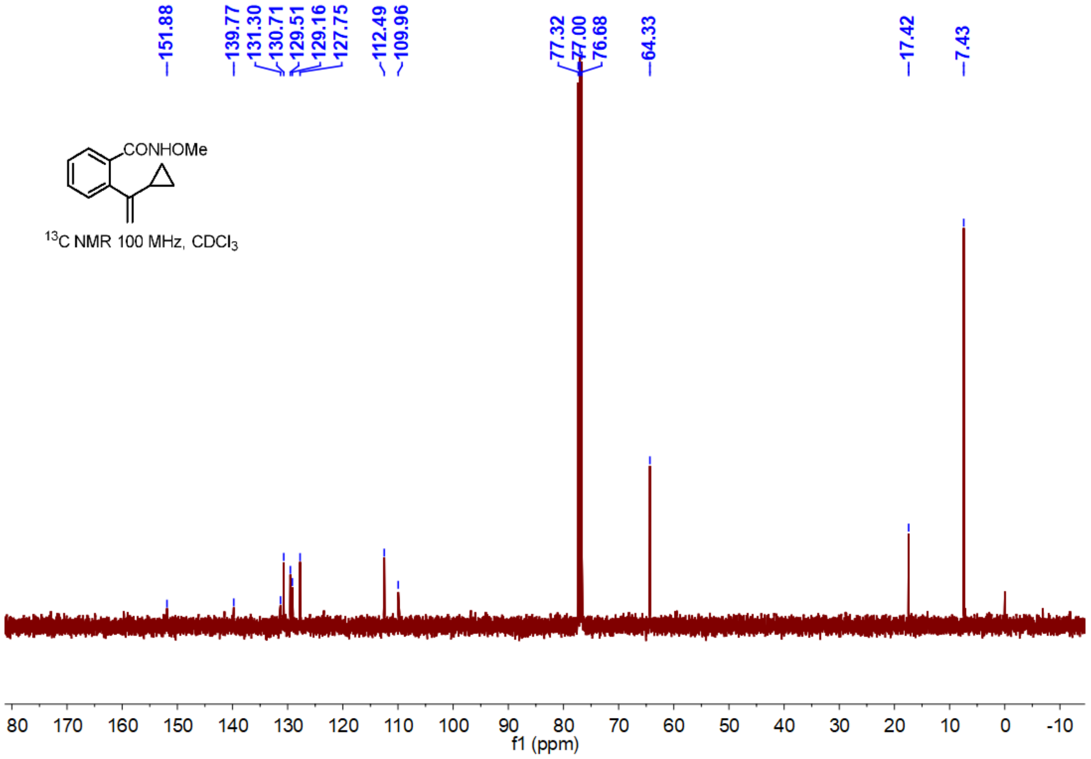
CONHOMe
13C NMR 100 MHz, CDCl3
151.88
139.77
131.30
130.71
129.51
129.16
127.75
112.49
109.96
77.32
77.00
76.68
64.33
17.42
7.43
f1 (ppm)

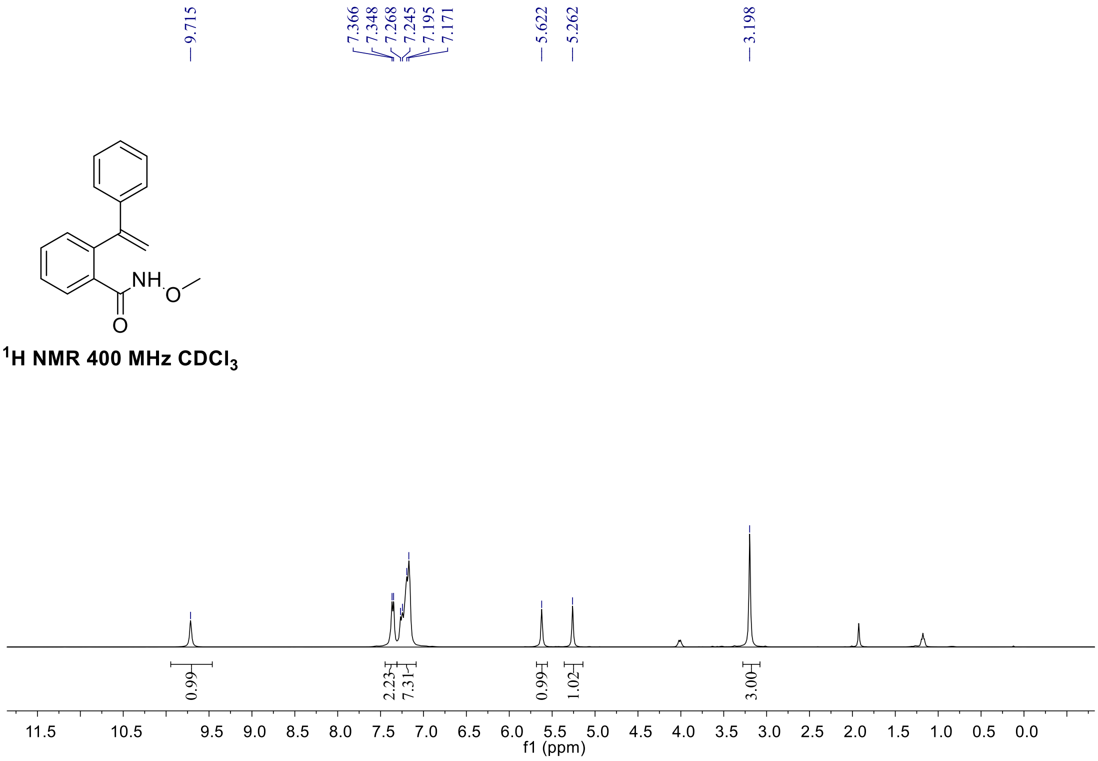
9.715
7.366
7.348
7.268
7.245
7.195
7.171
5.622
5.262
3.198
NH
O
O
^{1}H NMR 400 MHz CDCl$_3$
0.99
2.23
7.31
0.99
1.02
3.00
f1 (ppm)

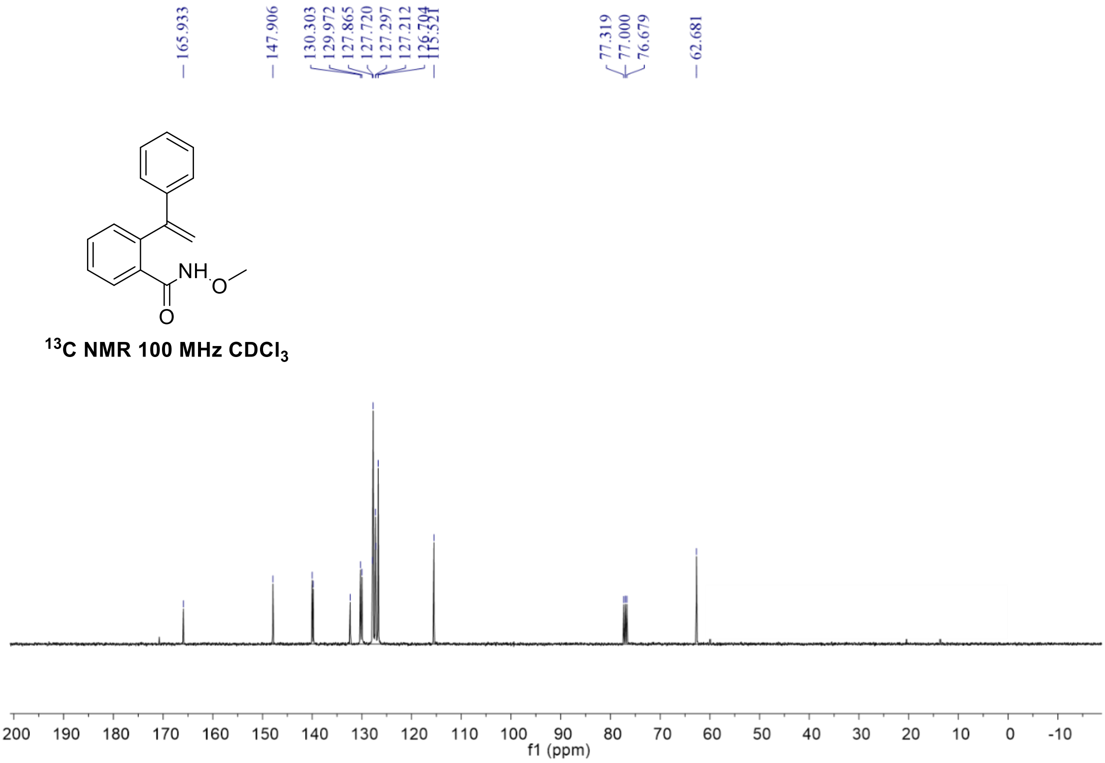
165.933
147.906
130.303
129.972
127.865
127.720
127.297
127.212
126.704
115.521
77.319
77.000
76.679
62.681
NH
O
O
^{13}C NMR 100 MHz CDCl$_3$
f1 (ppm)

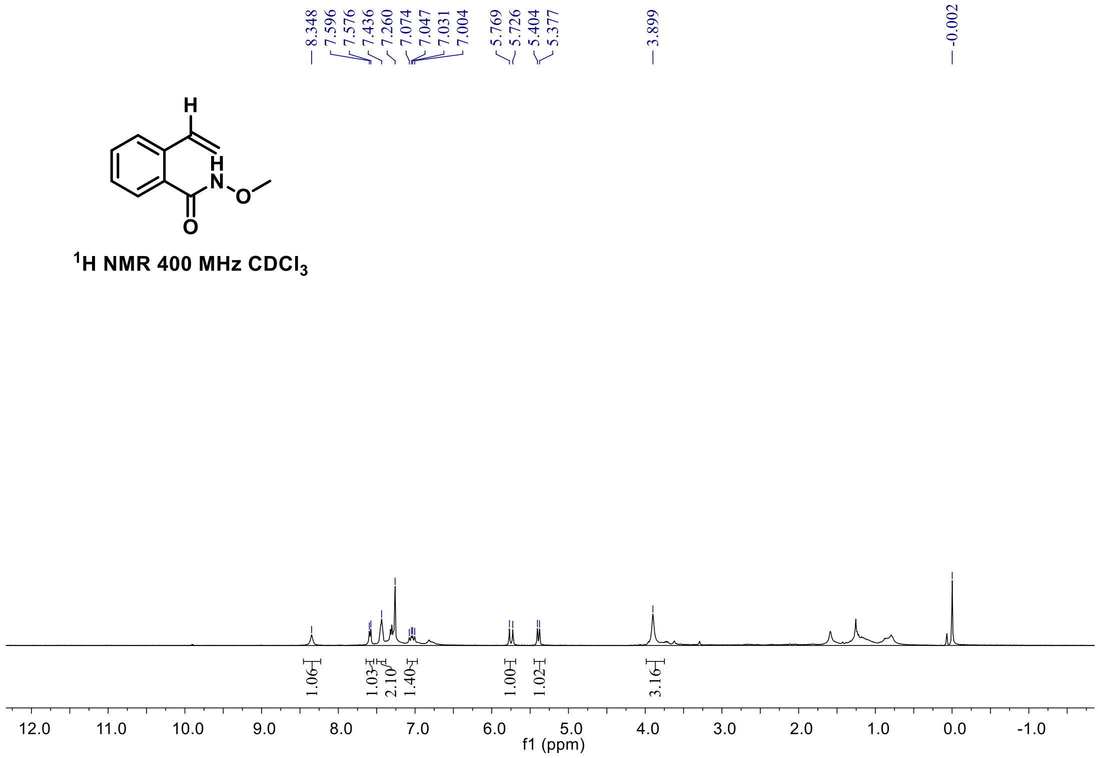
8.348
7.596
7.576
7.436
7.260
7.074
7.047
7.031
7.004
5.769
5.726
5.404
5.377
3.899
-0.002
H
N
H
O
O
1H NMR 400 MHz CDCl3
1.06
1.03
2.10
1.40
1.00
1.02
3.16
12.0 11.0 10.0 9.0 8.0 7.0 6.0 5.0 4.0 3.0 2.0 1.0 0.0 -1.0
f1 (ppm)

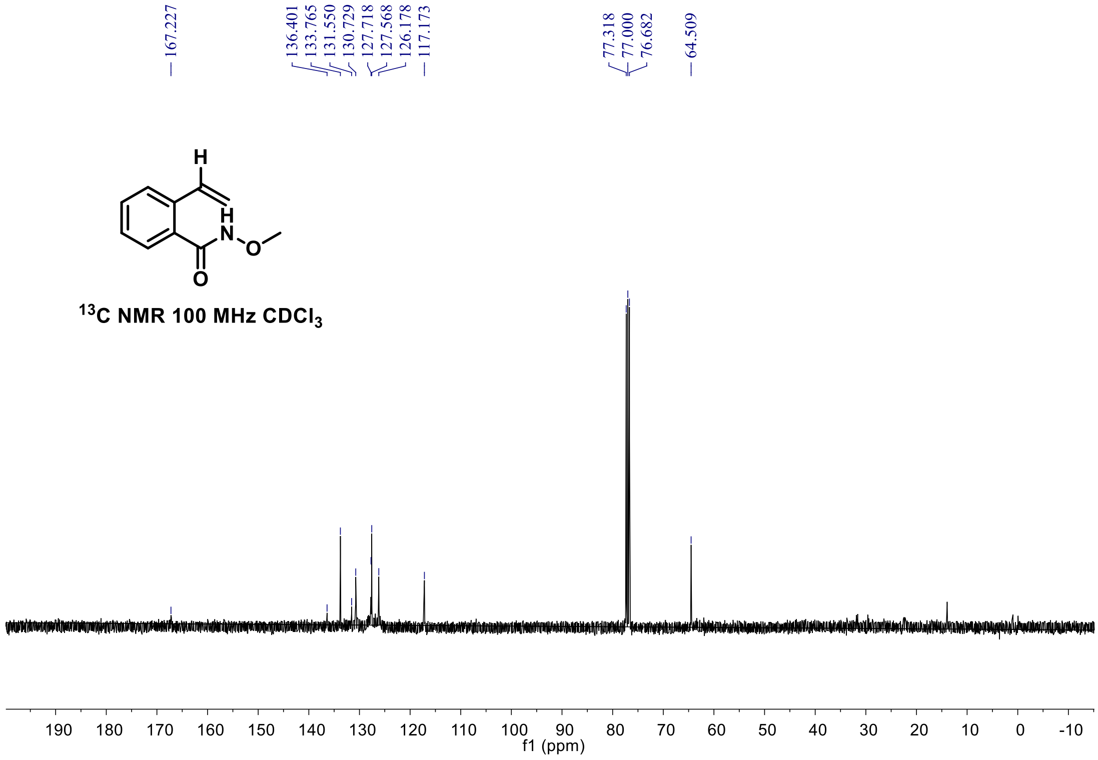
167.227
136.401
133.765
131.550
130.729
127.718
127.568
126.178
117.173
77.318
77.000
76.682
64.509
H
N
H
O
O
13C NMR 100 MHz CDCl3
190 180 170 160 150 140 130 120 110 100 90 80 70 60 50 40 30 20 10 0 -10
f1 (ppm)

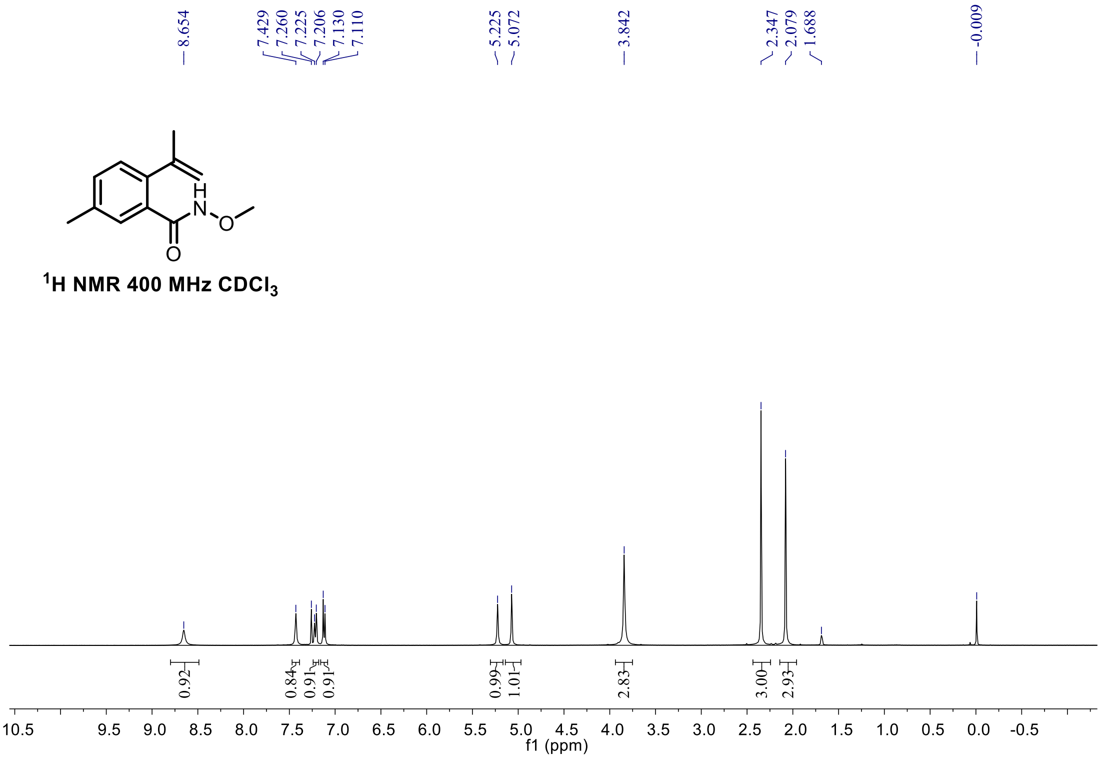
8.654
7.429
7.260
7.225
7.206
7.130
7.110
5.225
5.072
3.842
2.347
2.079
1.688
-0.009
H
N
O
O
1H NMR 400 MHz CDCl3
0.92
0.84
0.91
0.91
0.99
1.01
2.83
3.00
2.93
f1 (ppm)

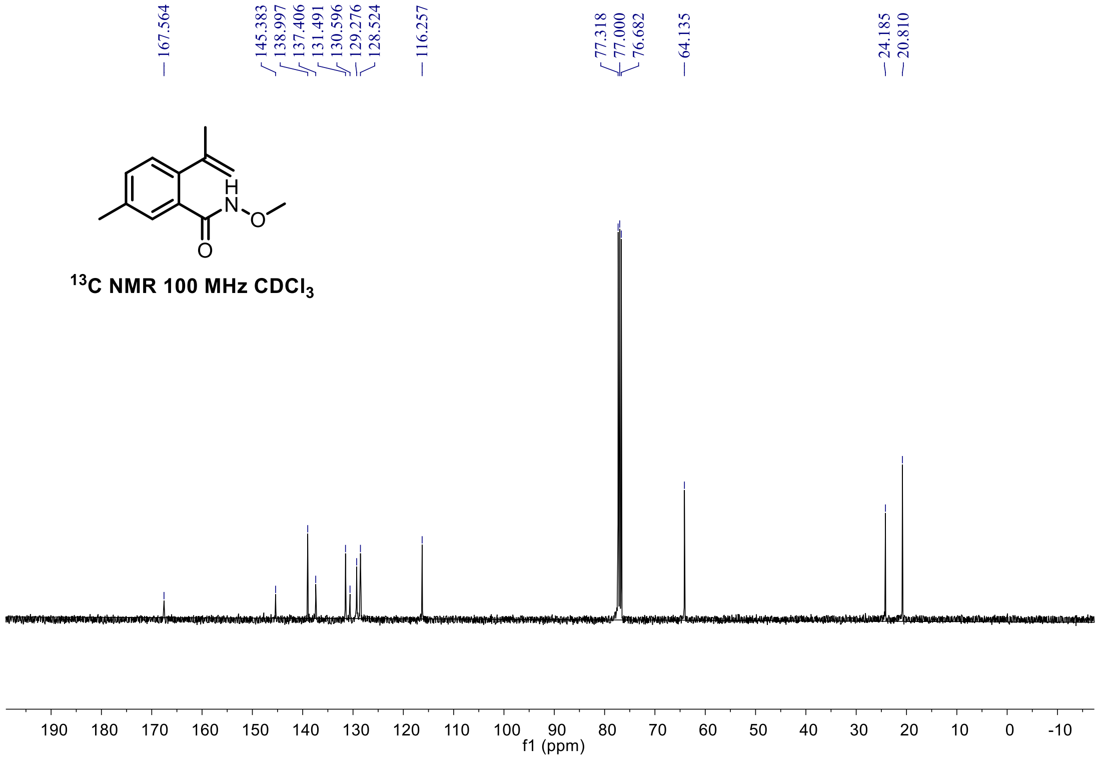
167.564
145.383
138.997
137.406
131.491
130.596
129.276
128.524
116.257
77.318
77.000
76.682
64.135
24.185
20.810
H
N
O
O
13C NMR 100 MHz CDCl3
f1 (ppm)

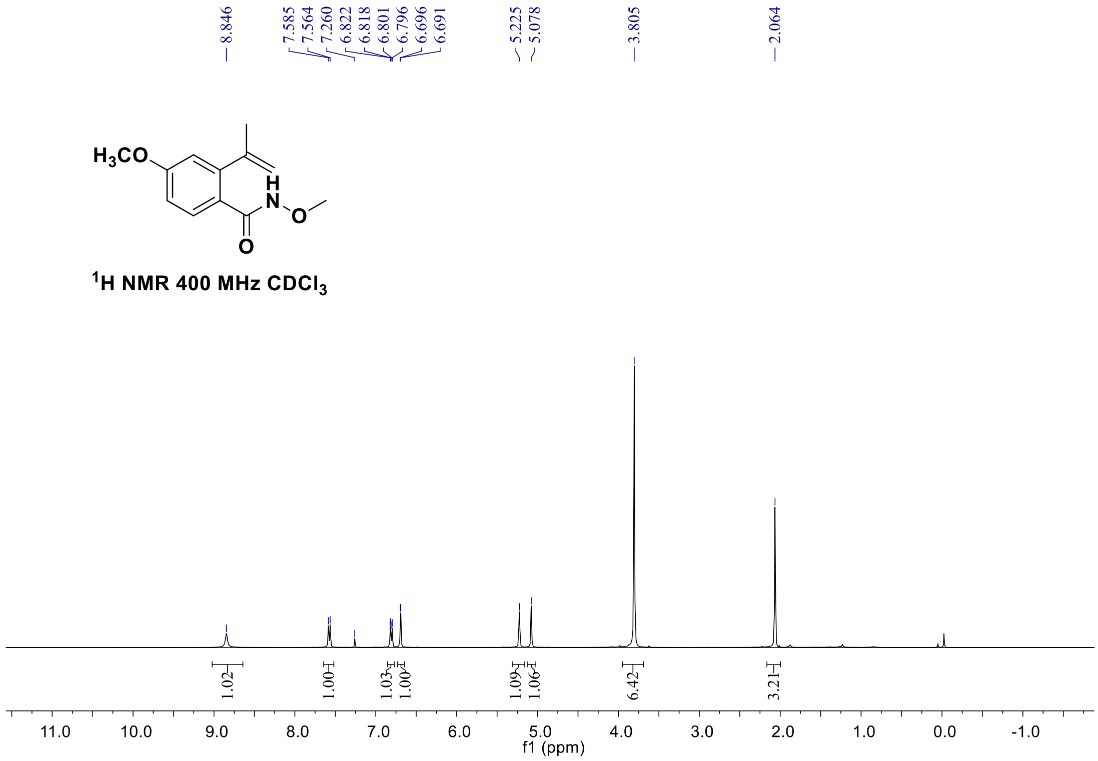
8.846
7.585
7.564
7.260
6.822
6.818
6.801
6.796
6.696
6.691
5.225
5.078
3.805
2.064
H3CO
H
N
O
O
1H NMR 400 MHz CDCl3
1.02
1.00
1.03
1.00
1.09
1.06
6.42
3.21
11.0
10.0
9.0
8.0
7.0
6.0
5.0
4.0
3.0
2.0
1.0
0.0
-1.0
f1 (ppm)

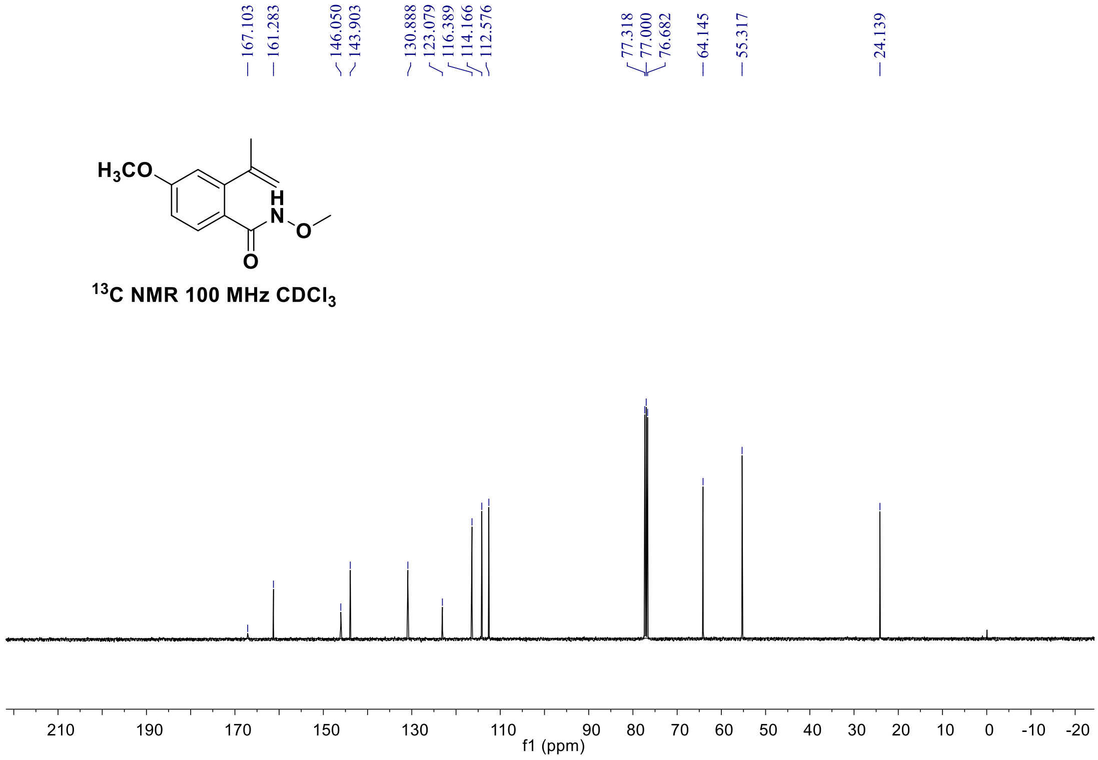
167.103
161.283
146.050
143.903
130.888
123.079
116.389
114.166
112.576
77.318
77.000
76.682
64.145
55.317
24.139
H3CO
H
N
O
O
13C NMR 100 MHz CDCl3
210
190
170
150
130
110
90
80
70
60
50
40
30
20
10
0
-10
-20
f1 (ppm)

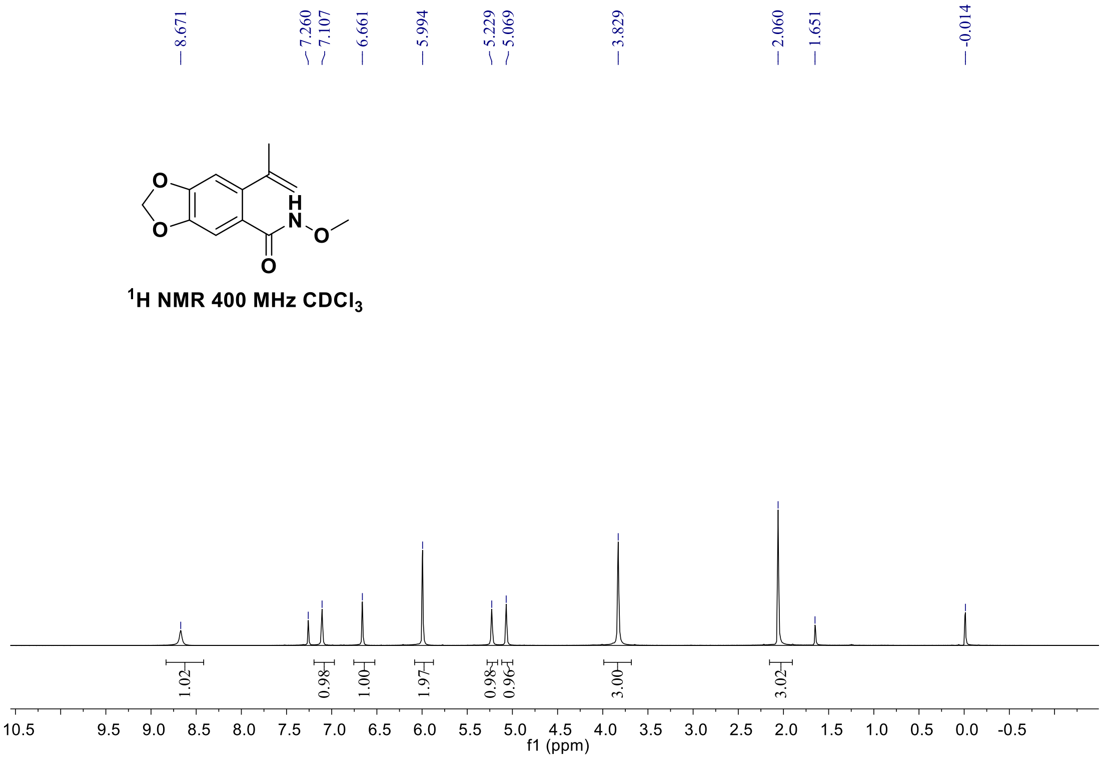
8.671
7.260
7.107
6.661
5.994
5.229
5.069
3.829
2.060
1.651
-0.014
H
N
O
O
O
O
1H NMR 400 MHz CDCl3
1.02
0.98
1.00
1.97
0.98
0.96
3.00
3.02
10.5 9.5 9.0 8.5 8.0 7.5 7.0 6.5 6.0 5.5 5.0 4.5 4.0 3.5 3.0 2.5 2.0 1.5 1.0 0.5 0.0 -0.5
f1 (ppm)

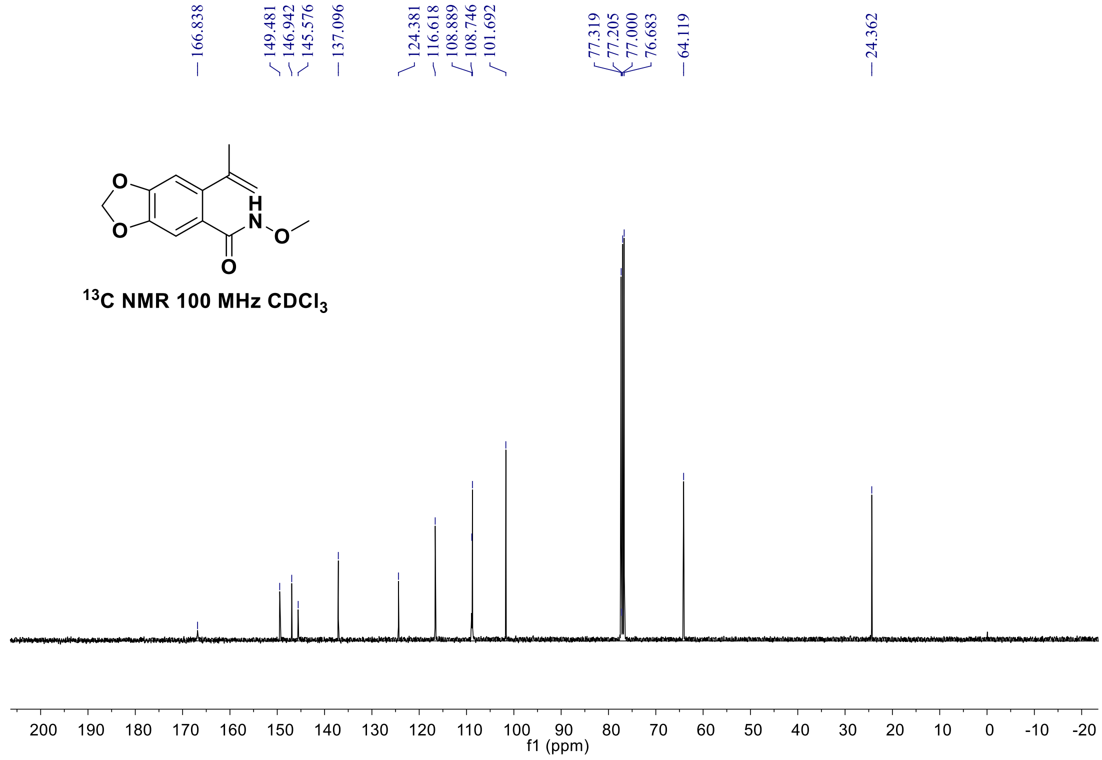
166.838
149.481
146.942
145.576
137.096
124.381
116.618
108.889
108.746
101.692
77.319
77.205
77.000
76.683
64.119
24.362
H
N
O
O
O
O
13C NMR 100 MHz CDCl3
200 190 180 170 160 150 140 130 120 110 100 90 80 70 60 50 40 30 20 10 0 -10 -20
f1 (ppm)

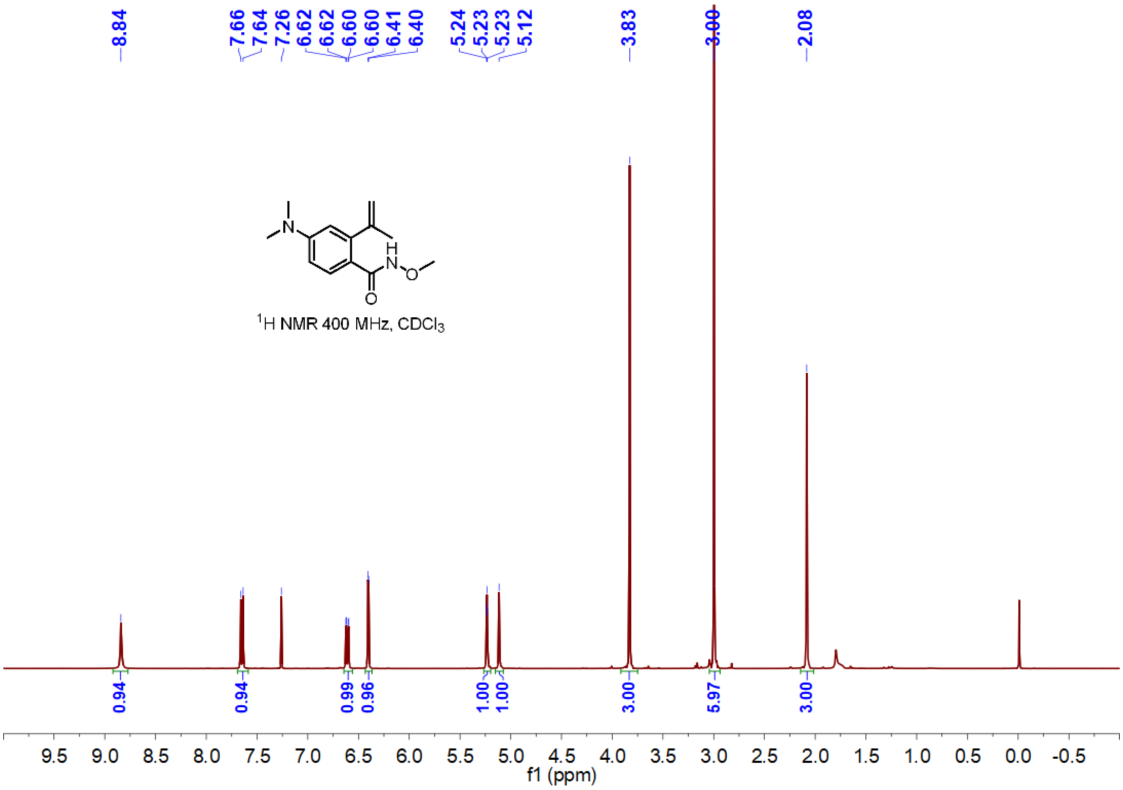
8.84
7.66
7.64
7.26
6.62
6.62
6.60
6.60
6.41
6.40
5.24
5.23
5.23
5.12
3.83
3.00
2.08
N
H
N
O
O
1H NMR 400 MHz, CDCl3
0.94
0.94
0.99
0.96
1.00
1.00
3.00
5.97
3.00
f1 (ppm)

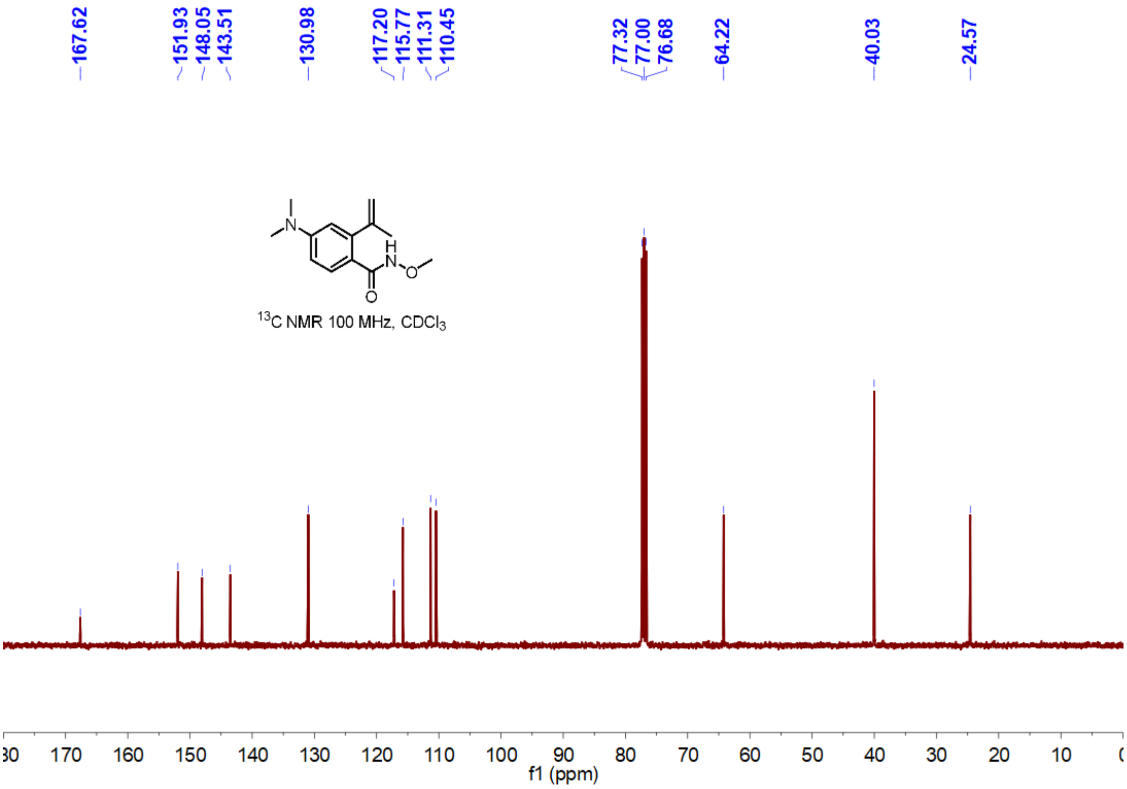
167.62
151.93
148.05
143.51
130.98
117.20
115.77
111.31
110.45
77.32
77.00
76.68
64.22
40.03
24.57
N
H
N
O
O
13C NMR 100 MHz, CDCl3
f1 (ppm)

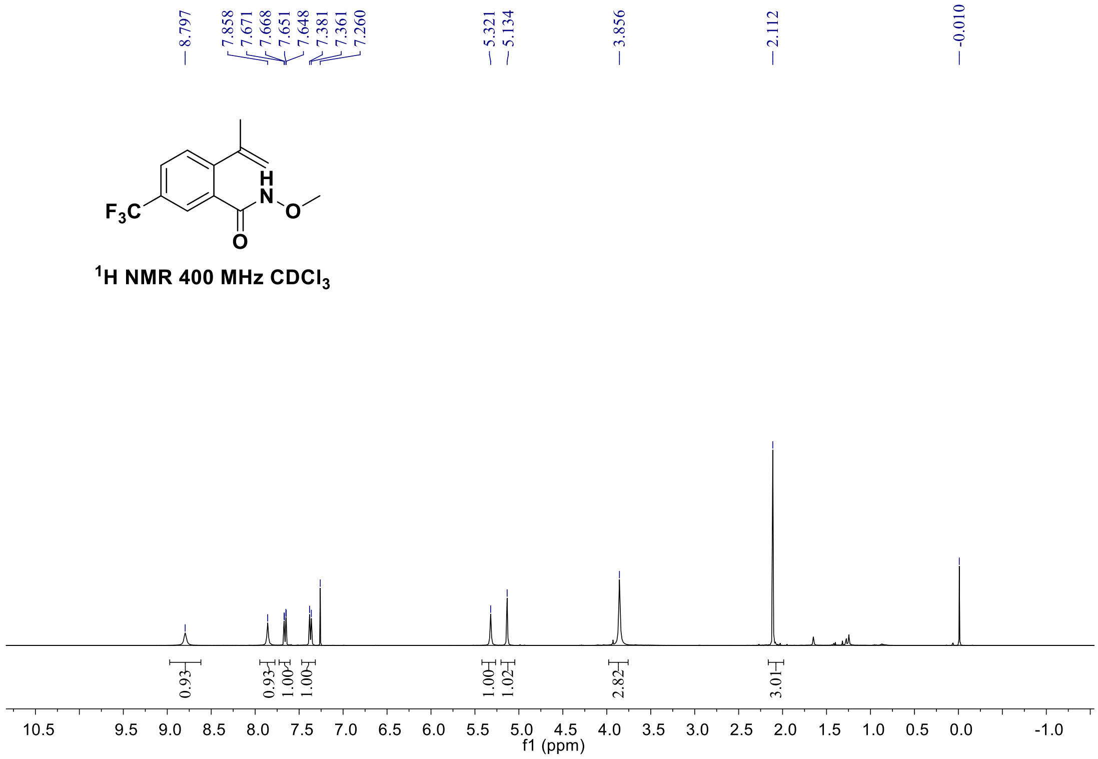
8.797
7.858
7.671
7.668
7.651
7.648
7.381
7.361
7.260
5.321
5.134
3.856
2.112
-0.010
F$_3$C
H
N
O
O
^{1}H NMR 400 MHz CDCl$_3$
0.93
0.93
1.00
1.00
1.00
1.02
2.82
3.01
f1 (ppm)

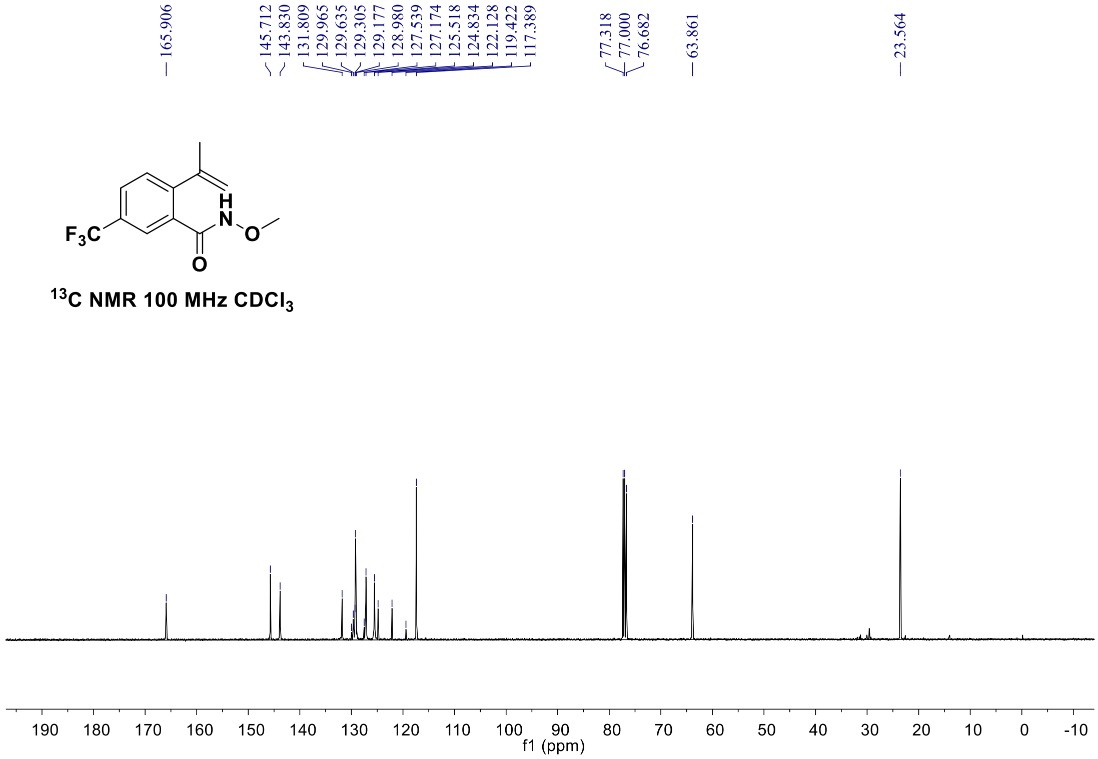
165.906
145.712
143.830
131.809
129.965
129.635
129.305
129.177
128.980
127.539
127.174
125.518
124.834
122.128
119.422
117.389
77.318
77.000
76.682
63.861
23.564
F$_3$C
H
N
O
O
^{13}C NMR 100 MHz CDCl$_3$
f1 (ppm)

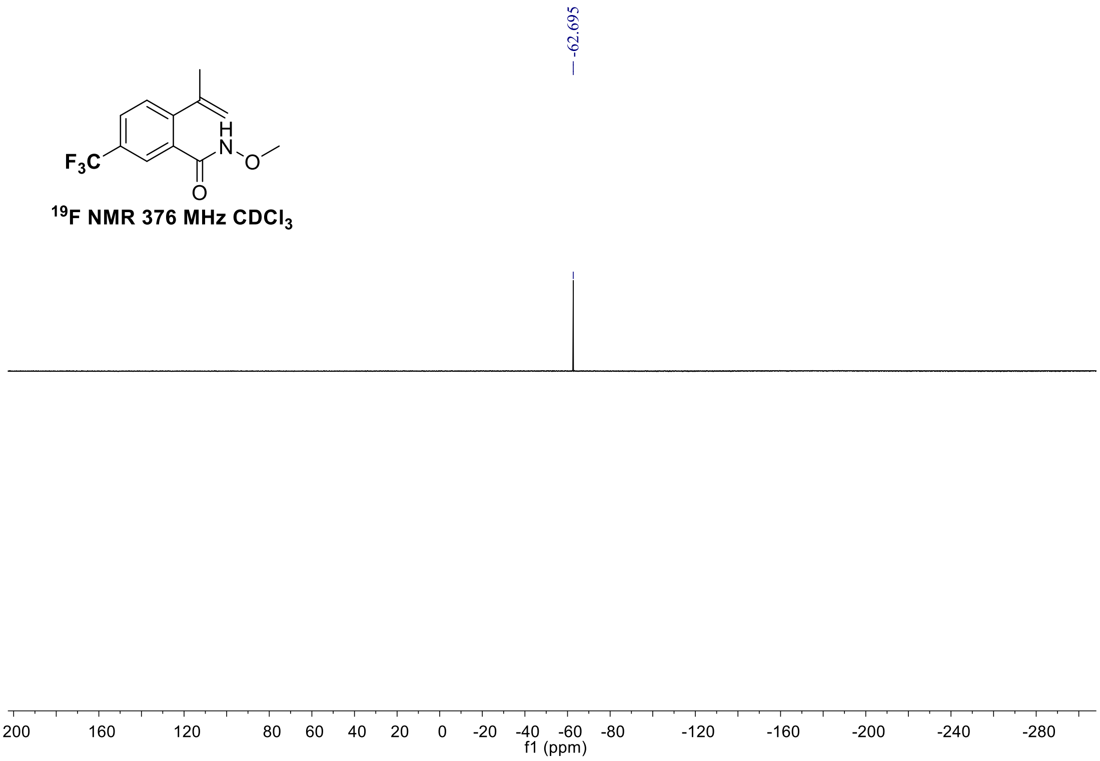

-62.695
F3C
H
N
O
O
19F NMR 376 MHz CDCl3
200 160 120 80 60 40 20 0 -20 -40 -60 -80 -120 -160 -200 -240 -280
f1 (ppm)

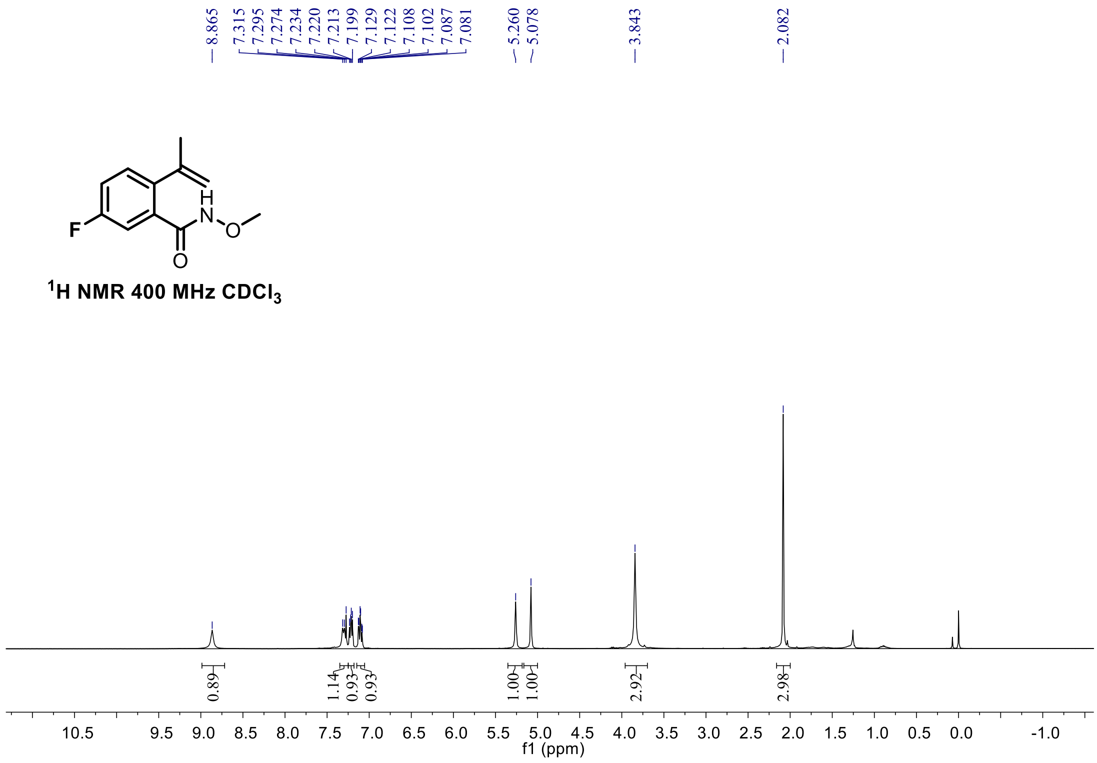

8.865
7.315
7.295
7.274
7.234
7.220
7.213
7.199
7.129
7.122
7.108
7.102
7.087
7.081
5.260
5.078
3.843
2.082
F
H
N
O
O
1H NMR 400 MHz CDCl3
0.89
1.14
0.93
0.93
1.00
1.00
2.92
2.98
10.5 9.5 9.0 8.5 8.0 7.5 7.0 6.5 6.0 5.5 5.0 4.5 4.0 3.5 3.0 2.5 2.0 1.5 1.0 0.5 0.0 -1.0
f1 (ppm)

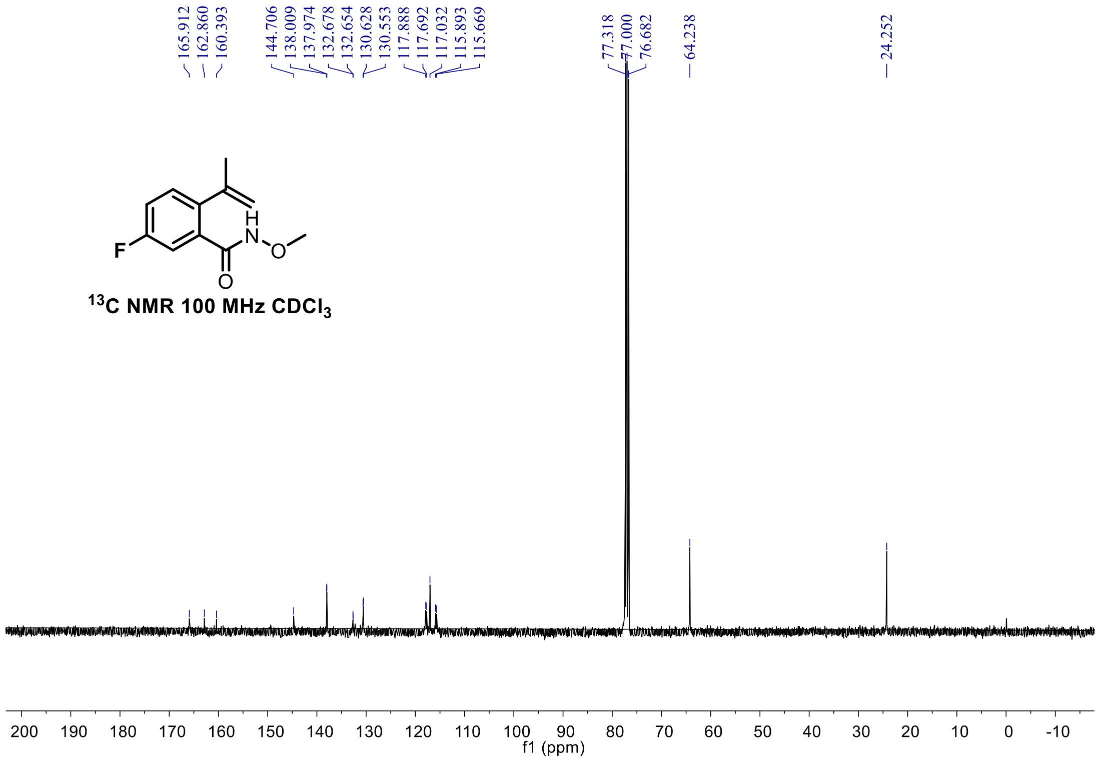

165.912
162.860
160.393
144.706
138.009
137.974
132.678
132.654
130.628
130.553
117.888
117.692
117.032
115.893
115.669
77.318
77.000
76.682
64.238
24.252
F
H
N
O
O
13C NMR 100 MHz CDCl3
200 190 180 170 160 150 140 130 120 110 100 90 80 70 60 50 40 30 20 10 0 -10
f1 (ppm)

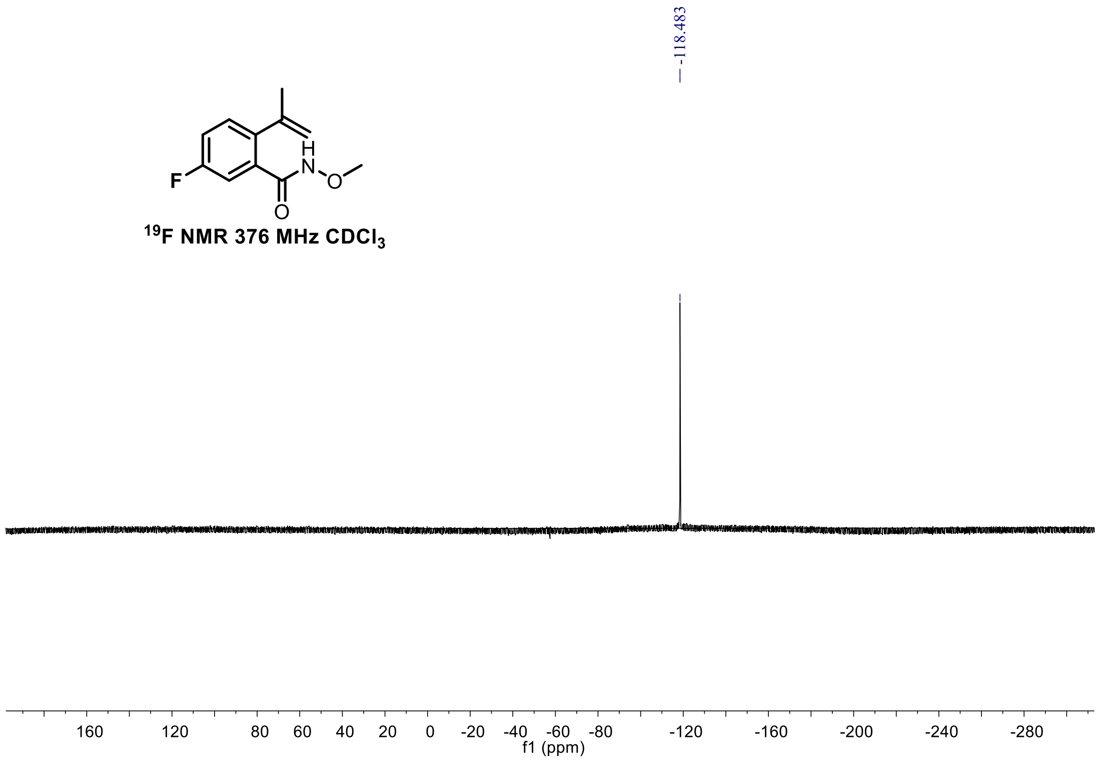

-118.483
F
H
N
O
O
19F NMR 376 MHz CDCl3
160 120 80 60 40 20 0 -20 -40 -60 -80 -120 -160 -200 -240 -280
f1 (ppm)

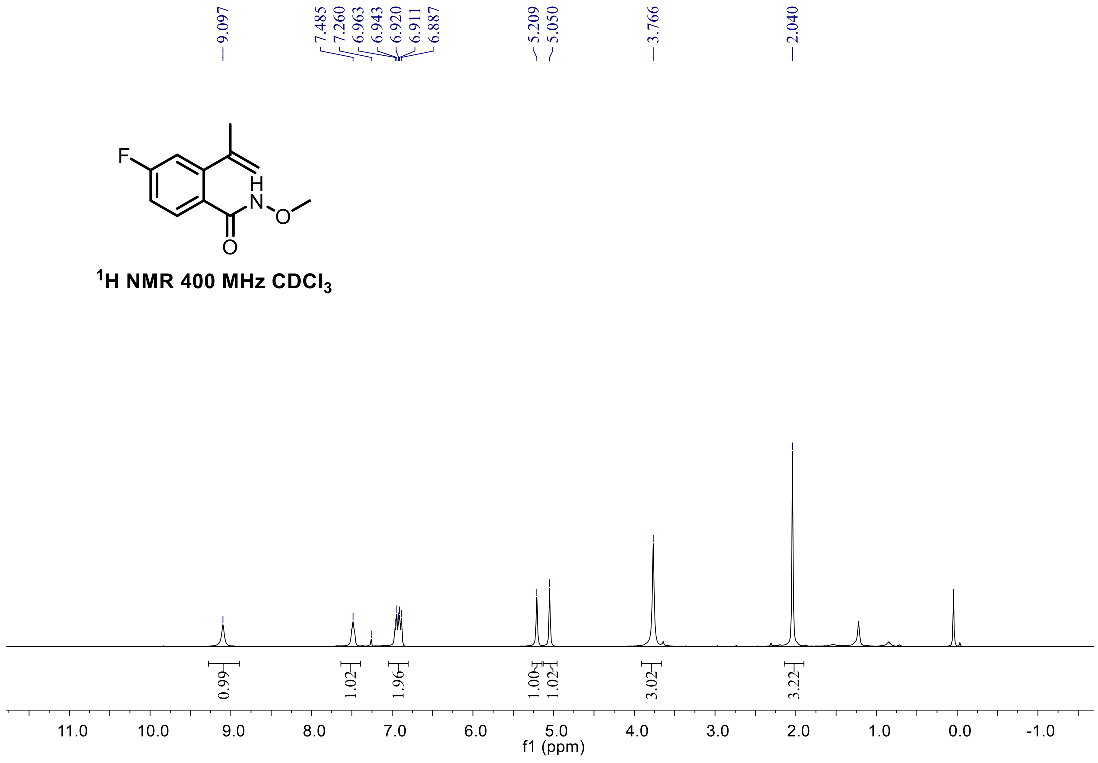

^{1}H NMR 400 MHz $CDCl_3$

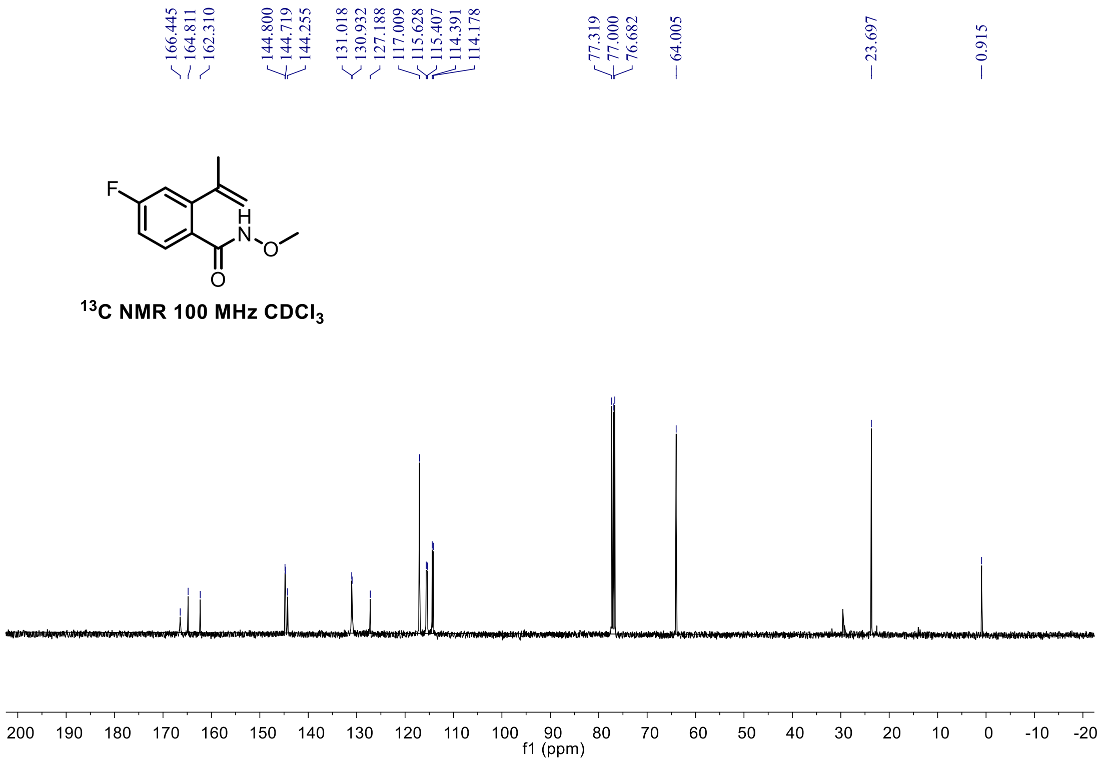

^{13}C NMR 100 MHz $CDCl_3$

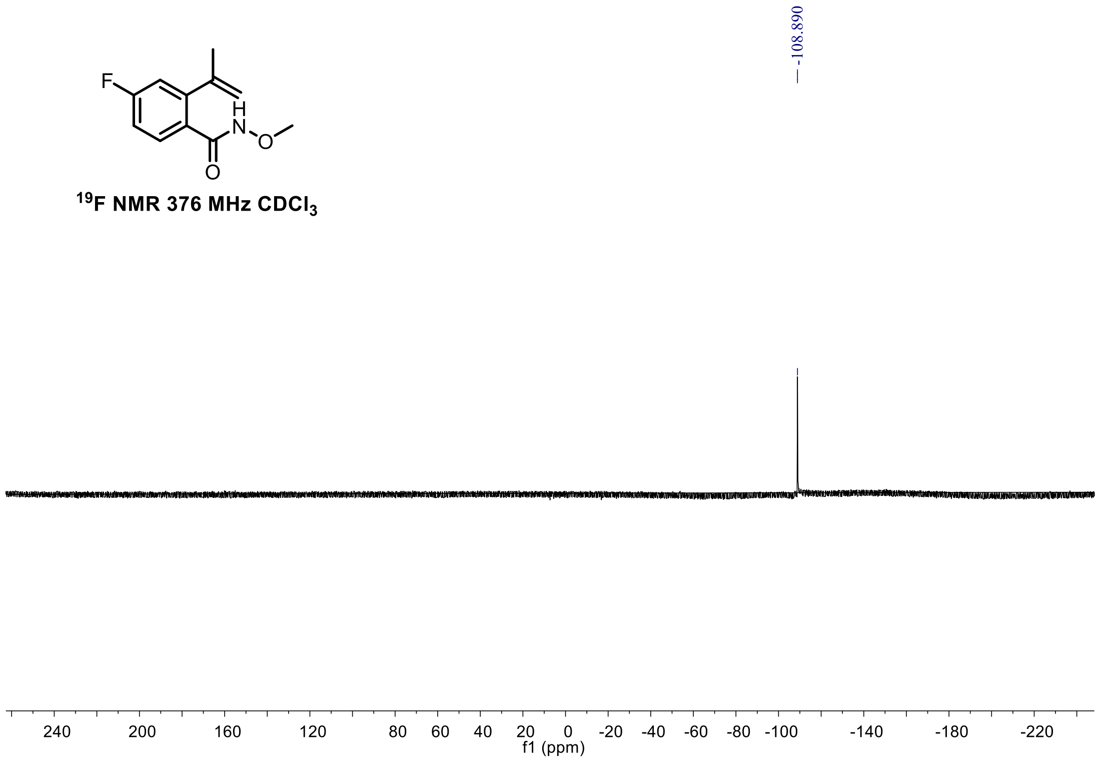

-108.890
F
H
N
O
O
19F NMR 376 MHz CDCl3
240 200 160 120 80 60 40 20 0 -20 -40 -60 -80 -100 -140 -180 -220
f1 (ppm)

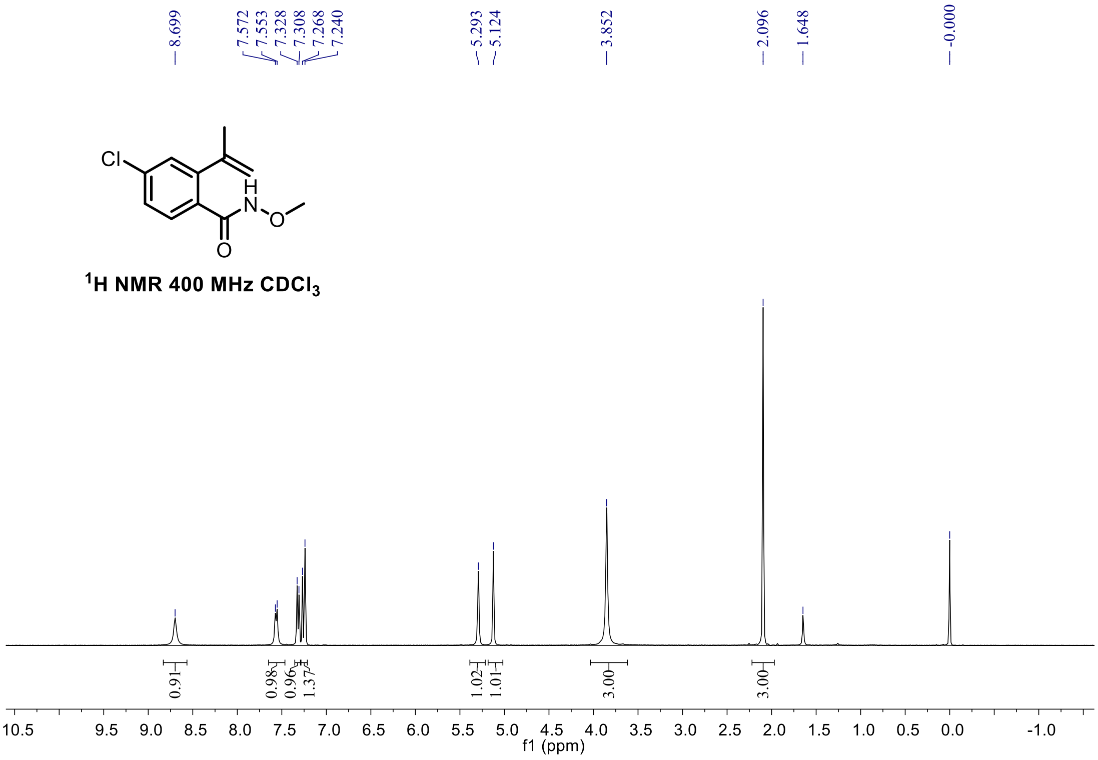

8.699
7.572
7.553
7.328
7.308
7.268
7.240
5.293
5.124
3.852
2.096
1.648
-0.000
Cl
H
N
O
O
1H NMR 400 MHz CDCl3
0.91
0.98
0.96
1.37
1.02
1.01
3.00
3.00
10.5 9.5 9.0 8.5 8.0 7.5 7.0 6.5 6.0 5.5 5.0 4.5 4.0 3.5 3.0 2.5 2.0 1.5 1.0 0.5 0.0 -1.0
f1 (ppm)

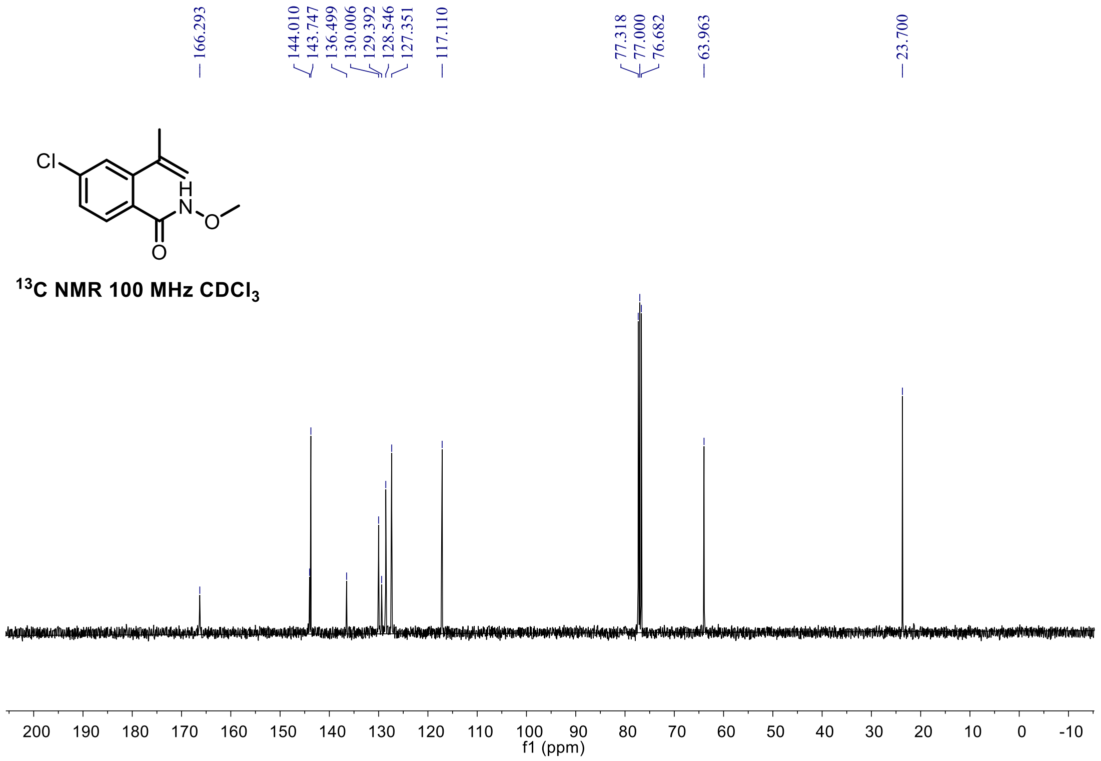
166.293
144.010
143.747
136.499
130.006
129.392
128.546
127.351
117.110
77.318
77.000
76.682
63.963
23.700
Cl
H
N
O
O
13C NMR 100 MHz CDCl3
f1 (ppm)

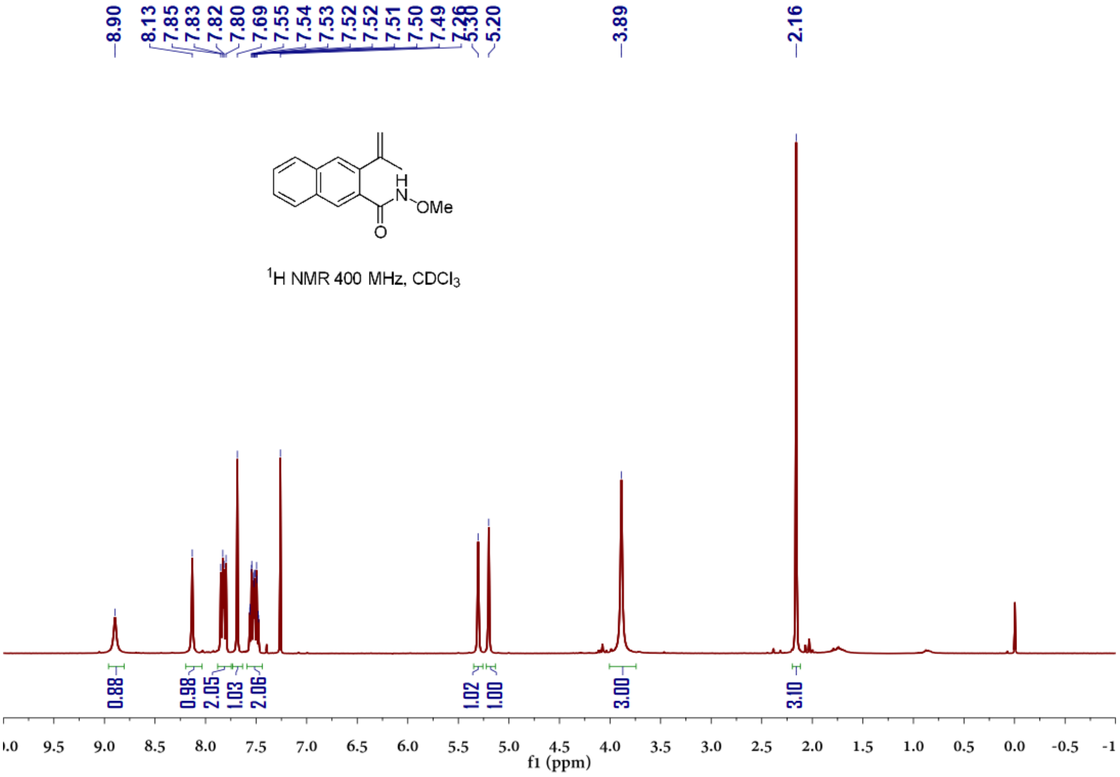
8.90
8.13
7.85
7.83
7.82
7.80
7.69
7.55
7.54
7.53
7.52
7.52
7.51
7.50
7.49
7.26
5.30
5.20
3.89
2.16
H
N
OMe
O
1H NMR 400 MHz, CDCl3
0.88
0.98
2.05
1.03
2.06
1.02
1.00
3.00
3.10
f1 (ppm)

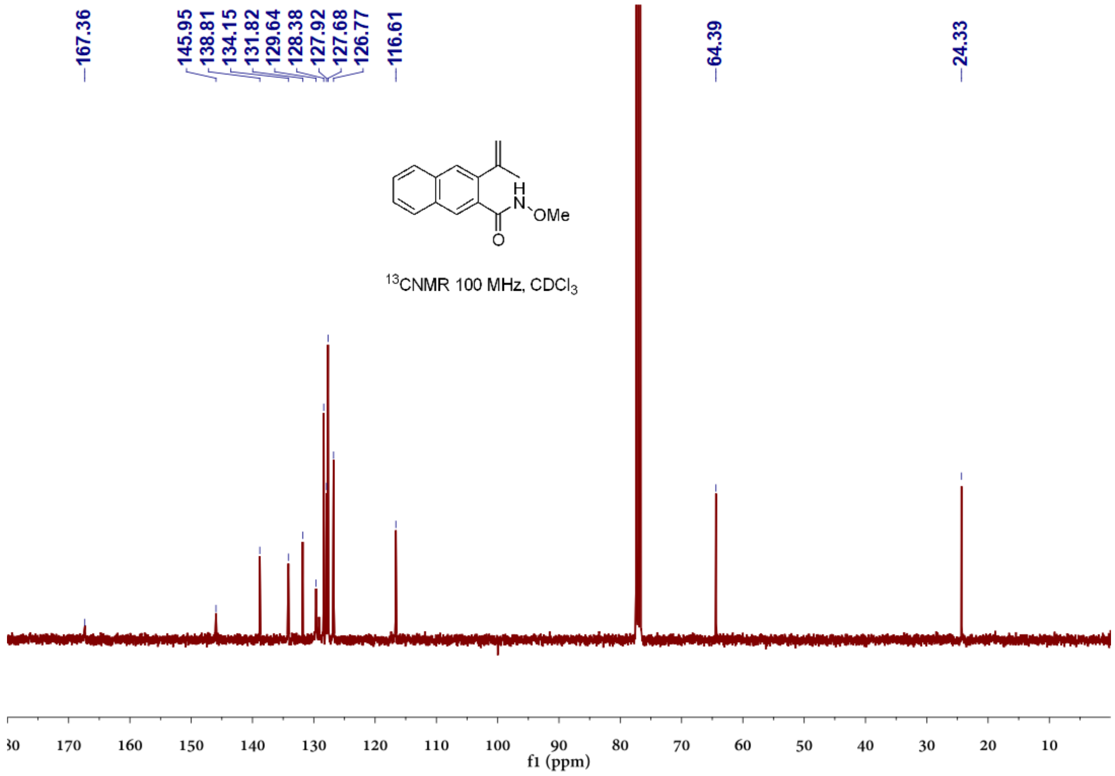
167.36
145.95
138.81
134.15
131.82
129.64
128.38
127.92
127.68
126.77
116.61
64.39
24.33
N
H
OMe
O
13CNMR 100 MHz, CDCl3
f1 (ppm)

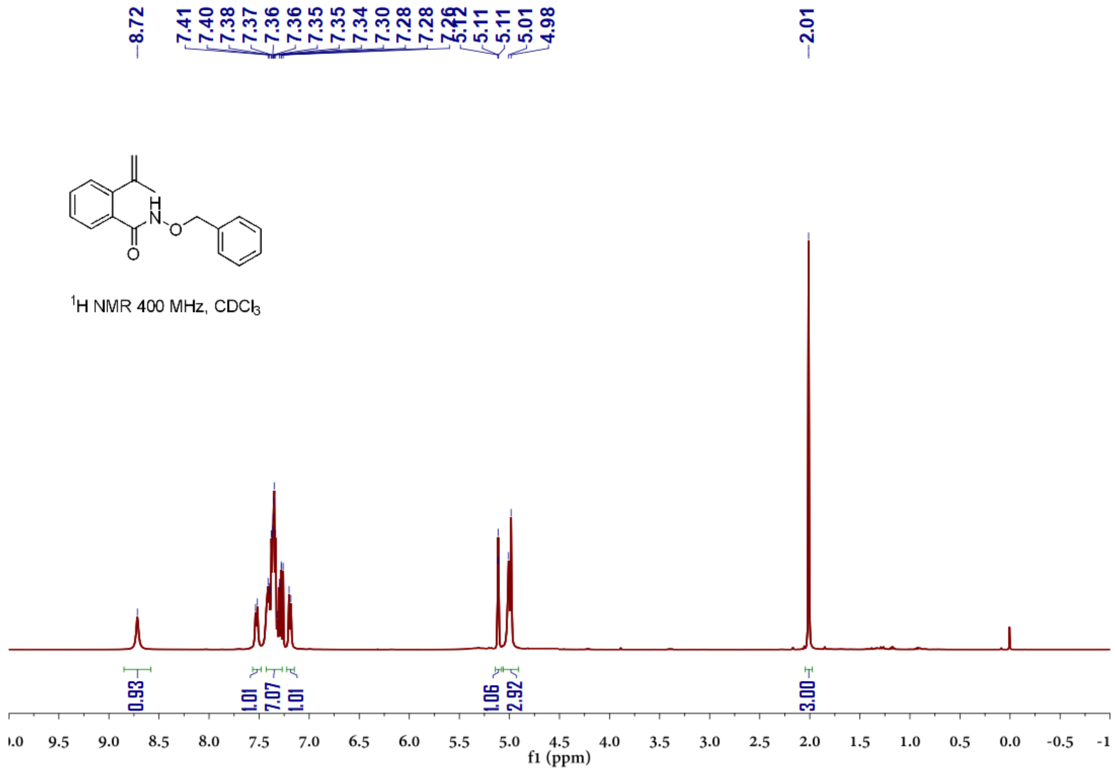
8.72
7.41
7.40
7.38
7.37
7.36
7.36
7.35
7.35
7.34
7.30
7.28
7.28
7.26
5.12
5.11
5.11
5.01
4.98
2.01
H
N
O
O
1H NMR 400 MHz, CDCl3
0.93
1.01
7.07
1.01
1.06
2.92
3.00
f1 (ppm)

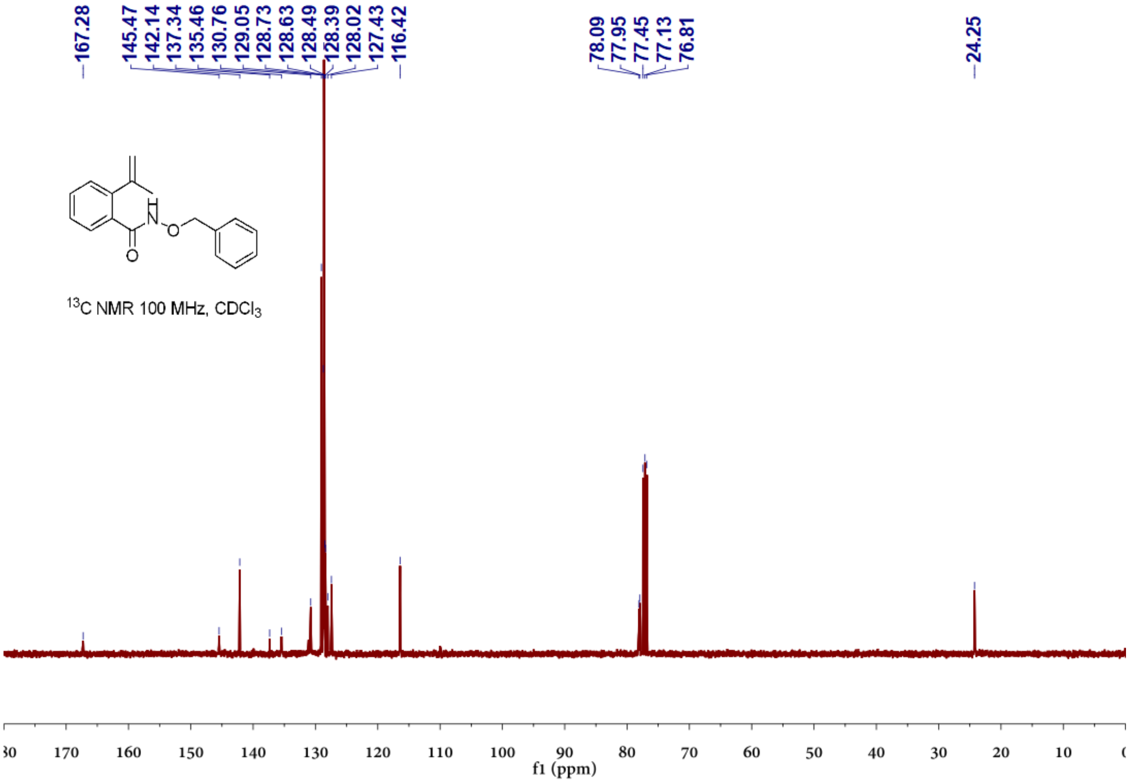
167.28
145.47
142.14
137.34
135.46
130.76
129.05
128.73
128.63
128.49
128.39
128.02
127.43
116.42
78.09
77.95
77.45
77.13
76.81
24.25
^{13}C NMR 100 MHz, CDCl$_3$
f1 (ppm)

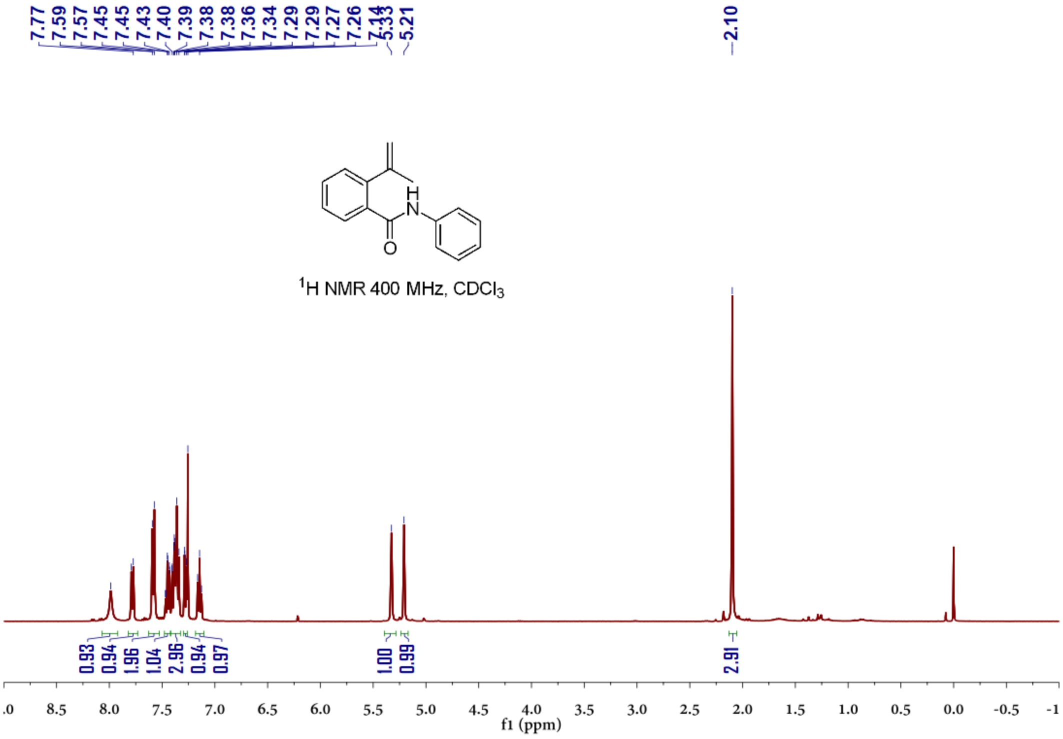
7.77
7.59
7.57
7.45
7.45
7.43
7.40
7.39
7.38
7.38
7.36
7.34
7.29
7.29
7.27
7.26
7.14
5.33
5.21
2.10
^{1}H NMR 400 MHz, CDCl$_3$
0.93
0.94
1.96
1.04
2.96
0.94
0.97
1.00
0.99
2.91
f1 (ppm)

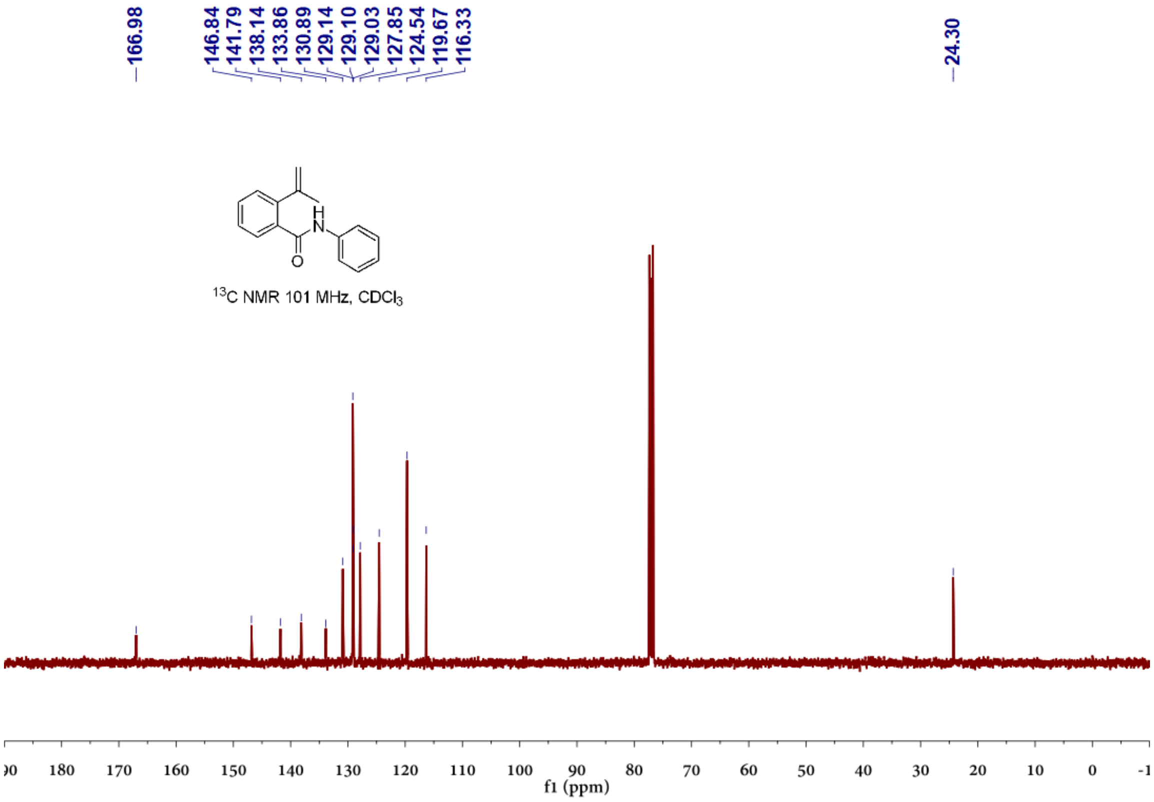

166.98
146.84
141.79
138.14
133.86
130.89
129.14
129.10
129.03
127.85
124.54
119.67
116.33
24.30
H
N
O
^{13}C NMR 101 MHz, CDCl$_3$
f1 (ppm)

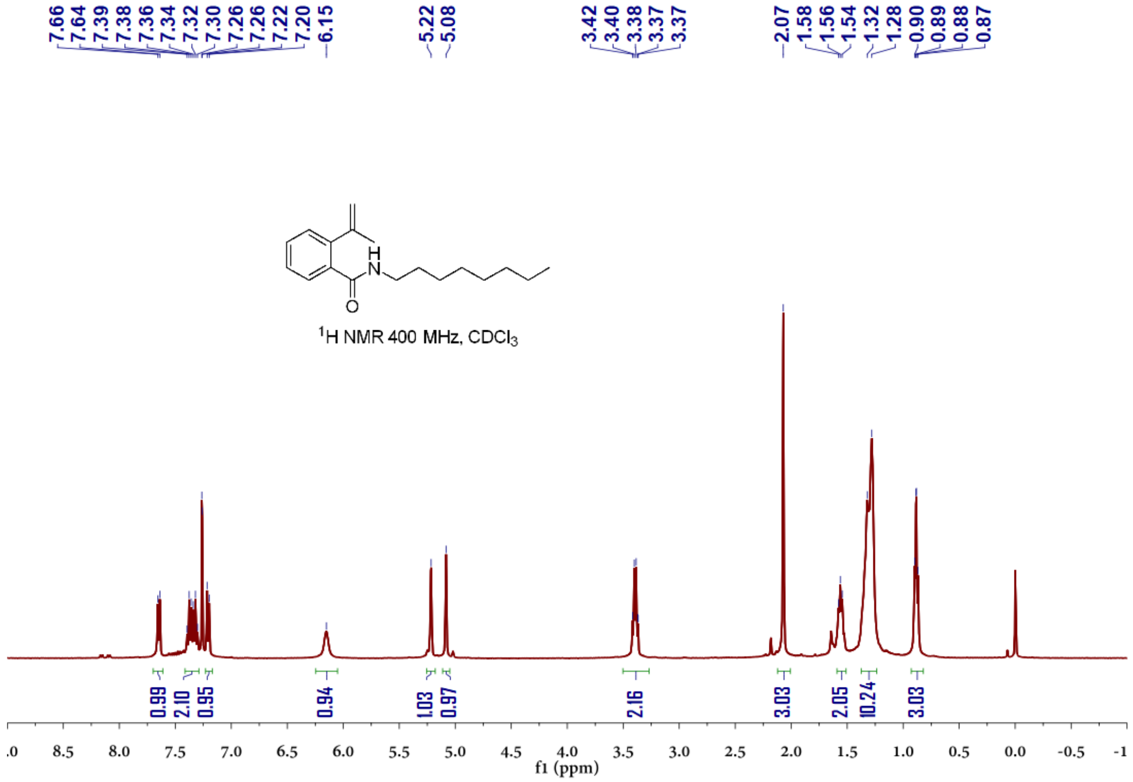

7.66
7.64
7.39
7.38
7.36
7.34
7.32
7.30
7.26
7.26
7.22
7.20
6.15
5.22
5.08
3.42
3.40
3.38
3.37
3.37
2.07
1.58
1.56
1.54
1.32
1.28
0.90
0.89
0.88
0.87
H
N
O
^{1}H NMR 400 MHz, CDCl$_3$
0.99
2.10
0.95
0.94
1.03
0.97
2.16
3.03
2.05
10.24
3.03
f1 (ppm)

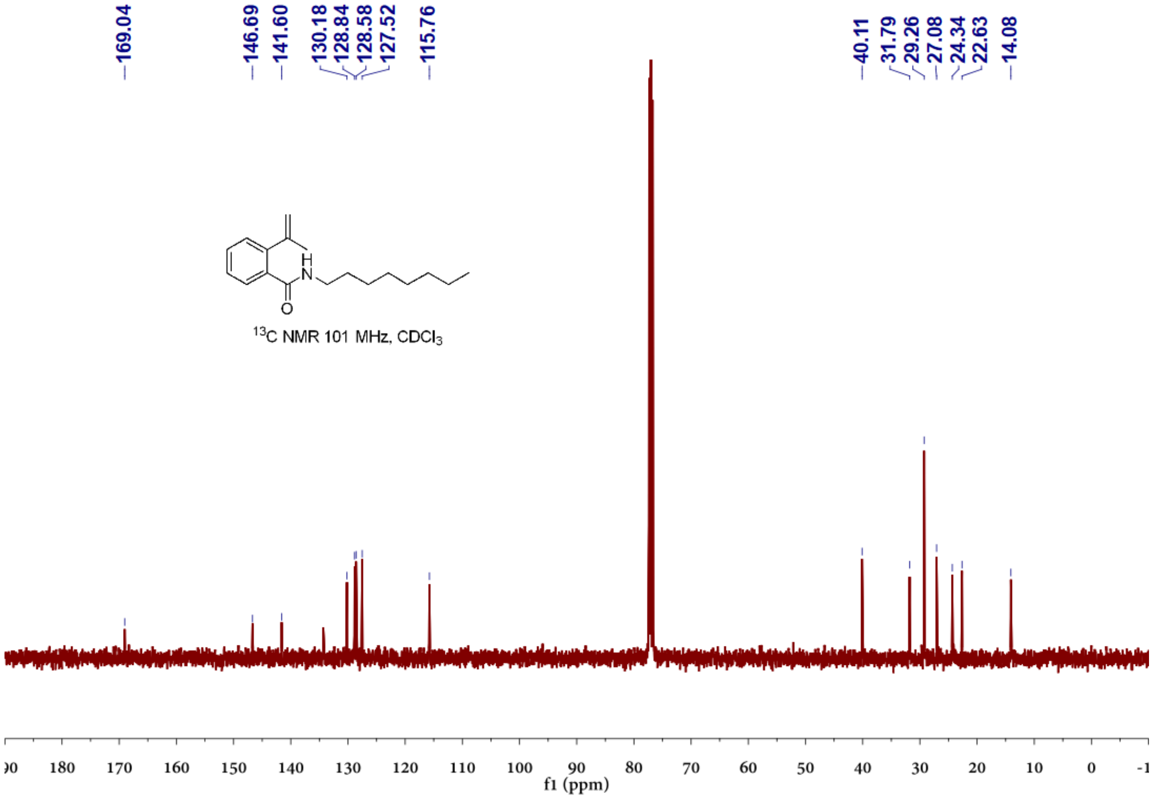
169.04
146.69
141.60
130.18
128.84
128.58
127.52
115.76
40.11
31.79
29.26
27.08
24.34
22.63
14.08
O
H
N
^{13}C NMR 101 MHz, CDCl$_3$
f1 (ppm)

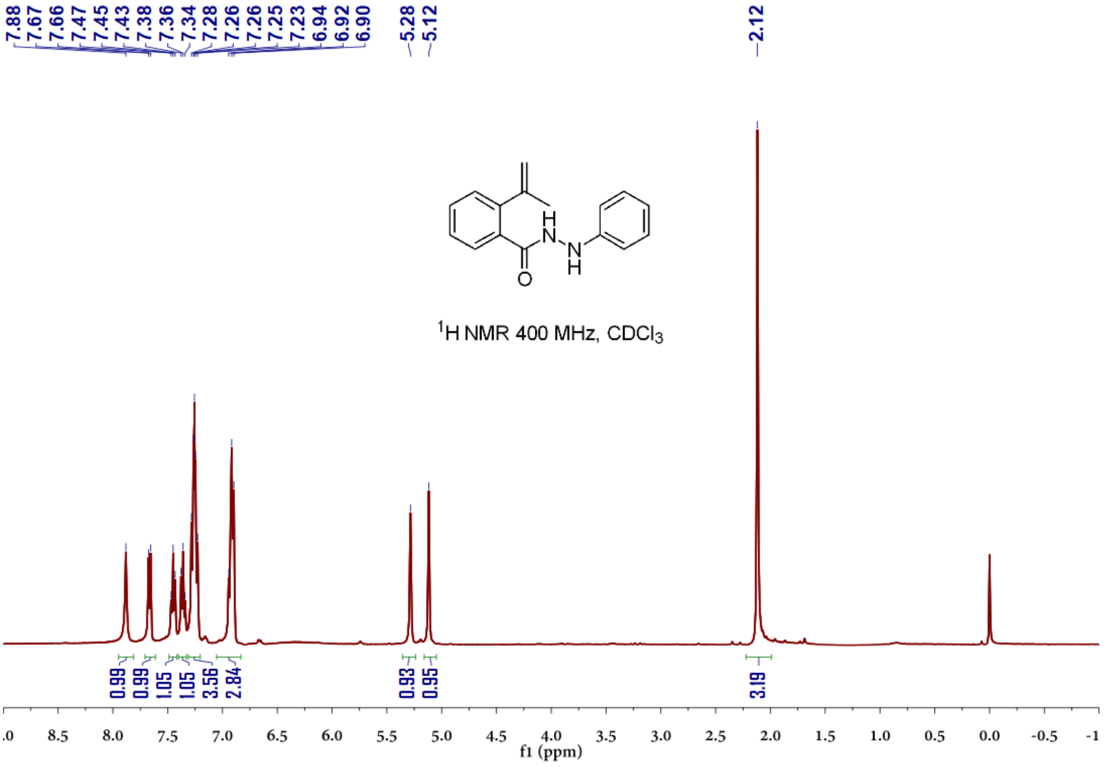
7.88
7.67
7.66
7.47
7.45
7.43
7.38
7.36
7.34
7.28
7.26
7.26
7.25
7.23
6.94
6.92
6.90
5.28
5.12
2.12
H
N
N
H
O
^{1}H NMR 400 MHz, CDCl$_3$
0.99
0.99
1.05
1.05
3.56
2.84
0.93
0.95
3.19
f1 (ppm)

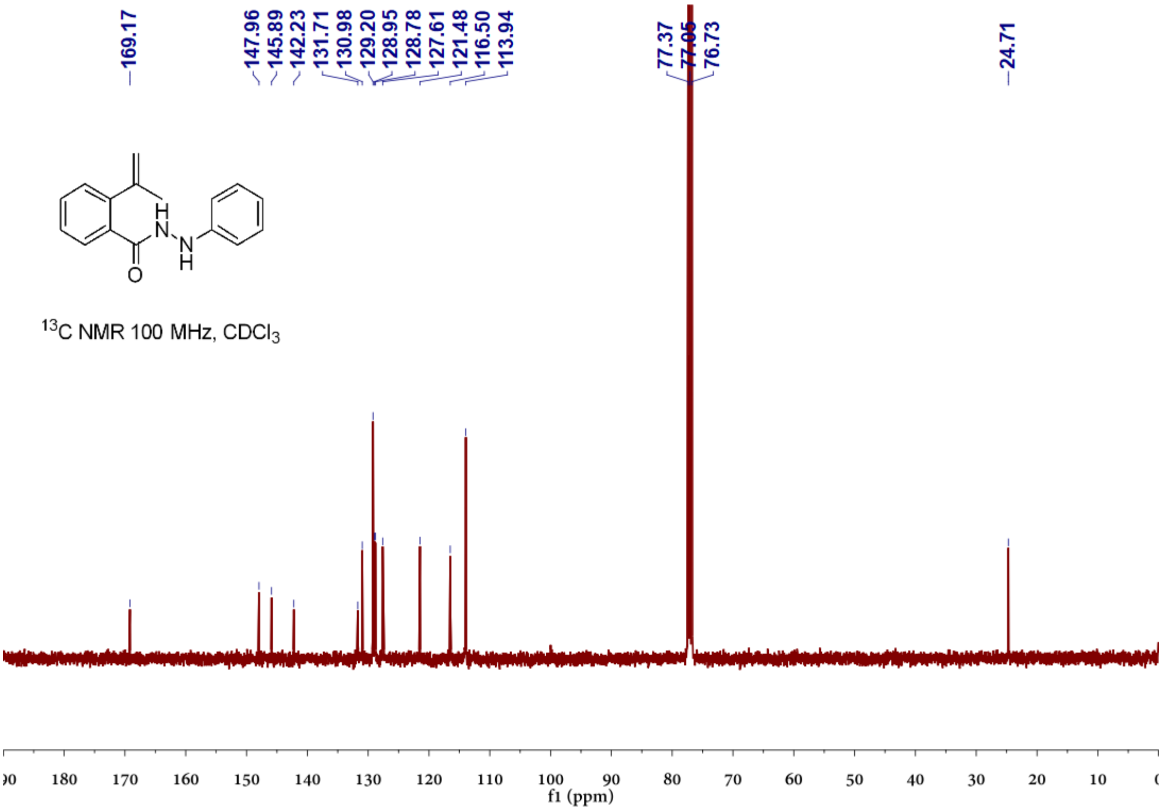

^{13}C NMR 100 MHz, $CDCl_3$

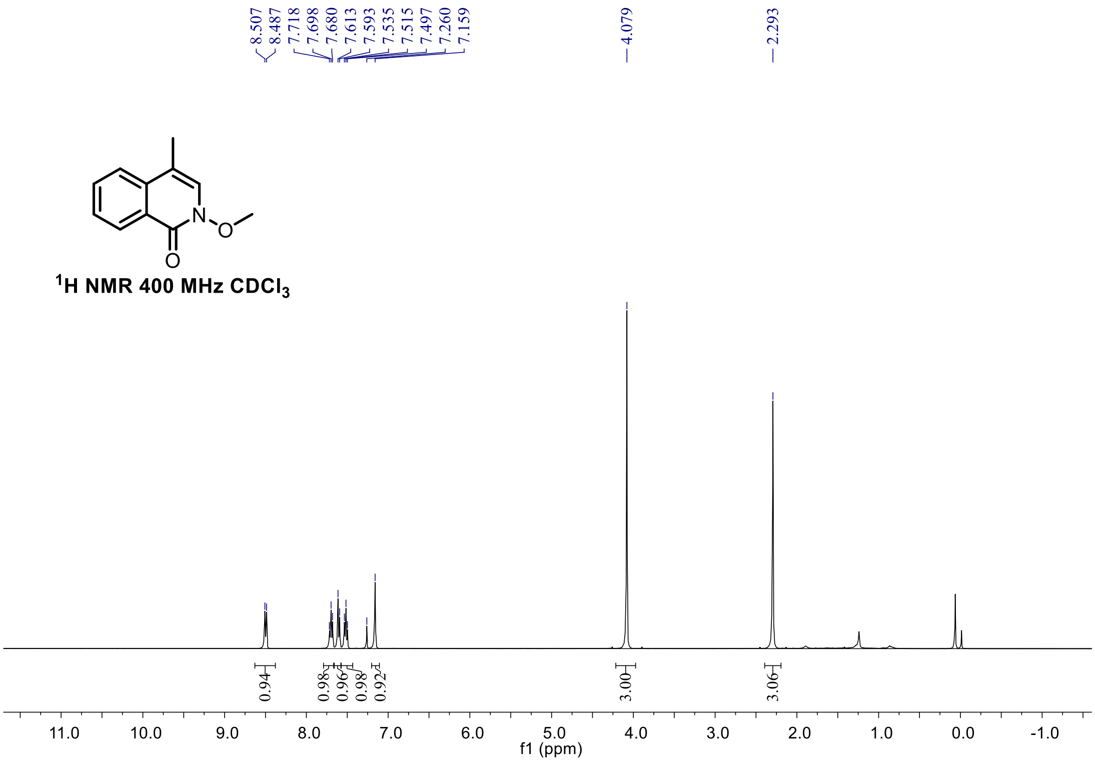

^{1}H NMR 400 MHz $CDCl_3$

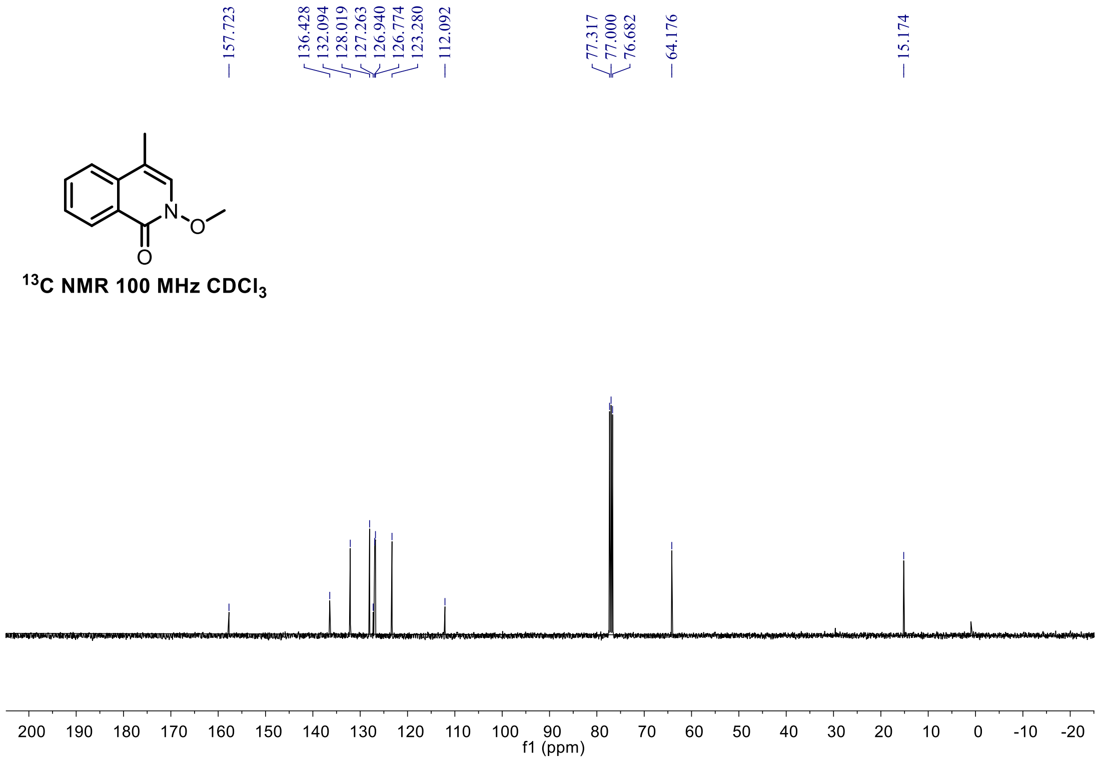
157.723
136.428
132.094
128.019
127.263
126.940
126.774
123.280
112.092
77.317
77.000
76.682
64.176
15.174
13C NMR 100 MHz CDCl3
200 190 180 170 160 150 140 130 120 110 100 90 80 70 60 50 40 30 20 10 0 -10 -20
f1 (ppm)

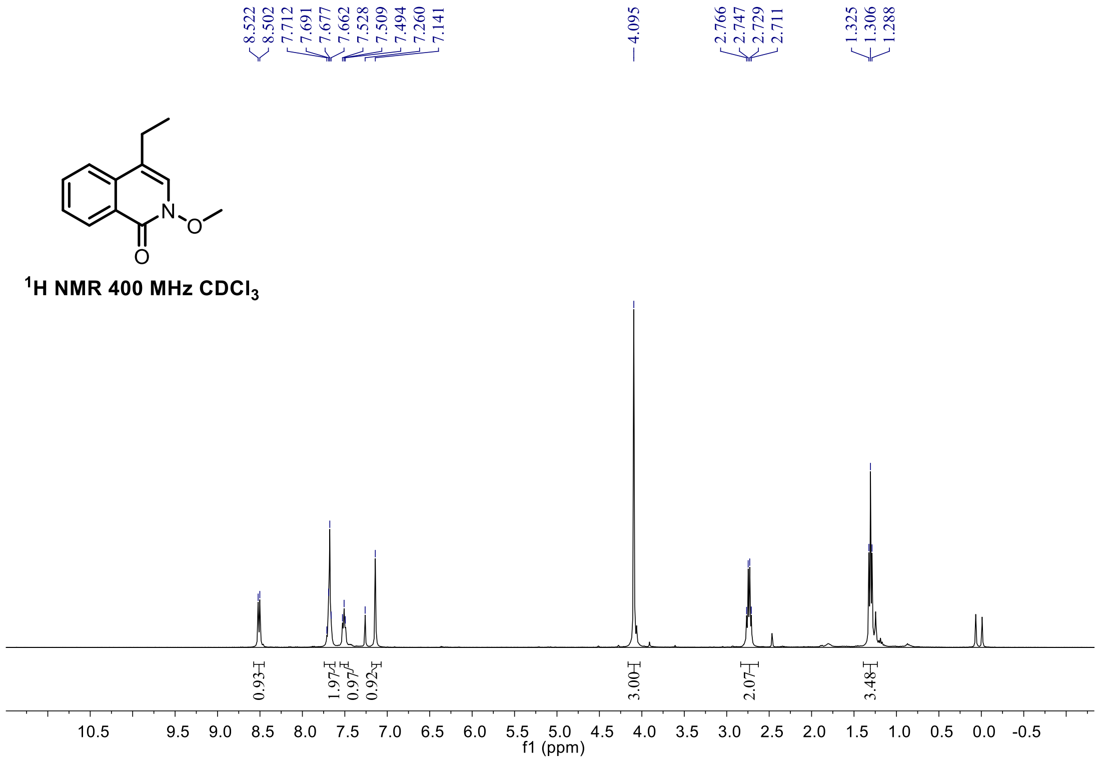
8.522
8.502
7.712
7.691
7.677
7.662
7.528
7.509
7.494
7.260
7.141
4.095
2.766
2.747
2.729
2.711
1.325
1.306
1.288
1H NMR 400 MHz CDCl3
0.93
1.97
0.97
0.92
3.00
2.07
3.48
10.5 9.5 9.0 8.5 8.0 7.5 7.0 6.5 6.0 5.5 5.0 4.5 4.0 3.5 3.0 2.5 2.0 1.5 1.0 0.5 0.0 -0.5
f1 (ppm)

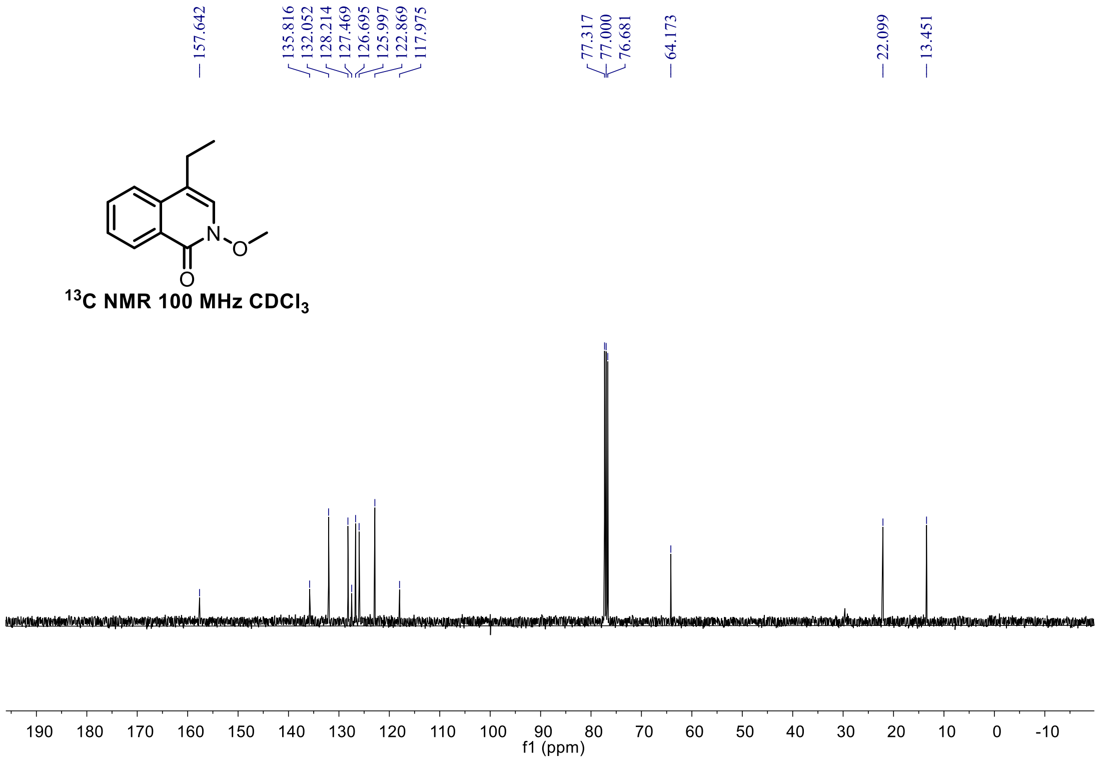
157.642
135.816
132.052
128.214
127.469
126.695
125.997
122.869
117.975
77.317
77.000
76.681
64.173
22.099
13.451
^{13}C NMR 100 MHz CDCl$_3$
f1 (ppm)

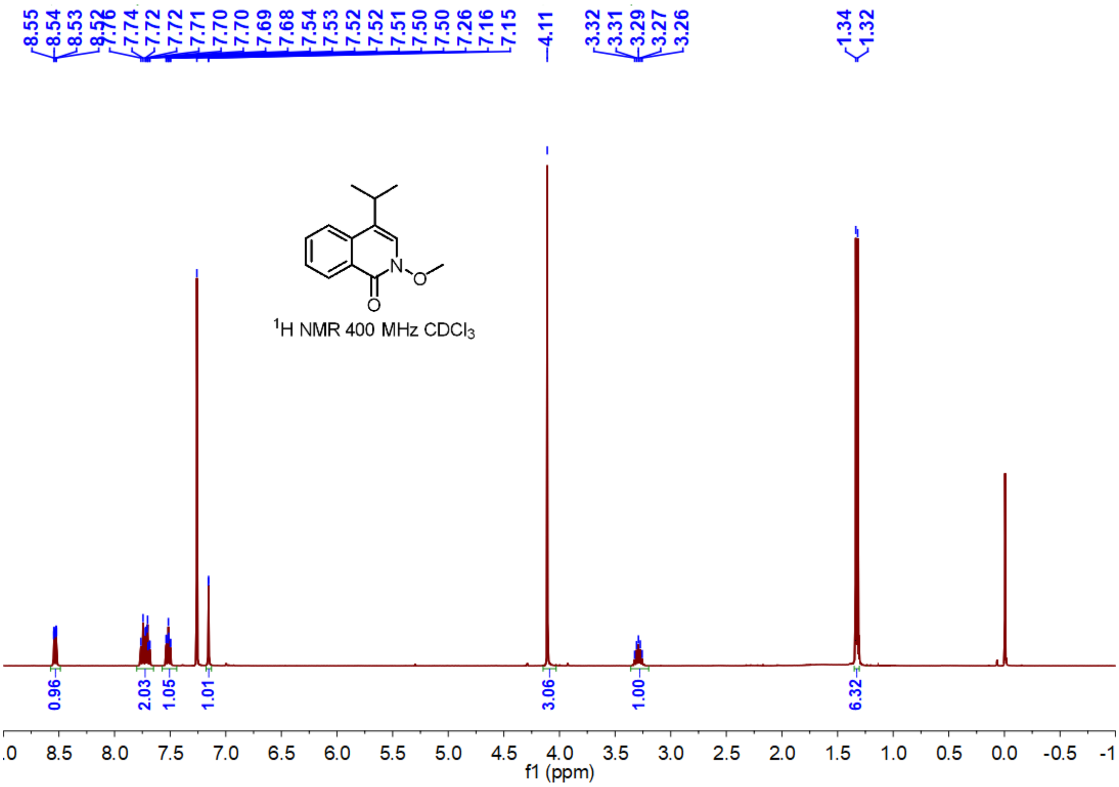
8.55
8.54
8.53
8.52
7.76
7.74
7.72
7.72
7.71
7.70
7.70
7.69
7.68
7.54
7.53
7.52
7.52
7.51
7.50
7.50
7.26
7.16
7.15
4.11
3.32
3.31
3.29
3.27
3.26
1.34
1.32
^{1}H NMR 400 MHz CDCl$_3$
0.96
2.03
1.05
1.01
3.06
1.00
6.32
f1 (ppm)

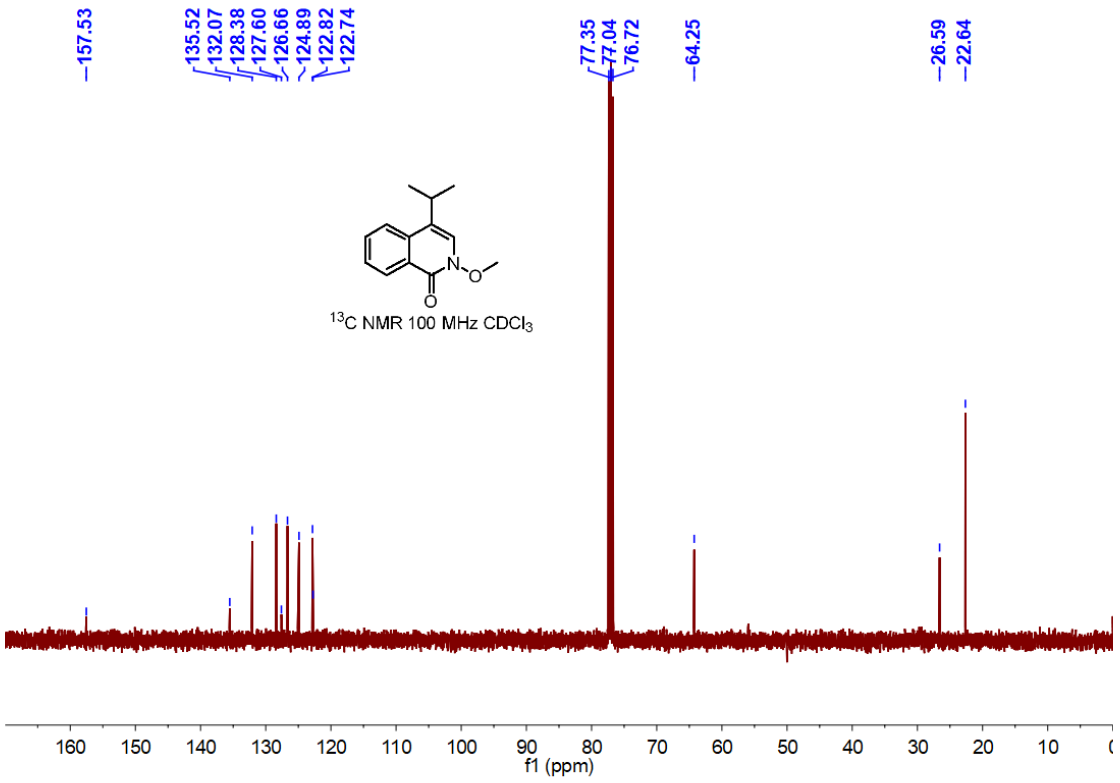
157.53
135.52
132.07
128.38
127.60
126.66
124.89
122.82
122.74
77.35
77.04
76.72
64.25
26.59
22.64
N
O
O
13C NMR 100 MHz CDCl3
160 150 140 130 120 110 100 90 80 70 60 50 40 30 20 10
f1 (ppm)

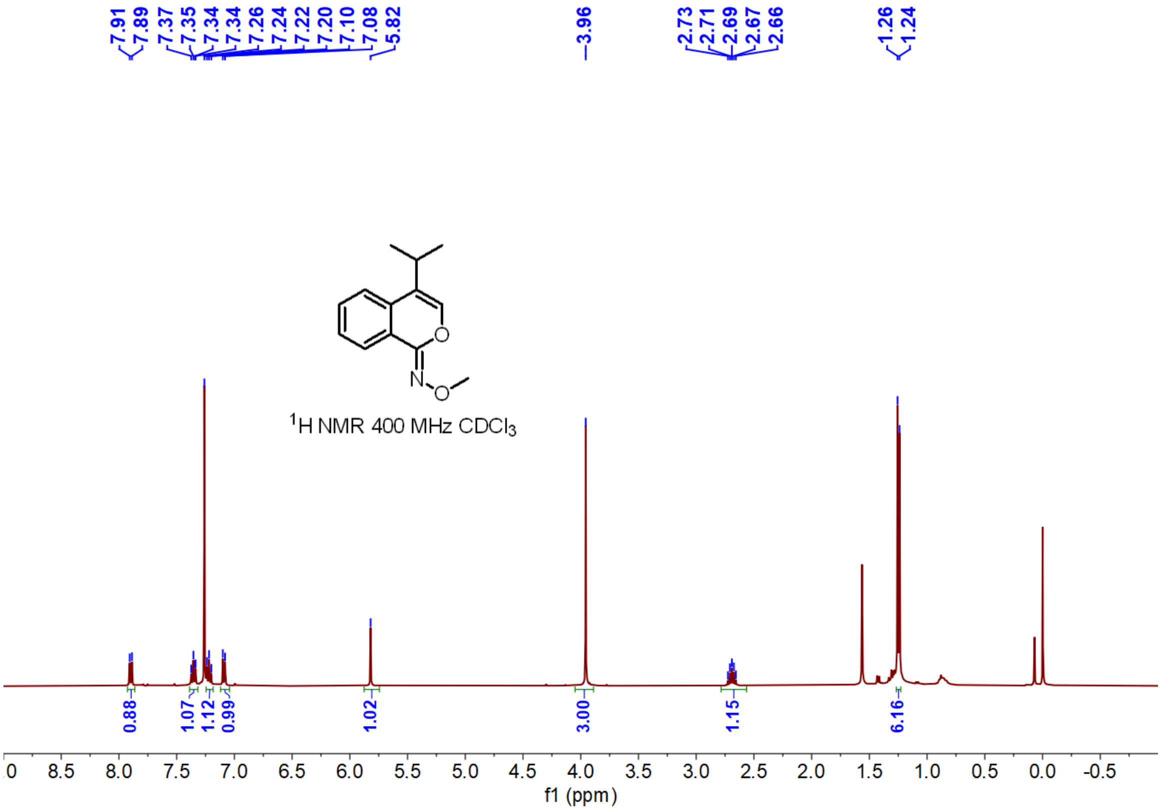
7.91
7.89
7.37
7.35
7.34
7.34
7.26
7.24
7.22
7.20
7.10
7.08
5.82
3.96
2.73
2.71
2.69
2.67
2.66
1.26
1.24
O
N
O
1H NMR 400 MHz CDCl3
0.88
1.07
1.12
0.99
1.02
3.00
1.15
6.16
8.5 8.0 7.5 7.0 6.5 6.0 5.5 5.0 4.5 4.0 3.5 3.0 2.5 2.0 1.5 1.0 0.5 0.0 -0.5
f1 (ppm)

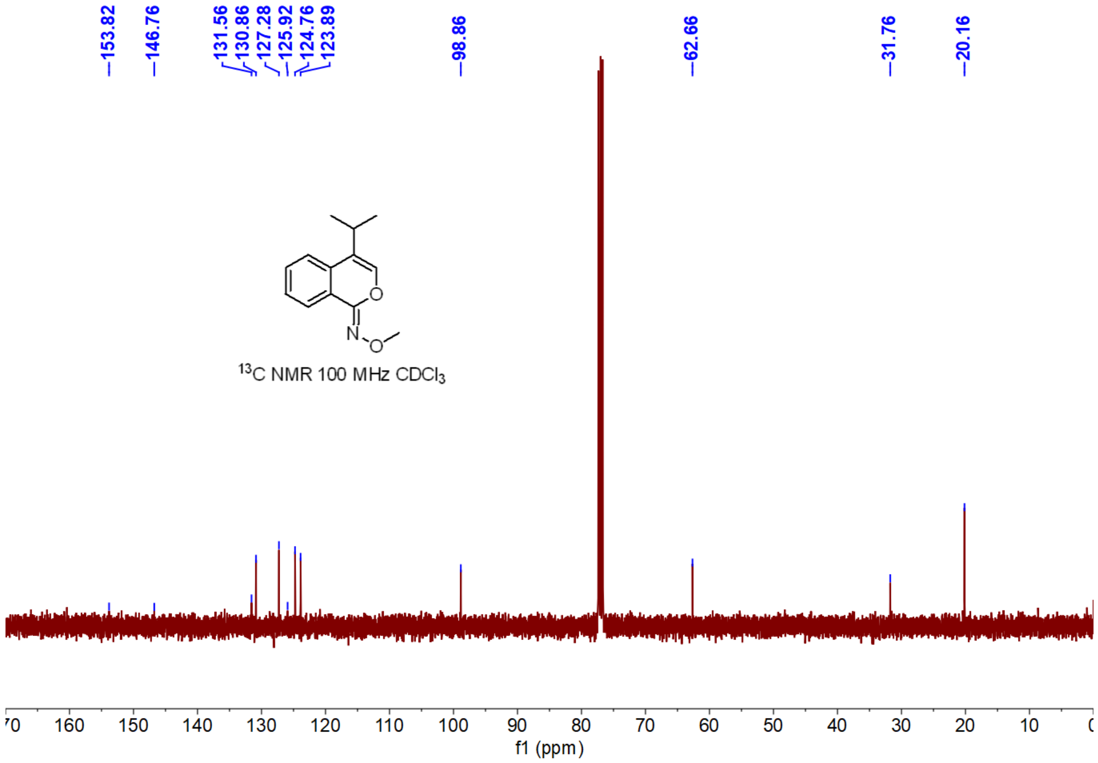

153.82
146.76
131.56
130.86
127.28
125.92
124.76
123.89
98.86
62.66
31.76
20.16
^{13}C NMR 100 MHz CDCl$_3$
f1 (ppm)

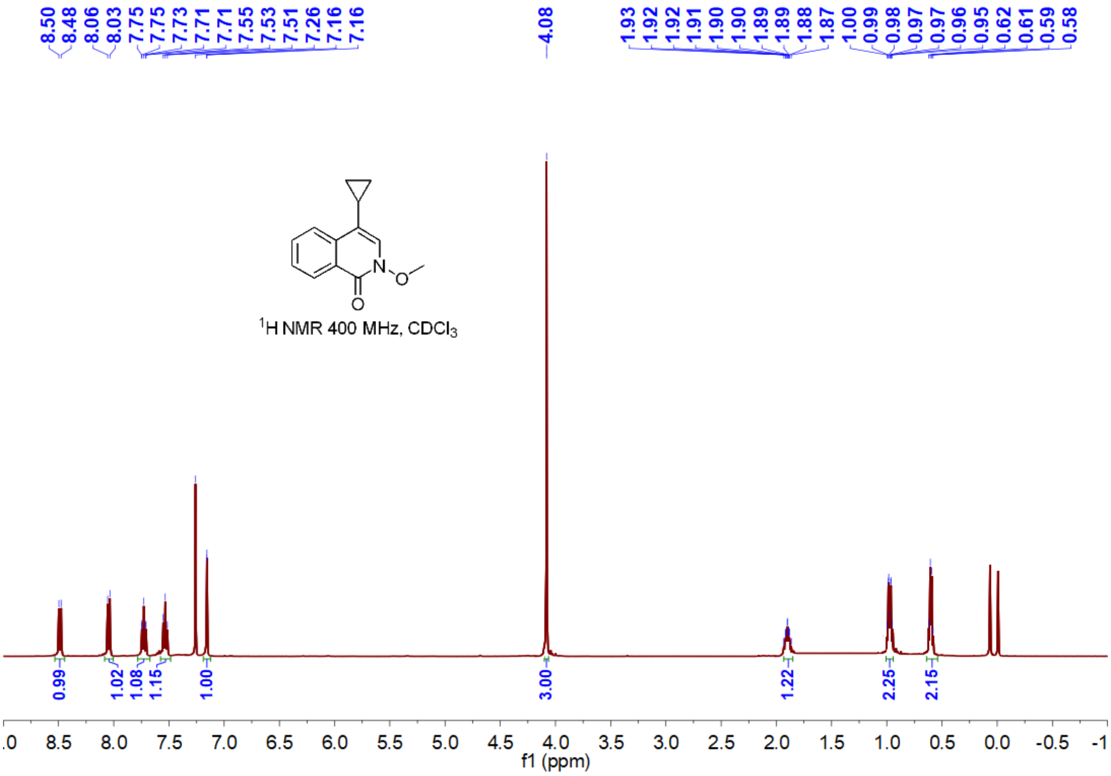

8.50
8.48
8.06
8.03
7.75
7.75
7.73
7.71
7.71
7.55
7.53
7.51
7.26
7.16
7.16
4.08
1.93
1.92
1.92
1.91
1.90
1.90
1.89
1.89
1.88
1.87
1.00
0.99
0.98
0.97
0.97
0.96
0.95
0.62
0.61
0.59
0.58
^{1}H NMR 400 MHz, CDCl$_3$
0.99
1.02
1.08
1.15
1.00
3.00
1.22
2.25
2.15
f1 (ppm)

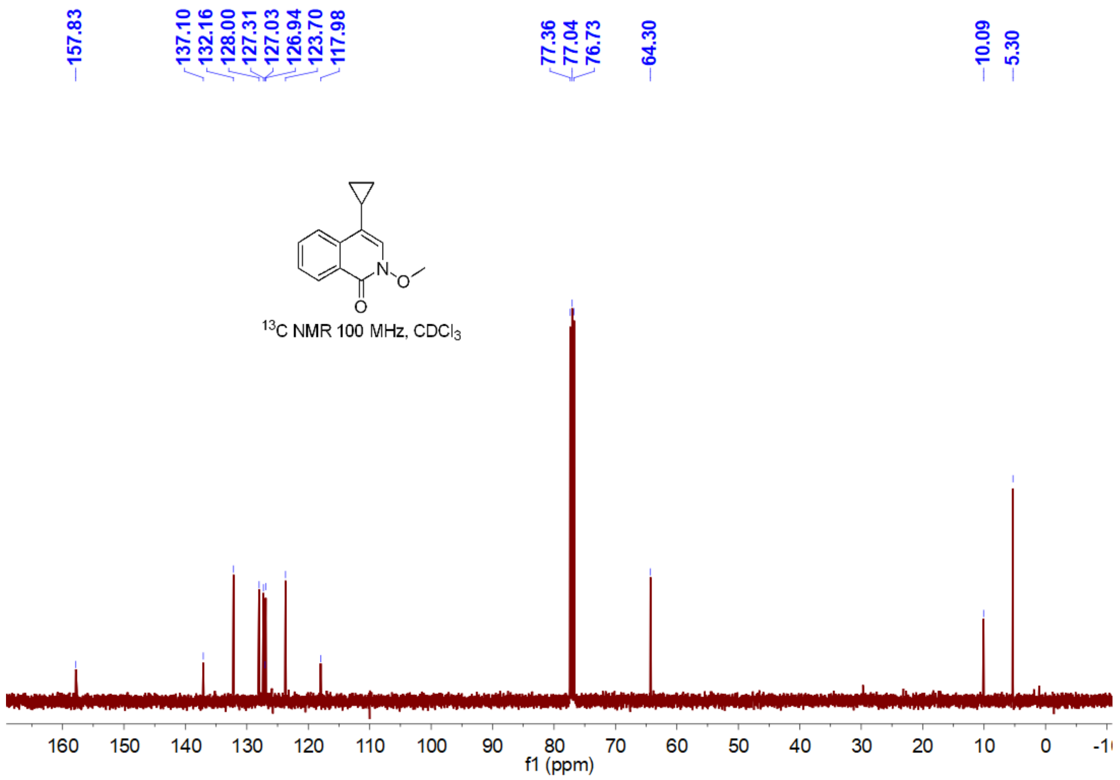

157.83
137.10
132.16
128.00
127.31
127.03
126.94
123.70
117.98
77.36
77.04
76.73
64.30
10.09
5.30
13C NMR 100 MHz, CDCl3
f1 (ppm)

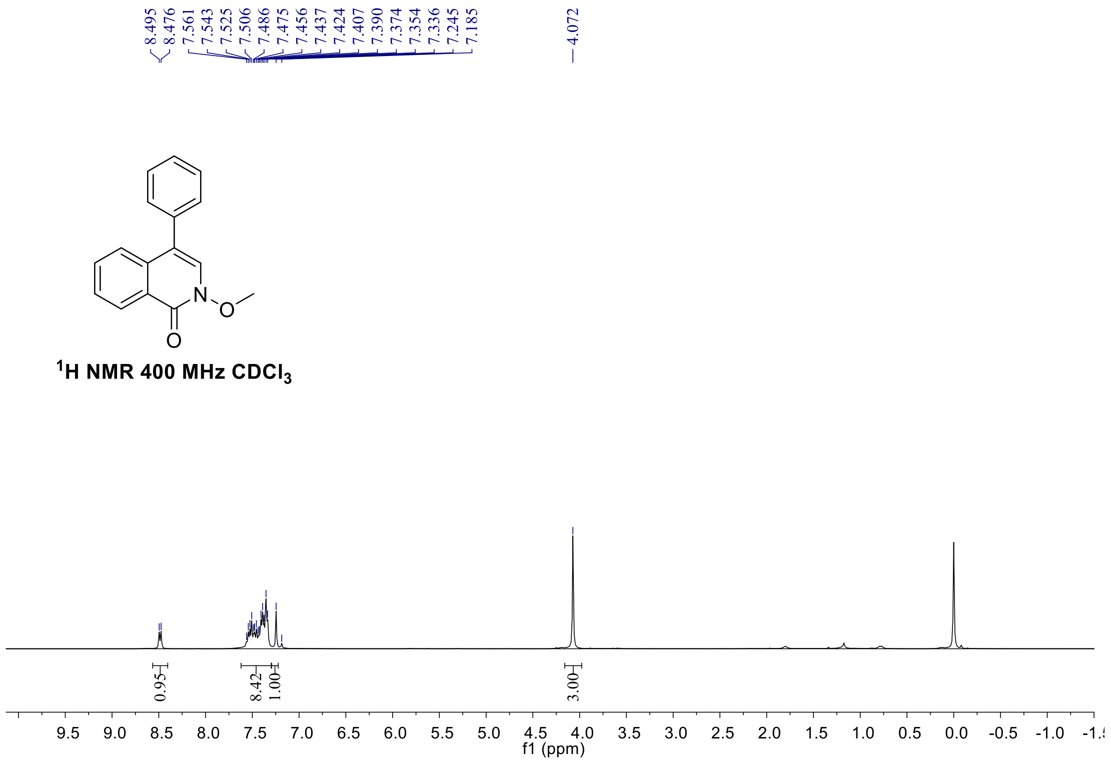

8.495
8.476
7.561
7.543
7.525
7.506
7.486
7.475
7.456
7.437
7.424
7.407
7.390
7.374
7.354
7.336
7.245
7.185
4.072
1H NMR 400 MHz CDCl3
0.95
8.42
1.00
3.00
f1 (ppm)

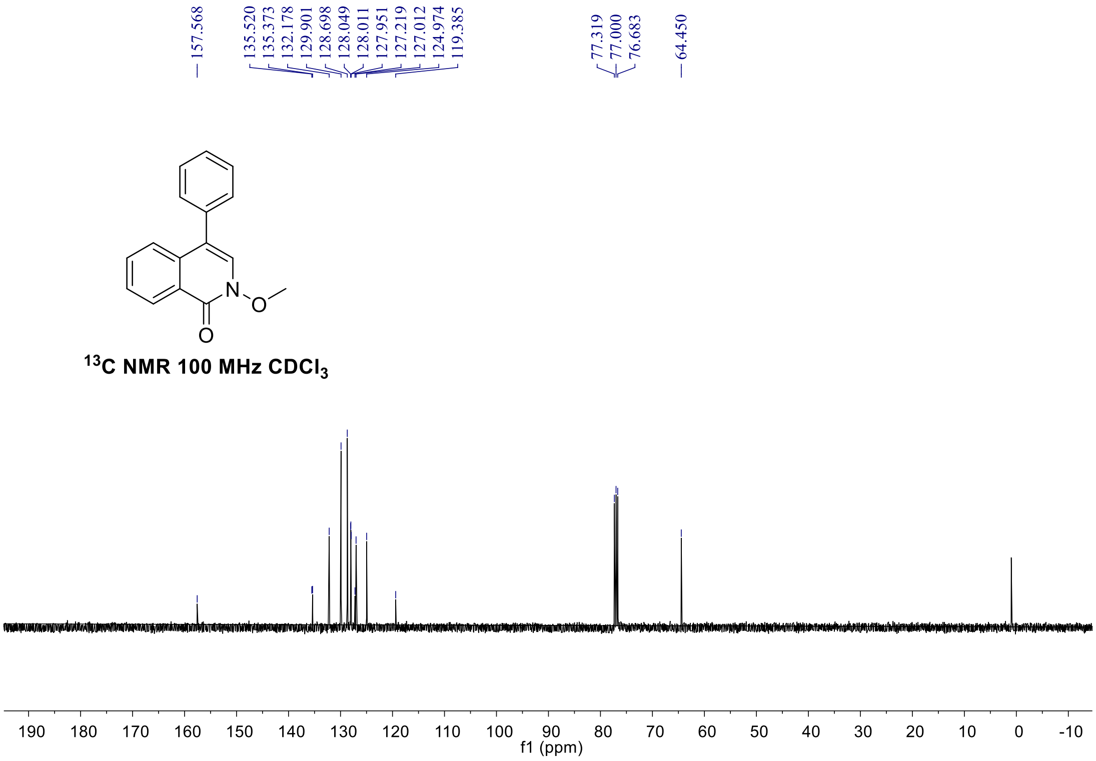
157.568
135.520
135.373
132.178
129.901
128.698
128.049
128.011
127.951
127.219
127.012
124.974
119.385
77.319
77.000
76.683
64.450
N
O
O
13C NMR 100 MHz CDCl3
190 180 170 160 150 140 130 120 110 100 90 80 70 60 50 40 30 20 10 0 -10
f1 (ppm)

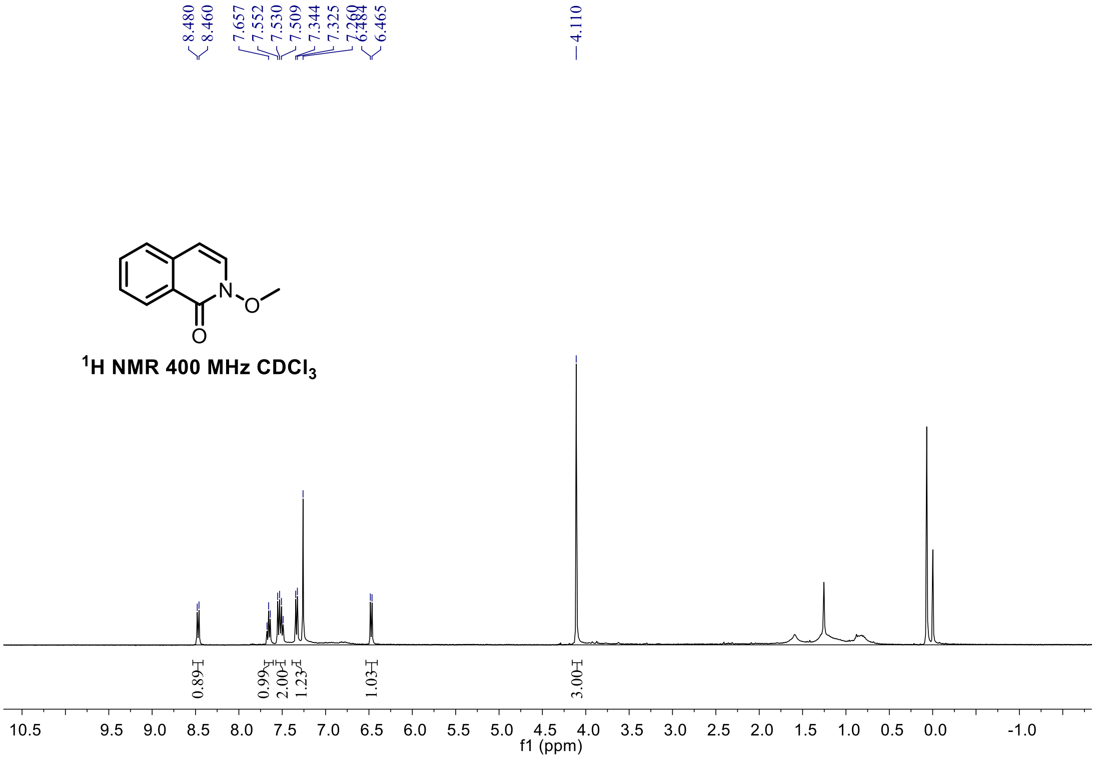
8.480
8.460
7.657
7.552
7.530
7.509
7.344
7.325
7.260
6.484
6.465
4.110
N
O
O
1H NMR 400 MHz CDCl3
0.89
0.99
2.00
1.23
1.03
3.00
10.5 9.5 9.0 8.5 8.0 7.5 7.0 6.5 6.0 5.5 5.0 4.5 4.0 3.5 3.0 2.5 2.0 1.5 1.0 0.5 0.0 -1.0
f1 (ppm)

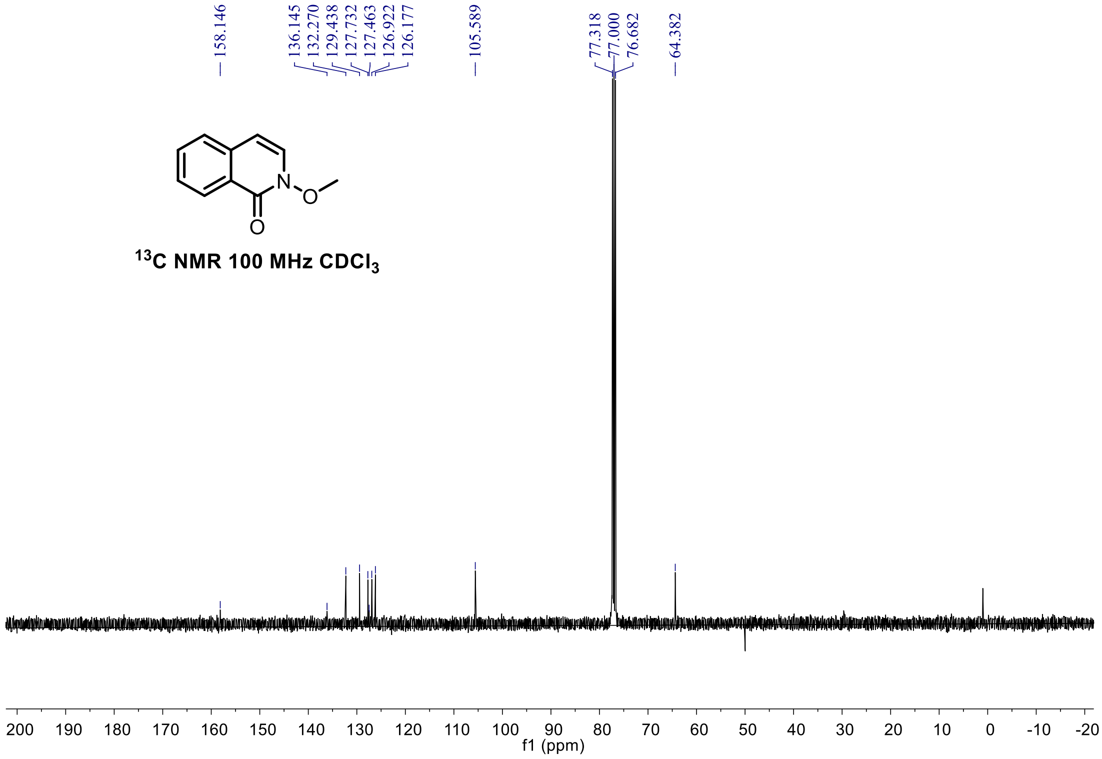
158.146
136.145
132.270
129.438
127.732
127.463
126.922
126.177
105.589
77.318
77.000
76.682
64.382
N
O
O
^{13}C NMR 100 MHz CDCl$_3$
200 190 180 170 160 150 140 130 120 110 100 90 80 70 60 50 40 30 20 10 0 -10 -20
f1 (ppm)

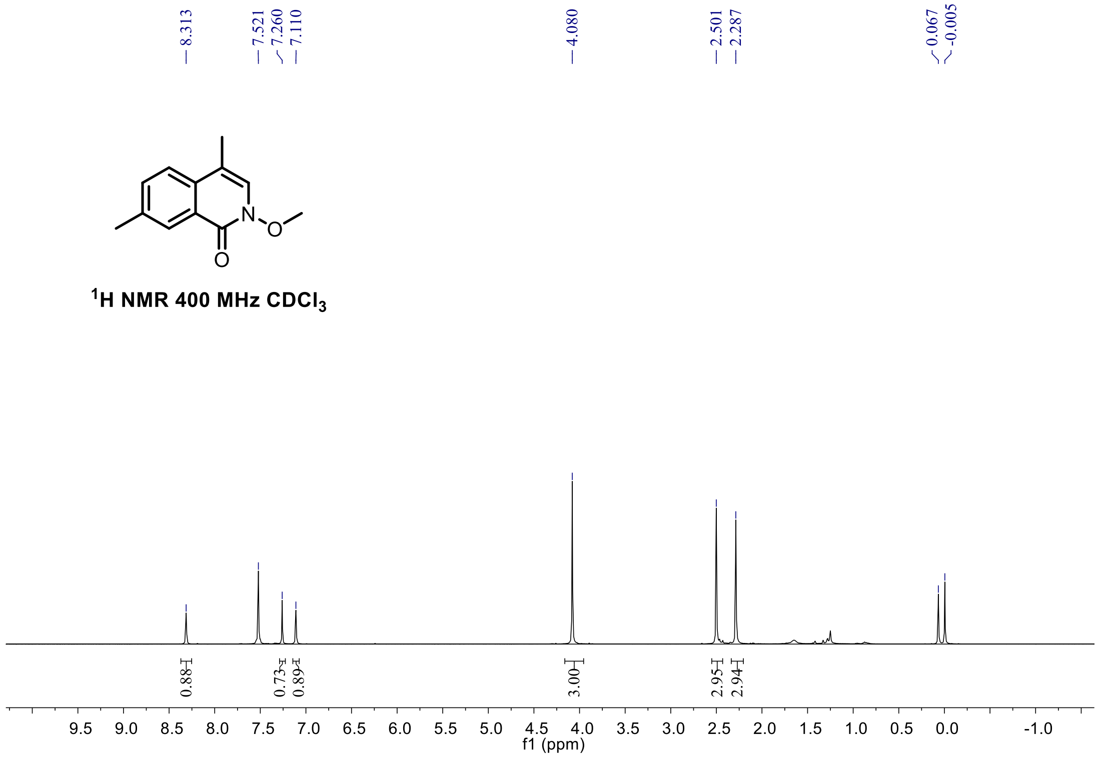
8.313
7.521
7.260
7.110
4.080
2.501
2.287
0.067
-0.005
N
O
O
^{1}H NMR 400 MHz CDCl$_3$
0.88
0.73
0.89
3.00
2.95
2.94
9.5 9.0 8.5 8.0 7.5 7.0 6.5 6.0 5.5 5.0 4.5 4.0 3.5 3.0 2.5 2.0 1.5 1.0 0.5 0.0 -1.0
f1 (ppm)

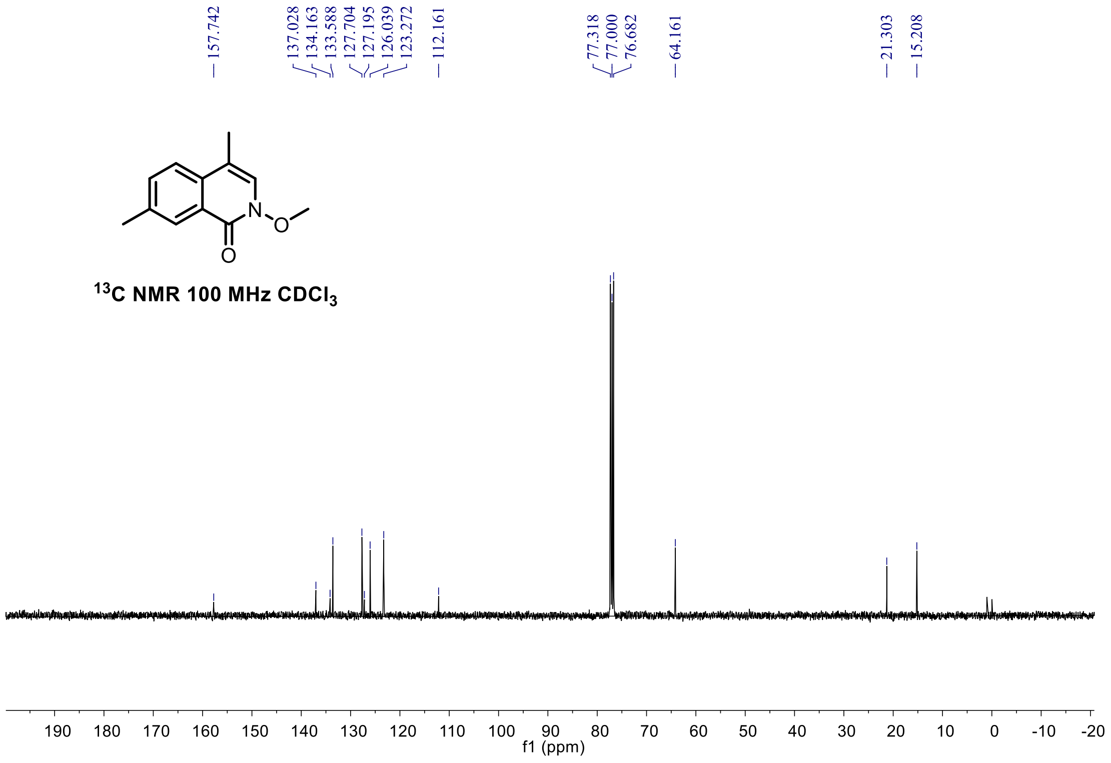
157.742
137.028
134.163
133.588
127.704
127.195
126.039
123.272
112.161
77.318
77.000
76.682
64.161
21.303
15.208
^{13}C NMR 100 MHz CDCl$_3$
190 180 170 160 150 140 130 120 110 100 90 80 70 60 50 40 30 20 10 0 -10 -20
f1 (ppm)

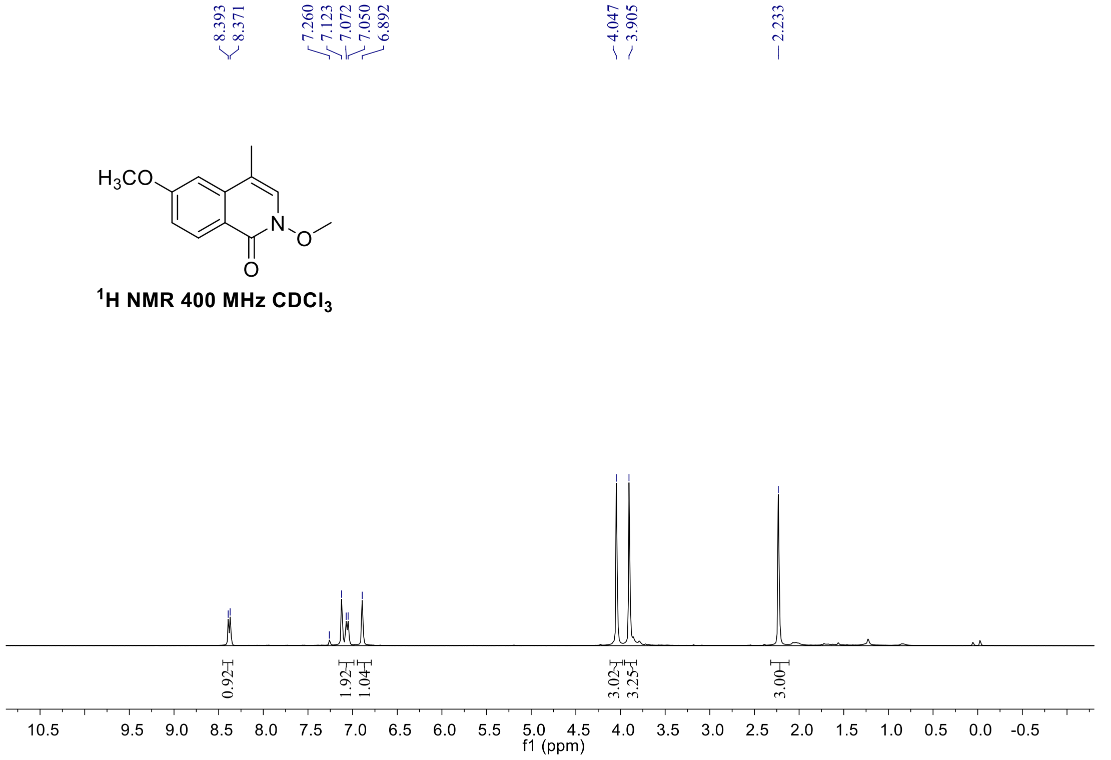
8.393
8.371
7.260
7.123
7.072
7.050
6.892
4.047
3.905
2.233
H$_3$CO
^{1}H NMR 400 MHz CDCl$_3$
0.92
1.92
1.04
3.02
3.25
3.00
10.5 9.5 9.0 8.5 8.0 7.5 7.0 6.5 6.0 5.5 5.0 4.5 4.0 3.5 3.0 2.5 2.0 1.5 1.0 0.5 0.0 -0.5
f1 (ppm)

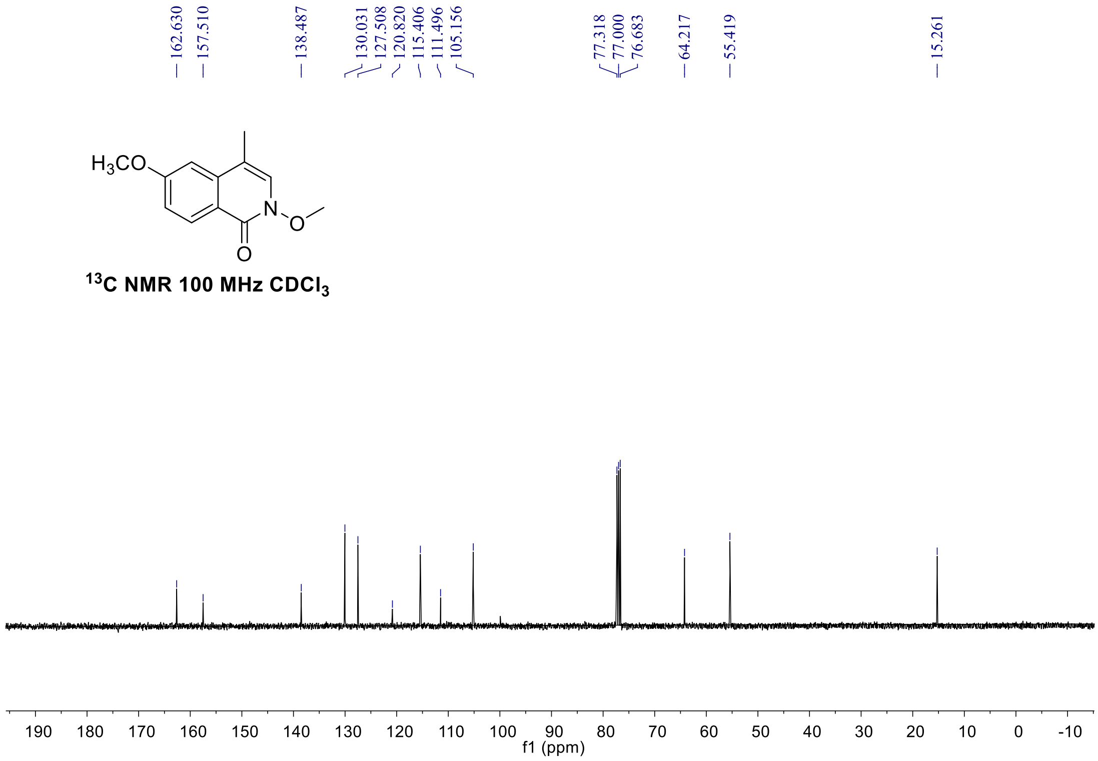
162.630
157.510
138.487
130.031
127.508
120.820
115.406
111.496
105.156
77.318
77.000
76.683
64.217
55.419
15.261
H3CO
N
O
O
^{13}C NMR 100 MHz $CDCl_3$
f1 (ppm)

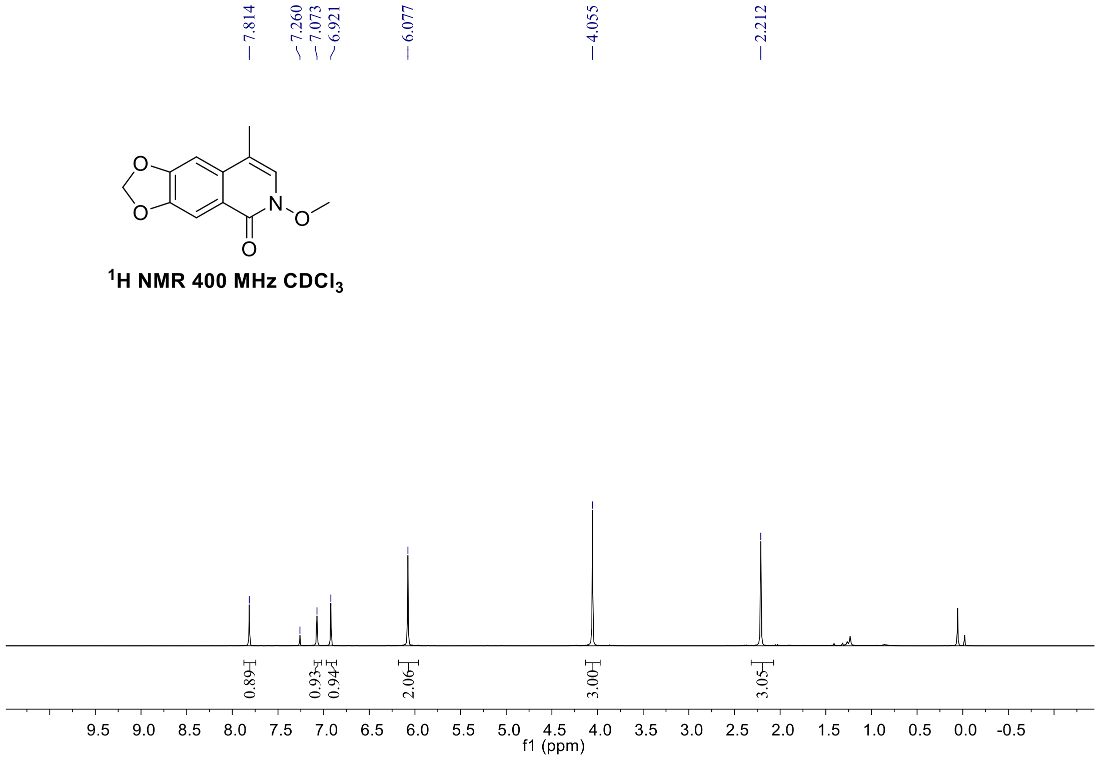
7.814
7.260
7.073
6.921
6.077
4.055
2.212
O
O
N
O
O
^{1}H NMR 400 MHz $CDCl_3$
0.89
0.93
0.94
2.06
3.00
3.05
f1 (ppm)

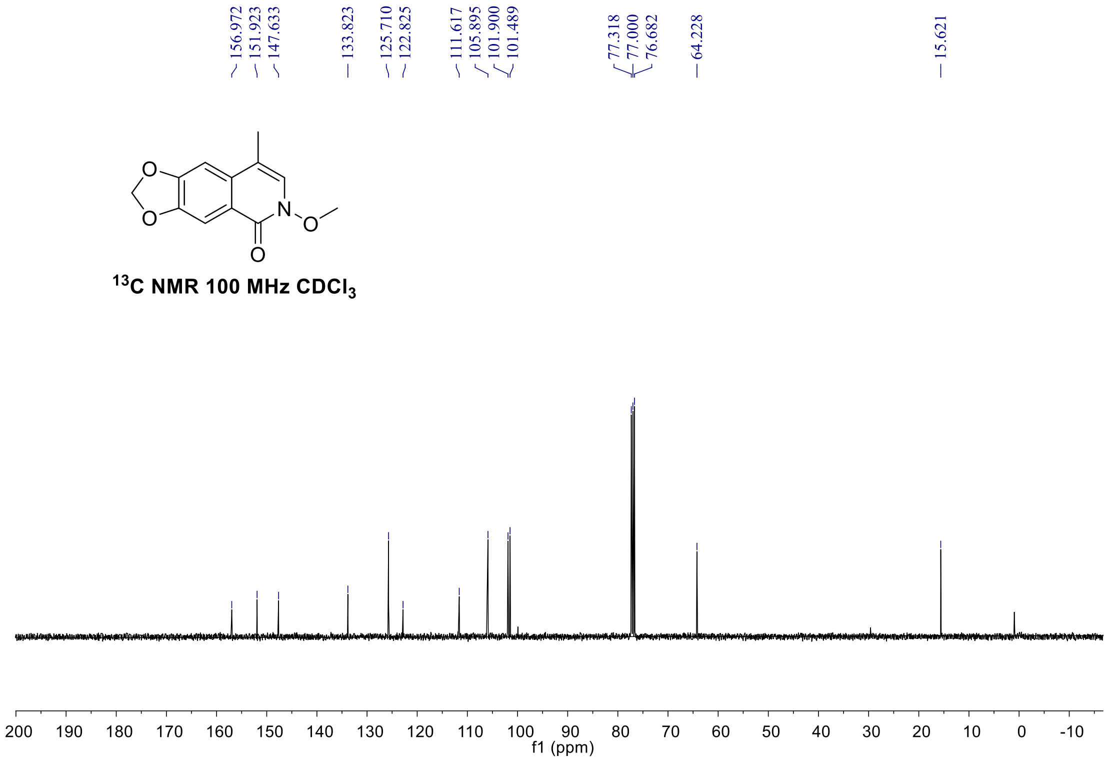
156.972
151.923
147.633
133.823
125.710
122.825
111.617
105.895
101.900
101.489
77.318
77.000
76.682
64.228
15.621
N
O
O
O
O
^{13}C NMR 100 MHz CDCl$_3$
f1 (ppm)

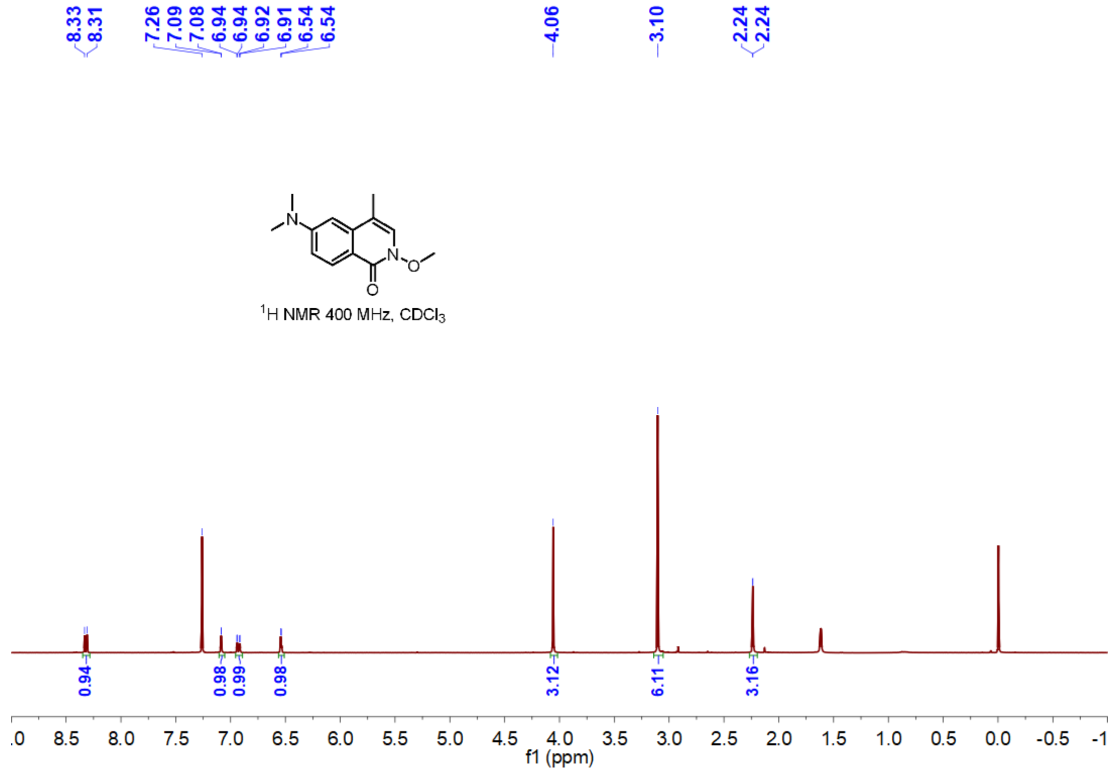
8.33
8.31
7.26
7.09
7.08
6.94
6.94
6.92
6.91
6.54
6.54
4.06
3.10
2.24
2.24
N
N
O
O
^{1}H NMR 400 MHz, CDCl$_3$
0.94
0.98
0.99
0.98
3.12
6.11
3.16
f1 (ppm)

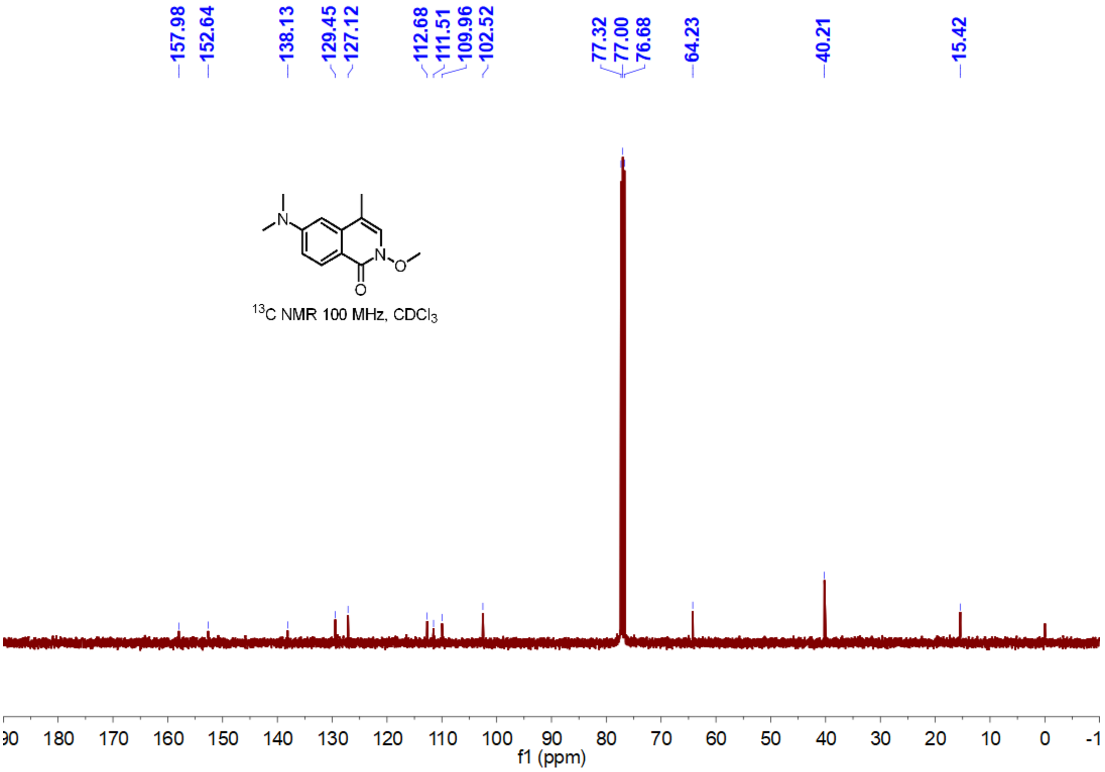
157.98
152.64
138.13
129.45
127.12
112.68
111.51
109.96
102.52
77.32
77.00
76.68
64.23
40.21
15.42
N
N
O
O
13C NMR 100 MHz, CDCl3
f1 (ppm)

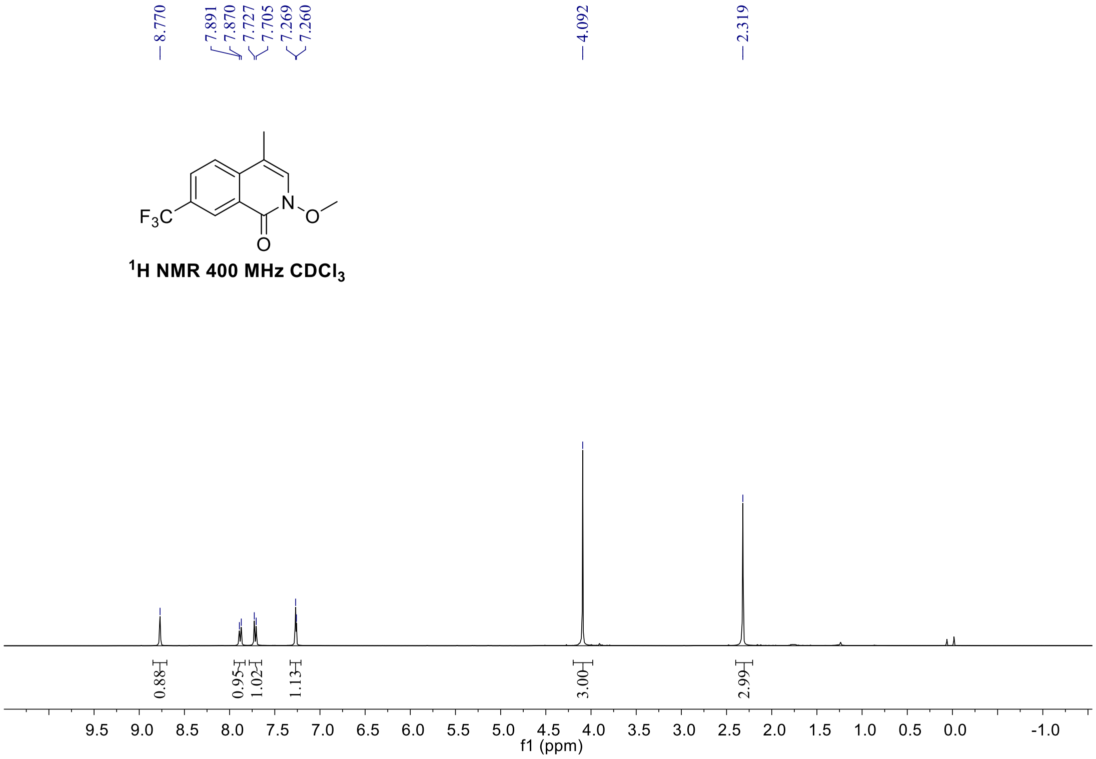
8.770
7.891
7.870
7.727
7.705
7.269
7.260
4.092
2.319
F3C
N
O
O
1H NMR 400 MHz CDCl3
0.88
0.95
1.02
1.13
3.00
2.99
f1 (ppm)

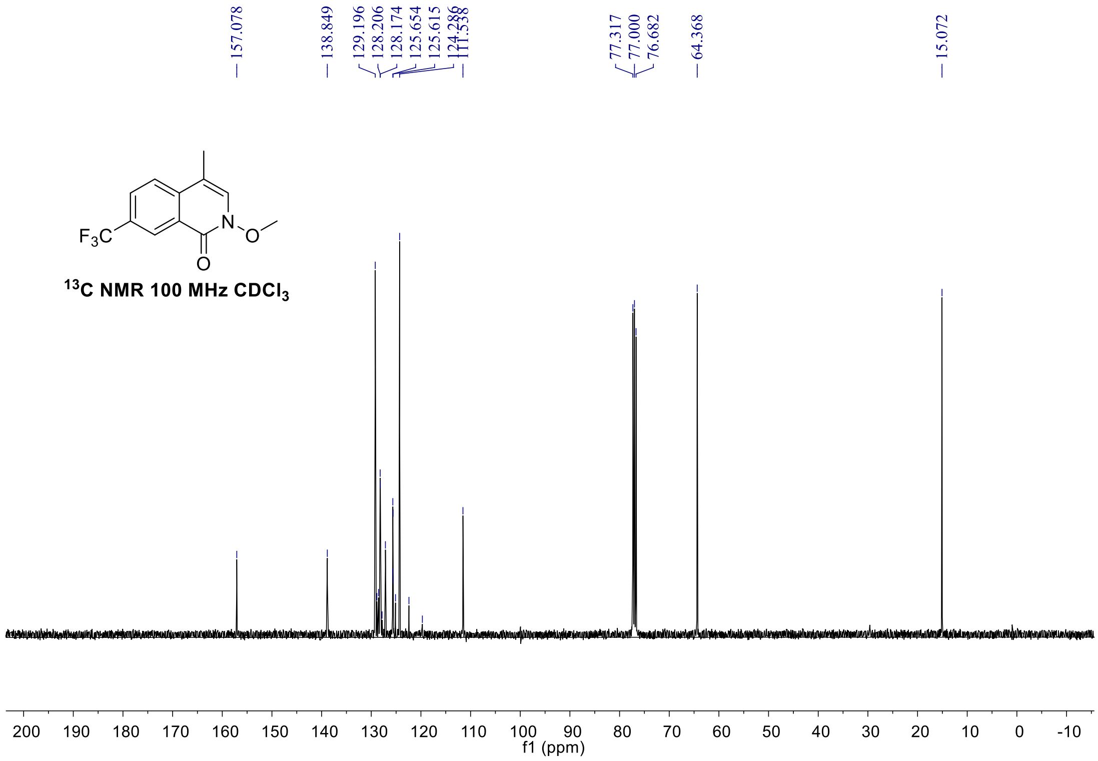
157.078
138.849
129.196
128.206
128.174
125.654
125.615
124.286
111.538
77.317
77.000
76.682
64.368
15.072
F3C
N
O
O
13C NMR 100 MHz CDCl3
200 190 180 170 160 150 140 130 120 110 100 90 80 70 60 50 40 30 20 10 0 -10
f1 (ppm)

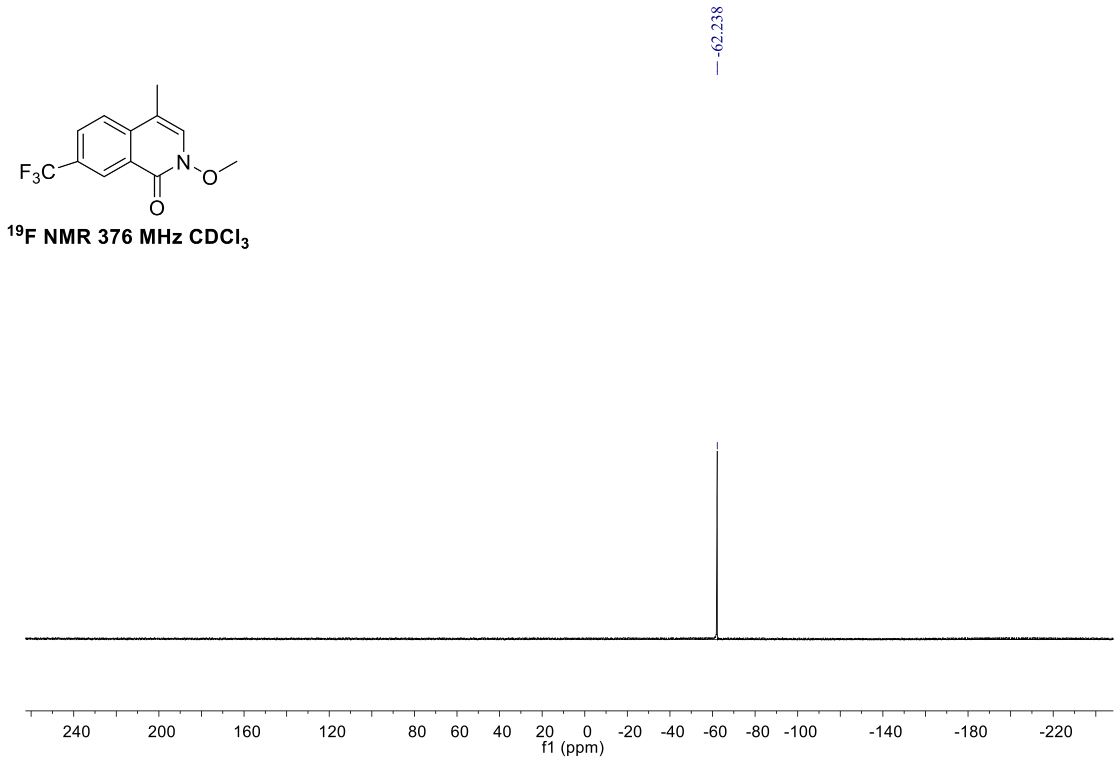
-62.238
F3C
N
O
O
19F NMR 376 MHz CDCl3
240 200 160 120 80 60 40 20 0 -20 -40 -60 -80 -100 -140 -180 -220
f1 (ppm)

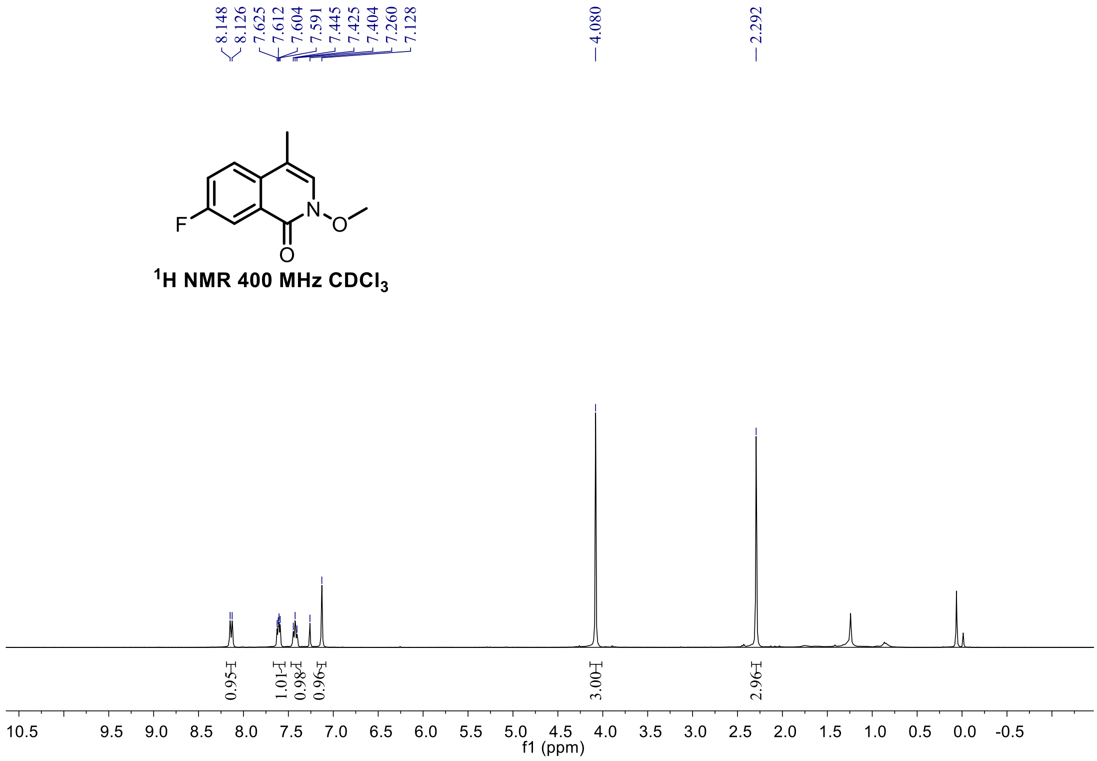
8.148
8.126
7.625
7.612
7.604
7.591
7.445
7.425
7.404
7.260
7.128
4.080
2.292
F
N
O
O
1H NMR 400 MHz CDCl3
0.95
1.01
0.98
0.96
3.00
2.96
10.5 9.5 9.0 8.5 8.0 7.5 7.0 6.5 6.0 5.5 5.0 4.5 4.0 3.5 3.0 2.5 2.0 1.5 1.0 0.5 0.0 -0.5
f1 (ppm)

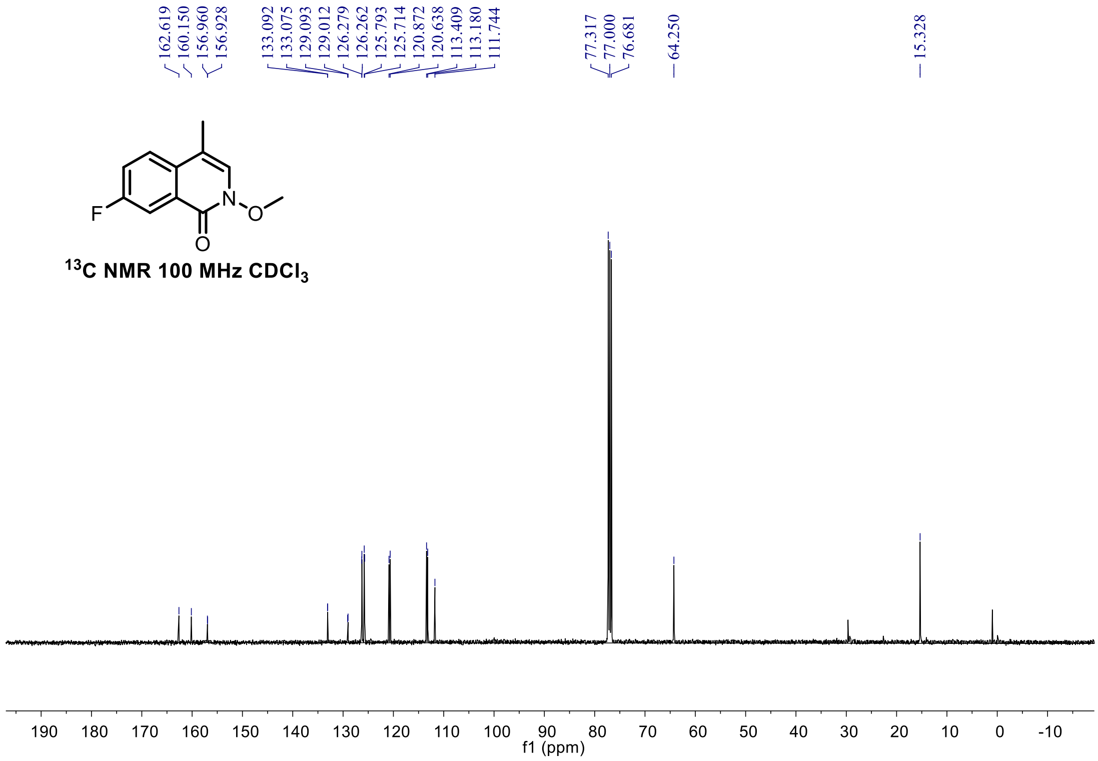
162.619
160.150
156.960
156.928
133.092
133.075
129.093
129.012
126.279
126.262
125.793
125.714
120.872
120.638
113.409
113.180
111.744
77.317
77.000
76.681
64.250
15.328
F
N
O
O
13C NMR 100 MHz CDCl3
190 180 170 160 150 140 130 120 110 100 90 80 70 60 50 40 30 20 10 0 -10
f1 (ppm)

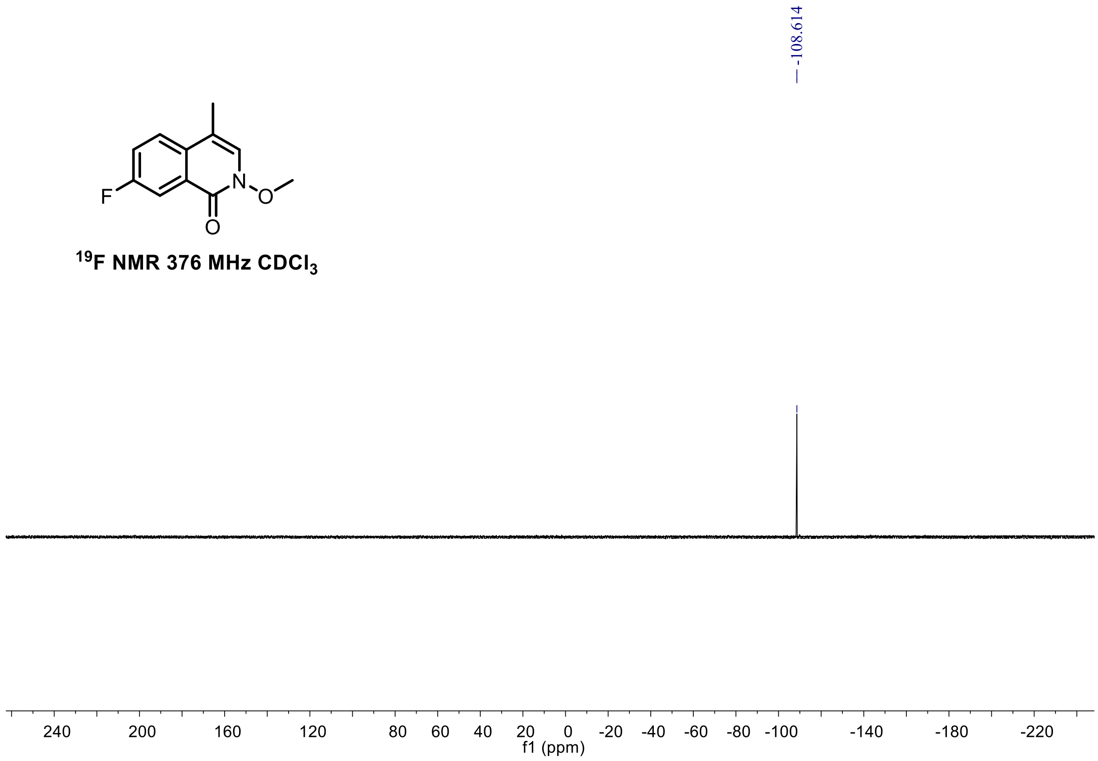
-108.614
F
N
O
O
19F NMR 376 MHz CDCl3
240 200 160 120 80 60 40 20 0 -20 -40 -60 -80 -100 -140 -180 -220
f1 (ppm)

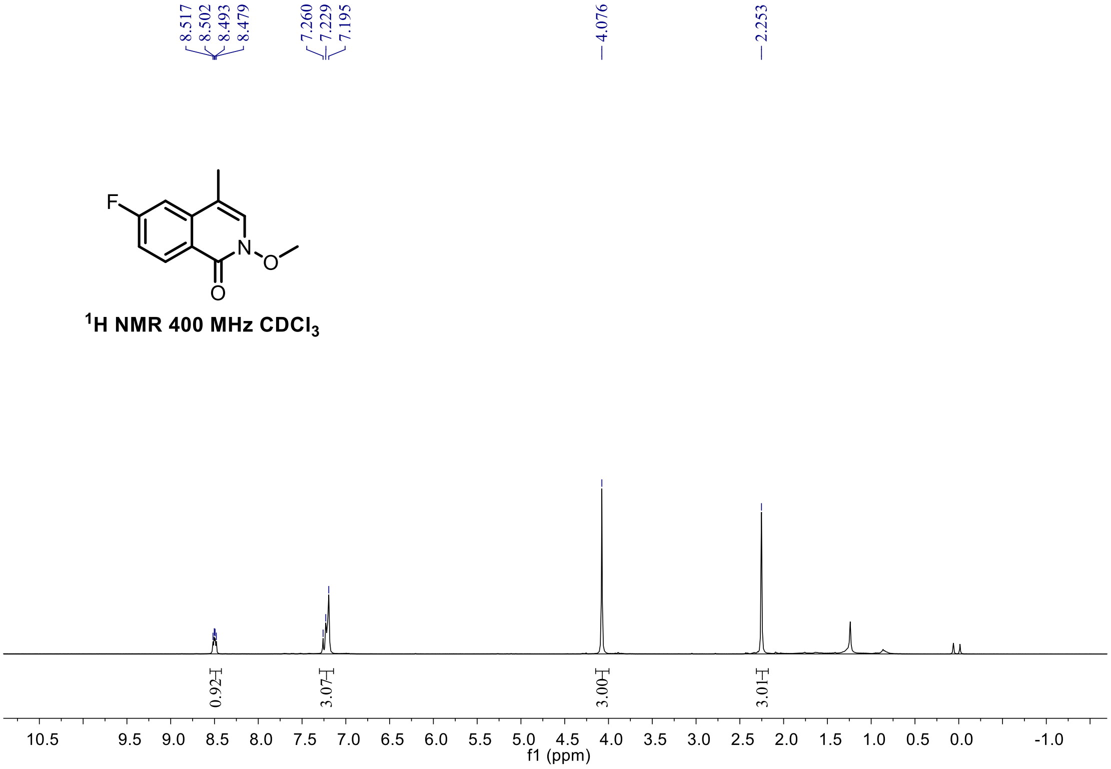
8.517
8.502
8.493
8.479
7.260
7.229
7.195
4.076
2.253
F
N
O
O
1H NMR 400 MHz CDCl3
0.92
3.07
3.00
3.01
10.5 9.5 9.0 8.5 8.0 7.5 7.0 6.5 6.0 5.5 5.0 4.5 4.0 3.5 3.0 2.5 2.0 1.5 1.0 0.5 0.0 -1.0
f1 (ppm)

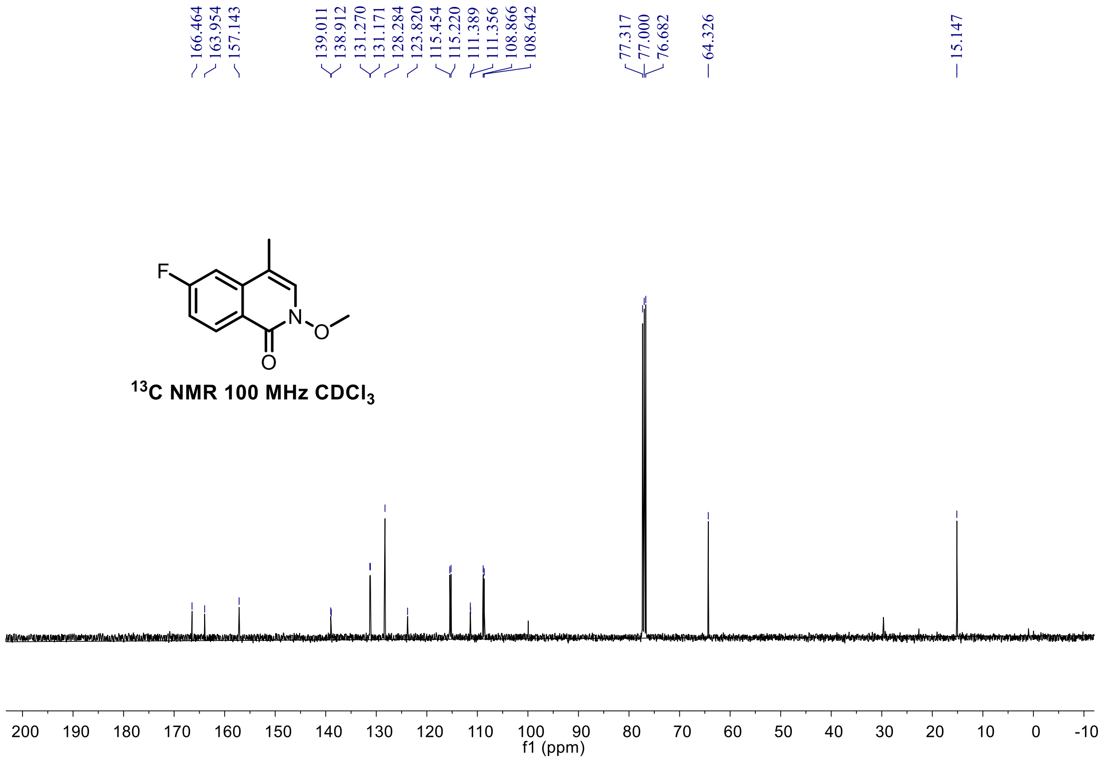
166.464
163.954
157.143
139.011
138.912
131.270
131.171
128.284
123.820
115.454
115.220
111.389
111.356
108.866
108.642
77.317
77.000
76.682
64.326
15.147
F
N
O
O
13C NMR 100 MHz CDCl3
200 190 180 170 160 150 140 130 120 110 100 90 80 70 60 50 40 30 20 10 0 -10
f1 (ppm)

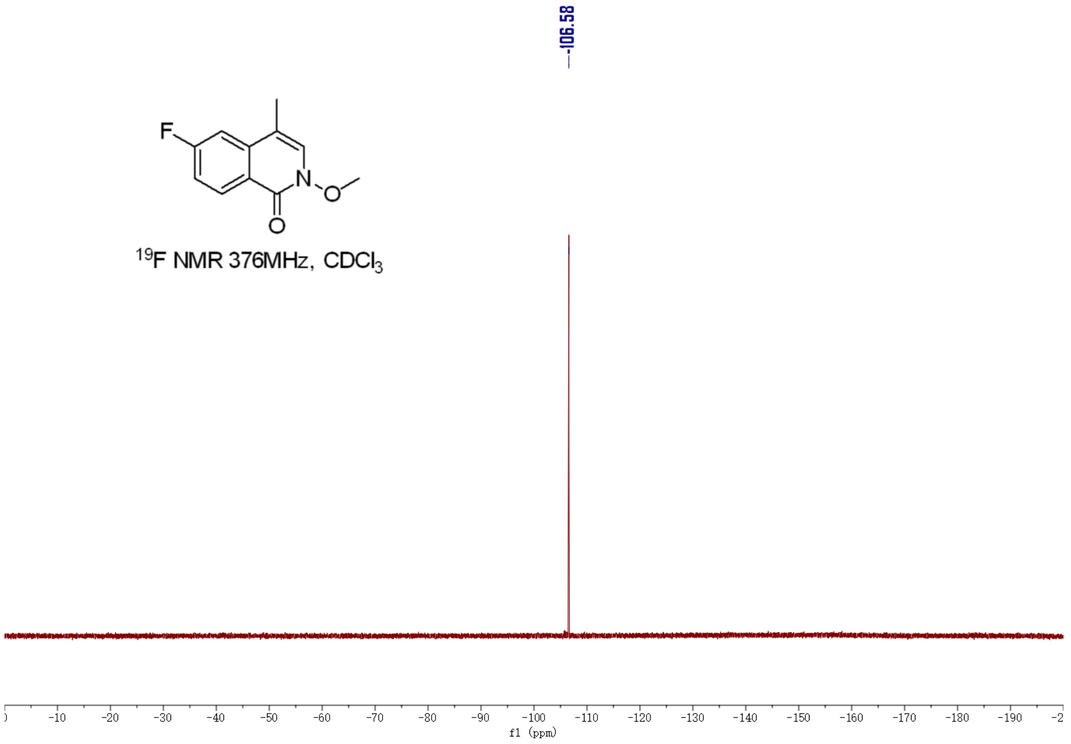
-106.58
F
N
O
O
19F NMR 376MHz, CDCl3
-10 -20 -30 -40 -50 -60 -70 -80 -90 -100 -110 -120 -130 -140 -150 -160 -170 -180 -190
f1 (ppm)

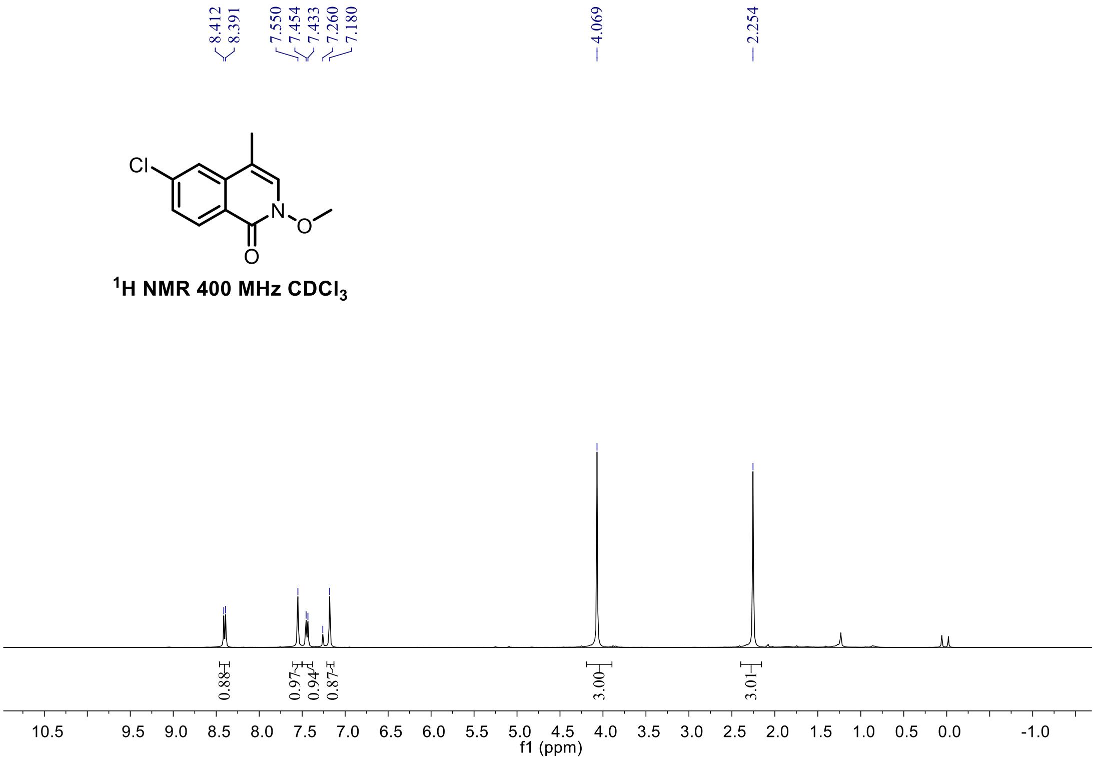
8.412
8.391
7.550
7.454
7.433
7.260
7.180
4.069
2.254
Cl
N
O
O
1H NMR 400 MHz CDCl3
0.88
0.97
0.94
0.87
3.00
3.01
10.5 9.5 9.0 8.5 8.0 7.5 7.0 6.5 6.0 5.5 5.0 4.5 4.0 3.5 3.0 2.5 2.0 1.5 1.0 0.5 0.0 -1.0
f1 (ppm)

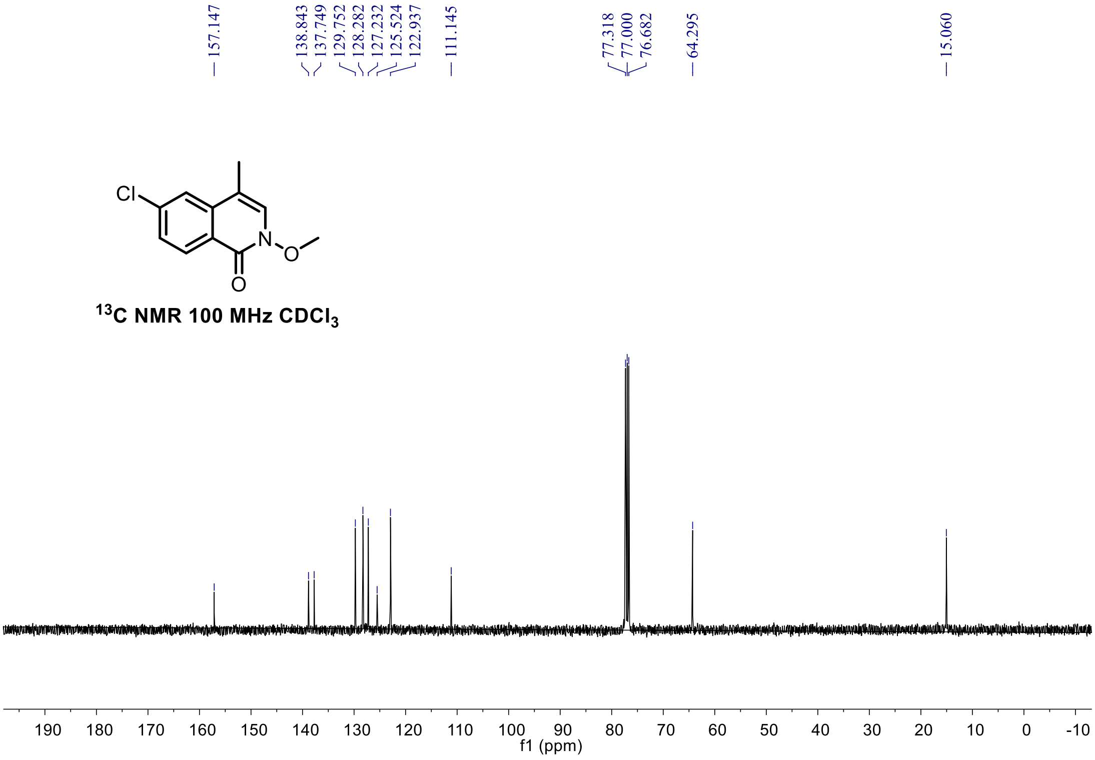
157.147
138.843
137.749
129.752
128.282
127.232
125.524
122.937
111.145
77.318
77.000
76.682
64.295
15.060
Cl
N
O
O
13C NMR 100 MHz CDCl3
190 180 170 160 150 140 130 120 110 100 90 80 70 60 50 40 30 20 10 0 -10
f1 (ppm)

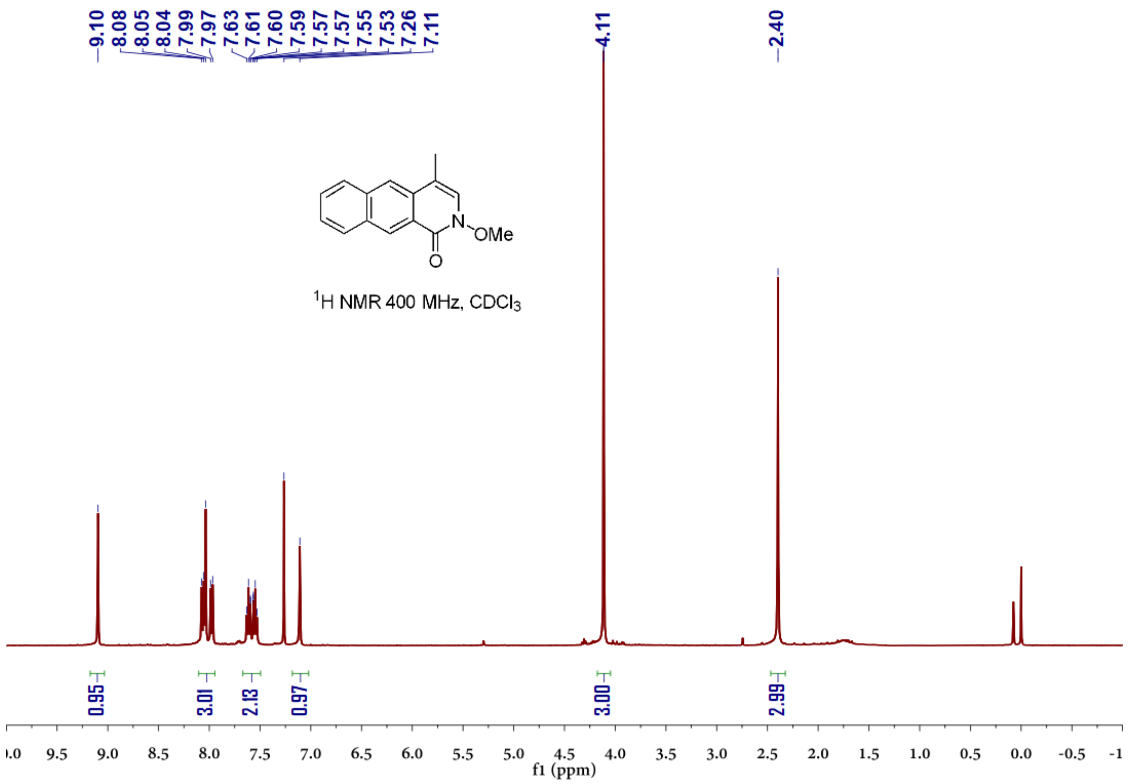
9.10
8.08
8.05
8.04
7.99
7.97
7.63
7.61
7.60
7.59
7.57
7.57
7.55
7.53
7.26
7.11
4.11
2.40
N
OMe
O
^{1}H NMR 400 MHz, CDCl$_3$
0.95
3.01
2.13
0.97
3.00
2.99
f1 (ppm)

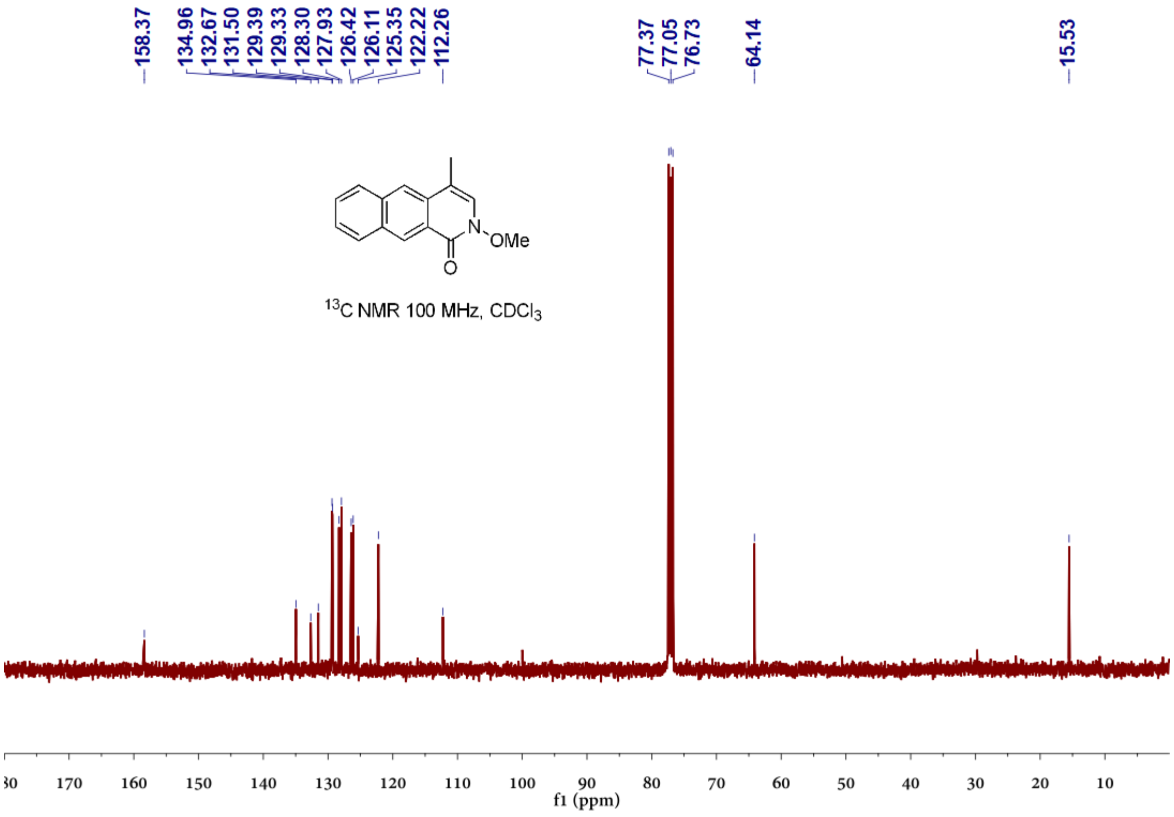
158.37
134.96
132.67
131.50
129.39
129.33
128.30
127.93
126.42
126.11
125.35
122.22
112.26
77.37
77.05
76.73
64.14
15.53
N
OMe
O
^{13}C NMR 100 MHz, CDCl$_3$
f1 (ppm)

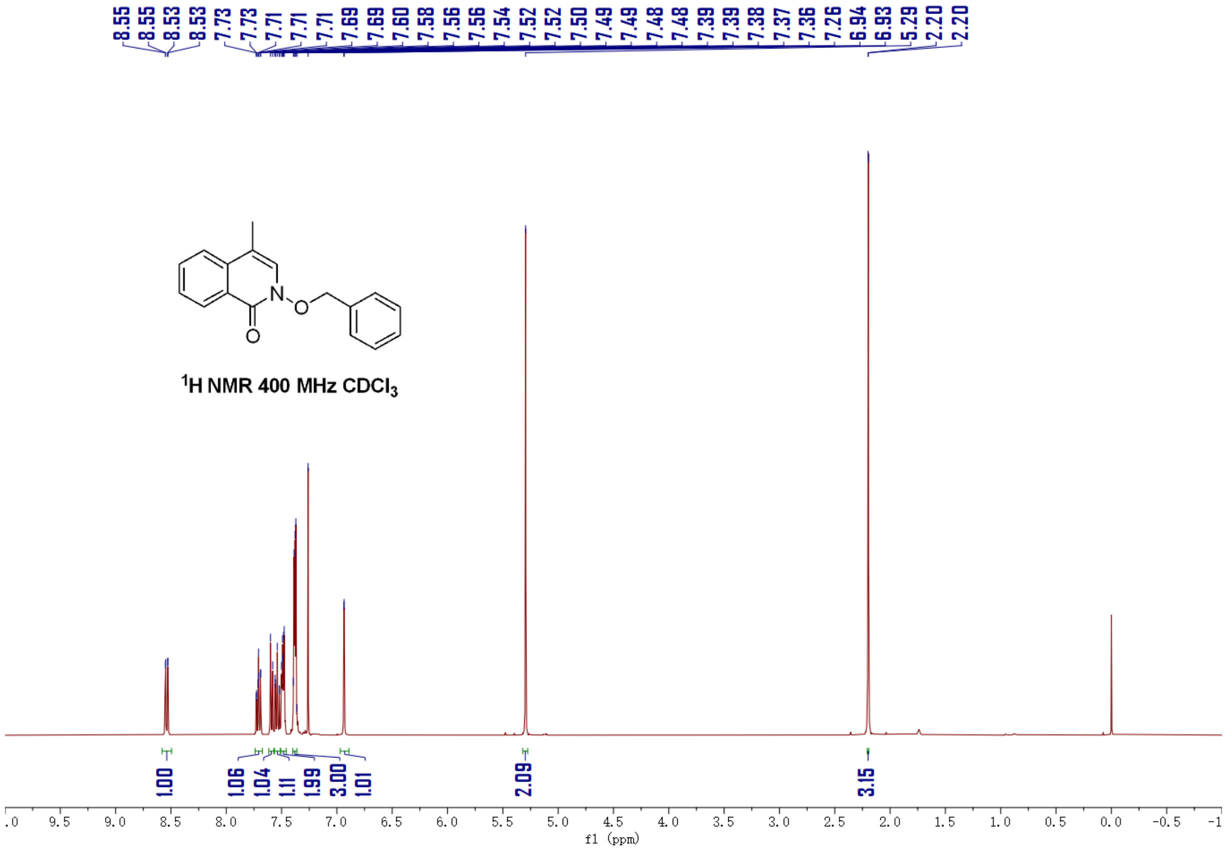

8.55
8.55
8.53
8.53
7.73
7.73
7.71
7.71
7.71
7.69
7.69
7.60
7.58
7.56
7.56
7.54
7.52
7.52
7.50
7.49
7.49
7.48
7.48
7.39
7.39
7.38
7.37
7.36
7.26
6.94
6.93
5.29
2.20
2.20
N
O
O
^{1}H NMR 400 MHz CDCl$_3$
1.00
1.06
1.04
1.11
1.99
3.00
1.01
2.09
3.15
f1 (ppm)

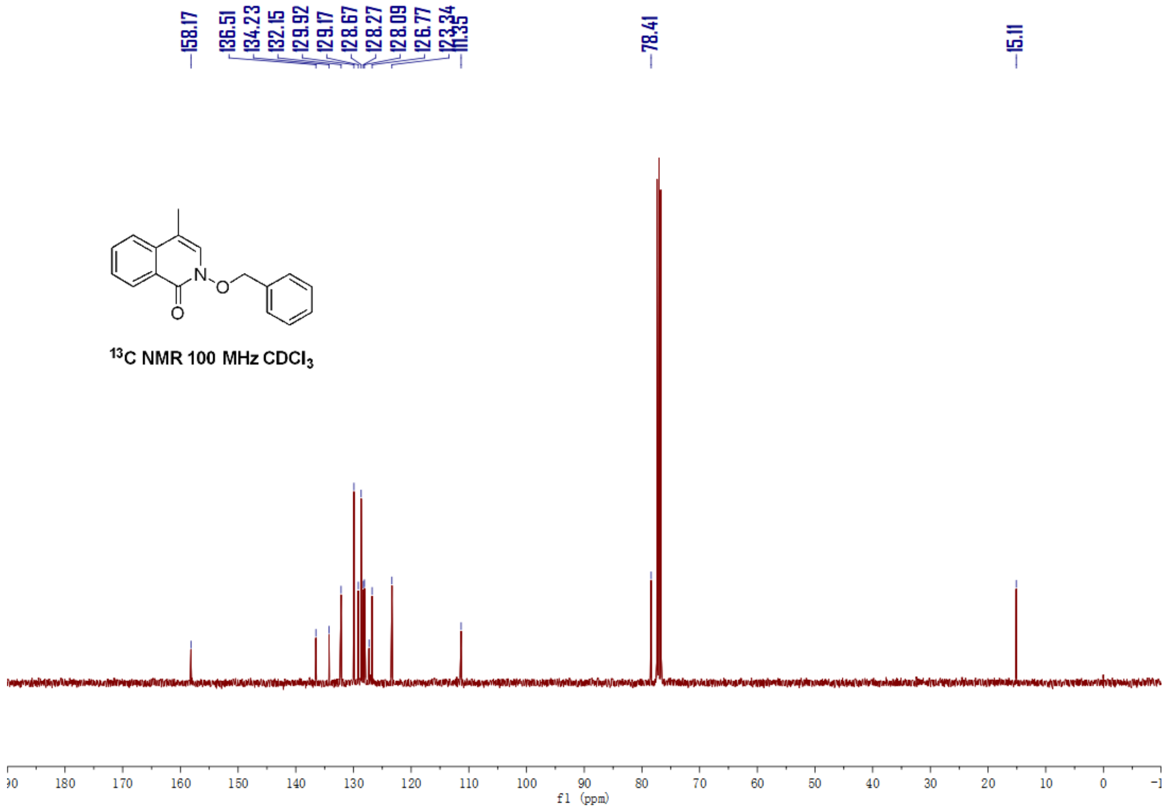

158.17
136.51
134.23
132.15
129.92
129.17
128.67
128.27
128.09
126.77
123.34
111.35
78.41
15.11
N
O
O
^{13}C NMR 100 MHz CDCl$_3$
f1 (ppm)

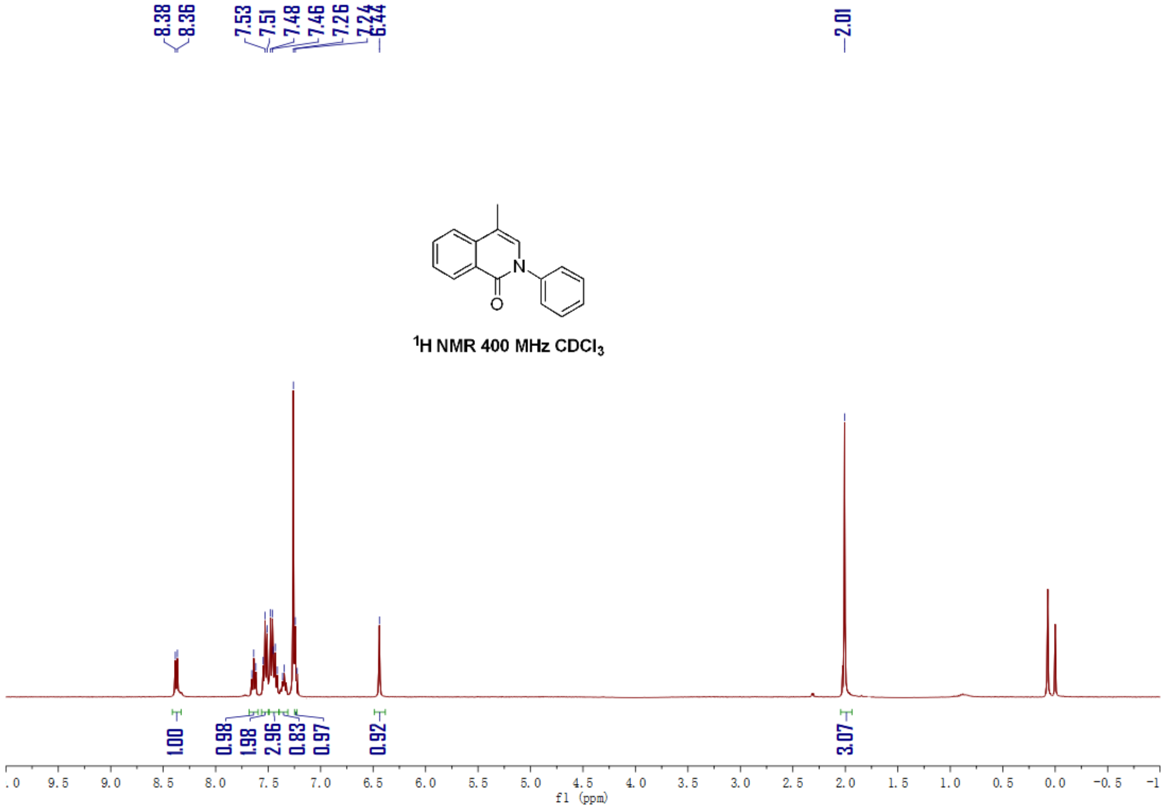
8.38
8.36
7.53
7.51
7.48
7.46
7.26
7.24
6.44
2.01
N
O
1H NMR 400 MHz CDCl3
1.00
0.98
1.98
2.96
0.83
0.97
0.92
3.07
f1 (ppm)

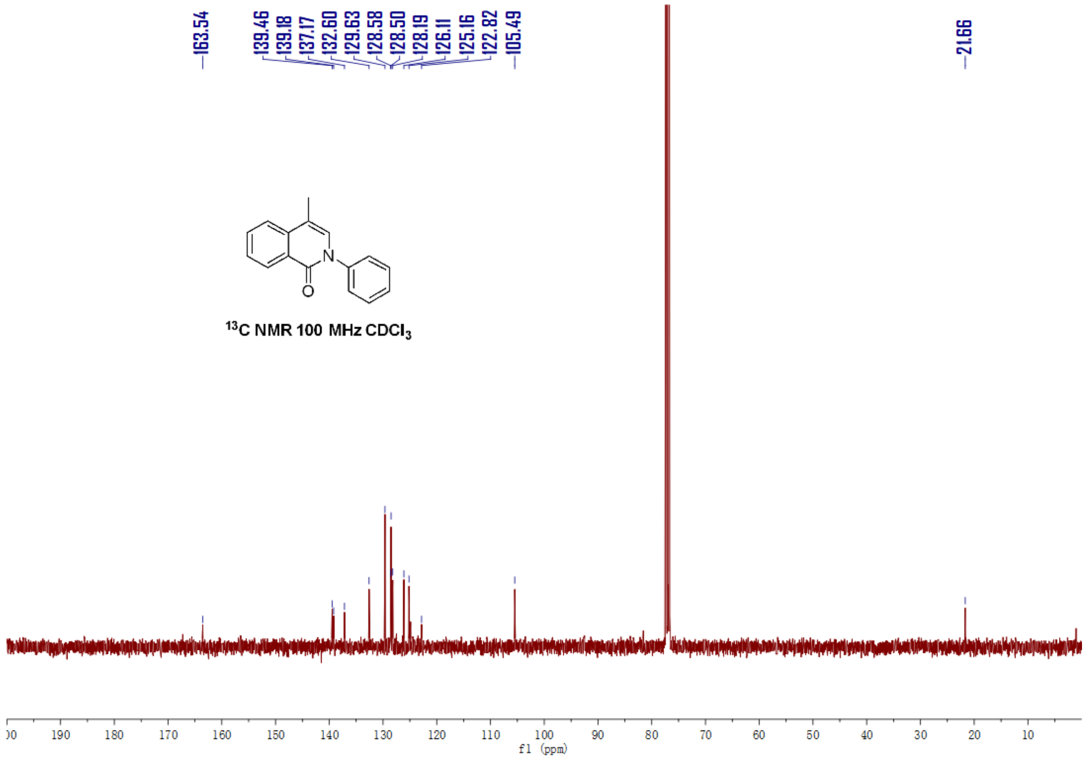
163.54
139.46
139.18
137.17
132.60
129.63
128.58
128.50
128.19
126.11
125.16
122.82
105.49
21.66
N
O
13C NMR 100 MHz CDCl3
f1 (ppm)

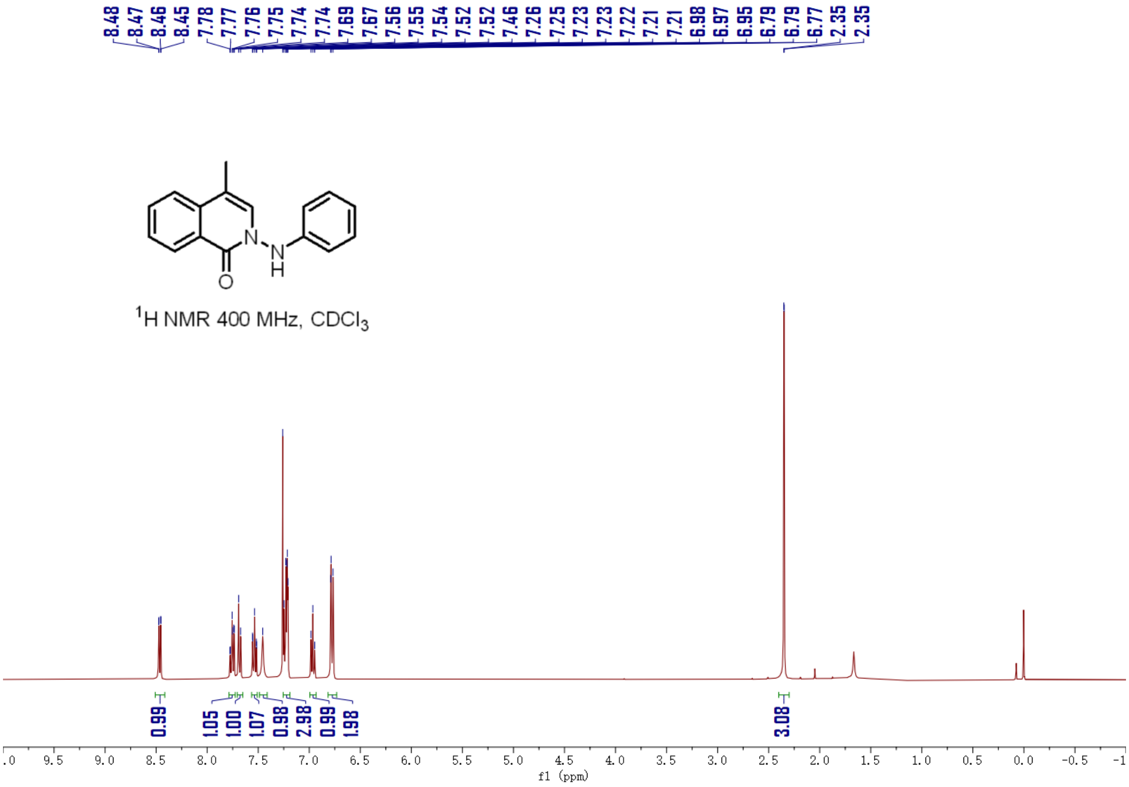
8.48
8.47
8.46
8.45
7.78
7.77
7.76
7.75
7.74
7.74
7.69
7.67
7.56
7.55
7.54
7.52
7.52
7.46
7.26
7.25
7.23
7.23
7.22
7.21
7.21
6.98
6.97
6.95
6.79
6.79
6.77
2.35
2.35
N
N
H
O
^{1}H NMR 400 MHz, $CDCl_3$
0.99
1.05
1.00
1.07
0.98
2.98
0.99
1.98
3.08
f1 (ppm)

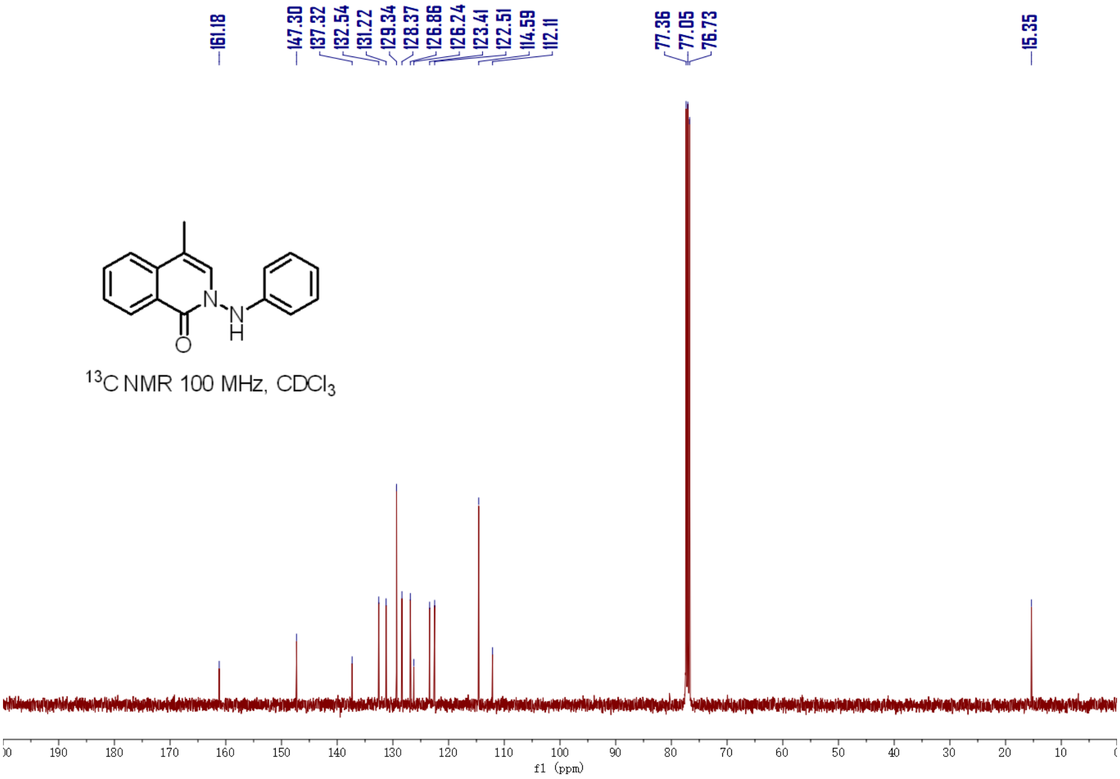
161.18
147.30
137.32
132.54
131.22
129.34
128.37
126.86
126.24
123.41
122.51
114.59
112.11
77.36
77.05
76.73
15.35
N
N
H
O
^{13}C NMR 100 MHz, $CDCl_3$
f1 (ppm)

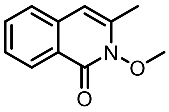

^1H NMR 400 MHz $CDCl_3$

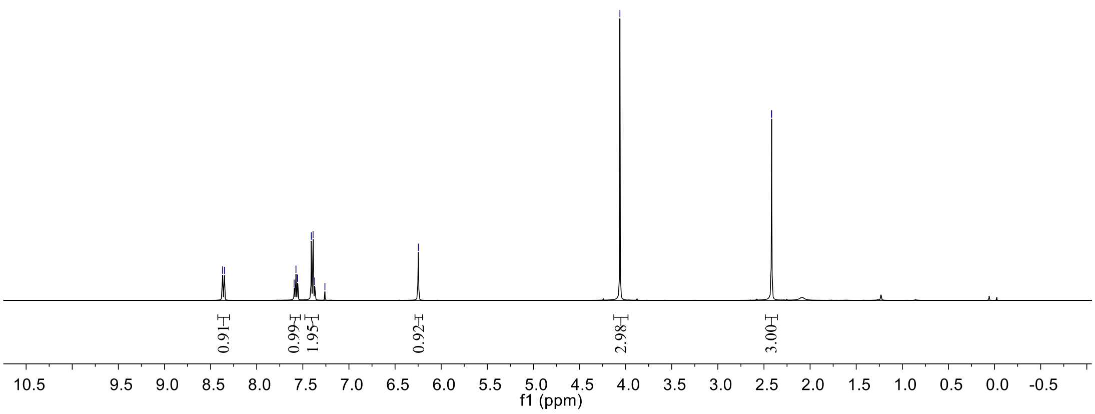

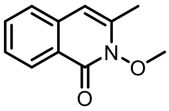

^{13}C NMR 100 MHz $CDCl_3$

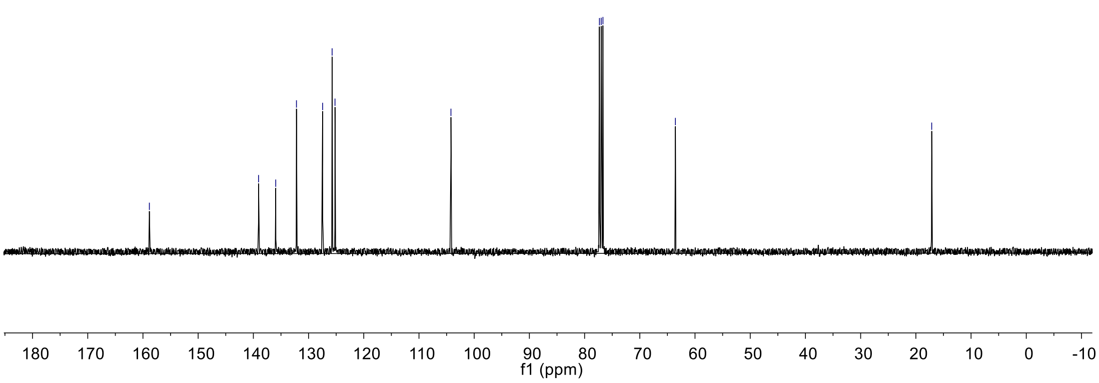

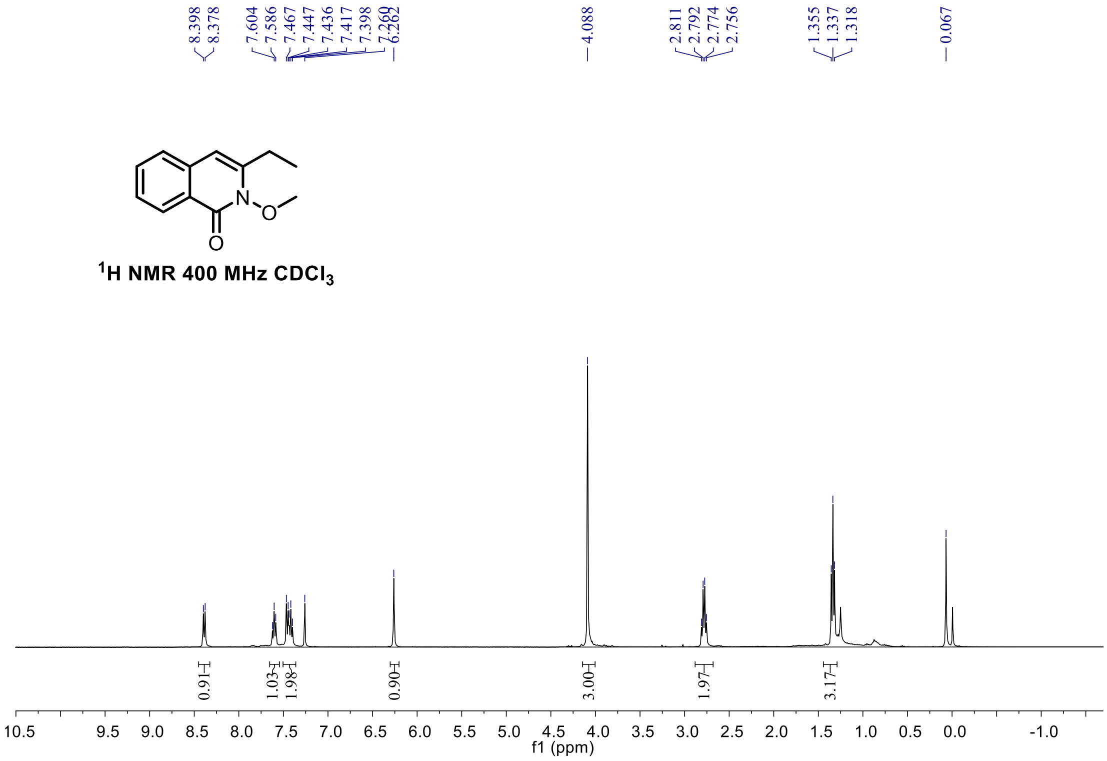
8.398
8.378
7.604
7.586
7.467
7.447
7.436
7.417
7.398
7.260
6.262
4.088
2.811
2.792
2.774
2.756
1.355
1.337
1.318
0.067
N
O
O
^{1}H NMR 400 MHz CDCl$_3$
0.91
1.03
1.98
0.90
3.00
1.97
3.17
f1 (ppm)

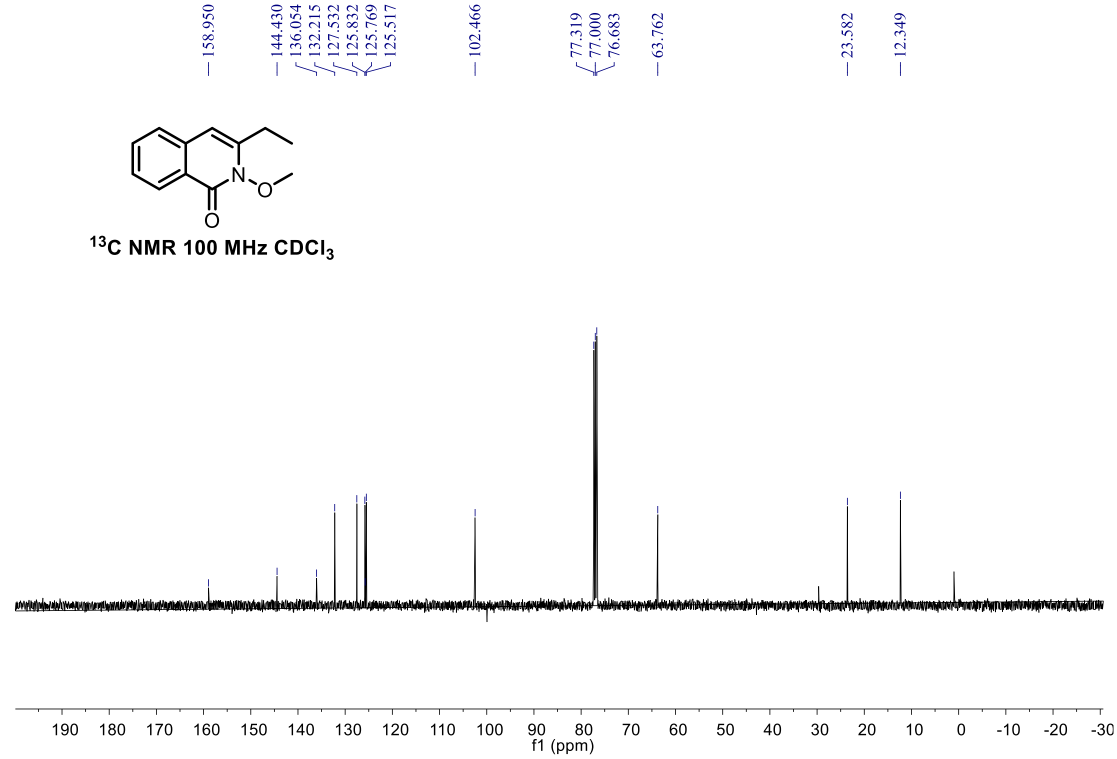
158.950
144.430
136.054
132.215
127.532
125.832
125.769
125.517
102.466
77.319
77.000
76.683
63.762
23.582
12.349
N
O
O
^{13}C NMR 100 MHz CDCl$_3$
f1 (ppm)

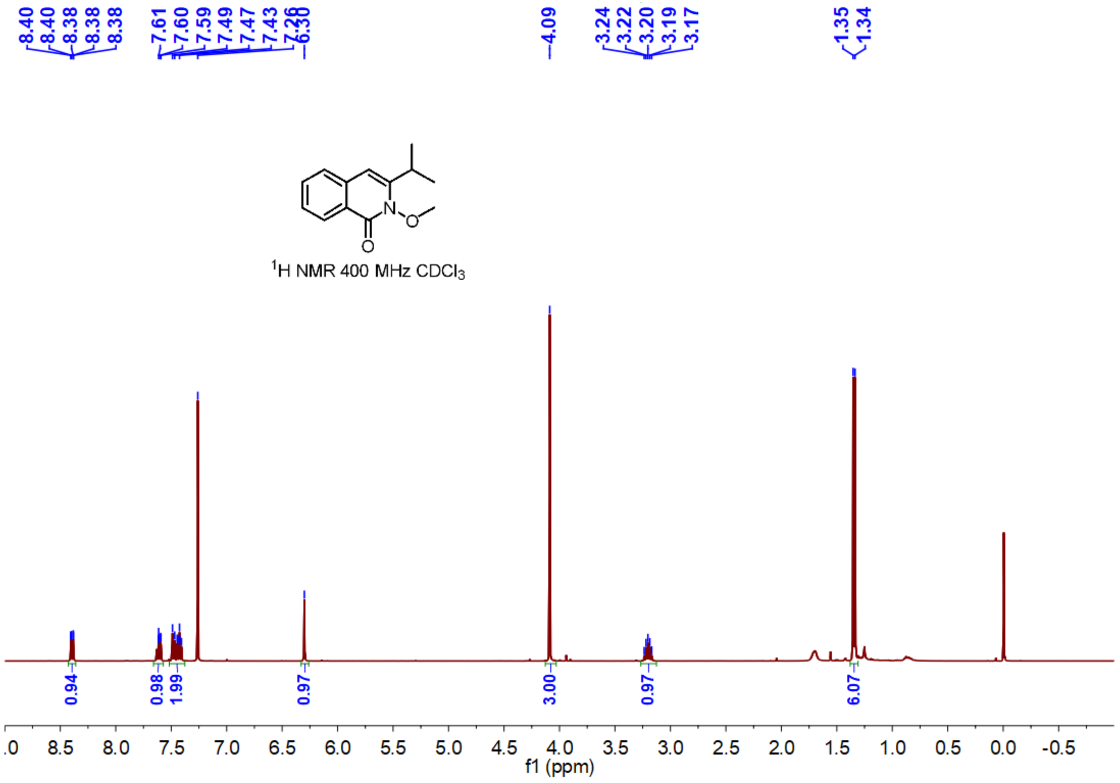
8.40
8.40
8.38
8.38
8.38
7.61
7.60
7.59
7.49
7.47
7.43
7.26
6.30
4.09
3.24
3.22
3.20
3.19
3.17
1.35
1.34
N
O
O
^{1}H NMR 400 MHz CDCl$_3$
0.94
0.98
1.99
0.97
3.00
0.97
6.07
f1 (ppm)

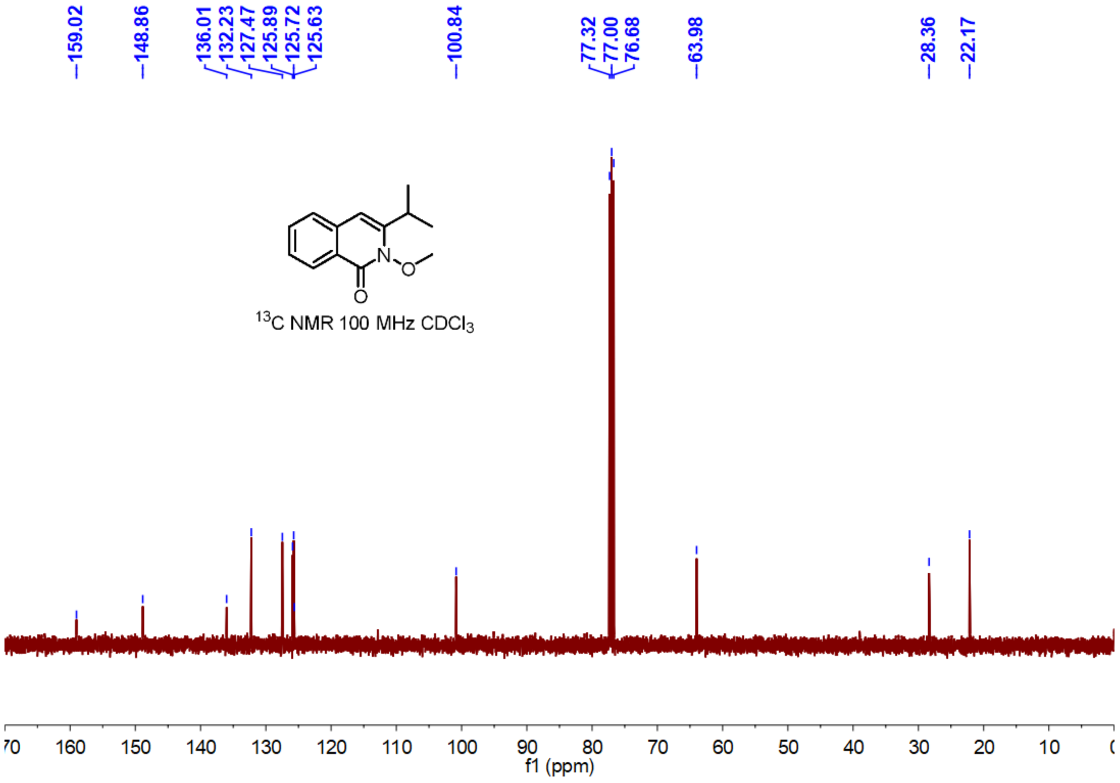
159.02
148.86
136.01
132.23
127.47
125.89
125.72
125.63
100.84
77.32
77.00
76.68
63.98
28.36
22.17
N
O
O
^{13}C NMR 100 MHz CDCl$_3$
f1 (ppm)

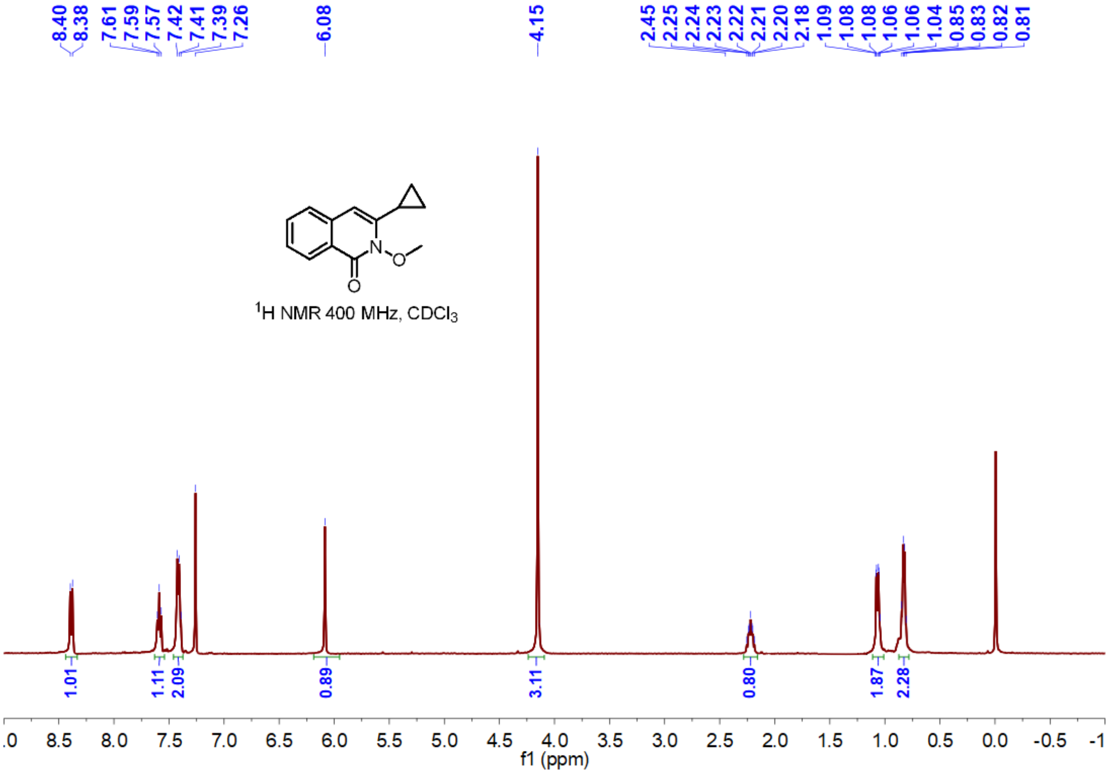

8.40
8.38
7.61
7.59
7.57
7.42
7.41
7.39
7.26
6.08
4.15
2.45
2.25
2.24
2.23
2.22
2.21
2.20
2.18
1.09
1.08
1.08
1.06
1.06
1.04
0.85
0.83
0.82
0.81
N
O
O
^{1}H NMR 400 MHz, CDCl$_3$
1.01
1.11
2.09
0.89
3.11
0.80
1.87
2.28
f1 (ppm)

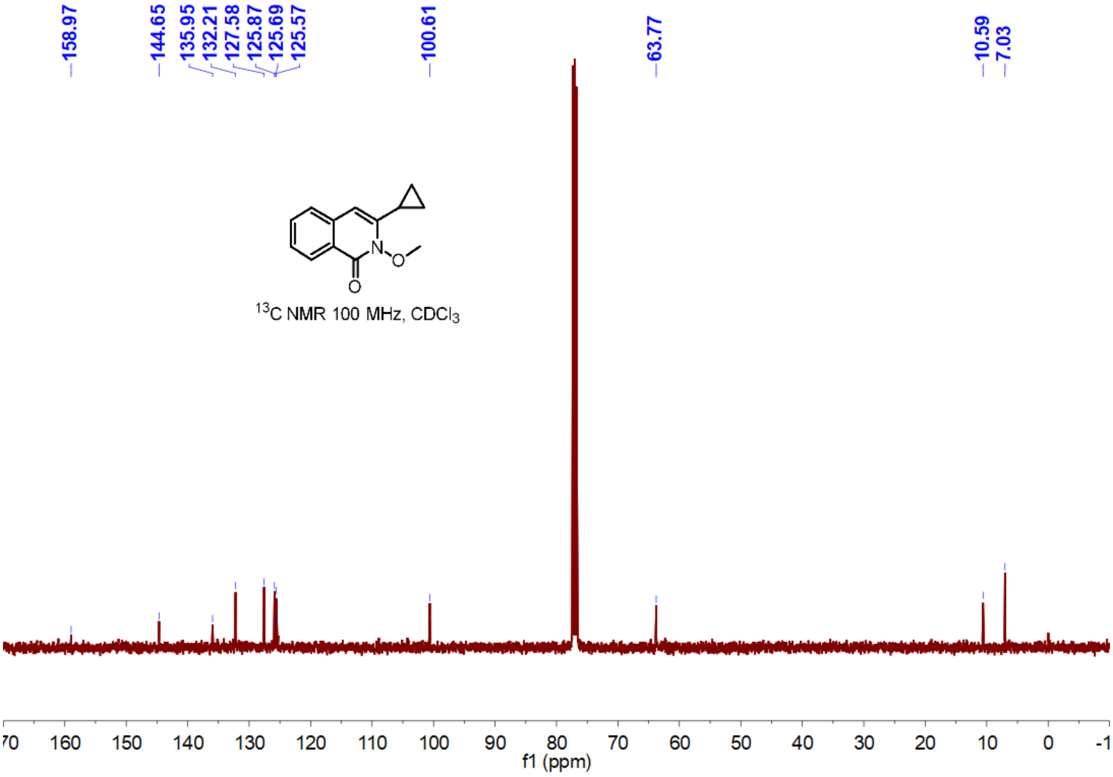

158.97
144.65
135.95
132.21
127.58
125.87
125.69
125.57
100.61
63.77
10.59
7.03
N
O
O
^{13}C NMR 100 MHz, CDCl$_3$
f1 (ppm)

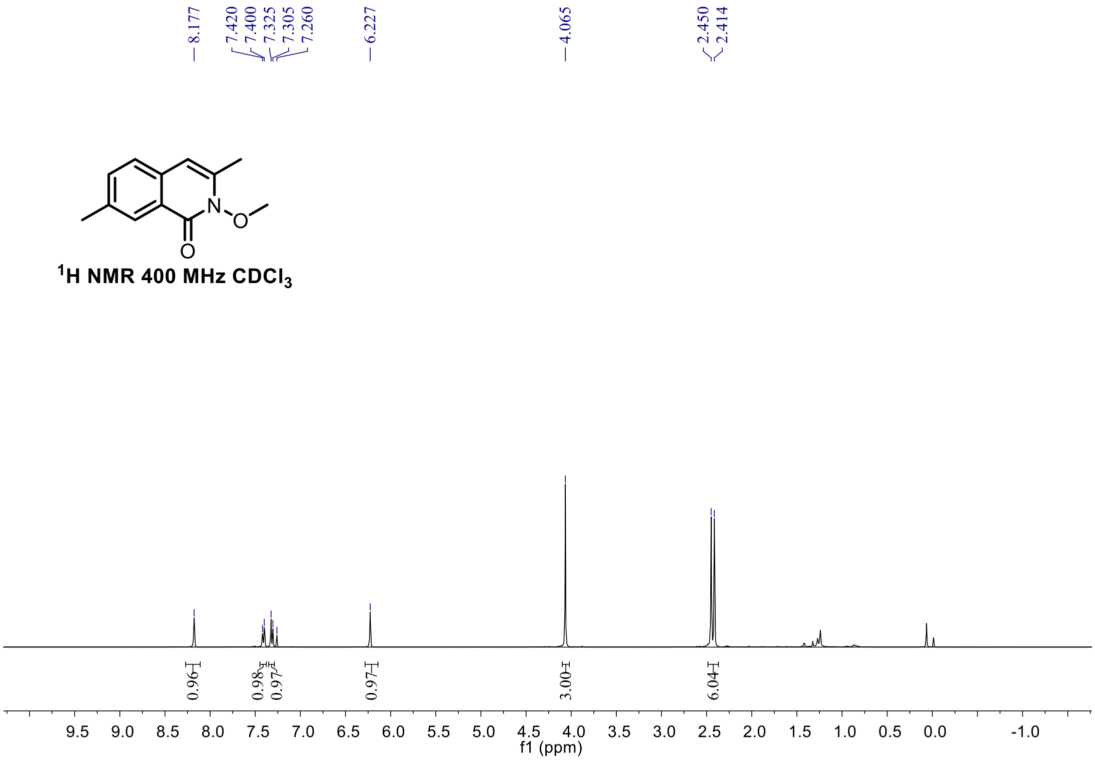
8.177
7.420
7.400
7.325
7.305
7.260
6.227
4.065
2.450
2.414
^{1}H NMR 400 MHz $CDCl_3$
0.96
0.98
0.97
0.97
3.00
6.04
f1 (ppm)

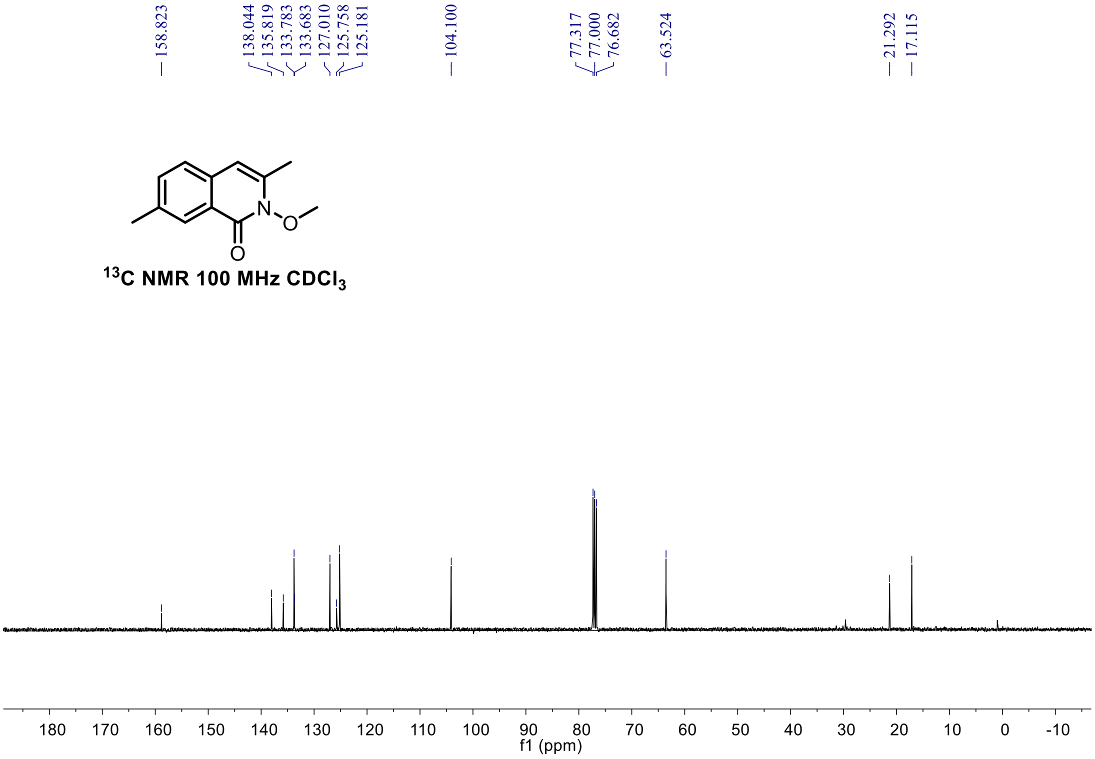
158.823
138.044
135.819
133.783
133.683
127.010
125.758
125.181
104.100
77.317
77.000
76.682
63.524
21.292
17.115
^{13}C NMR 100 MHz $CDCl_3$
f1 (ppm)

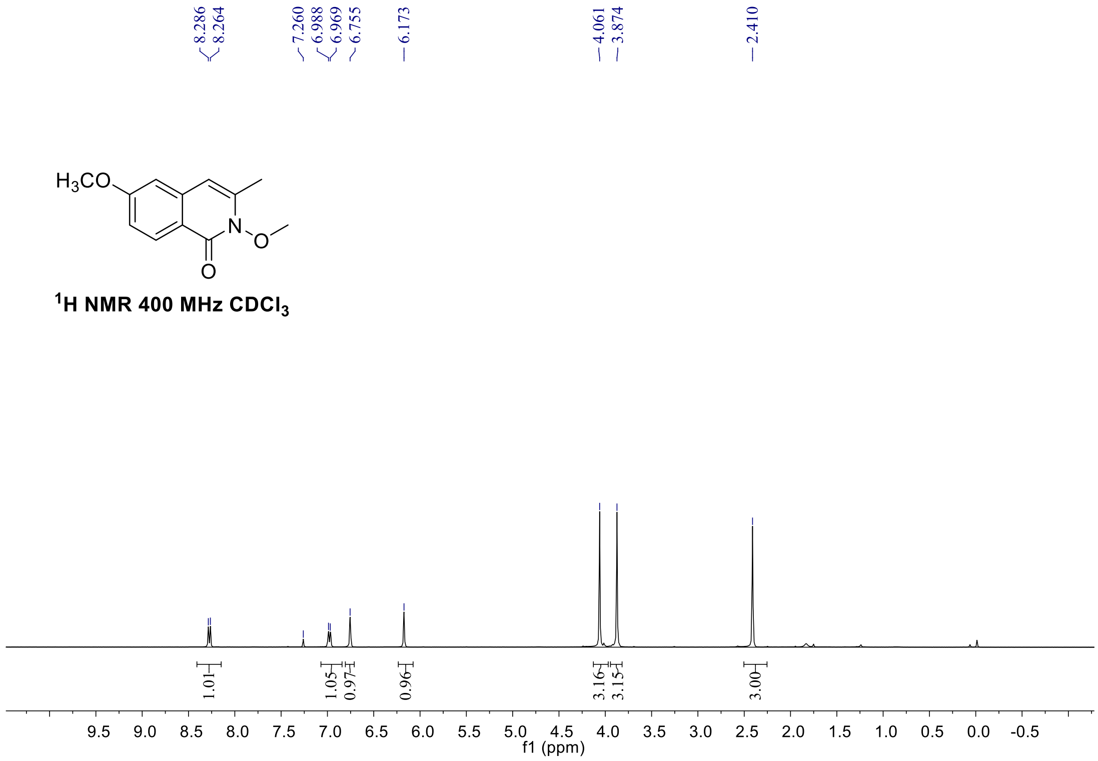
8.286
8.264
7.260
6.988
6.969
6.755
6.173
4.061
3.874
2.410
H3CO
N
O
O
1H NMR 400 MHz CDCl3
1.01
1.05
0.97
0.96
3.16
3.15
3.00
f1 (ppm)

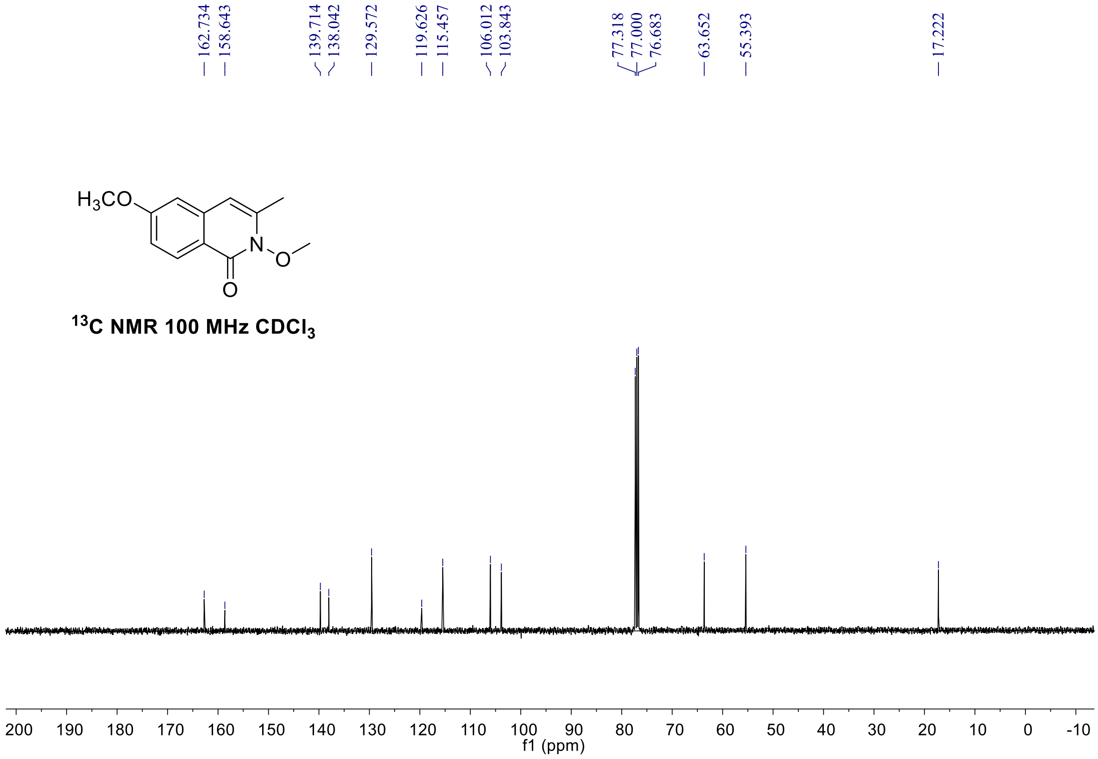
162.734
158.643
139.714
138.042
129.572
119.626
115.457
106.012
103.843
77.318
77.000
76.683
63.652
55.393
17.222
H3CO
N
O
O
13C NMR 100 MHz CDCl3
f1 (ppm)

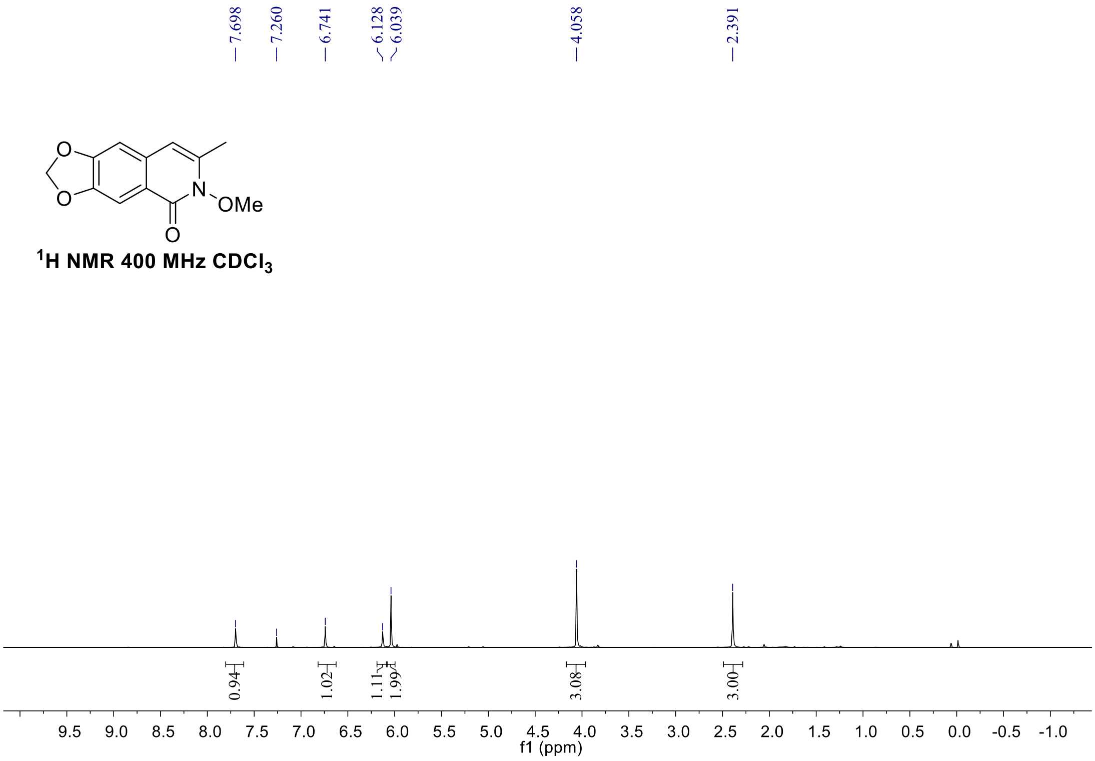
7.698
7.260
6.741
6.128
6.039
4.058
2.391
OMe
N
O
¹H NMR 400 MHz CDCl₃
0.94
1.02
1.11
1.99
3.08
3.00
9.5 9.0 8.5 8.0 7.5 7.0 6.5 6.0 5.5 5.0 4.5 4.0 3.5 3.0 2.5 2.0 1.5 1.0 0.5 0.0 -0.5 -1.0
f1 (ppm)

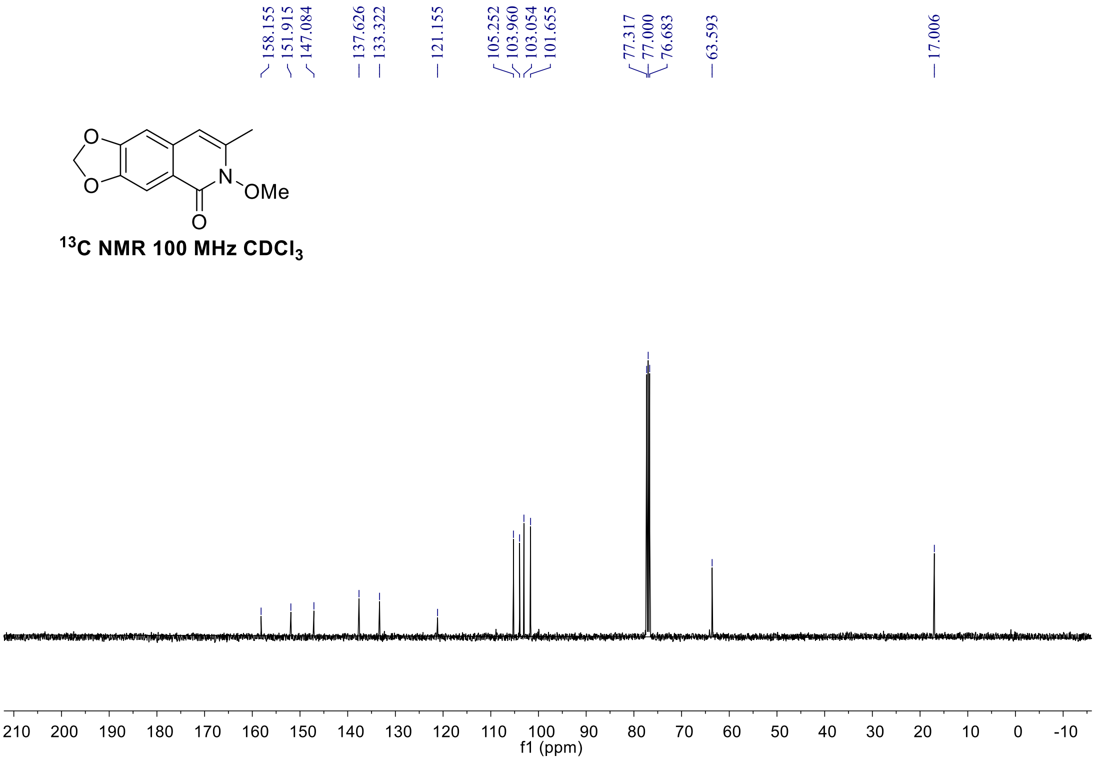
158.155
151.915
147.084
137.626
133.322
121.155
105.252
103.960
103.054
101.655
77.317
77.000
76.683
63.593
17.006
OMe
N
O
¹³C NMR 100 MHz CDCl₃
210 200 190 180 170 160 150 140 130 120 110 100 90 80 70 60 50 40 30 20 10 0 -10
f1 (ppm)

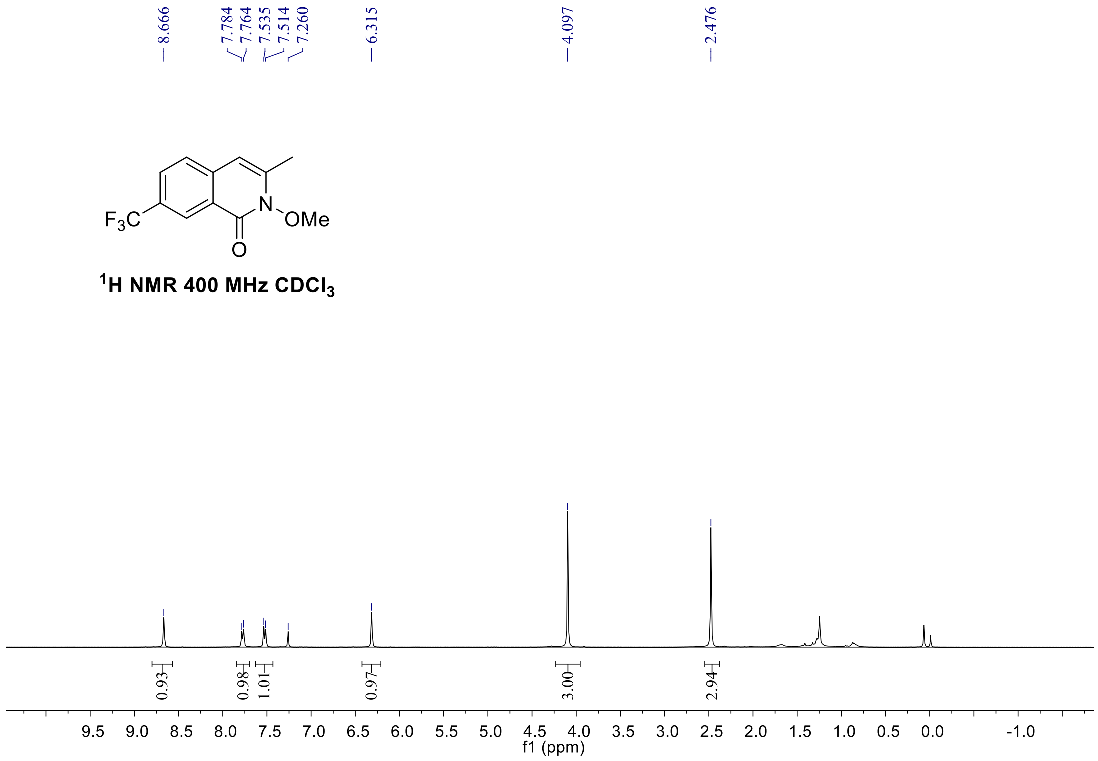
8.666
7.784
7.764
7.535
7.514
7.260
6.315
4.097
2.476
F3C
N
OMe
O
1H NMR 400 MHz CDCl3
0.93
0.98
1.01
0.97
3.00
2.94
9.5 9.0 8.5 8.0 7.5 7.0 6.5 6.0 5.5 5.0 4.5 4.0 3.5 3.0 2.5 2.0 1.5 1.0 0.5 0.0 -1.0
f1 (ppm)

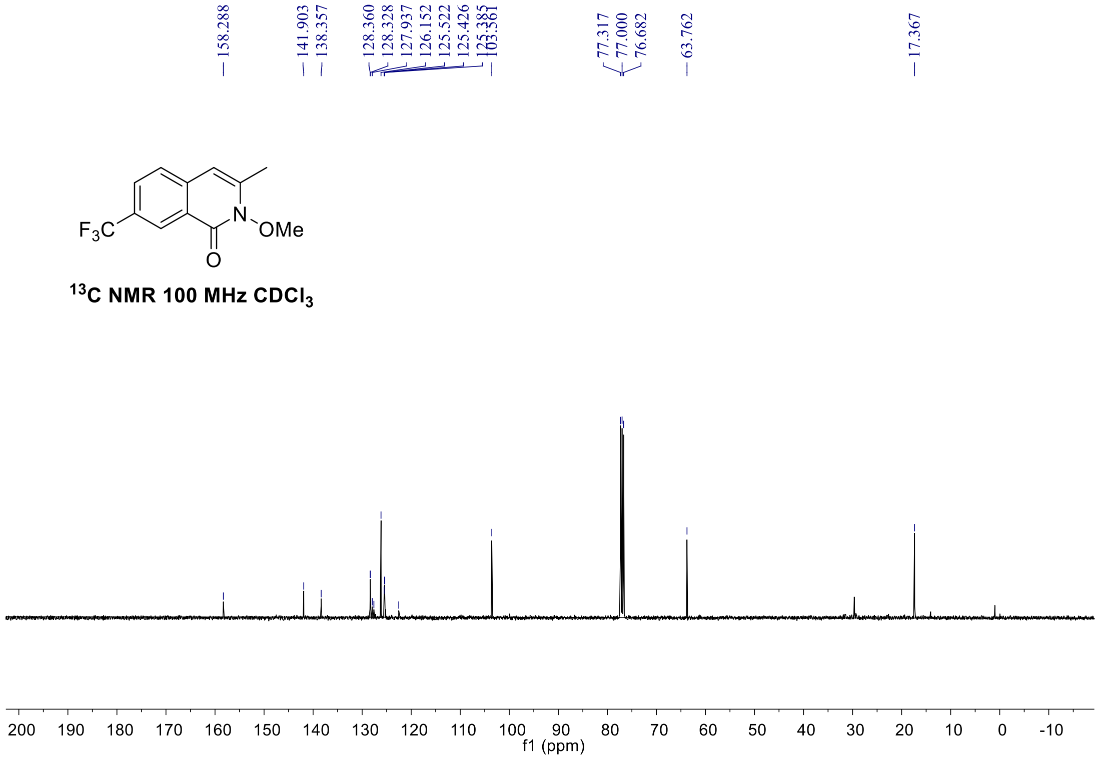
158.288
141.903
138.357
128.360
128.328
127.937
126.152
125.522
125.426
125.385
103.561
77.317
77.000
76.682
63.762
17.367
F3C
N
OMe
O
13C NMR 100 MHz CDCl3
200 190 180 170 160 150 140 130 120 110 100 90 80 70 60 50 40 30 20 10 0 -10
f1 (ppm)

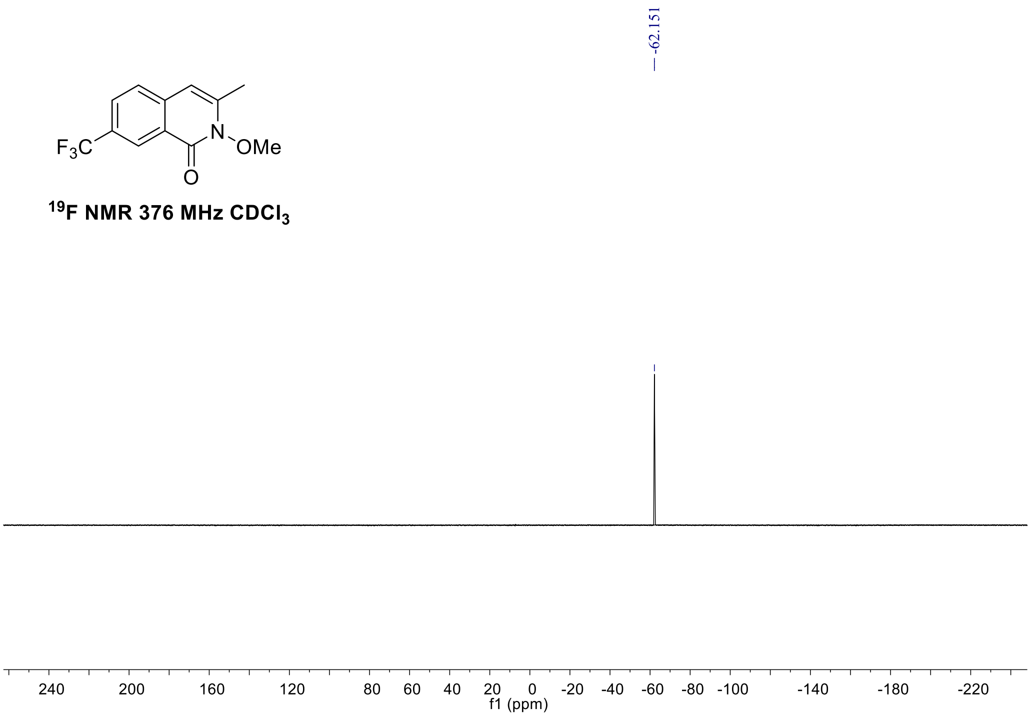

-62.151
F3C
N
OMe
O
19F NMR 376 MHz CDCl3
240 200 160 120 80 60 40 20 0 -20 -40 -60 -80 -100 -140 -180 -220
f1 (ppm)

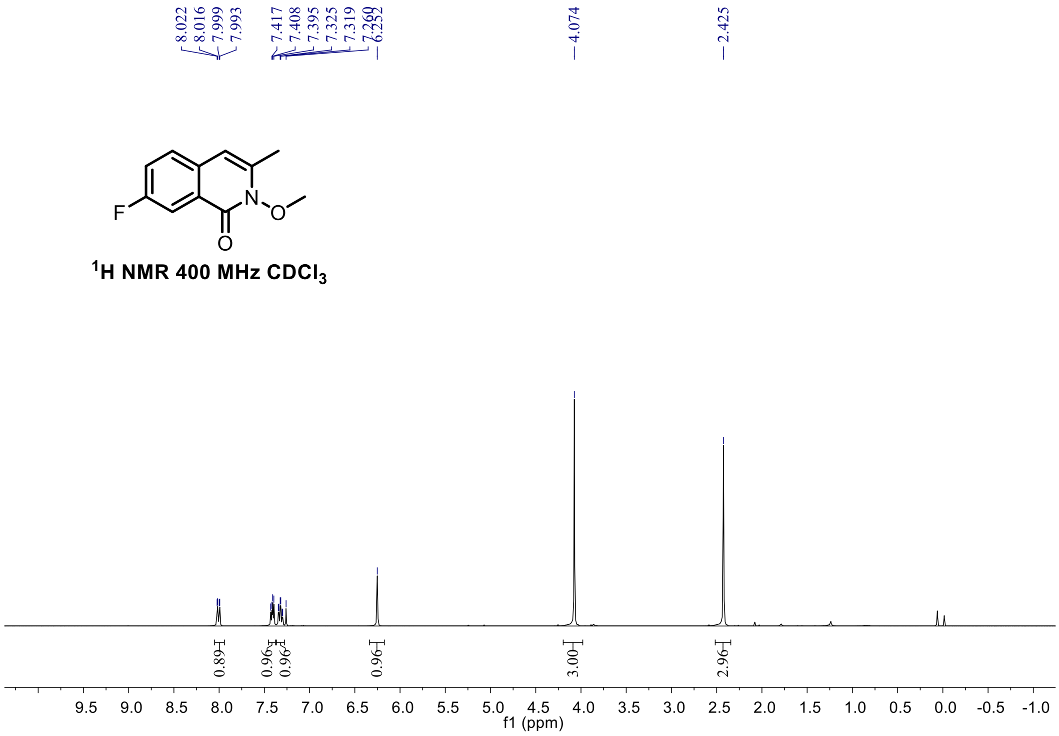

8.022
8.016
7.999
7.993
7.417
7.408
7.395
7.325
7.319
7.260
6.252
4.074
2.425
F
N
O
O
1H NMR 400 MHz CDCl3
0.89
0.96
0.96
0.96
3.00
2.96
9.5 9.0 8.5 8.0 7.5 7.0 6.5 6.0 5.5 5.0 4.5 4.0 3.5 3.0 2.5 2.0 1.5 1.0 0.5 0.0 -0.5 -1.0
f1 (ppm)

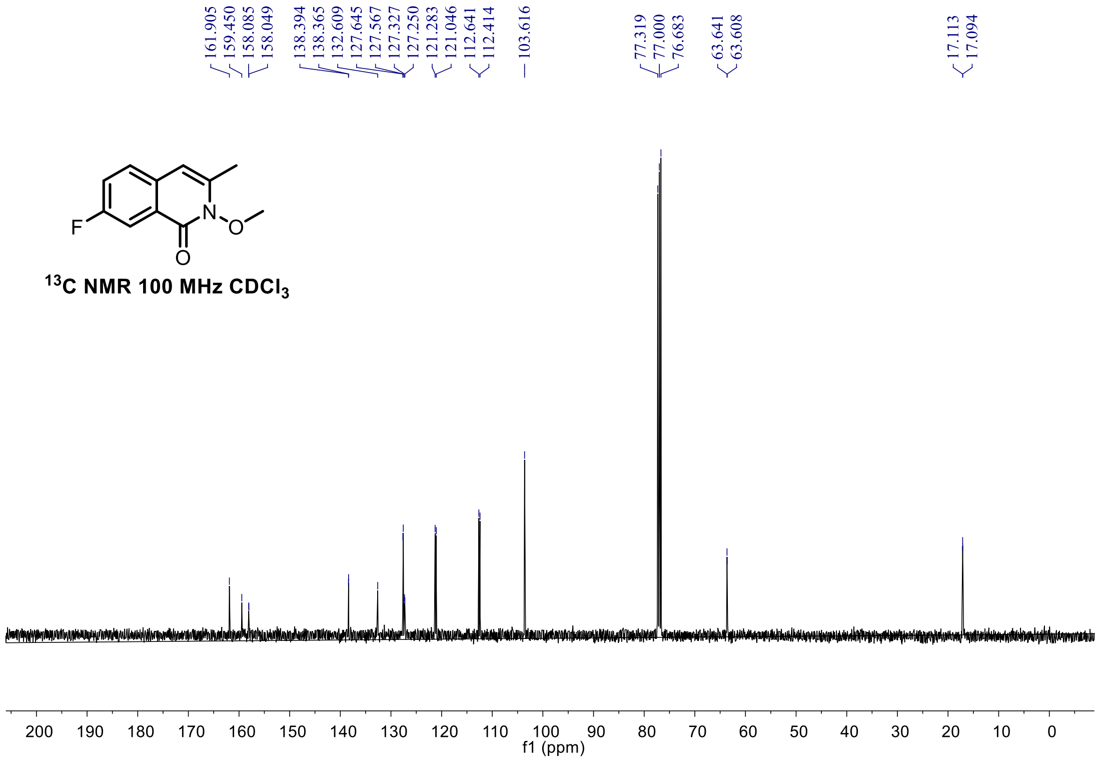
161.905
159.450
158.085
158.049
138.394
138.365
132.609
127.645
127.567
127.327
127.250
121.283
121.046
112.641
112.414
103.616
77.319
77.000
76.683
63.641
63.608
17.113
17.094
F
N
O
O
^{13}C NMR 100 MHz $CDCl_3$
200 190 180 170 160 150 140 130 120 110 100 90 80 70 60 50 40 30 20 10 0
f1 (ppm)

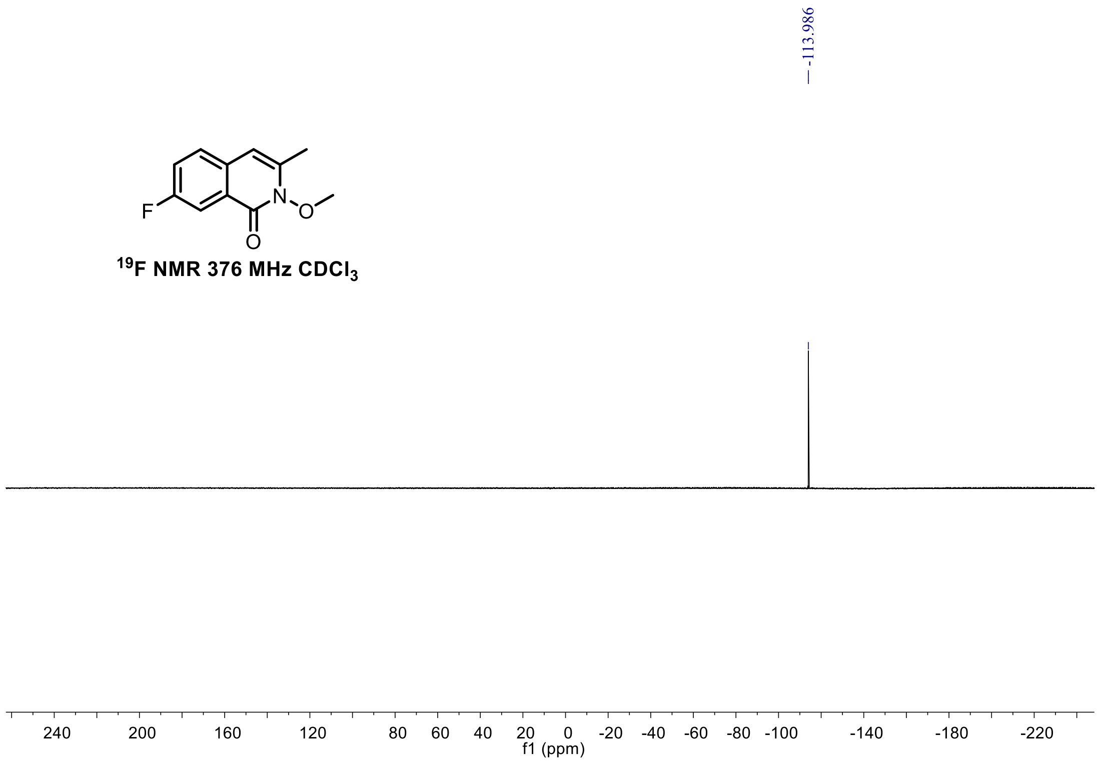
-113.986
F
N
O
O
^{19}F NMR 376 MHz $CDCl_3$
240 200 160 120 80 60 40 20 0 -20 -40 -60 -80 -100 -140 -180 -220
f1 (ppm)

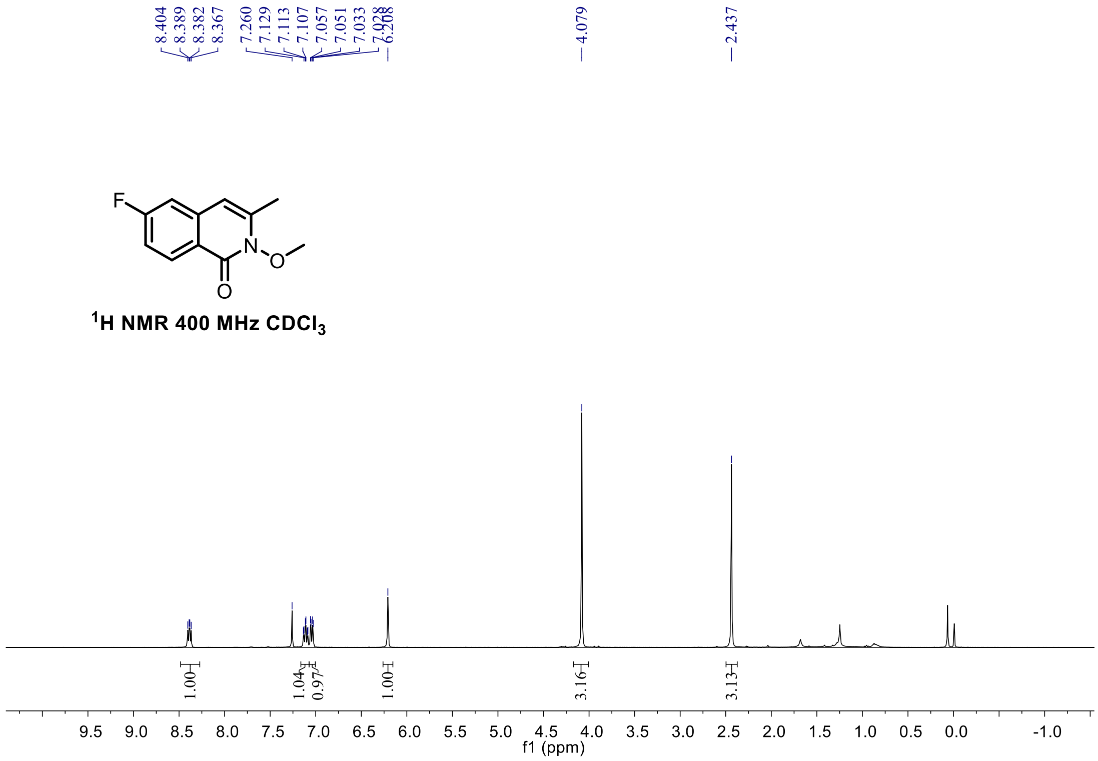
8.404
8.389
8.382
8.367
7.260
7.129
7.113
7.107
7.057
7.051
7.033
7.028
6.208
4.079
2.437
F
N
O
O
1H NMR 400 MHz CDCl3
1.00
1.04
0.97
1.00
3.16
3.13
9.5 9.0 8.5 8.0 7.5 7.0 6.5 6.0 5.5 5.0 4.5 4.0 3.5 3.0 2.5 2.0 1.5 1.0 0.5 0.0 -1.0
f1 (ppm)

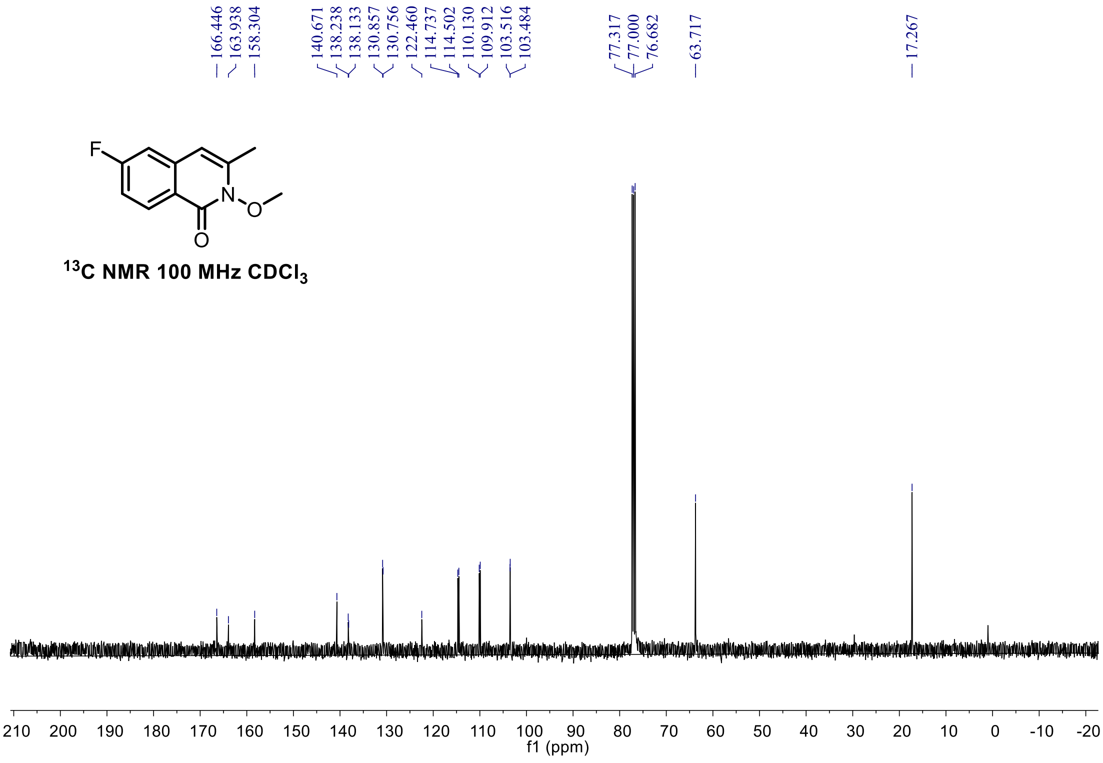
166.446
163.938
158.304
140.671
138.238
138.133
130.857
130.756
122.460
114.737
114.502
110.130
109.912
103.516
103.484
77.317
77.000
76.682
63.717
17.267
F
N
O
O
13C NMR 100 MHz CDCl3
210 200 190 180 170 160 150 140 130 120 110 100 90 80 70 60 50 40 30 20 10 0 -10 -20
f1 (ppm)

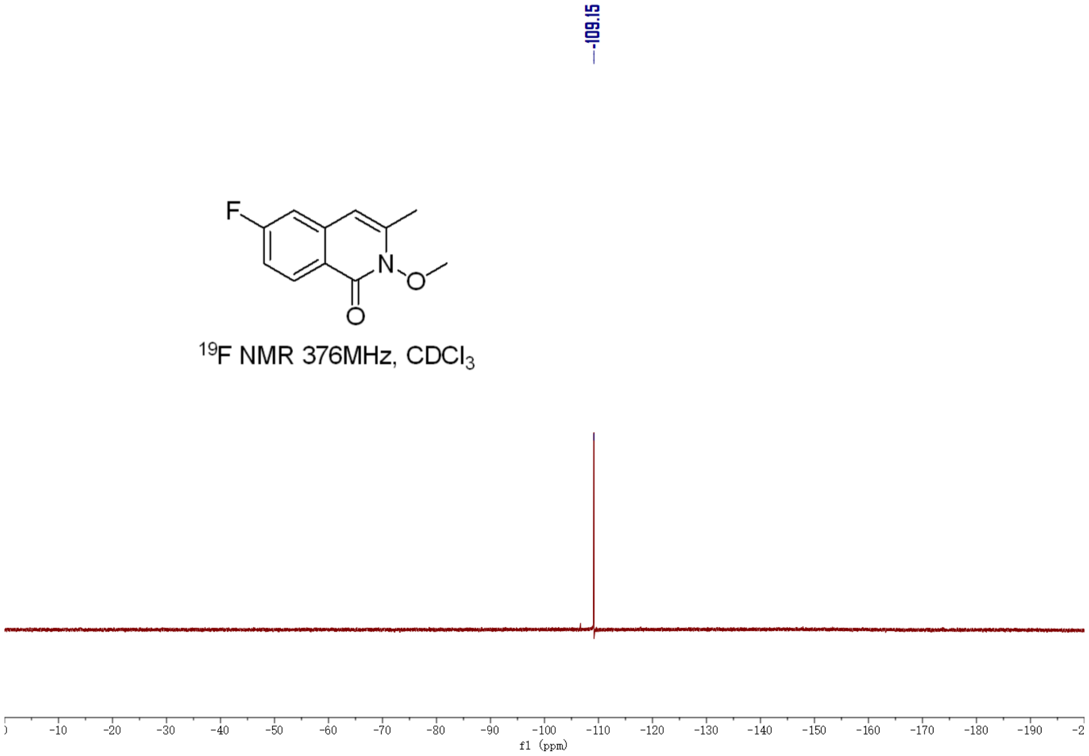

-109.15
F
N
O
O
^{19}F NMR 376MHz, CDCl$_3$
f1 (ppm)

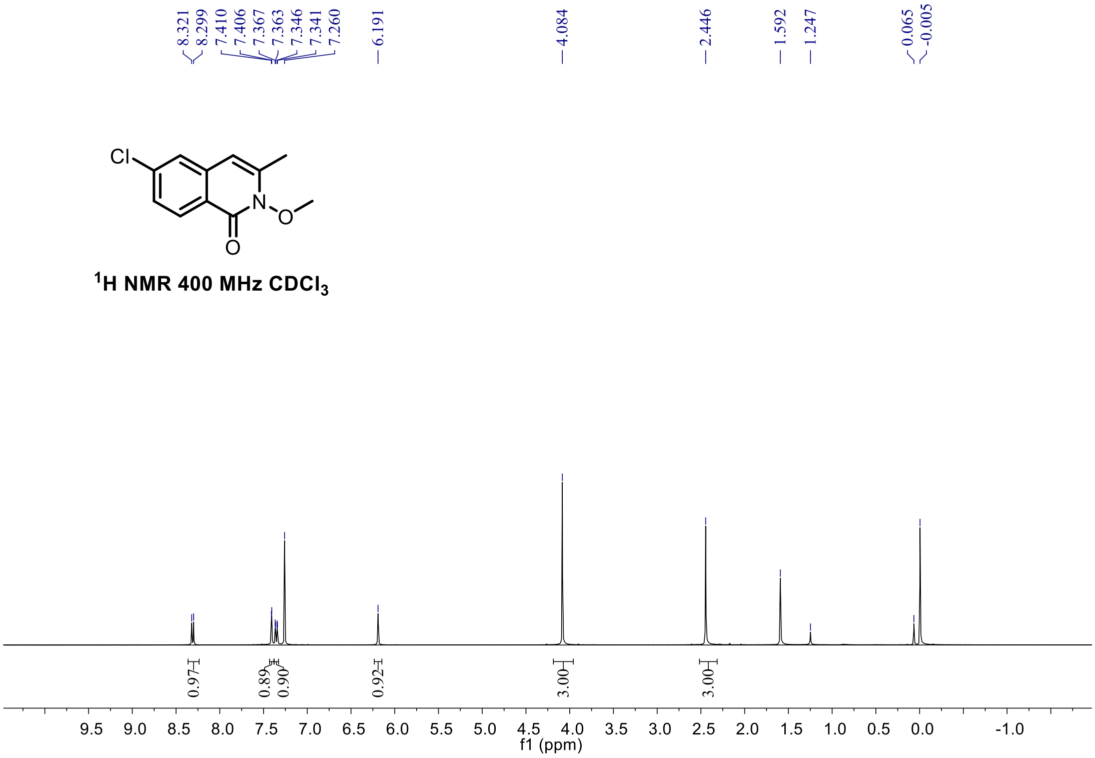

8.321
8.299
7.410
7.406
7.367
7.363
7.346
7.341
7.260
6.191
4.084
2.446
1.592
1.247
0.065
-0.005
Cl
N
O
O
^{1}H NMR 400 MHz CDCl$_3$
0.97
0.89
0.90
0.92
3.00
3.00
f1 (ppm)

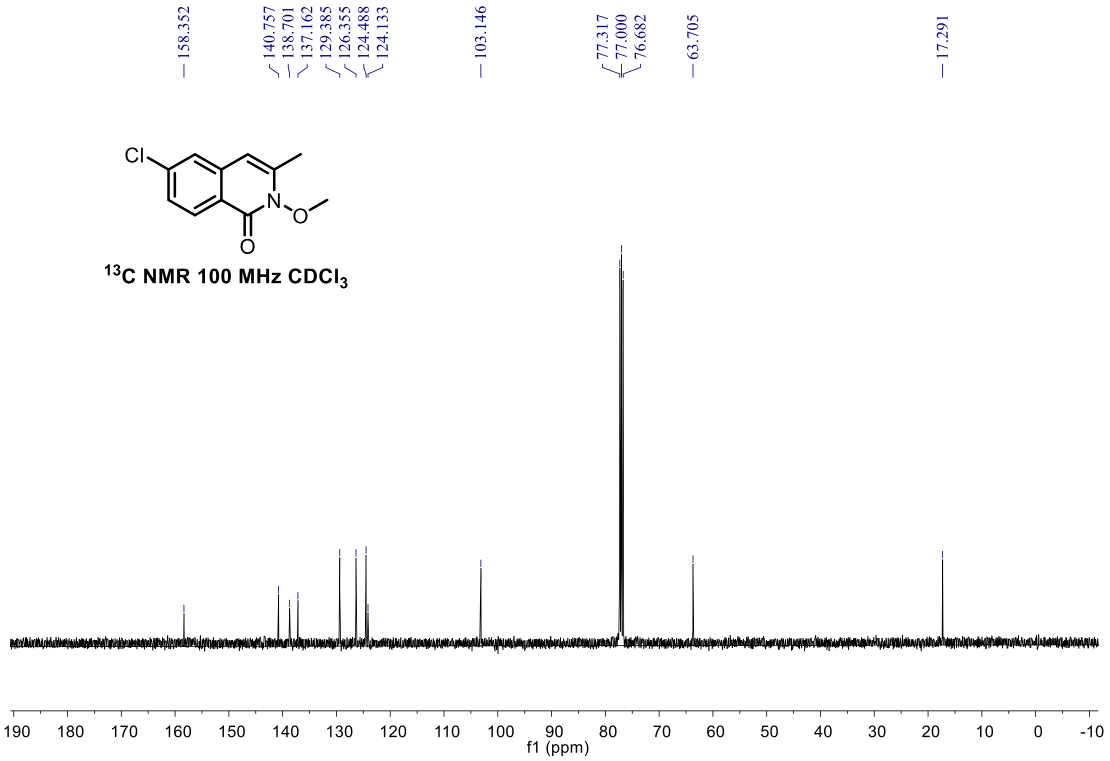
158.352
140.757
138.701
137.162
129.385
126.355
124.488
124.133
103.146
77.317
77.000
76.682
63.705
17.291
Cl
N
O
O
^{13}C NMR 100 MHz CDCl$_3$
190 180 170 160 150 140 130 120 110 100 90 80 70 60 50 40 30 20 10 0 -10
f1 (ppm)

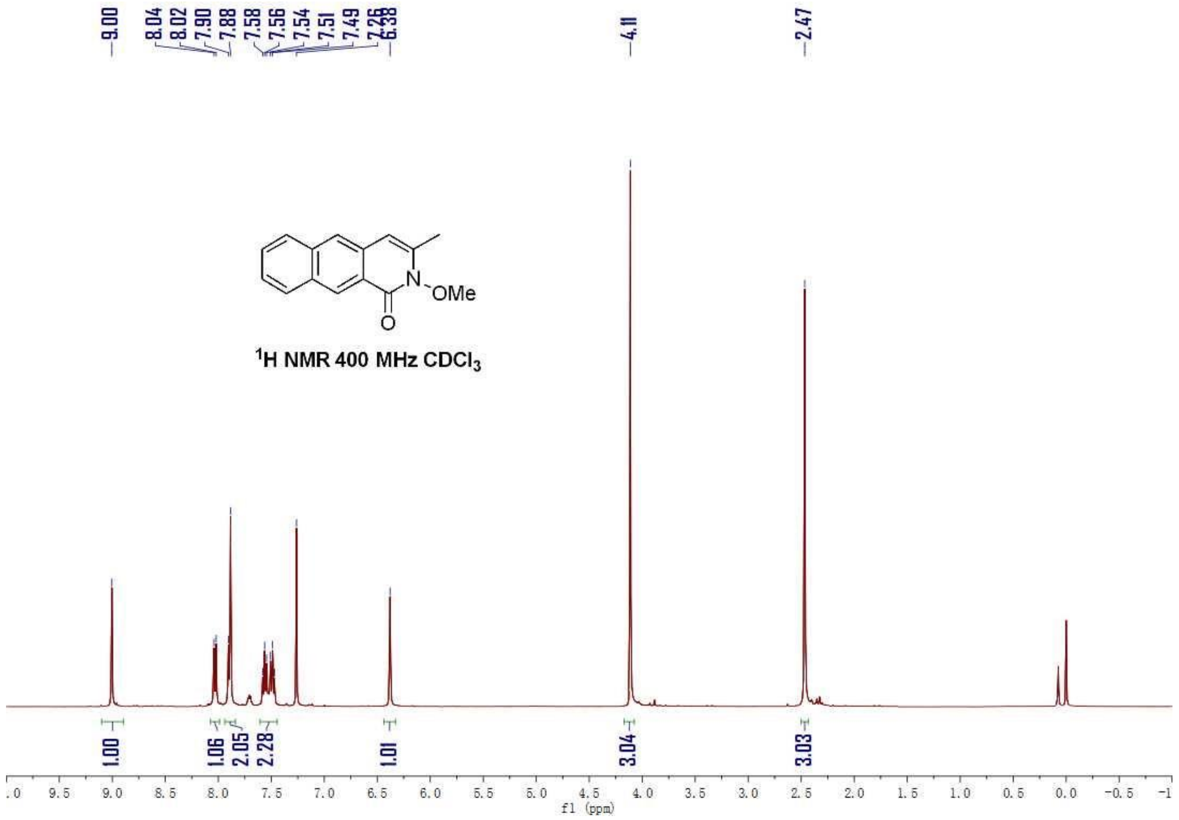
9.00
8.04
8.02
7.90
7.88
7.58
7.56
7.54
7.51
7.49
7.26
6.38
4.11
2.47
N
OMe
O
^{1}H NMR 400 MHz CDCl$_3$
1.00
1.06
2.05
2.28
1.01
3.04
3.03
9.5 9.0 8.5 8.0 7.5 7.0 6.5 6.0 5.5 5.0 4.5 4.0 3.5 3.0 2.5 2.0 1.5 1.0 0.5 0.0 -0.5
f1 (ppm)

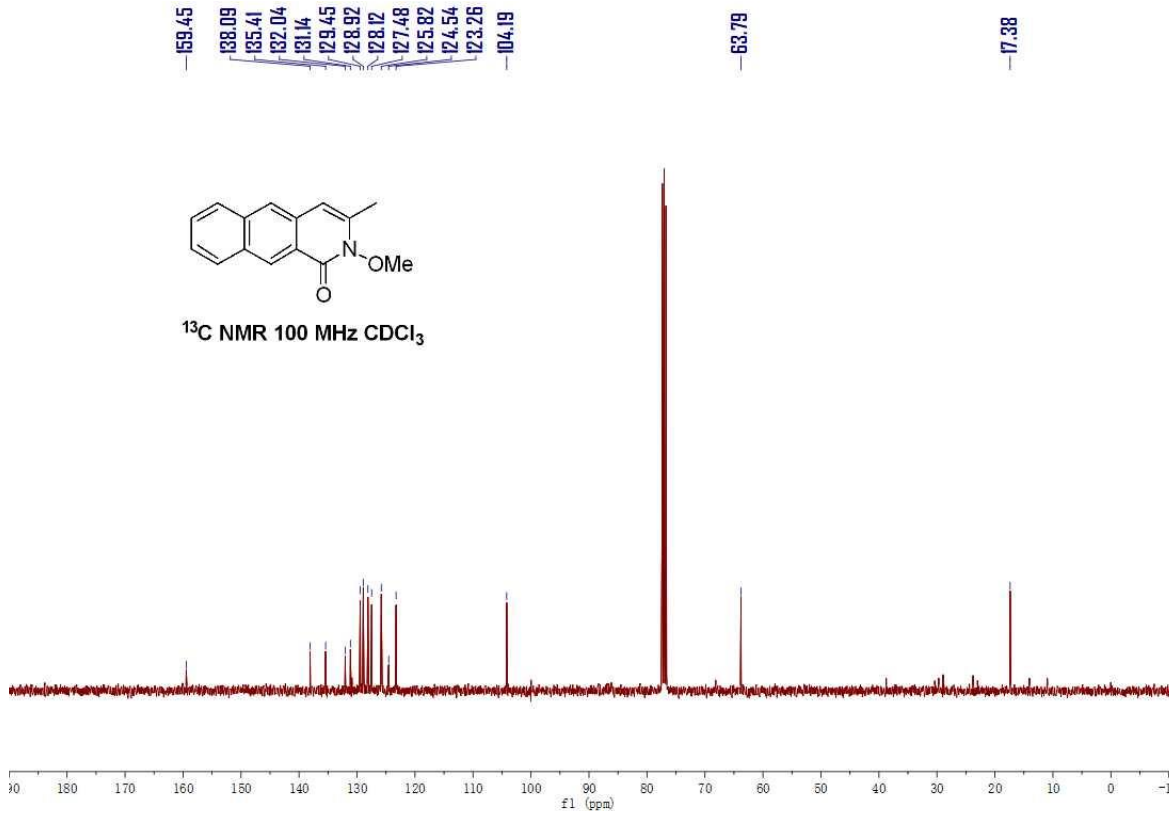
159.45
138.09
135.41
132.04
131.14
129.45
128.92
128.12
127.48
125.82
124.54
123.26
104.19
63.79
17.38
N
OMe
O
^{13}C NMR 100 MHz CDCl$_3$
f1 (ppm)

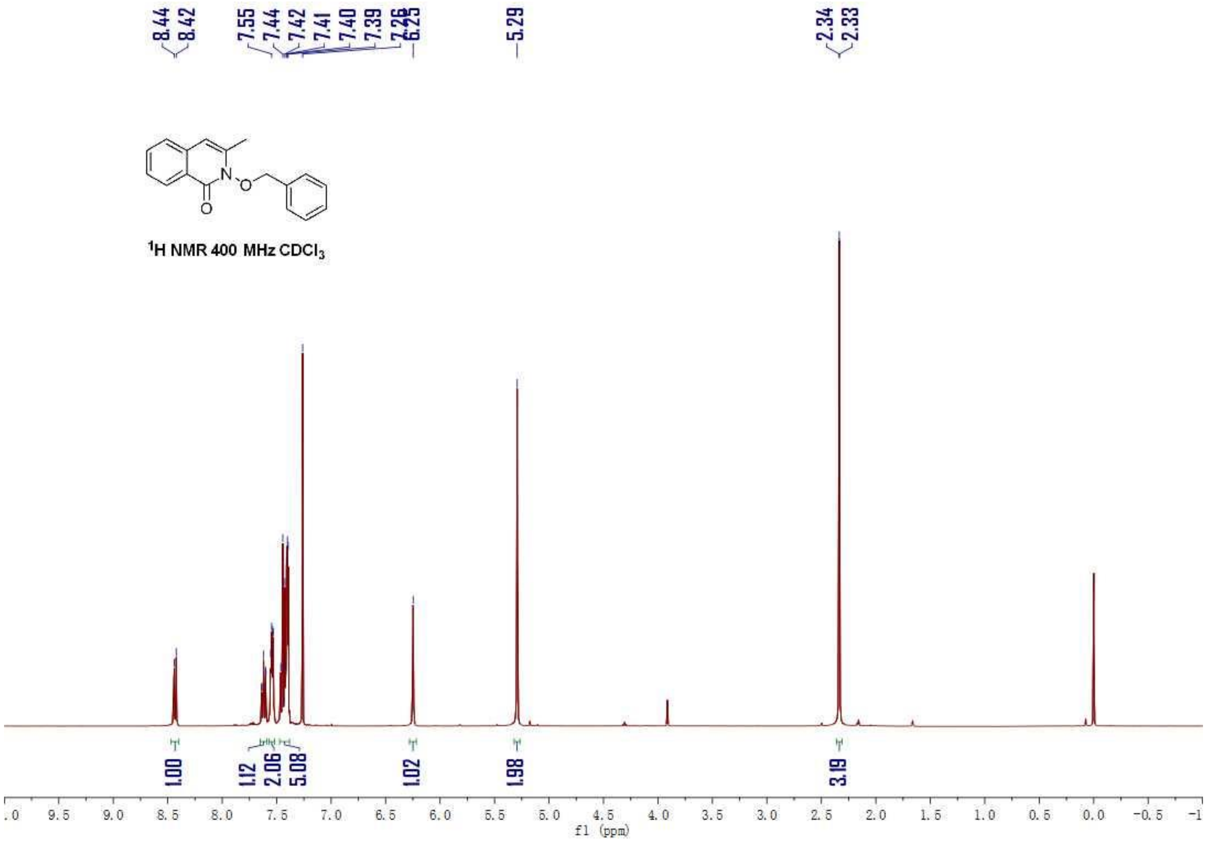
8.44
8.42
7.55
7.44
7.42
7.41
7.40
7.39
7.26
6.25
5.29
2.34
2.33
N
O
O
^{1}H NMR 400 MHz CDCl$_3$
1.00
1.12
2.06
5.08
1.02
1.98
3.19
f1 (ppm)

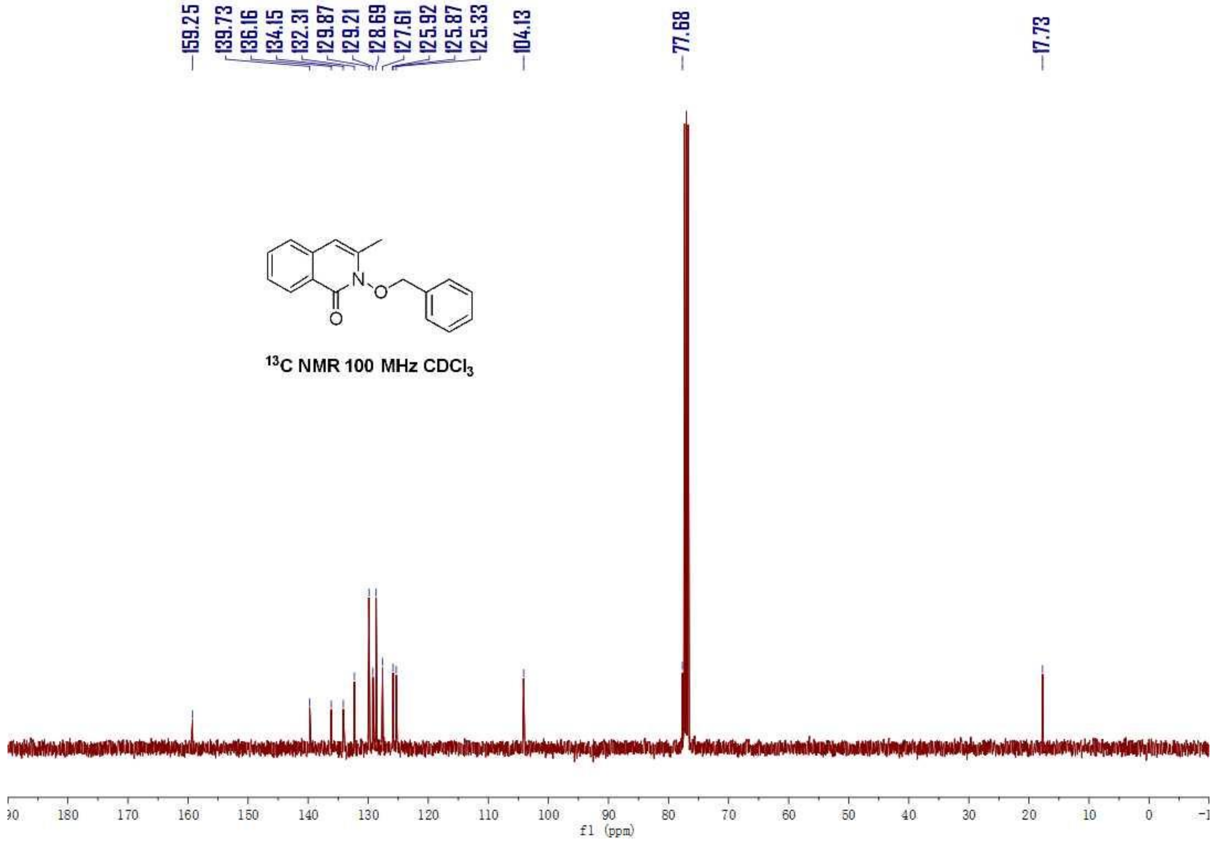
159.25
139.73
136.16
134.15
132.31
129.87
129.21
128.69
127.61
125.92
125.87
125.33
104.13
77.68
17.73
^{13}C NMR 100 MHz $CDCl_3$
f1 (ppm)

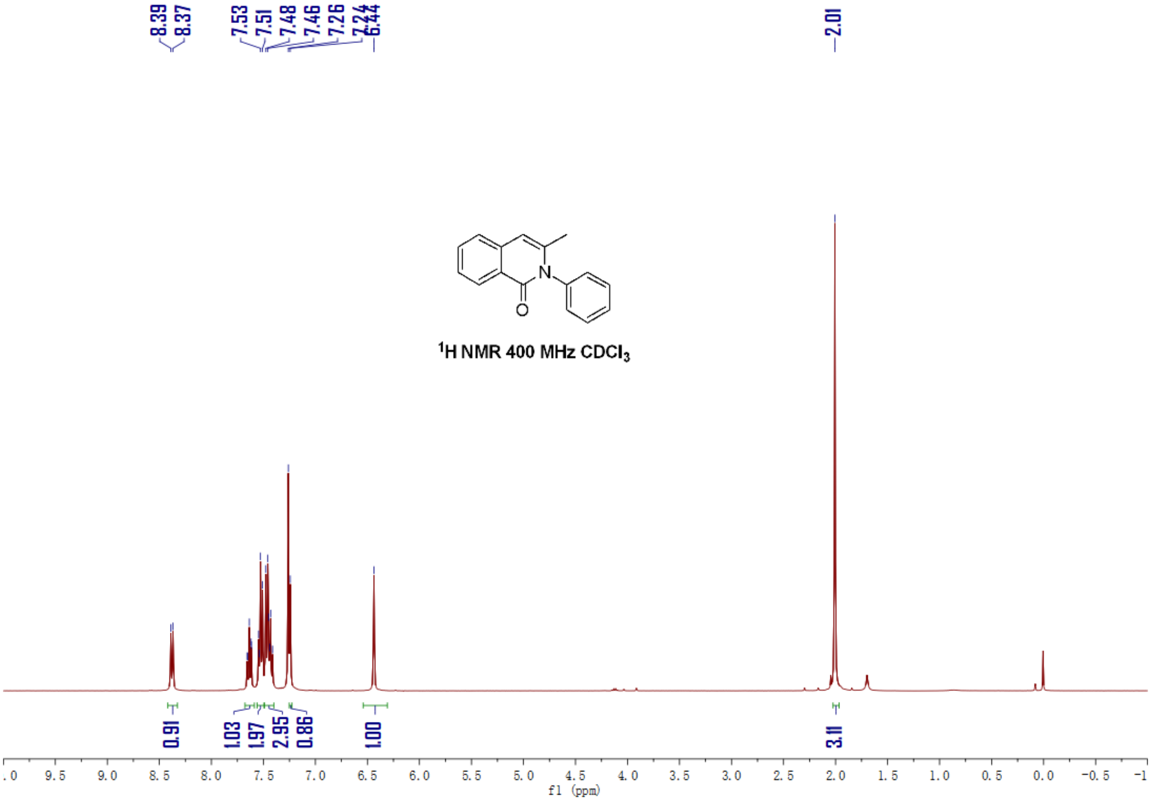
8.39
8.37
7.53
7.51
7.48
7.46
7.26
7.24
6.44
2.01
^{1}H NMR 400 MHz $CDCl_3$
0.91
1.03
1.97
2.95
0.86
1.00
3.11
f1 (ppm)

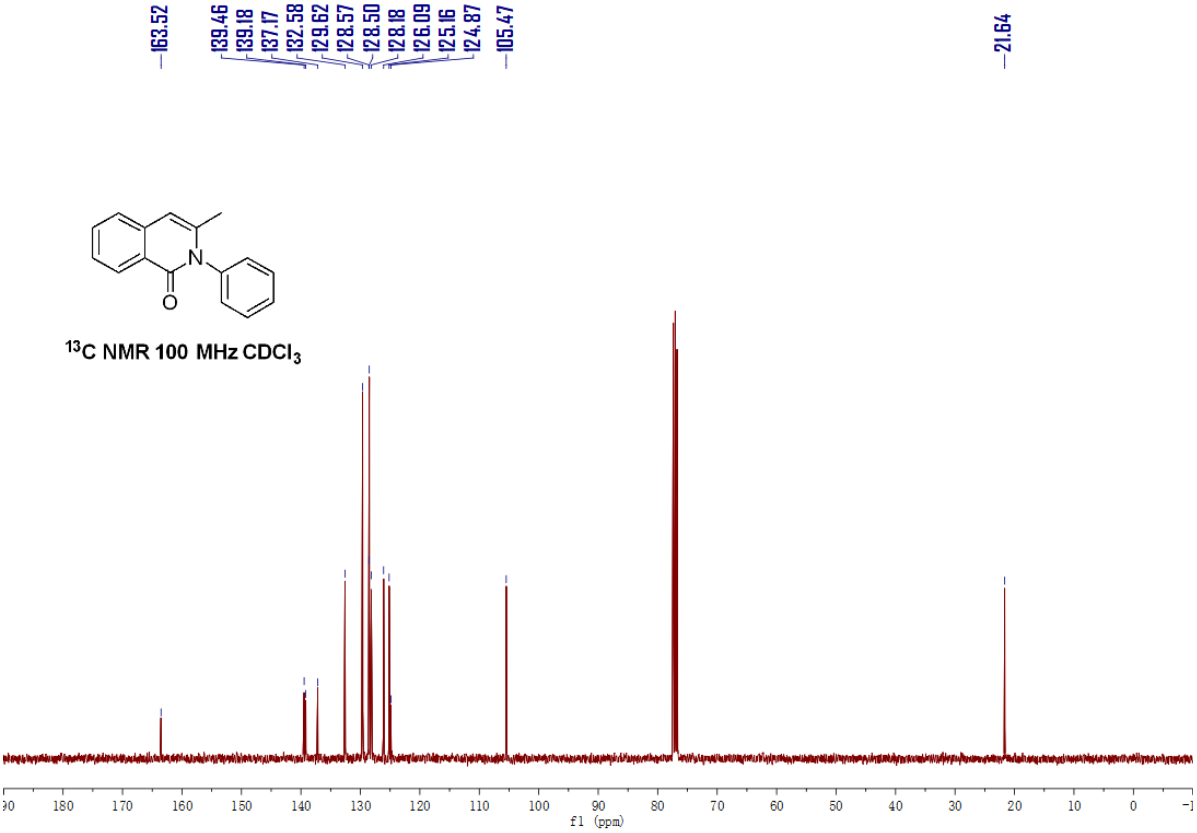
163.52
139.46
139.18
137.17
132.58
129.62
128.57
128.50
128.18
126.09
125.16
124.87
105.47
21.64
N
O
13C NMR 100 MHz CDCl3
f1 (ppm)

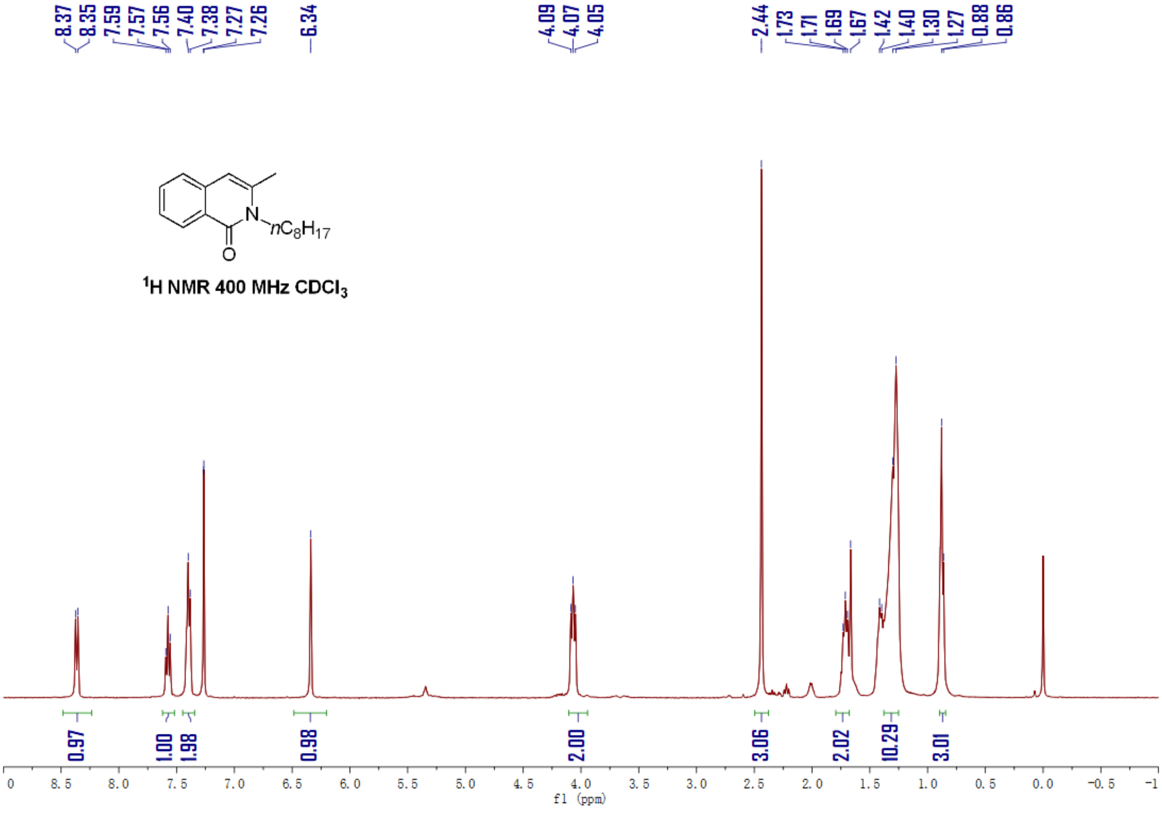
8.37
8.35
7.59
7.57
7.56
7.40
7.38
7.27
7.26
6.34
4.09
4.07
4.05
2.44
1.73
1.71
1.69
1.67
1.42
1.40
1.30
1.27
0.88
0.86
N
nC8H17
O
1H NMR 400 MHz CDCl3
0.97
1.00
1.98
0.98
2.00
3.06
2.02
10.29
3.01
f1 (ppm)

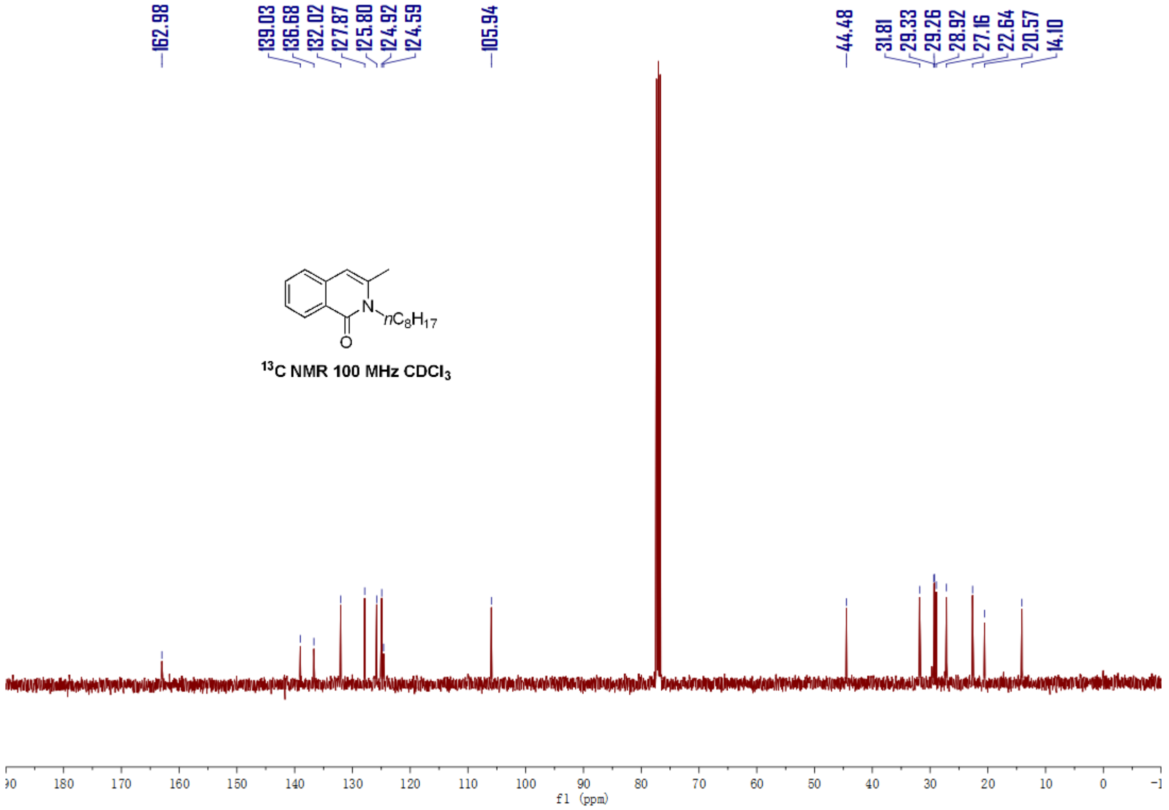

162.98
139.03
136.68
132.02
127.87
125.80
124.92
124.59
105.94
44.48
31.81
29.33
29.26
28.92
27.16
22.64
20.57
14.10
N
nC8H17
O
13C NMR 100 MHz CDCl3
f1 (ppm)
